# Discovery and Structure–Activity Relationship Studies of Novel Adenosine A_1_ Receptor-Selective Agonists

**DOI:** 10.1021/acs.jmedchem.2c01414

**Published:** 2022-10-21

**Authors:** Barbara Preti, Anna Suchankova, Giuseppe Deganutti, Michele Leuenberger, Kerry Barkan, Iga Manulak, Xianglin Huang, Sabrina Carvalho, Graham Ladds, Martin Lochner

**Affiliations:** † Institute of Biochemistry and Molecular Medicine, University of Bern, Bühlstrasse 28, 3012Bern, Switzerland; ‡ Department of Pharmacology, University of Cambridge, Tennis Court Road, CambridgeCB2 1PD, U.K.; § Centre for Sport, Exercise and Life Sciences, Faculty of Health and Life Sciences, Coventry University, CoventryCV1 5FB, U.K.

## Abstract

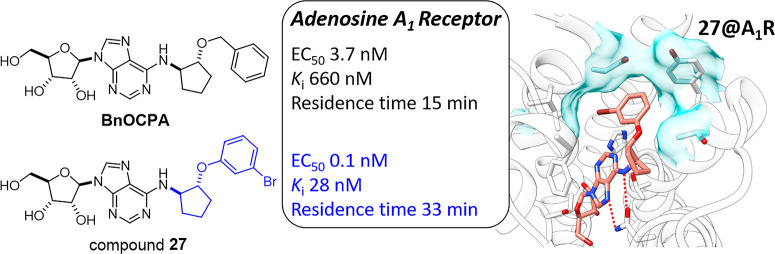

A series of benzyloxy and phenoxy derivatives of the adenosine receptor agonists *N*^6^-cyclopentyl adenosine (CPA) and *N*^6^-cyclopentyl 5′-*N*-ethylcarboxamidoadenosine (CP-NECA) were synthesized, and their potency and selectivity were assessed. We observed that the most potent were the compounds with a halogen in the *meta* position on the aromatic ring of the benzyloxy- or phenoxycyclopentyl substituent. In general, the NECA-based compounds displayed greater A_1_R selectivity than the adenosine-based compounds, with *N*^6^-2-(3-bromobenzyloxy)cyclopentyl-NECA and *N*^6^-2-(3-methoxyphenoxy)cyclopentyl-NECA showing ∼1500-fold improved A_1_R selectivity compared to NECA. In addition, we quantified the compounds’ affinity and kinetics of binding at both human and rat A_1_R using a NanoBRET binding assay and found that the halogen substituent in the benzyloxy- or phenoxycyclopentyl moiety seems to confer high affinity for the A_1_R. Molecular modeling studies suggested a hydrophobic subpocket as contributing to the A_1_R selectivity displayed. We believe that the identified selective potent A_1_R agonists are valuable tool compounds for adenosine receptor research.

## Introduction

The adenosine A_1_ receptor (A_1_R) is a G protein-coupled receptor (GPCR) that belongs to the adenosine receptor family consisting of four receptor subtypes (A_1_R, A_2A_R, A_2B_R, and A_3_R). All four receptor subtypes are nonselectively activated by the endogenous ligand adenosine, a naturally occurring purine nucleoside. The adenosine receptors are widely expressed in the body and therefore implicated in various pathological conditions including cancer; sleep regulation; and cardiovascular, neurodegenerative, and inflammatory diseases.^1−9^ The wide expression pattern has led to the reality that despite more than four decades of intense medicinal research, very few compounds have actually made it to the clinic due to unacceptable side effects based on insufficient subtype selectivity and/or low efficacy, leaving a big untapped need for subtype-selective compounds.^10,11^

The selective activation of the A_1_R, in particular, is a very promising strategy for the treatment of glaucoma, type 2 diabetes mellitus, pain, epilepsy, heart arrhythmias, and cerebral ischemia, in which there are clear unmet clinical needs that could be addressed with novel more selective therapeutics.^11,12^ Although all members of the adenosine receptor family are activated by endogenous adenosine, the A_1_R and A_3_R receptors are predominantly G_i/o_-coupled, while the A_2A_R and A_2B_R are predominantly G_s_-coupled. The classical pathway following G_i/o_ activation is the inhibition of adenylyl cyclase (AC) and subsequent inhibition of 3′,5′-cyclic adenosine monophosphate (cAMP) accumulation in the cell, while activation of G_s_ activates AC, resulting in the promotion of cAMP accumulation.

Several potent and A_1_R-selective agonists are based on the endogenous adenosine scaffold. Substitution at the purine C-2 position, e.g., with chloride, and at the *N*^6^ position with cycloalkyl- and bicycloalkyl groups has led to potent and A_1_R-selective agonists.^11−15^ In addition, the ribose moiety has been the focus of synthetic modifications in AR agonist development. The ribose C-5′ position tolerates certain substitutions, such as a 5′-carboxamido group in the prototypical, very potent, albeit not highly subtype-selective AR agonist 5′-*N*-ethylcarboxyamidoadenosine (NECA). Small 5′-chlorine substituents have also been used, and it was shown that they, together with *N*^6^-bicycloalkyl groups, lead to high-affinity and highly selective human A_1_R agonists, which have antinociceptive effects in mice without affecting motor or cardiovascular functions.^16,17^ More bulky pyrazole groups have also been employed at this C-5′ position and yielded potent and selective A_1_R agonists that showed analgesic effects in mice.^15^ Other successful selective and CNS active A_1_R agonist examples feature conformationally constrained ribose ring systems.^14^ Alternatively, non-nucleoside 3,5-dicyanopyridines have been synthetically optimized to yield potent and A_1_R-selective full agonists, which have also been developed into PET tracers very recently.^18^ Rather than modulating receptor activity by orthosteric exogenous agonists, Christopoulos and collaborators have recently presented MIPS521, a positive allosteric modulator of the A_1_R, with which they were able to show *in vivo* analgesic efficacy in rats.^19^ Their cryo-EM structural study of the human A_1_R bound to adenosine, MIPS521, and a G_i2_ protein heterotrimer (PDB code 7LD3) revealed the allosteric binding pocket at the lipid interface that could spark structure-based drug design campaigns.

**Chart 1:**
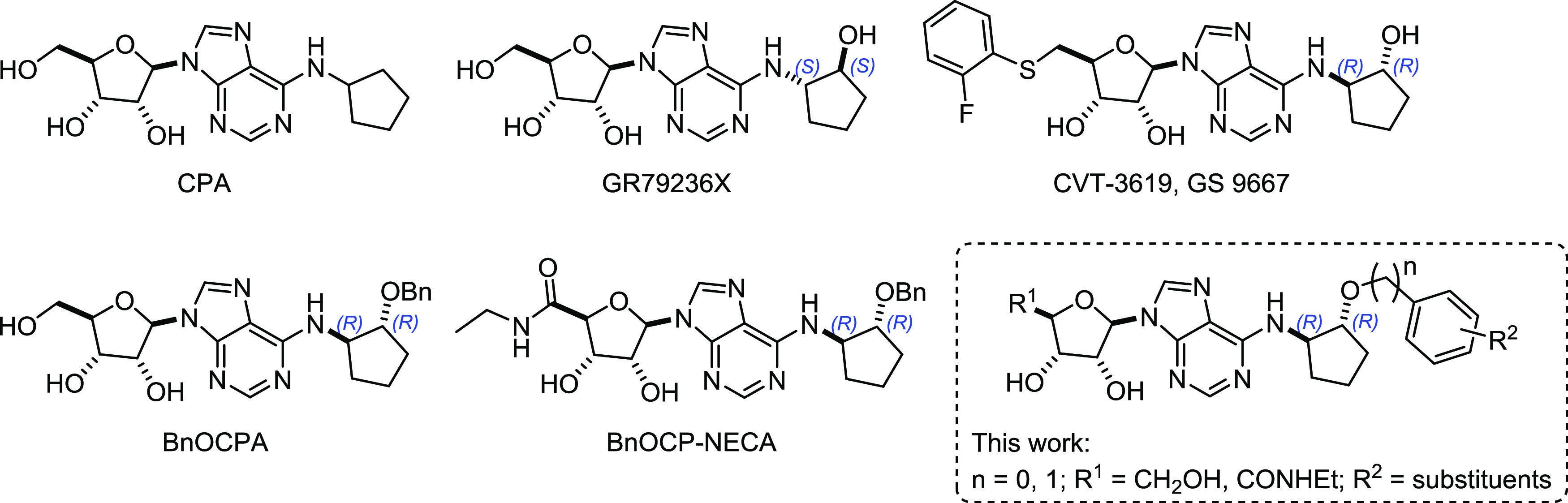
Known *N*^6^-Cyclopentyl Adenosine A_1_R Agonists, Previously Synthesized A_1_R Agonists, and Derivatives Presented in This Work

We have previously reported the adenosine-based potent and highly A_1_R-selective full agonist BnOCPA (Chart 1), which emerged from a structure–activity relationship (SAR) study with respect to cyclic and bicyclic purine *N*^6^ substituents.^13^ Our SAR study also showed that the synthetic NECA derivatives, such as BnOCP-NECA (Chart 1), were generally less subtype-selective than the adenosine ones. BnOCPA and BnOCP-NECA are extension derivatives of the prototypical, non-subtype-selective AR agonist *N*^6^-cyclopentyl adenosine (CPA). The *N*^6^-hydroxycyclopentyl moiety is present in the known A_1_R-selective partial agonist CVT-3619 (later renamed GS 9667) and full agonist GR79236X. It should be noted that the stereochemical configuration of the *N*^6^-hydroxycyclopentyl group in GR79236X is opposite to that in CVT-3619 and BnOCPA (Chart 1). Both GR79236X and CVT-3619 are able to increase insulin sensitivity and thus have been evaluated in clinical trials for the treatment of type II diabetes; however, their development was not successful and later discontinued.^20,21^ Appending a benzyl group to the *N*^6^-hydroxylcyclopentyl moiety, we have found previously that BnOCPA retained high potency at A_1_R and displayed very high A_1_R selectivity compared to the nonbenzylated congener.^13^

Subsequently, we demonstrated that BnOCPA was able to specifically activate Gα_ob_ protein subtype-mediated signaling, which translated into potent *in vivo* analgesia without causing sedation, bradycardia, hypotension, or respiratory depression.^22^ Molecular dynamics (MD) simulations using the cryo-EM structure of the active adenosine-bound A_1_R-heterotrimeric G_i2_ protein complex (PDB code 6D9H(23)) proposed four binding modes of BnOCPA^22^ due to the high flexibility of the *N*^6^-appended benzyloxy group.^24^

Based on these molecular modeling studies, we have designed a series of adenosine- and NECA-based compounds with extended *N*^6^-benzyloxy- and *N*^6^-phenoxycyclopentyl substituents (Chart 1) with the aim of improving the potency at A_1_R while maintaining or improving the subtype selectivity. To test the potency, selectivity, and affinity of the designed compounds at the adenosine receptors, we have employed the cAMP accumulation and NanoBRET binding assays previously validated and employed at ARs.^25^ We explored subtype selectivity at human A_1_R, A_2A_R, A_2B_R, and A_3_R in mammalian CHO-K1 cells and confirmed the binding of the compounds at both human and rat A_1_R in HEK293 cells. Kinetic studies were performed to dissect the binding properties of the compounds to hA_1_R and rA_1_R and outline their structure–kinetic relationship (SKR). Finally, we have also used MD modeling to evaluate the binding pose of some of the agonists and validated the findings using mutagenesis. Together, this approach has identified novel adenosine- and NECA-based derivatives with improved potency, selectivity, and affinity at the A_1_R receptor. Hence, these compounds constitute valuable tools for cellular studies of the A_1_R receptor and show interesting therapeutic promise.

## Results and Discussion

### Chemistry

Our initial synthetic strategy was designed for keeping the route as concise as possible and entailed a projected *O*-alkylation of ribose-protected *N*^6^-hydroxycyclopentyl adenosine and NECA precursors. After exploring different *O*-alkylation protocols and ribose hydroxyl protecting groups, we abandoned this synthetic plan, as our attempts resulted in low conversions, trace amounts of desired products, and many side products stemming from the loss or migration of protecting groups, elimination reactions, and *N*-alkylation of amine and amide groups (Scheme S1).

**Scheme 1:**
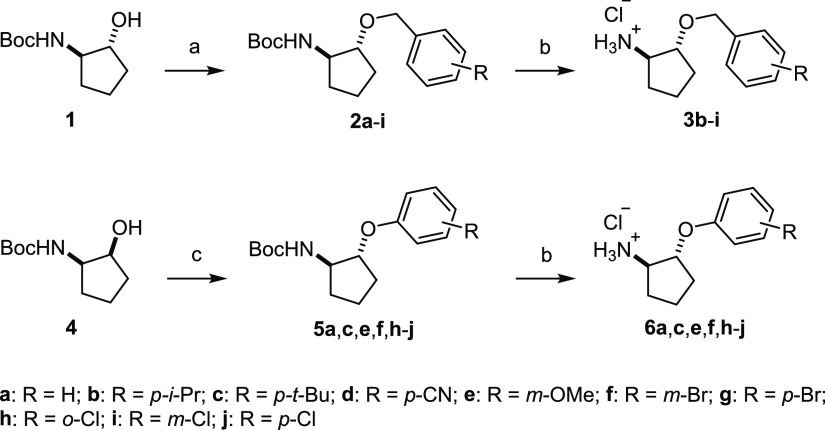
Synthesis of Benzyloxycyclopentyl and Phenoxycyclopentyl Amine Building Blocks Reagents and conditions: (a) R-BnBr (1 equiv), NaH (2 equiv), THF, 0 °C, 2–4 h, 30–91%; (b) HCl (4 M in dioxane), 1,4-dioxane, rt, 4–18 h, 50%-quant; and (c) R-PhOH, PPh_3_, DIAD, THF, 0 °C to rt, 18 h, 51–62%.

We therefore adapted our strategy and carried out the *O*-alkylation on *N*-protected (1*R*,2*R*)-2-aminocyclopentanol **1**(26) (Scheme 1). Under optimized conditions, treating a mixture of **1** and benzyl bromide (1 equiv) in THF at 0 °C with sodium hydride (2 equiv) yielded 58% of desired **2a** after 4 h. It was important to monitor this reaction carefully, as after a certain time (2–4 h), side products started to emerge that diminished the isolated yields. These optimized conditions were applied for the preparation of benzyloxycyclopentyl intermediates **2b**–**i**, which were isolated in moderate to very good yields (30–91%).

For the introduction of the phenoxy substituents on the cyclopentyl ring, we first tosylated epimeric *N*-protected (1*S*,2*R*)-2-aminocyclopentanol **4** (*p*-TsCl, pyridine, rt, 24 h, 57% yield), which was followed by S_N_2-type substitution with phenoxide (Figure S1). However, the latter reaction required forcing conditions (phenol, K_2_CO_3_, DMF, 70 °C, 3 days) to obtain **5a** with the desired (1*R*,2*R*) stereochemistry in acceptable 61% yield. We therefore sought a milder, more efficient method and hypothesized that the Mitsunobu reaction^27^ might allow us to directly access protected phenoxycyclopentyl amines **5** from **4** (Scheme 1). The Mitsunobu reaction is commonly used to convert primary and secondary alcohols to a variety of functional groups with inversion at the alcohol stereogenic center and requires an acidic nucleophile (e.g. carboxylic acids). In rare cases, phenols (p*K*_a_ ∼9–10) were employed as nucleophiles, but to the best of our knowledge, cyclic secondary alcohols were not reported as Mitsunobu substrates to date. We were therefore pleased to find that adding diisopropyl azodicarboxylate (DIAD) to a solution of **4**, phenol, and triphenylphosphine at 0 °C and subsequent stirring at room temperature overnight delivered **5a** in 60% yield. ^1^H NMR spectra of **5a** obtained via Mitsunobu reaction and of **5a** isolated after S_N_2 phenoxide substitution of tosylated **4** were identical, confirming full inversion at C-1 during the Mitsunobu reaction (Figure S1). For comparison, reaction with epimeric **1** under identical Mitsunobu conditions yielded the C-1 epimer of **5a** with a distinctively different ^1^H NMR spectrum. Overall, our new synthetic strategy efficiently produced phenoxycyclopentyl building block **5** in moderate to good yields. Clean removal of the Boc-protecting group from **2** and **5** was achieved with HCl in dioxane to deliver ammonium salts **3** and **6** (Scheme 1).

**Scheme 2:**
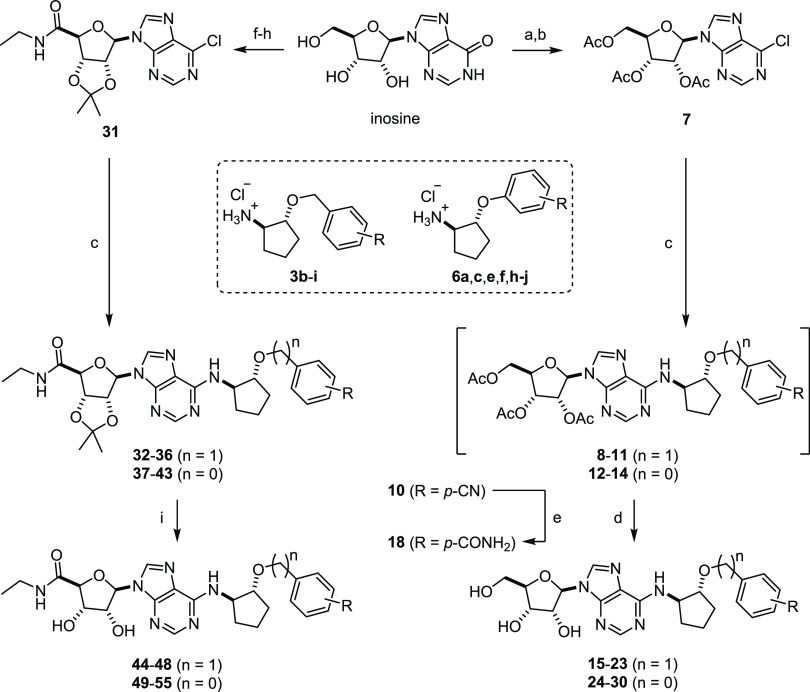
Synthesis of Benzyloxy- and Phenoxycyclopentyl Adenosine and NECA Derivatives Reagents and conditions: (a) Ac_2_O, pyridine, rt 18 h, quant.; (b) SOCl_2_, DMF, CH_2_Cl_2_, 50 °C, 18 h, 86%; (c) **3b**–**i** or **6a**,**c**,**e**,**f**,**h**–**j**, NaHCO_3_, *i-*PrOH, 105 °C, 18 h, 36%-quant.; (d) K_2_CO_3_, MeOH, rt, 30 min, 36–93%; (e) K_2_CO_3_, H_2_O_2_ (aq. 30%), MeOH, 40 °C, 7 h, 83%; (f) 2,2-dimethoxypropane, *p*-TsOH, acetone, rt, 18 h, 32%; (g) TEMPO, DAIB, MeCN/H_2_O, rt, 18 h, 80%; (h) SOCl_2_, DMF, CH_2_Cl_2_, 50 °C, 18 h and then EtNH_2_ (2 M in THF), 0 °C to rt, 30 min, 41%; and (i) AcOH, H_2_O, 80 °C, 18 h, 43–73%.

Nucleophilic aromatic substitution (S_N_Ar) of 6-chloropurines **7** and **31** with amines **3** and **6** assembled the final agonist scaffolds (Scheme 2). Chloropurines **7** and **31** were synthesized starting from inosine using procedures adopted from Kotra *et al*.^28^ and Middleton *et al*.,^29^ with minor experimental modifications. Removal of the acetate groups was carried out with potassium carbonate in methanol at room temperature, while removal of the ribose acetonide group was accomplished with acetic acid in water at 80 °C, yielding final adenosine derivatives **15**–**30** and NECA derivatives **44**–**55** in high purity and sufficient quantity.

In the adenosine series, we observed partial cleavage of the acetate groups during the S_N_Ar reaction for some substrates, which led to complex but separable mixtures of desired nucleosides **8**–**14** and various deacetylated side products. We found that it was more convenient to take these crude mixtures into the subsequent deprotection step and isolate the fully deacetylated final products **15**–**30** (Scheme 2). Primary carboxamide derivative **18** was obtained from protected nitrile nucleoside **10** through hydrolysis with basic hydrogen peroxide in methanol at elevated temperatures.^30^

### Biological Activity at the Human A_1_ Receptor

BnOCPA has previously been identified as a high-potency A_1_R-selective full agonist.^13,22^ Using insights from BnOCPA MD simulations^22,24^ and with the aim of further improving the A_1_R selectivity and potency, we designed extended BnOCPA derivatives **15**–**18**. Their binding and activity at human A_1_R (hA_1_R) were then explored using both a NanoBRET binding assay and a cAMP accumulation assay, respectively (Table 1). To determine the A_1_R mediated G_i/o_ response, CHO-K1-A_1_R cells were co-stimulated for 30 min with 10 μM forskolin (which promotes cAMP production by activating adenylyl cyclase), and the test compounds **15**–**18** were added in a range of concentrations (10^–13^ to 10^–4^ M).

**Table 1:**
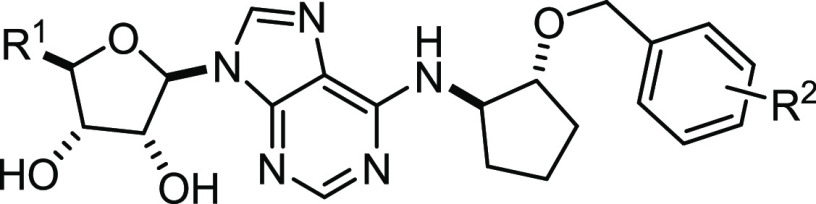
Affinity (p*K*_i_) and Potency (pEC_50_) of Extended BnOCPA Derivatives at Human A_1_R^a^

compd	R^1^	R^2^	pEC_50_ (hA_1_R)^b^	*E*_max_^c^	p*K*_i_ (hA_1_R)^d^
BnOCPA	–CH_2_OH	H	8.43 ± 0.09	51.49 ± 1.9	6.18 ± 0.09
**15**	–CH_2_OH	*p*-*i-*Pr	7.87 ± 0.16	51.95 ± 3.1	5.77 ± 0.08
**16**	–CH_2_OH	*p*-*t-*Bu	8.20 ± 0.13	54.59 ± 2.8	5.58 ± 0.10
**17**	–CH_2_OH	*p*-CN	7.40 ± 0.19	58.03 ± 4.2	5.85 ± 0.06
**18**	–CH_2_OH	*p*-CONH_2_	7.71 ± 0.13	59.51 ± 4.1	5.98 ± 0.05

a Data are the mean ± SEM of at least three independent repeats conducted in duplicate.

b The negative logarithm of the agonist concentration required to produce a half-maximal inhibition response of the 10 μM forskolin-induced cAMP accumulation in CHO-K1-A_1_R cells.

c The % maximal inhibition of cAMP accumulation for each agonist. Calculated as the % inhibition of the 10 μM forskolin response.

d Binding affinity (p*K*_i_) determined through the NanoBRET binding assay in HEK293 cells stably expressing human Nluc-A_1_R. The resulting concentration-dependent decrease in NanoBRET ratio at 10 min was used to calculate p*K*_i_. Statistical significance (*p < 0.05) determined using one-way ANOVA and Dunnett’s post-test and presented as described by Curtis *et al*.^31^

**Figure 1:**
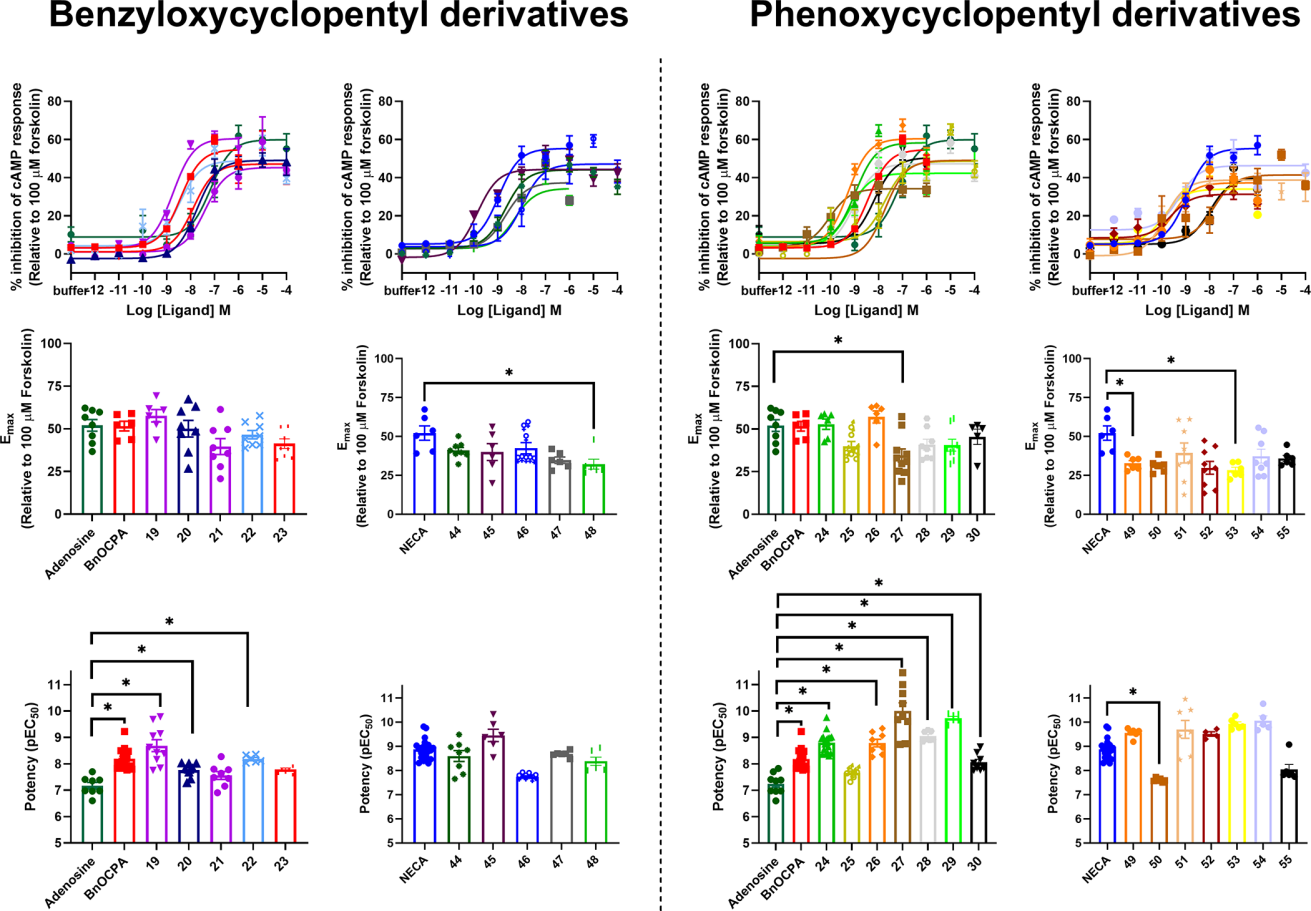
Efficacy and potency of synthetic benzyloxy- and phenoxycyclopentyl adenosine and NECA derivatives at A_1_R. cAMP response in CHO-K1 cells stably expressing human A_1_R in response to varying concentrations of AR ligands and 10 μM forskolin. *E*_max_ and pEC_50_ values for individual repeats are plotted at the bottom. Data are the mean ± SEM of at least three independent repeats conducted in duplicate. Statistical significance (*p < 0.05) determined using one-way ANOVA and Dunnett’s post-test, presented as described in ref (31).

All four compounds were found to be agonists at the hA_1_R using the inhibiting forskolin-stimulated cAMP accumulation assay with equivalent *E*_max_ values to that of the full agonist BnOCPA. **16** showed the highest potency with pEC_50_ of 8.20 ± 0.13, which was similar to BnOCPA (pEC_50_ of 8.43 ± 0.09). **15**–**18** were further tested for their ability to displace the specific binding of CA200645, a fluorescent A_1_R/A_3_R antagonist, in HEK293 cells stably expressing an N-terminally tagged human Nanoluc-hA_1_R (Nluc-hA_1_R), as described previously.^25^ All four explored BnOCPA derivative compounds **15**–**18** displayed similar affinity for Nluc-hA_1_R with *K*_i_ in the range of 1–3 μM, which remained similar or lower than BnOCPA (*K*_i_ of 0.66 μM). Since none of derivatives **15**–**18** improved upon BnOCPA potency or affinity at the A_1_R, we have decided to not continue with these compounds further and instead designed a new series of compounds based on adenosine (**19**–**30**) and their structural analogs based on NECA (**44**–**55**). Full cAMP inhibition curves in the CHO-K1-hA_1_R cells were obtained as described above (Figure 1, Tables 2 and 3). Except for **27**, **48**, **49**, and **53**, which showed partial activity, all the tested compounds behaved as full agonists at the hA_1_R. **27** was the most potent (pEC_50_ of 10.0 ± 0.24), closely followed by **26**, **45**, **49**, and **51**–**54**. Furthermore, all these compounds displayed a higher potency than adenosine, NECA, or BnOCPA, making them very promising candidate compounds. It is interesting to note that, except for **49** and **53**, the most potent compounds have a substituent in the *meta* position and, except for **26**, **49** and **51**, all have a halogen substituent. Therefore, it seems that a halogen in the *meta* position on the aromatic ring confers high potency at the hA_1_R. In addition, all most potent hA_1_R agonists except **45** feature a *N*^6^-phenoxycyclopentyl moiety.

**Table 2:**
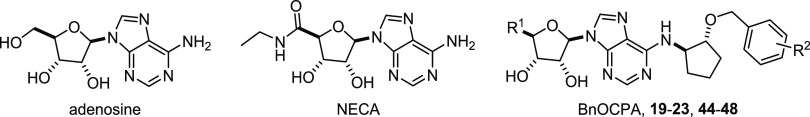
Affinity (p*K*_i_) and Potency (pEC_50_) of Synthetic Adenosine and NECA Benzyloxycyclopentyl Derivatives

compd	R^1^	R^2^	hA_1_R		hA_2A_R		hA_2B_R		hA_3_R		hA_1_R	rA_1_R
			pEC_50_^a^	*E*_max_^b^	pEC_50_^a^	*E*_max_^c^	pEC_50_^a^	*E*_max_^c^	pEC_50_^a^	*E*_max_^b^	p*K*_i_^d^	p*K*_i_^d^
adenosine			7.16 ± 0.23	51.09 ± 4.9	7.60 ± 0.11	21.53 ± 0.9	7.28 ± 0.12	59.07 ± 2.9	7.87 ± 0.23	24.71 ± 2.5	6.09 ± 0.06	6.06 ± 0.05
BnOCPA	–CH_2_OH	H	8.43 ± 0.09*	51.49 ± 1.9	4.95 ± 0.38*	17.13 ± 4.6	N.D.^e^		N.R.^f^		6.18 ± 0.09	6.41 ± 0.06*
**19**	–CH_2_OH	*m*-OMe	8.74 ± 0.10*	57.41 ± 2.4	N.D.		N.D.		5.98 ± 0.47*	9.52 ± 2.1*	6.67 ± 0.10*	6.55 ± 0.06*
**20**	–CH_2_OH	*m*-Br	7.74 ± 0.13*	51.44 ± 2.8	N.R.		N.D.		5.08 ± 0.26*	12.59 ± 1.8*	6.16 ± 0.10	6.13 ± 0.08
**21**	–CH_2_OH	*p*-Br	7.39 ± 0.16	44.25 ± 3.2	N.D.		N.D.		N.R.		5.94 ± 0.07	6.06 ± 0.07
**22**	–CH_2_OH	*o*-Cl	8.47 ± 0.24*	44.66 ± 4.3	5.17 ± 0.33*	18.55 ± 3.5	4.57 ± 0.12*	96.93 ± 8.1*	N.R.		6.56 ± 0.07*	6.56 ± 0.04*
**23**	–CH_2_OH	*m*-Cl	7.88 ± 0.17	45.97 ± 3.3	N.D.		N.D.		N.R.		6.15 ± 0.07	6.43 ± 0.03*
NECA			8.96 ± 0.11	50.11 ± 2.4	7.95 ± 0.26	22.03 ± 2.4	7.20 ± 0.07	68.12 ± 2.0	7.83 ± 0.26	34.34 ± 3.7	6.61 ± 0.06	6.38 ± 0.04
**44**	–CONHEt	*m*-OMe	8.67 ± 0.19	41.15 ± 3.3	N.R.		4.42 ± 0.16*	73.07 ± 8.9	5.38 ± 0.09*	46.56 ± 2.1	6.39 ± 0.08	6.11 ± 0.07*
**45**	–CONHEt	*m*-Br	9.85 ± 0.19	46.12 ± 5.2	N.R.		N.D.		5.56 ± 0.10*	49.17 ± 2.4*	6.54 ± 0.15	6.46 ± 0.07
**46**	–CONHEt	*p*-Br	7.97 ± 0.24	43.32 ± 4.5	N.D.		N.D.		5.82 ± 0.20*	30.56 ± 2.8	6.15 ± 0.06*	6.38 ± 0.03
**47**	–CONHEt	*o*-Cl	8.67 ± 0.15	34.67 ± 2.2	5.68 ± 0.32	20.34 ± 3.1	5.36 ± 0.08*	87.58 ± 3.3	5.85 ± 0.13*	43.92 ± 2.8	6.63 ± 0.07	6.85 ± 0.05*
**48**	–CONHEt	*m*-Cl	8.29 ± 0.16	31.57 ± 2.1*	5.04 ± 0.29*	17.18 ± 2.9	4.72 ± 0.11*	96.14 ± 6.8*	5.83 ± 0.39*	31.06 ± 5.7	6.49 ± 0.08	6.90 ± 0.05*

a The negative logarithm of the agonist concentration required to produce a half-maximal response in the cAMP accumulation assay in CHO-K1 cells stably expressing a human AR subtype. Forskolin is included in the assay (10 and 1 μM for A_1_R and A_3_R, respectively).

b The % maximal inhibition of cAMP accumulation for each agonist. Forskolin is included in the assay (10 and 1 μM for A_1_R and A_3_R, respectively).

c The % maximal accumulation of each agonist relative to 10 μM forskolin stimulation.

d Binding affinity (p*K*_i_) determined through the NanoBRET binding assay in HEK293 cells stably expressing human or rat Nluc-A_1_R. The resulting concentration-dependent decrease in NanoBRET ratio at 10 min was used to calculate p*K*_i_.

e N.D., not determined. Full dose–response curve was not feasible.

f N.R., no response detected in the assay. All data are the mean ± SEM of at least three independent repeats conducted in duplicate. Statistical significance (*p < 0.05) determined using one-way ANOVA and Dunnett’s post-test, presented according to ref (31). Adenosine derivatives were compared to adenosine, while NECA derivatives were compared to NECA.

**Table 3:**
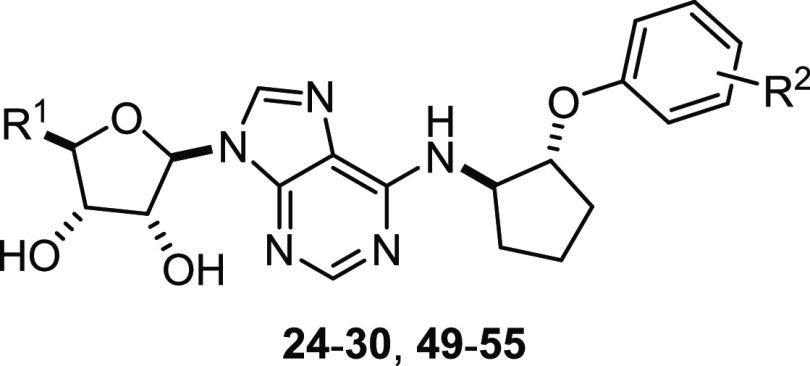
Affinity (p*K*_i_) and Potency (pEC_50_) of Synthetic Adenosine and NECA Phenoxycyclopentyl Derivatives

compd	R^1^	R^2^	hA_1_R		hA_2A_R		hA_2B_R		hA_3_R		hA_1_R	rA_1_R
			pEC_50_^a^	*E*_max_^b^	pEC_50_^a^	*E*_max_^c^	pEC_50_^a^	*E*_max_^c^	pEC_50_^a^	*E*_max_^b^	p*K*_i_^d^	p*K*_i_^d^
adenosine			7.16 ± 0.23	51.09 ± 4.9	7.60 ± 0.11	21.53 ± 0.9	7.28 ± 0.12	59.07 ± 2.9	7.87 ± 0.23	24.71 ± 2.5	6.09 ± 0.06	6.06 ± 0.05
**24**	–CH_2_OH	H	8.98 ± 0.14*	52.71 ± 3.1	N.D.^e^		4.90 ± 0.14*	64.19 ± 5.4	5.78 ± 0.42*	17.83 ± 3.7	6.84 ± 0.06*	6.60 ± 0.02*
**25**	–CH_2_OH	*p*-*t-*Bu	7.74 ± 0.28	42.14 ± 5.1	N.D.^f^		N.D.		N.R.		6.35 ± 0.08	6.70 ± 0.05*
**26**	–CH_2_OH	*m*-OMe	9.28 ± 0.10*	56.74 ± 2.4	5.24 ± 0.55*	17.25 ± 5.0	N.D.		N.R.		6.61 ± 0.07*	6.56 ± 0.06*
**27**	–CH_2_OH	*m*-Br	10.0 ± 0.24*	30.55 ± 3.3*	N.D.		4.63 ± 0.12*	82.53 ± 6.2	N.R.		7.55 ± 0.11*	6.94 ± 0.08*
**28**	–CH_2_OH	*o*-Cl	9.03 ± 0.19*	44.15 ± 3.6	5.96 ± 0.28*	17.01 ± 2.2	5.31 ± 0.09*	96.77 ± 4.4*	6.73 ± 0.42	13.42 ± 2.4*	7.17 ± 0.06*	7.28 ± 0.04*
**29**	–CH_2_OH	*m*-Cl	9.21 ± 0.19*	38.25 ± 3.0	6.26 ± 0.34	13.63 ± 2.1	5.29 ± 0.07*	96.93 ± 8.1*	6.81 ± 0.47	11.77 ± 2.3*	7.19 ± 0.07*	7.36 ± 0.03*
**30**	–CH_2_OH	*p*-Cl	8.19 ± 0.18*	45.06 ± 3.6	4.86 ± 0.58*	15.15 ± 5.5	N.D.		6.88 ± 0.60	12.5 ± 3.2*	6.23 ± 0.11	6.22 ± 0.06
NECA			8.96 ± 0.11	50.11 ± 2.4	7.95 ± 0.26	22.03 ± 2.4	7.20 ± 0.07	68.12 ± 2.0	7.83 ± 0.26	34.34 ± 3.7	6.61 ± 0.06	6.38 ± 0.04
**49**	–CONHEt	H	9.53 ± 0.20	32.68 ± 2.5*	5.48 ± 0.42*	15.41 ± 3.0	6.04 ± 0.09*	87.99 ± 3.7*	7.17 ± 0.16	51.24 ± 3.4*	7.30 ± 0.05*	7.41 ± 0.03*
**50**	–CONHEt	*p*-*t*-Bu	7.81 ± 0.41*	33.66 ± 6.1	4.84 ± 0.30*	20.11 ± 3.8	4.77 ± 0.08*	96.35 ± 5.0*	6.63 ± 0.19*	39.85 ± 3.2	6.35 ± 0.07	6.85 ± 0.05*
**51**	–CONHEt	*m*-OMe	9.88 ± 0.29	39.71 ± 5.9	5.20 ± 1.11*	5.16 ± 3.1*	5.10 ± 0.07*	84.76 ± 3.3	5.56 ± 0.14*	59.38 ± 4.0*	7.26 ± 0.14*	6.85 ± 0.06*
**52**	–CONHEt	*m*-Br	9.62 ± 0.35	39.71 ± 5.9	4.58 ± 0.87*	11.48 ± 6.7	5.37 ± 0.11*	61.12 ± 3.3	5.52 ± 0.12*	68.26 ± 3.8*	7.05 ± 0.16*	6.82 ± 0.10*
**53**	–CONHEt	*o*-Cl	9.91 ± 0.23	28.65 ± 2.7*	5.67 ± 0.46*	14.69 ± 3.2	6.22 ± 0.09*	80.91 ± 3.1	7.00 ± 0.19*	41.6 ± 3.3	7.39 ± 0.04*	7.60 ± 0.04*
**54**	–CONHEt	*m*-Cl	9.28 ± 0.28	34.67 ± 2.1	5.86 ± 0.41	13.32 ± 2.6	6.01 ± 0.09*	78.81 ± 3.4	6.79 ± 0.14*	44.20 ± 2.6	7.43 ± 0.05*	7.51 ± 0.06*
**55**	–CONHEt	*p*-Cl	7.99 ± 0.15	35.07 ± 2.5	4.86 ± 0.32*	25.01 ± 5.2	5.10 ± 0.09*	83.73 ± 4.5	6.92 ± 0.17*	47.16 ± 3.3	6.86 ± 0.10	7.08 ± 0.04*

a The negative logarithm of the agonist concentration required to produce a half-maximal response in the cAMP accumulation assay in CHO-K1 cells stably expressing a human AR subtype. Forskolin is included in the assay (10 and 1 μM for A_1_R and A_3_R, respectively).

b The % maximal inhibition of cAMP accumulation for each agonist. Forskolin is included in the assay (10 and 1 μM for A_1_R and A_3_R, respectively).

c The % maximal accumulation of each agonist relative to 10 μM forskolin stimulation.

d Binding affinity (p*K*_i_) determined through the NanoBRET binding assay in HEK293 cells stably expressing human or rat Nluc-A_1_R. The resulting concentration-dependent decrease in NanoBRET ratio at 10 min was used to calculate p*K*_i_.

e N.D., not determined. Full dose–response curve was not feasible.

f N.R., no response detected in the assay. All data are the mean ± SEM of at least three independent repeats conducted in duplicate. Statistical significance (*p < 0.05) determined using one-way ANOVA and Dunnett’s post-test, presented according to ref (31). Adenosine derivatives were compared to adenosine, while NECA derivatives were compared to NECA.

### Subtype Selectivity of Adenosine and NECA Derivatives

**Figure 2:**
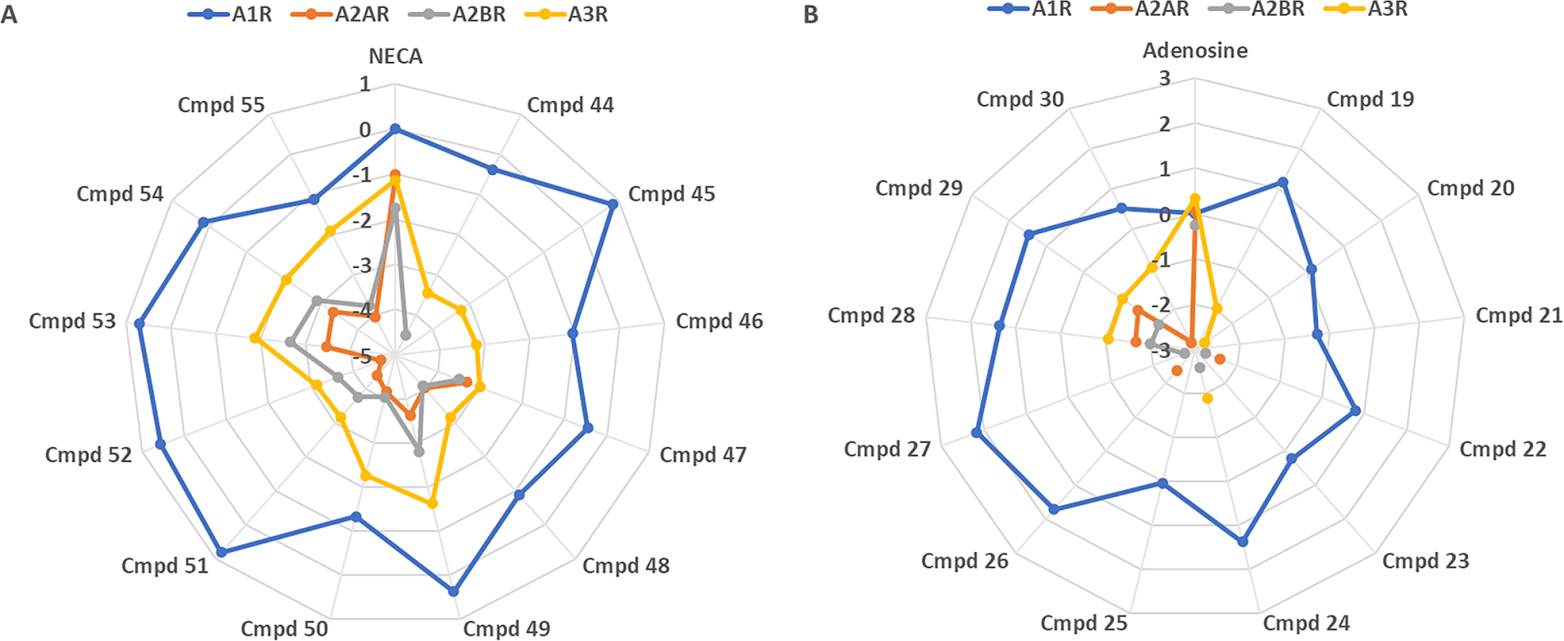
Adenosine and NECA derivatives show selectivity towards A_1_R subtype. Log(RA) values of AR ligands at human A_1_R, A_2A_R, A_2B_R, and A_3_R normalized to (A) NECA or (B) adenosine response at A_1_R.

As the structural similarity between the orthosteric site of the four adenosine receptor subtypes often results in reduced selectivity of the compounds targeting them, we utilized CHO-K1 cells stably expressing human A_2A_R, A_2B_R, or A_3_R (hA_2A_R, hA_2B_R, or hA_3_R) and incubated them with increasing concentrations of the tested compounds (10^–13^ to 10^–4^ M) to measure the cAMP accumulation in the cells in response to the agonists. For the G_i/o_-coupled hA_3_R, 1 μM forskolin was also included. This addition was not required for hA_2A_R or hA_2B_R since both are G_s_-coupled and thus stimulate cAMP production. All the tested compounds displayed only weak efficacy at either the hA_2A_R or hA_2B_R, with many failing to generate full dose-dependent response curves at the concentrations tested, resulting in adenosine and NECA remaining as the only potent compounds at these two receptors (Tables 2 and 3). At the hA_3_R, the adenosine derivatives showed either a loss of efficacy or partial activity, while all the NECA-based compounds (**44**–**55**) behaved as full agonists, although with reduced potency compared to NECA alone. To further assess the compound selectivity, we have calculated the relative activity (RA) for all agonists at the different receptor subtypes (Figure 2).

Overall, all the compounds display at least partial selectivity for hA_1_R except adenosine that is close to being an equipotent agonist at all the receptors. From the adenosine-based derivatives, compounds **22**, **23**, **26**, and **27** display the most hA_1_R selectivity, while compounds **28**–**30** also show activity at hA_3_R. However, with NECA itself being hA_1_R selective by ∼10-fold, it is the NECA-based compounds that display the highest hA_1_R selectivity, in particular compounds **44**, **45**, and **51**–**53**. Compounds **45** and **51** are ∼1500-fold more hA_1_R selective than NECA itself, suggesting >10,000-fold selectivity overall.

### Differences between Adenosine and NECA Derivatives

When comparing the adenosine and NECA analogs, the compounds based on NECA seem to be generally more potent at inhibiting cAMP accumulation at the hA_1_R receptor. Their potencies are either equivalent to or reduced compared to NECA at the other three AR subtypes. As a result, the NECA-based derivatives are more hA_1_R-selective than the adenosine derivatives. When we looked more closely at the adenosine and NECA-derived analog pairs, most of them displayed very similar selectivity across all AR subtypes (Tables 2 and 3). For example, adenosine-derived **29** and its NECA-derived analog **54** are both potent hA_1_R full agonists (pEC_50_ = 9.21 ± 0.19 and 9.28 ± 0.28, respectively), whereas **30** and **55** are relatively less potent dual hA_1_R and hA_3_R agonists (pEC_50_ = 8.19 ± 0.18, 7.99 ± 0.15 (hA_1_R) and 6.88 ± 0.60, 6.92 ± 0.17 (hA_3_R), respectively). Therefore, for these compounds, it seems to be the position of the substituent on the phenoxy group that has the most effect on compound selectivity. For some analog pairs, however, the patterns do not show such a close relationship. **27** and **52** are both potent agonists at hA_1_R (pEC_50_ = 10.0 ± 0.24 and 9.62 ± 0.35, respectively), but **52** also weakly activated hA_3_R (pEC_50_ = 5.52 ± 0.12), while **27** showed no response for this subtype. Consequently, in this case, the ribose C-5′ substituent group also affects the selectivity of the compounds, with the adenosine-derived compound being more hA_1_R selective.

### Kinetics of Binding of Adenosine and NECA Derivatives at Human and Rat A_1_R

**Figure 3:**
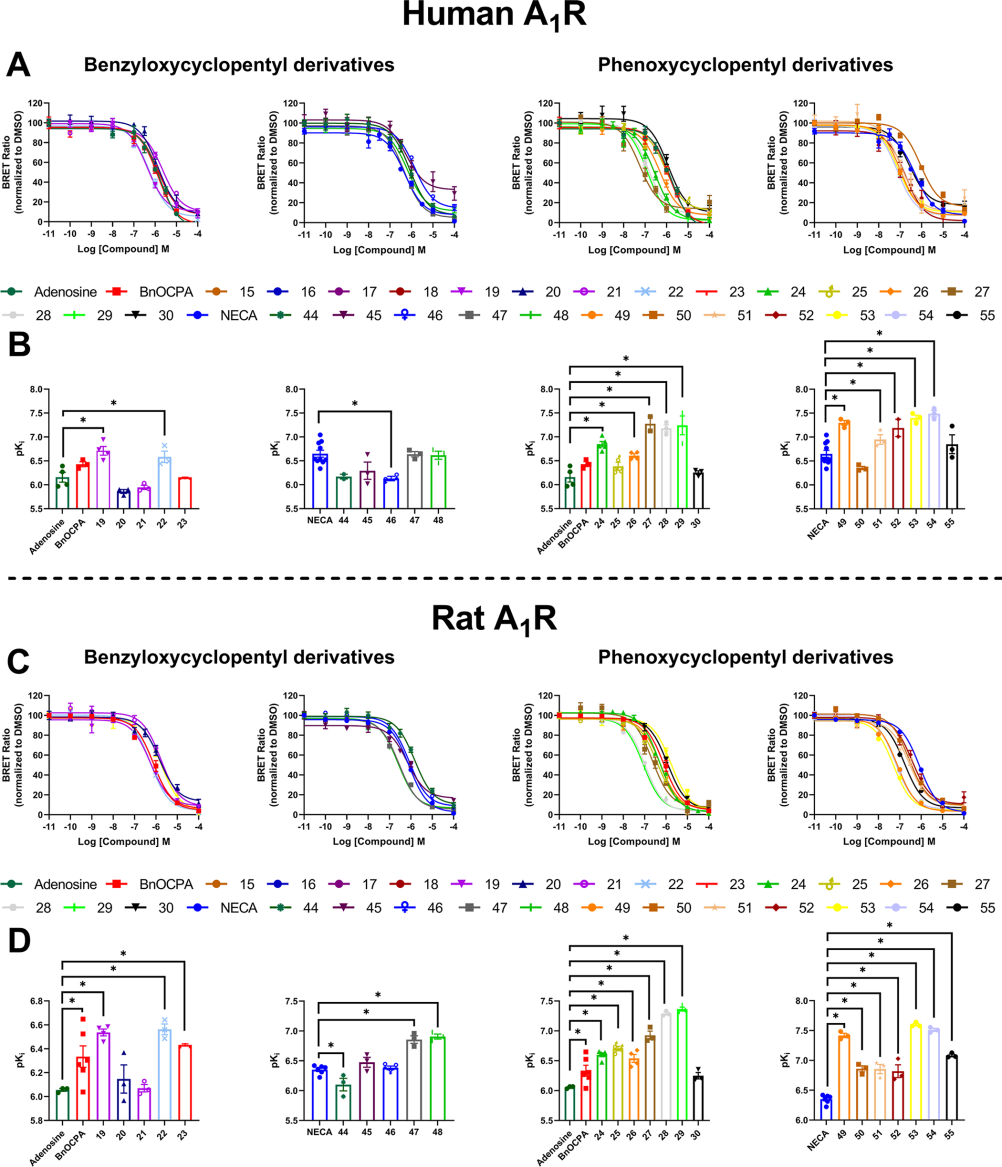
Binding affinity of AR ligands at human and rat A1R measured by NanoBRET. HEK293 cells stably expressing (A) human or (C) rat Nluc-A_1_R were treated with 20 nM CA200645 and increasing concentrations of unlabeled AR ligand. p*K*_i_ values for individual repeats from (B) human A Nluc-A_1_R and (D) rat Nluc-A_1_R. Data are the mean ± SEM of at least three independent repeats conducted in duplicate. Statistical significance (*p < 0.05) determined using one-way ANOVA and Dunnett’s post-test, presented as described in ref (31).

Since A_1_R agonists are promising compounds for the treatment of glaucoma, type 2 diabetes mellitus, pain, epilepsy, and cerebral ischemia, it is important to assess their binding properties at both human and rat A_1_R (rA_1_R), as the latter is commonly used as a model in preclinical studies.^2,11,12^ We have tested the compounds’ ability to displace the specific binding (at equilibrium) of CA200645 in HEK293 cells stably expressing human and rat Nluc-A_1_R (Figure 3, Tables 2 and 3).

The NanoBRET binding assay can also enable determination of real-time kinetics and affinities of the compound binding, as was previously described at the ARs.^25,32−34^ Values were derived using the ″kinetics of competitive binding″ model^35^ built into GraphPad Prism v9.1, enabling determinations of the compounds’ *k*_on_ and *k*_off_ values (Tables 4 and 5).

**Table 4:**
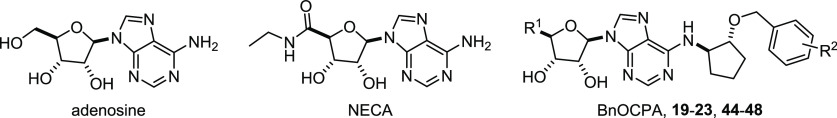
Kinetics of Binding for Synthetic Adenosine and NECA Benzyloxycyclopentyl Derivatives to the Orthosteric Binding Site at Human and Rat A_1_R

compd	R^1^	R^2^	hA_1_R				rA_1_R			
			*k*_on_ (*k*_3_) ×10^5^ (M^–1^ min^–1^)^a^	*k*_off_ (*k*_4_) (min^–1^)^b^	p*K*_d_^c^	RT (min)^d^	*k*_on_ (*k*_3_) ×10^5^ (M^–1^ min^–1^)^a^	*k*_off_ (*k*_4_) (min^–1^)^b^	p*K*_d_^c^	RT (min)^d^
adenosine			1.65 ± 0.24	0.048 ± 0.002	6.53 ± 0.07	21.15 ± 0.84	0.52 ± 0.22	0.079 ± 0.007	5.71 ± 0.17	12.86 ± 1.10
BnOCPA	–CH_2_OH	H	1.47 ± 0.33	0.068 ± 0.002	6.30 ± 0.09	14.75 ± 0.56	1.57 ± 0.54	0.054 ± 0.007	6.40 ± 0.11	19.42 ± 2.21
NECA			2.04 ± 0.07	0.049 ± 0.005	6.63 ± 0.06	20.97 ± 1.98	2.29 ± 0.39	0.066 ± 0.008	6.53 ± 0.04	15.84 ± 2.05
**19**	–CH_2_OH	*m*-OMe	2.51 ± 0.36	0.041 ± 0.004	6.77 ± 0.02	24.64 ± 1.98	2.52 ± 0.31	0.083 ± 0.016	6.49 ± 0.03	13.22 ± 2.10
**20**	–CH_2_OH	*m*-Br	0.56 ± 0.12	0.080 ± 0.016	5.84 ± 0.02	14.84 ± 3.83	1.50 ± 0.29	0.097 ± 0.008	6.17 ± 0.09	10.56 ± 0.81
**21**	–CH_2_OH	*p*-Br	0.66 ± 0.02	0.065 ± 0.010	6.02 ± 0.06	16.72 ± 3.01	1.13 ± 0.09	0.066 ± 0.010	6.24 ± 0.03	16.05 ± 2.09
**22**	–CH_2_OH	*o*-Cl	2.75 ± 0.14	0.055 ± 0.012	6.74 ± 0.10	22.22 ± 6.21	5.07 ± 0.26	0.090 ± 0.015	6.77 ± 0.06	12.10 ± 2.06
**23**	–CH_2_OH	*m*-Cl	1.40 ± 0.08	0.051 ± 0.015	6.48 ± 0.09	24.12 ± 5.29	2.85 ± 0.22	0.060 ± 0.006	6.68 ± 0.06	17.13 ± 1.54
**44**	–CONHEt	*m*-OMe	1.12 ± 0.09	0.110 ± 0.024	6.04 ± 0.10	10.59 ± 2.34	1.96 ± 0.13	0.141 ± 0.018	6.15 ± 0.06	7.41 ± 0.82
**45**	–CONHEt	*m*-Br	0.83 ± 0.06	0.095 ± 0.011	5.94 ± 0.02	10.78 ± 1.28	2.95 ± 0.41	0.121 ± 0.012	6.38 ± 0.06	8.51 ± 0.82
**46**	–CONHEt	*p*-Br	1.01 ± 0.06	0.076 ± 0.007	6.13 ± 0.03	13.55 ± 1.36	2.14 ± 0.14	0.058 ± 0.003	6.56 ± 0.01	17.30 ± 1.02
**47**	–CONHEt	*o*-Cl	3.64 ± 0.33	0.044 ± 0.009	6.94 ± 0.08	25.70 ± 4.63	7.31 ± 0.61	0.046 ± 0.002	7.20 ± 0.03	21.98 ± 1.13
**48**	–CONHEt	*m*-Cl	1.80 ± 0.17	0.040 ± 0.006	6.67 ± 0.05	27.96 ± 5.57	7.54 ± 0.30	0.073 ± 0.004	7.02 ± 0.03	13.90 ± 0.72

a *k*_on_ (*k*_3_) for ligands as determined using NanoBRET binding assays using either human or rat Nluc-A_1_R expressing HEK 293 cells and determined through fitting with the ″kinetics of competitive binding″ model.^35^

b *k*_off_ (*k*_4_) for ligands determined as in footnote *a*.

c Kinetic dissociation constant (p*K*_d_) for each ligand as determined from *k*_off_/*k*_on_.

d Residence time of each ligand as determined by the reciprocal of the *k*_off_. All data are the mean ± SEM of at least three independent repeats conducted in duplicate.

**Table 5:**
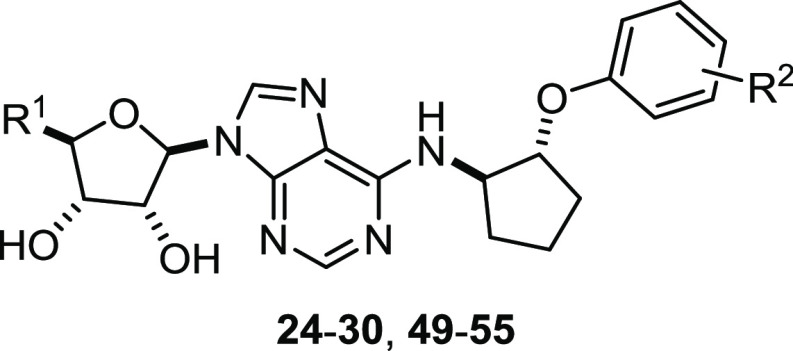
Kinetics of Binding for Synthetic Adenosine and NECA Phenoxycyclopentyl Derivatives to the Orthosteric Binding Site at Human and Rat A_1_R

compd	R^1^	R^2^	hA_1_R				rA_1_R			
			*k*_on_ (*k*_3_) ×10^5^ (M^–1^ min^–1^)^a^	*k*_off_ (*k*_4_) (min^–1^)^b^	p*K*_d_^c^	RT (min)^d^	*k*_on_ (*k*_3_) ×10^5^ (M^–1^ min^–1^)^a^	*k*_off_ (*k*_4_) (min^–1^)^b^	p*K*_d_^c^	RT (min)^d^
**24**	–CH_2_OH	H	4.78 ± 0.93	0.048 ± 0.004	6.97 ± 0.06	21.56 ± 2.13	2.70 ± 0.34	0.053 ± 0.001	6.70 ± 0.06	18.91 ± 0.30
**25**	–CH_2_OH	*p*-*t-*Bu	1.48 ± 0.04	0.054 ± 0.005	6.44 ± 0.03	19.12 ± 2.01	4.84 ± 0.37	0.484 ± 0.173	6.17 ± 0.30	5.53 ± 3.31
**26**	–CH_2_OH	*m*-OMe	2.97 ± 0.27	0.037 ± 0.003	6.90 ± 0.01	27.61 ± 2.09	3.32 ± 0.52	0.085 ± 0.007	6.58 ± 0.03	12.06 ± 1.03
**27**	–CH_2_OH	*m*-Br	10.12 ± 1.29	0.038 ± 0.012	7.47 ± 0.09	33.13 ± 10.40	13.30 ± 1.21	0.050 ± 0.005	7.42 ± 0.03	20.40 ± 1.86
**28**	–CH_2_OH	*o*-Cl	11.61 ± 1.31	0.052 ± 0.004	7.34 ± 0.04	19.55 ± 1.48	17.92 ± 1.30	0.048 ± 0.005	7.58 ± 0.01	21.46 ± 2.08
**29**	–CH_2_OH	*m*-Cl	25.90 ± 2.14	0.040 ± 0.003	7.81 ± 0.02	25.37 ± 1.70	30.07 ± 4.02	0.071 ± 0.010	7.63 ± 0.12	15.20 ± 2.50
**30**	–CH_2_OH	*p*-Cl	1.01 ± 0.31	0.056 ± 0.009	6.22 ± 0.13	19.46 ± 3.34	2.51 ± 0.90	0.056 ± 0.001	6.58 ± 0.15	17.90 ± 0.25
**49**	–CONHEt	H	16.43 ± 2.81	0.028 ± 0.002	7.63 ± 0.01	32.82 ± 1.02	25.12 ± 2.08	0.037 ± 0.002	7.83 ± 0.05	27.61 ± 1.66
**50**	–CONHEt	*p*-*t*-Bu	1.53 ± 0.11	0.091 ± 0.010	6.23 ± 0.06	11.47 ± 1.48	6.29 ± 0.62	0.070 ± 0.007	6.95 ± 0.01	14.76 ± 1.64
**51**	–CONHEt	*m*-OMe	4.27 ± 0.03	0.027 ± 0.007	7.23 ± 0.11	42.68 ± 10.88	11.91 ± 1.33	0.030 ± 0.006	7.62 ± 0.11	38.05 ± 8.74
**52**	–CONHEt	*m*-Br	5.57 ± 2.56	0.075 ± 0.038	6.91 ± 0.04	27.87 ± 14.23	4.46 ± 1.28	0.056 ± 0.008	6.85 ± 0.13	19.17 ± 2.68
**53**	–CONHEt	*o*-Cl	17.27 ± 1.12	0.041 ± 0.010	7.66 ± 0.11	29.44 ± 6.91	37.59 ± 1.91	0.048 ± 0.003	7.90 ± 0.00	21.23 ± 1.22
**54**	–CONHEt	*m*-Cl	20.28 ± 1.11	0.043 ± 0.013	7.75 ± 0.16	34.70 ± 13.43	35.85 ± 3.95	0.062 ± 0.007	7.76 ± 0.10	16.78 ± 2.22
**55**	–CONHEt	*p*-Cl	3.75 ± 1.04	0.076 ± 0.024	6.71 ± 0.03	17.48 ± 4.79	11.62 ± 0.26	0.064 ± 0.003	7.26 ± 0.01	15.65 ± 0.78

a *k*_on_ (*k*_3_) for ligands as determined using NanoBRET binding assays using either human or rat Nluc-A_1_R expressing HEK 293 cells and determined through fitting with the ″kinetics of competitive binding″ model.^35^

b *k*_off_ (*k*_4_) for ligands determined as in footnote *a*.

c Kinetic dissociation constant (p*K*_d_) for each ligand as determined from *k*_off_/*k*_on_.

d Residence time of each ligand as determined by the reciprocal of the *k*_off_. All data are the mean ± SEM of at least three independent repeats conducted in duplicate.

The reciprocal of the *k*_off_ enables a determination of the residence time (RT) of a compound.^25^ RT is a quantification of the time a ligand spends bound to the receptor, and it is increasingly considered in drug design because of its correlation with pharmacodynamics.^36^ Beyond this, we also determined the p*K*_d_ of the compounds (*k*_off_*/k*_on_) from the kinetics assays and compared these values to those determined from the saturation binding assays. The kinetic parameters for CA200645 binding at the human Nluc-A_1_R were determined as *k*_on_ (*k*_1_) = 3.67 ± 0.34 × 10^6^ M^–1^ min^–1^ and *k*_off_ (*k*_2_) = 0.064 ± 0.0023 min^–1^ with a *K*_d_ = 18.29 ± 2.4 nM. For the rA_1_R, the kinetics of binding for CA200645 were determined as *k*_on_ (*k*_1_) = 2.93 ± 0.24 × 10^6^ M^–1^ min^–1^ and *k*_off_ (*k*_2_) = 0.066 ± 0.0022 min^–1^ with a *K*_d_ = 32.96 ± 2.8 nM. With the help of these parameters, we were then able to provide estimates of the kinetics of binding for adenosine and NECA benzyloxycyclopentyl and phenoxycyclopentyl derivatives **19**–**30** and **44**–**55** at the human and rat A_1_R (Tables 4 and 5).

The adenosine and NECA benzyloxycyclopentyl derivatives (Table 4) displayed RT comparable to adenosine and NECA on hA_1_R (∼21 min), while the phenoxycyclopentyl analogs generally had RT > 20 min (Table 5). As a general trend, the compounds are faster binders at rA_1_R regardless of linker length. The reason for this could be the divergent amino acid composition of the extracellular loops between hA_1_R and rA_1_R, which would favor different binding paths to the orthosteric site.^24^

Overall, the compounds displayed a very similar binding profile across the human and rat A_1_R, suggesting that further studies in rats would be highly relevant for the potential use of the compounds in humans. The adenosine and NECA-derived analog pairs also display very similar affinities for both human and rat A_1_R, suggesting that it is the R^2^ substituent on the phenoxy or benzyloxy ring that is key in determining the compound affinity for A_1_R. At the hA_1_R, the compounds with the highest affinity are **27**–**29**, **49**, **51**, **53**, and **54**. All of these have higher affinity at hA_1_R than adenosine and NECA alone and are all phenoxycyclopentyl derivatives. It is interesting to note that except for **49** and **51**, all of these compounds have a halogen (chloride or bromide) substituent, mostly in the *meta*-position of the aromatic phenoxy ring. **27**, **49**, **51**, **53**, and **54** all also have RT = 29–43 min at the human A_1_R, while the RT for the rest of the compounds is lower. By comparison, the benzyloxycyclopentyl derivatives generally display weaker binding and lower RT. At the rA_1_R, compounds with the highest affinity are **28**, **29**, **49**, **53**, and **54**. **27** and **52**, which have the bromide substituent on the aromatic ring, display reduced affinity at the rA_1_R as well as the hA_1_R when compared to **29** and **54**, respectively, which bear the chloride substituent at the same position. Considering the substitution position on the phenoxy ring, we observed the highest affinity with the chloride in the *meta*-position (**29**, **54**) followed by *ortho*- (**28**, **53**) and the *para*-position (**30**, **55**). Overall, halogen substituents as the R^2^ group on the aromatic ring seem to confer high affinity for the A_1_R, with chloride being preferential over bromide for binding at both the human and rat versions of the receptor.

**Figure 4:**
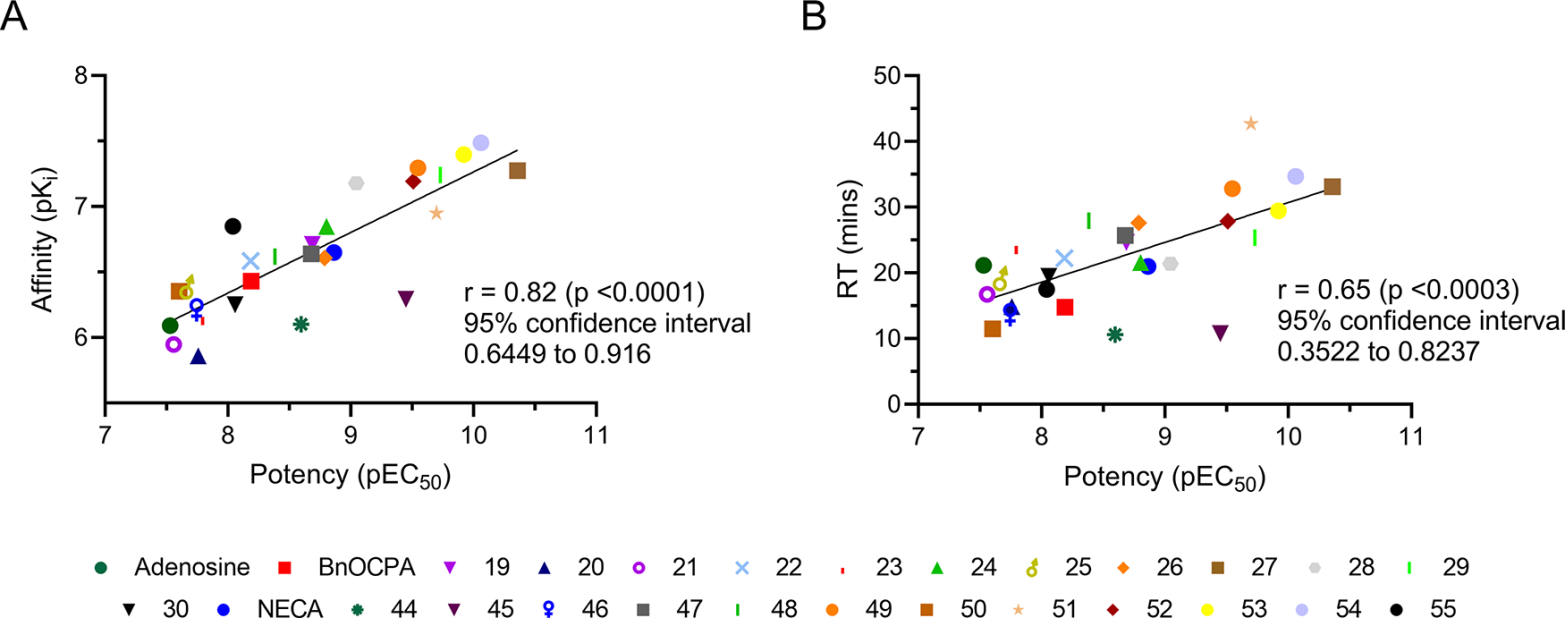
NECA and adenosine derivatives show correlation between potency and affinity or residence time at the hA_1_R. (A) Potency pEC_50_ values of individual compounds from cAMP inhibition experiments plotted against p*K*_i_ values from NanoBRET experiments at hA_1_R. (B) Potency pEC_50_ values of individual compounds from cAMP inhibition experiments plotted against RT values from NanoBRET experiments at hA_1_R.

Finally, we performed a comparison of the affinity data obtained from the NanoBRET binding assay with the potency for inhibition of cAMP accumulation for the hA_1_R, which showed a clear positive correlation (*r* = 0.82) with compounds **27**, **29**, **49**, and **51**–**54** identified as both the most potent and strongest binders (Figure 4A). A similar correlation was also observed between potency and compounds’ residence time (Figure 4B, *r* = 0.65). Overall, in this work, we have identified high-affinity, very selective potent hA_1_R agonists, namely, **27**, **49**, and **51**–**54**.

### Molecular Dynamics Simulations

To retrieve insight into the possible binding mode of the studied agonists and rationalize the selectivity displayed, *in silico* experiments were performed on the phenoxycyclopentyl adenosine derivative **27**, the most A_1_R-selective and potent agonist, and its benzyloxycyclopentyl congener **20**. A_1_R and A_2A_R structures solved in complex with adenosine or NECA (or homology models obtained from them, see the Experimental Section) present a closed conformation of the extracellular vestibule due to the lack of induced fit by *N*^6^ substituents, not present on adenosine or NECA. This structural feature does not allow molecular docking of compounds bearing bulky *N*^6^ groups to reproduce the binding mode of AR full agonists (Figure S2), which is characterized by the fundamental hydrogen bonds between the purine scaffold and the conserved Asn residue in position 6.55 and between the ribose ring and the Ser/Thr^7.42^ or His^7.43^. Therefore, molecular dynamics (MD) simulations of the four ARs subtypes were performed in the absence of any orthosteric agonists to sample receptors’ conformations more open at the extracellular loop 2 (ECL2) and ECL3 levels. Molecular docking results for **20** and **27** on the MD-derived AR structures were remarkably enhanced in the case of experimental structures A_1_R and A_2A_R, were slightly improved for the structural A_3_R model, and produced very little improvement for the A_2B_R model (Figure S3).

**Figure 5:**
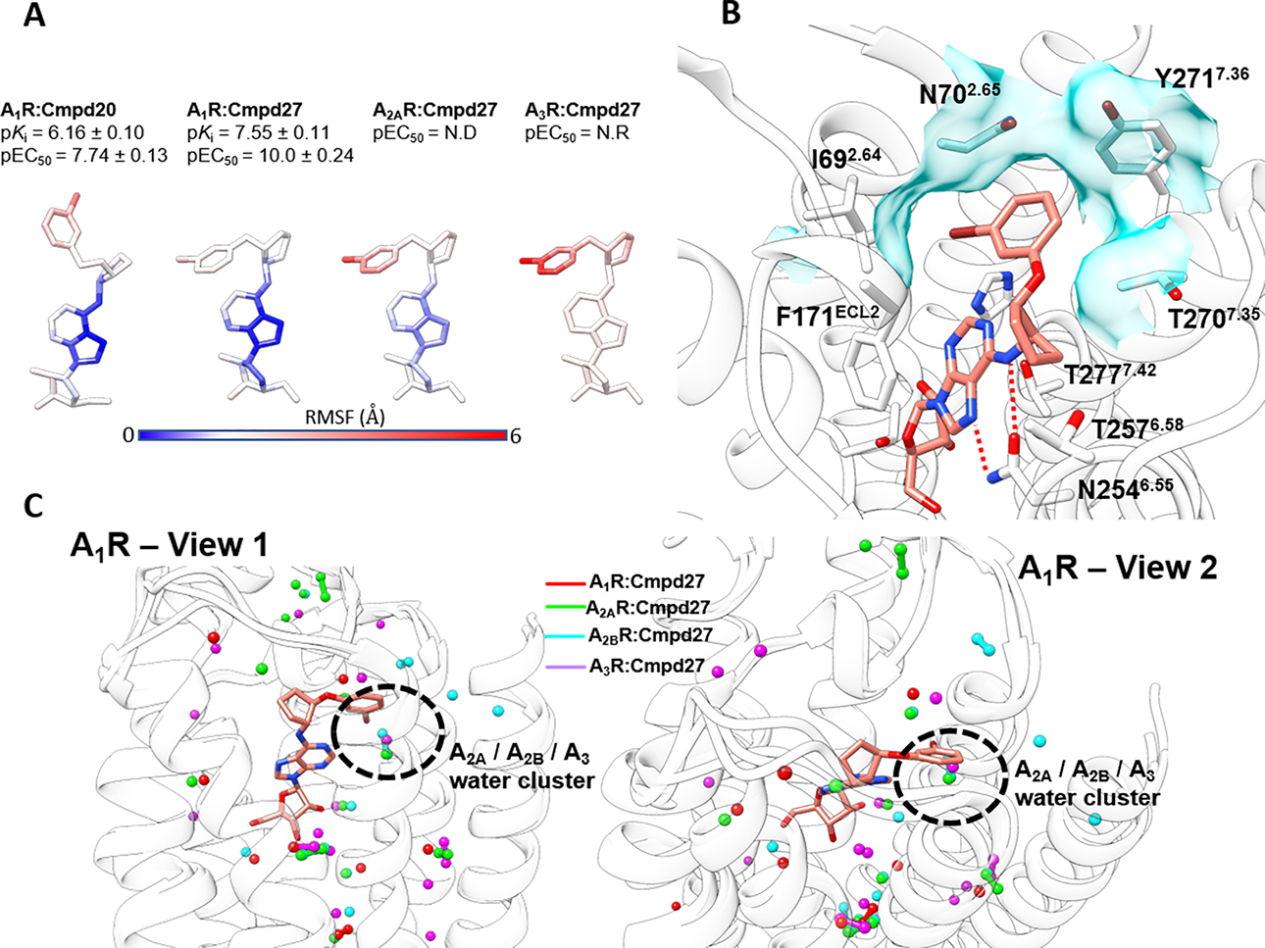
Molecular dynamics docking of **20** and **27**. (A) Atomic root mean square fluctuation (RMSF) of **20** within A_1_R and **27** within A_1_R, A_2A_R, and A_3_R plotted on the agonists’ structure. (B) Compound **27** (salmon stick representation) binding mode within A_1_R (white ribbon and sticks); the key hydrogen bonds with N254^6.55^ are shown as red dotted lines, while the hydrophobic subpocket is shown as a cyan transparent surface (coordinates provided in the Supporting Information (A1R_cmpd27_binding_mode.pdb)). (C) Two views (view 1, side; view 2, top) comparing the structural water molecules detected in A_1_R (red spheres), A_2A_R (green), A_2B_R (cyan), and A_3_R (purple). The position of the stable water cluster only present in A_2A_R, A_2B_R, and A_3_R is highlighted. Binding mode of **27** (salmon sticks) within A_1_R is superimposed for reference. Presented data are based on PDB structures 6D9H (A_1_R) and 5G53 (A_2A_R) and AlphaFold2 models of A_2B_R and A_3_R.

The best pose (in terms of similarity to adenosine) of **20** within A_1_R and the best pose of **27** within A_1_R or A_2A_R were further evaluated during 6 μs of MD simulations. For a complete comparison (Movie S1) of all four ARs subtypes, the best docking pose of **27** obtained on A_2A_R was superimposed on both A_2B_R and A_3_R and subjected to MD simulations. During the MD trajectories, **27** remained stably bound to A_1_R and A_2A_R but displayed less stable binding modes within A_3_R and, in particular, the A_2B_R orthosteric site (Movie S1, Figure S4A). Compound **20** within A_1_R (Movie S2) was steady throughout the simulations (Movie S2), as indicated by RMSD values in line with **27** (Figure S4A). In terms of flexibility, *N*^6^ substituents explored divergent conformations in the different systems (Figure 5A, Movies S1 and S2): the 3-bromophenyl group of **27** was highly flexible in A_3_R or A_2A_R and more stable in A_1_R, while the 3-bromobenzyl group of **20** displayed intermediate flexibility.

Compound **27** bound to A_1_R formed key hydrogen bonds with N254^6.55^ and hydrophobic contacts with F171^ECL2^ and oriented the 3-bromophenyl moiety in a hydrophobic subpocket formed by I69^2.64^, N70^2.65^, Y271^7.36^, and T270^7.35^ (Figure 5B, Movies S1 and S2). The bulkier analog **20** was not able to completely accommodate the 3-bromobenzyl group within this pocket and therefore displayed higher flexibility at the *N*^6^ level (Figure 5A, Movie S2). It is plausible that this contributes to the reduced A_1_R affinity and potency of **20** (p*K*_i_ = 6.16 ± 0.10, pEC_50_ = 7.74 ± 0.13) compared to **27** (p*K*_i_ = 7.55 ± 0.11, pEC_50_ = 10.0 ± 0.24). On the other hand, the interaction fingerprints of **20** (bound to A_1_R) and **27** are unique for each simulated complex (Figure S4B) and do not allow a straightforward rationalization of the selectivity displayed by the agonists. We therefore focused on the water molecule network present in the apo forms of the four AR subtypes (Figure 5C, Figure S4C–F). Our data suggest the presence of structural water molecules (A_2A_/A_2B_/A_3_ water cluster in Figure 5C) in the proximity of positions 2.64 and 2.65 of A_2_AR, A_2B_R, and A_3_R but not A_1_R stabilized by the short polar side chain of Ser^2.65^ (Asn^2.65^ in A_1_R, Figure S4B). It follows that the hydrophobic subpocket is putatively present only in A_1_R; hence, **27** cannot be completely stabilized by the other AR subtypes.

Taken together, computational results suggest that a one-atom linker between the *N*^6^-cyclopentane and the phenyl rings is optimal for stable binding to the hydrophobic pocket in A_1_R. The absence of this pocket and the presence of stable water molecules competing with the ligands in A_2A_R, A_2B_R, and A_3_R are probably responsible for the loss in affinity and potency of the tested agonists. The better complementarity with hA_1_R could explain why adenosine and NECA benzyloxycyclopentyl derivatives (Table 4) displayed RT comparable to adenosine and NECA at hA_1_R (∼21 min), while the phenoxycyclopentyl analogs generally had RT > 20 min (Table 5). Indeed, the shorter linker, as present in **27**, would stabilize the compounds and increase the energy required to produce dissociation.

### Validating the Predictions from the *In Silico* Experiments for A_1_R Bound to **20** and **27**

As described in Figure 5B, MD simulations suggested that the 3-bromophenyl moiety binds in a hydrophobic subpocket formed by I69^2.64^, N70^2.65^, Y271^7.36^, and T270^7.35^, while **20** was not able to completely accommodate the 3-bromobenzyl group within this pocket (Movie S2). To test these observations, we made use of previously described mutants (I69^2.64^A, N70^2.65^A, Y271^7.36^A) of the Nluc-A_1_R^24^ that enable comparison of ligand affinities with the wild-type receptor. We also included the mutant T257^6.58^A since we have previously shown that this residue is a good discriminator between different A_1_R agonists.^24^ We did not consider mutations of N254^6.55^ or F171^ELC2^ since these are known to prevent ligand binding to the A_1_R (including CA200645) and therefore cannot be studied.^24,37^ Furthermore, we did not consider mutating T270^7.35^ since, when we compared the sequences of the hA_1_R and the rA_1_R, we observed that, in the rA_1_R, the equivalent residue at position T270^7.35^ is an Ile. Comparison of the binding affinities (p*K*_i_) for **20** and **27** between the hA_1_R and the rA_1_R shows that **20** is equipotent between the two species while **27** has reduced affinity at the rA_1_R (p*K*_i_ (hA_1_R) = 7.55 ± 0.11; p*K*_i_ (rA_1_R) = 6.94 ± 0.08). Initially, we determined the *K*_d_ for CA200645 at each of the four A_1_R mutants (I69^2.64^A, N70^2.65^A, T257^6.58^A, and Y271^7.36^A) and found the values to show close agreement with those previously reported.^24^ We next performed a NanoBRET competition binding assay for the four mutants with BnOCPA (as a reference agonist), **20**, and **27** (Table 6, Figure S5).

**Table 6:** NanoBRET Competition-Binding Assay at Human Wild-Type and Mutant Nluc-A_1_R^a^

A_1_R	p*K*_i_^a^ (cmpd)					
	BnOCPA	*n*	**20**	*n*	**27**	*n*
WT	6.24 ± 0.04	3	6.38 ± 0.17	3	7.20 ± 0.16	3
I69^2.64^A	5.03 ± 0.02*	3	5.06 ± 0.04*	3	6.37 ± 0.10*	3
N70^2.65^A	6.01 ± 0.05	3	5.48 ± 0.15*	3	7.00 ± 0.24	3
T257^6.58^A	6.93 ± 0.10*	3	6.23 ± 0.02	3	8.09 ± 0.18*	3
Y271^7.36^A	5.40 ± 0.23*	3	5.19 ± 0.13*	3	6.33 ± 0.10*	3

a Compound affinity (p*K*_i_) determined through NanoBRET competition-binding assays with CA200645 in wild-type (WT) or mutant Nluc-A_1_R stably expressing HEK293 cells. The resulting concentration-dependent decrease in BRET ratio at 10 min was used to calculate p*K*_i_. Data are expressed as mean ± SEM obtained in *n* separate experiments. All individual experiments were conducted in duplicate. Statistical significance (*p < 0.05) compared to WT was determined by one-way ANOVA with Dunnett’s post-test and presented according to ref (31).

Consistent with MD simulation predictions, mutation of I69^2.64^ and Y271^7.36^ reduced the affinity of BnOCPA, **20**, and **27**. Interestingly, while the affinity at the A_1_R of BnOCPA and **27** was not affected by the mutation of N70^2.65^, **20** was significantly reduced. A closer analysis of the MD simulations suggested that the side chain of N70^2.65^ orients differently between **20** and **27**. For **20**, simulations predicted N70^2.65^ amidic side chain group interactions with the purine ring (Figure S6, Movie S2), which are lost in N70^2.65^A. These interactions can comprise water bridges involving the first solvation shell around the purine scaffold of the ligand.^38^ Conversely, for **27**, the N70^2.65^ amidic side chain group does not interact with the purine ring, but instead, it forms hydrophobic interactions through its methylene group with the bromobenzene ring (Figure 5B, Movie S2), implying that the mutation to Ala does not play a significant role in binding. Finally, as we have previously reported,^24^ the mutation of T257^6.58^A did provide a clear discriminator between the three different agonists. **27** together with BnOCPA both showed increases in binding affinity, while **20** displayed no significant change. In our previous studies, we observed that both CPA and BnOCPA displayed increased affinities at the T257^6.58^A mutant, while NECA showed reduced affinity and there was no change for adenosine. We attributed these changes to an increase in the lipophilicity of the protein environment underneath extracellular loop 3 (ECL3), which surrounds the cyclopentyl groups of the molecules. It is therefore apparent that the small molecule **27** favors a more hydrophobic environment within the binding pocket that is already suitable for **20**.

## Conclusions

Herein, we report the synthesis of novel *N*^6^-benzyloxycyclopentyl and *N*^6^-phenoxycyclopentyl derivatives of adenosine and NECA. These compounds were evaluated using the cAMP accumulation assay in CHO-K1 cells and the NanoBRET binding assay in HEK293 cells for potency, selectivity, and binding at ARs. Our pharmacology data show that compounds including halogen substituents, chloride in particular, on the aromatic phenoxy and benzyloxy rings confer high affinity for the human and rat A_1_R. These compounds also have high potency at the A_1_R, particularly ones with a *meta* substituent on the aromatic rings. Furthermore, we also show that NECA-based derivatives have generally higher A_1_R selectivity over the other AR subtypes. Molecular modeling studies suggest that the selectivity is driven by a short linker and the absence of stable water molecules within a subpocket of the hA_1_R orthosteric site. It is worth noting that compounds **45** and **51** show approximately 1500 times improved A_1_R selectivity over NECA itself. Overall, we have identified very selective and very potent A_1_R agonists with high affinity for the receptor, namely, phenoxycyclopentyl compounds **27**, **49**, and **51**–**54**, which have great therapeutic promise for overcoming insufficient receptor selectivity and potency that many current compounds face.

## Experimental Section

### General Chemistry

All reactions were performed in dry glassware under an inert argon atmosphere. Anhydrous solvents were purchased as dry over molecular sieves from Sigma-Aldrich (Merck). Solvents were evaporated under reduced pressure at approximately 45 °C using a Büchi Rotavapor or under high vacuum on a Schlenk line. Reagents were purchased from Sigma-Aldrich (Merck), Fluorochem, or Brunschwig and used without further purification. Reactions were monitored by thin layer chromatography (TLC) using aluminum sheets precoated with 0.2 mm silica (Macherey-Nagel ALUGRAM Xtra SII, G/UV_254_) or aluminum oxide (Macherey-Nagel POLYGRAM Alox N/UV_254_). Detection was under a UV light source (λ_max_ 254 nm or 366 nm) or through staining with a vanillin solution, with subsequent heating. Flash column chromatography was carried out on a Teledyne ISCO CombiFlash using prepacked RediSep Normal-phase Silica Flash Columns.

Proton nuclear magnetic resonance (^1^H NMR) and carbon nuclear magnetic resonance (^13^C NMR) spectra were recorded at room temperature using a Bruker Avance IIIHD-400, II-400, or IIIHD-300 spectrometer operating at 400 or 300 MHz, respectively, for ^1^H and at 101 and 75 MHz, respectively, for ^13^C. Chemical shifts (δ) are reported in parts per million (ppm) and are referenced to the residual solvent peak (DMSO-*d*_6_: δ_H_ = 2.50 ppm, δ_C_ = 39.52 ppm; CDCl_3_: δ_H_ = 7.26 ppm, δ_C_ = 77.16 ppm; methanol-*d*_4_: δ_H_ = 3.31 ppm, δ_C_ = 49.00 ppm) or Me_4_Si (δ_H_ = 0.00 ppm). The order of citation in parentheses is (1) multiplicity: s (singlet), d (doublet), t (triplet), q (quartet), quint (quintet), m (multiplet), *etc.*, and br (broad); (2) coupling constants (*J*) in hertz (Hz); and (3) number of equivalent nuclei (by integration). COSY, HSQC, and DEPT were routinely used to assign peaks in ^1^H and ^13^C NMR spectra. Addition of D_2_O was used to confirm the assignment of OH and NH peaks. High-resolution mass spectra (HRMS) were recorded on a Thermo-Scientific LTQ Orbitrap XL spectrometer consisting of a linear ion trap (LTQ) featuring an HCD collision cell, coupled to the Orbitrap mass analyzer, equipped with a nanoelectrospray ion source (NSI). HRMS spectra were determined by the Mass Spectrometry Group at the Department of Chemistry, Biochemistry, and Pharmaceutical Sciences, University of Bern, Switzerland (Prof. Dr. S. Schürch).

The purity of the compounds was determined with UPLC-MS on a Dionex UltiMate 3000 Rapid Separation LC system using a reversed-phase column (Acclaim RSLC, 120 C18, 3 × 50 mm, 2.2 μm, pore size 120 Å, flow rate 1.2 mL/min), which was coupled to a ESI-MS Micromass Platform (quadrupole mass spectrometer). The gradient used was 100% A to 100% D over 7 min, with A = MilliQ H_2_O + 0.1% TFA and D = 10% MilliQ H_2_O/90% HPLC-grade MeCN + 0.1% TFA. Compounds **24** and **29** were measured on a Thermo-Scientific UltiMate 3000 HPLC equipped with a reverse-phase column (Acclaim 120 C18, 4.6 × 150 mm, 5 μm, pore size 120 Å) and eluted with a gradient of MilliQ H_2_O/HPLC-grade MeCN + 0.1% TFA. Purity was determined by total absorbance at 254 nm. All tested compounds were >95% pure, except for **24** and **29** that were 95 and 94% pure, respectively (Tables S1 and S2).

### Established Adenosine Receptor Agonists

Adenosine and 5′-*N*-ethylcarboxamidoadenosine (NECA) were purchased from R & D Systems (Bristol, UK). Where possible, compounds were prepared as 10 mM stocks in DMSO.

### Chemical Synthesis

Intermediates **1**, **7**, and **31** and BnOCPA were synthesized as described previously.^13,39^

#### General Procedure A (*O*-Alkylation) for the Synthesis of Intermediates **2a**–**i**

Boc-protected (1*R*,2*R*)-2-aminocyclopentanol **1** and the appropriate benzyl bromide were dissolved in dry THF (50–100 mM). The reaction mixture was cooled to 0 °C, and NaH (60% dispersion in mineral oil) was added. After stirring at 0 °C, the reaction was quenched with MeOH (0.1 mL) and sat. aq. NH_4_Cl. The reaction mixture was extracted with EtOAc, and the organic phase was dried over Na_2_SO_4_ and concentrated under reduced pressure. The crude material was purified by flash column chromatography.

#### General Procedure B (Mitsunobu) for the Synthesis of Intermediates **5a,c,e,f,h**–**j**

Boc-protected (1*S,*2*R*)-2-aminocyclopentanol **4**, the appropriate phenol, and PPh_3_ were dissolved in dry THF (50–100 mM) and cooled to 0 °C. DIAD was added dropwise. Cooling was removed, and the reaction mixture was left to warm to room temperature and stirred overnight. Water was added, and the aqueous phase was extracted with EtOAc. The organic phase was dried over Na_2_SO_4_ and concentrated under reduced pressure. The crude material was purified by flash column chromatography.

#### General Procedure C (Boc Deprotection) for the Synthesis of Intermediates **3b**–**i** and **6a,c,e,f,h**–**j**

Boc-protected precursors **2b**–**i** and **5a**,**c**,**e**,**f**,**h**–**j** were dissolved in dioxane (85–830 mM), and HCl (4 M in dioxane) was added. After stirring the reaction mixture at room temperature, the solvent was removed under reduced pressure. The residual ammonium chloride salt was co-evaporated with CH_2_Cl_2_ and dried.

#### General Procedure D (S_N_Ar Reaction) for the Synthesis of Intermediates **8**–**14** and **32**–**43**

The appropriate 6-chloropurine (**7** or **31**) and the appropriate benzyloxy- or phenoxycyclopentyl amine intermediate (**3b**–**i** or **6a**,**c**,**e**,**f**,**h**–**j**) were dissolved in *i*-PrOH (11–36 mM). NaHCO_3_ was added, and the reaction mixture was heated at reflux (*ca*. 105 °C) overnight. After cooling, the solid was filtered off and washed with EtOH, and the solvents were removed under reduced pressure. The crude material was purified by flash column chromatography. In some examples, loss of acetate groups on the secondary alcohols was observed during the S_N_Ar reaction with **7**. In these cases, the crude material was subjected directly to the deprotection protocol (see general procedure G).

#### General Procedure E (Acetate Deprotection) for the Synthesis of Compounds **15**–**17**, **19**, **24**, **26**, and **30**

Acetate-protected intermediates **8**–**14** were dissolved in MeOH (9–22 mM), and K_2_CO_3_ was added. The reaction mixture was stirred at room temperature, filtered, and concentrated under reduced pressure. The crude material was purified by flash column chromatography.

#### General Procedure F (Acetonide Deprotection) for the Synthesis of Compounds **44**–**55**

Acetonide-protected intermediates **32**–**43** were dissolved in water (38–75 mM) and acetic acid and stirred at 80 °C overnight. The water and acetic acid were removed in vacuo, and the crude material was purified by flash column chromatography.

#### General Procedure G (S_N_Ar Reaction and Subsequent Acetate Deprotection) for the Synthesis of Compounds **20**–**23**, **25**, and **27**–**29**

6-Chloropurine **7** and the appropriate benzyloxy- or phenoxycyclopentyl amine intermediate (**3f**–**i** or **6c**,**f**,**h**,**i**) were dissolved in *i*-PrOH (23–46 mM). NaHCO_3_ was added, and the reaction mixture was heated at reflux (*ca*. 105 °C) overnight. After cooling, the solid was filtered off and washed with EtOH, and the solvents were removed under reduced pressure. The crude material was dissolved in MeOH (11–17 mM), and K_2_CO_3_ was added. The reaction mixture was stirred at room temperature, filtered, and concentrated under reduced pressure. The crude material was purified by flash column chromatography.

#### *tert*-Butyl ((1*R*,2*R*)-2-(Benzyloxy)cyclopentyl)carbamate (**2a**)

**2a** was synthesized according to general procedure A using **1** (0.497 mmol), benzyl bromide (0.497 mmol), and NaH (0.994 mmol). The reaction was run for 2 h. After purification with flash column chromatography (EtOAc/cHex, 15%), **2a** was obtained (83 mg, 0.285 mmol, 58%). ^1^H NMR: (300 MHz, DMSO-*d*_6_) δ 7.30 (m, 5H), 6.90 (d, *J* = 7.7, 1H), 4.53 (d, *J* = 12.2, 1H), 4.58–4.41 (m, 1H), 3.82–3.74 (m, 1H), 3.73–3.66 (m, 1H), 1.95–1.73 (m, 2H), 1.60 (m, 3H), 1.43–1.36 (m, 1H) 1.40 (s, 9H). ^13^C NMR: (75 MHz, DMSO-*d*6) δ 155.5, 139.4, 128.6, 127.8, 127.7, 85.0, 78.0, 70.3, 56.9, 30.6, 30.5, 28.8, 21.8. HRMS: (NSI+) *m/z* calcd for C_17_H_26_NO_3_ [M + H]^+^ 292.1902, found 292.1907.

#### *tert*-Butyl ((1*R*,2*R*)-2-((4-Isopropylbenzyl)oxy)cyclopentyl)carbamate (**2b**)

**2b** was synthesized according to general procedure A using **1** (1.242 mmol), 4-isopropylbenzyl bromide (1.242 mmol), and NaH (2.484 mmol). The reaction was run for 1 h 20 min. After purification with flash column chromatography (EtOAc/cHex, 15%), **2b** was obtained (135 mg, 0.406 mmol, 33%). ^1^H NMR: (300 MHz, DMSO-*d*_6_) δ 7.24–7.18 (m, 4H), 6.89 (d, *J* = 7.8, 1H), 4.5–4.38 (m, 2H), 3.80–3.72 (m, 1H), 3.72–3.65 (m, 1H), 2.87 (sept, *J* = 6.9, 1H), 1.93–1.73 (m, 2H), 1.67–1.49 (m, 3H), 1.42–1.32 (m, 1H) 1.40 (s, 9H), 1.19 (d, *J* = 6.9, 6H). HRMS: (NSI+) *m/z* calcd for C_20_H_32_NO_3_ [M + H]^+^ 334.2369, found 334.2377.

#### *tert*-Butyl ((1*R*,2*R*)-2-((4-(*tert*-Butyl)benzyl)oxy)cyclopentyl)carbamate (**2c**)

**2c** was synthesized according to general procedure A using **1** (1.242 mmol), 4-*tert*-butylbenzyl bromide (1.242 mmol), and NaH (2.484 mmol). The reaction was run for 1 h 30 min. After purification with flash column chromatography (EtOAc/cHex, 20%), **2c** was obtained as an oil (126 mg, 0.363 mmol, 30%). ^1^H NMR: (300 MHz, DMSO-*d*_6_) δ 7.35 (d, *J* = 8.2, 2H), 7.22 (d, *J* = 8.2, 2H), 6.89 (d, *J* = 7.8, 1H), 4.51–4.40 (m, 2H), 3.82–3.72 (m, 1H), 3.71–3.64 (m, 1H), 1.94–1.72 (m, 2H), 1.65–1.51 (m, 3H), 1.45–1.33 (m, 1H), 1.40 (s, 9H), 1.27 (s, 9H). ^13^C NMR: (101 MHz, DMSO-*d*_6_) δ 155.4, 150.1, 136.3, 127.8, 125.3, 84.8, 78.0, 70.1, 56.9, 34.7, 31.6, 30.7, 30.6, 28.8, 21.9. HRMS: (NSI+) *m/z* calcd for C_21_H_34_NO_3_ [M + H]^+^ 348.2533, found 348.2533.

#### *tert*-Butyl ((1*R*,2*R*)-2-((4-Cyanobenzyl)oxy)cyclopentyl)carbamate (**2d**)

**2d** was synthesized according to general procedure A using **1** (1 mmol), 4-(bromomethyl)benzonitrile (1 mmol), and NaH (2 mmol). The reaction was run for 6 h 30 min. After purification with flash column chromatography (EtOAc/cHex, 20%), **2d** was obtained as an oil (287 mg, 0.91 mmol, 91%). ^1^H NMR: (300 MHz, methanol-*d*_4_) δ 7.69 (d, *J* = 8.3, 2H), 7.58–7.47 (m, 2H), 4.71–4.56 (m, 2H), 3.96–3.83 (m, 1H), 3.83–3.71 (m, 1H), 2.14–1.61 (m, 5H), 1.44 (s, 10H). ^13^C NMR: (101 MHz, methanol-*d*_4_) δ 151.2, 146.3, 133.2, 129.0, 118.9, 87.0, 71.0, 58.2, 31.3, 31.3, 28.8, 22.5. HRMS: (NSI+) *m/z* calcd for C_18_H_25_N_2_O_3_ [M + H]^+^ 317.1860, found 317.1858.

#### *tert*-Butyl ((1*R*,2*R*)-2-((3-Methoxybenzyl)oxy)cyclopentyl)carbamate (**2e**)

**2e** was synthesized according to general procedure A using **1** (0.497 mmol), 3-methoxybenzyl bromide (0.497 mmol), and NaH (0.994 mmol). The reaction was run for 3 h. After purification with flash column chromatography (EtOAc/cHex, 20%), **2e** was obtained (76 mg, 0.235 mmol, 47%). ^1^H NMR: (300 MHz, DMSO-*d*_6_) δ 7.24 (t, *J* = 8.0, 1H), 6.93–6.80 (m, 3H), 4.53–4.42 (m, 2H), 3.82–3.72 (m, 1H), 3.75 (s, 3H), 3.72–3.65 (m, 1H), 1.95–1.73 (m, 2H), 1.69–1.49 (m, 3H), 1.44–1.32 (m, 1H),1.39 (s, 9H). ^13^C NMR: (75 MHz, DMSO-*d*_6_) δ 159.7, 155.5, 141.0, 129.7, 119.9, 113.2, 84.8, 78.0, 70.1, 56.9, 55.4, 30.6, 30.5, 28.7, 21.8. HRMS: (NSI+) *m/z* calcd for C_18_H_28_NO_4_ [M + H]^+^ 322.2021, found 322.2013.

#### *tert*-Butyl ((1*R*,2*R*)-2-((3-Bromobenzyl)oxy)cyclopentyl)carbamate (**2f**)

**2f** was synthesized according to general procedure A using **1** (1.242 mmol), 3-bromobenzyl bromide (1.242 mmol), and NaH (2.484 mmol). The reaction was run for 3 h. After purification with flash column chromatography (EtOAc/cHex, 15–20%), **2f** was obtained (268 mg, 0.725 mmol, 58%). ^1^H NMR: (300 MHz, DMSO-*d*_6_) δ 7.51–7.46 (m, 1H), 7.38–7.31 (m, 1H), 7.27–7.13 (m, 2H), 6.91 (d, *J* = 7.7, 1H), 4.57–4.45 (m, 2H), 3.81–3.72 (m, 1H), 3.72–3.64 (m, 1H), 1.95–1.75 (m, 2H), 1.68–1.49 (m, 3H), 1.46–1.33 (m, 1H), 1.40 (s, 9H). ^13^C NMR: (75 MHz, methanol-*d*_4_) δ 156.5, 141.5, 130.1, 130.1, 129.7, 125.9, 121.9, 85.2, 78.6, 69.7, 56.7, 30.0, 27.5, 26.6, 21.1. HRMS: (NSI+) *m/z* calcd for C_17_H_24_BrNNaO_3_ [M + Na]^+^ 392.0830, found 392.0832.

#### *tert*-Butyl ((1*R*,2*R*)-2-((4-Bromobenzyl)oxy)cyclopentyl)carbamate (**2g**)

**2g** was synthesized according to general procedure A using **1** (0.8 mmol), 4-bromobenzyl bromide (0.8 mmol), and NaH (1.6 mmol). The reaction was run for 3 h. After purification with flash column chromatography (EtOAc/cHex, 20%), **2g** was obtained (198 mg, 0.535 mmol, 67%). ^1^H NMR (300 MHz, DMSO-*d*_6_) δ 7.52 (d, *J* = 8.4, 2H), 7.27 (d, *J* = 8.4, 2H), 4.48 (m, 2H), 3.82–3.78 (m, 1H), 3.70–3.59 (m, 1H), 1.94–1.74 (m, 2H), 1.59 (m, 3H), 1.44–1.34 (m, 1H), 1.39 (s, 9H). ^13^C NMR: (101 MHz, methanol-*d*_4_) δ 156.5, 144.9, 131.8, 127.6, 118.4, 110.6, 85.6, 78.6, 69.6, 56.8, 29.9, 27.4, 21.1.

#### *tert*-Butyl ((1*R*,2*R*)-2-((2-Chlorobenzyl)oxy)cyclopentyl)carbamate (**2h**)

**2h** was synthesized according to general procedure A using **1** (0.994 mmol), 2-chlorobenzyl bromide (0.994 mmol), and NaH (1.987 mmol). The reaction was run for 1.5 h at 0 °C and then for another 1.5 h at room temperature. After purification with flash column chromatography (EtOAc/cHex, 20%), **2h** was obtained (178 mg, 0.504 mmol, 51%). ^1^H NMR: (300 MHz, DMSO-*d*_6_) δ 7.53–7.40 (m, 2H), 7.37–7.29 (m, 2H), 6.94 (d, *J* = 7.7, 1H), 4.65–4.50 (m, 2H), 3.85–3.70 (m 2H), 1.99–1.78 (m, 2H), 1.68–1.53 (m, *J* = 9.3, 3H), 1.46–1.35 (m, 1H), 1.40 (s, 9H). ^13^C NMR: (75 MHz, methanol-*d*_4_) δ 156.5, 136.3, 132.6, 129.05, 128.8, 128.5, 126.5, 85.6, 78.6, 67.9, 56.8, 30.0, 29.8, 27.4, 21.0. HRMS: (NSI+) *m/z* calcd for C_17_H_24_ClNNaO_3_ [M + Na]^+^ 348.1336, found 348.1337.

#### *tert*-Butyl ((1*R*,2*R*)-2-((3-Chlorobenzyl)oxy)cyclopentyl)carbamate (**2i**)

**2i** was synthesized according to general procedure A using **1** (0.994 mmol), 3-chlorobenzyl bromide (0.994 mmol), and NaH (1.987 mmol). The reaction was run for 3 h. After purification with flash column chromatography (EtOAc/cHex, 20%), **2i** was obtained (224 mg, 0.688 mmol, 70%). ^1^H NMR: (300 MHz, DMSO-*d*_6_) δ 7.42–7.23 (m, 4H), 6.91 (d, *J* = 7.7, 1H), 4.58–4.45 (m, 2H), 3.81–3.73 (m, 1H), 3.73–3.65 (m, 1H), 1.96–1.71 (m, 2H), 1.68–1.47 (m, 3H), 1.46–1.33 (m, 1H), 1.40 (s, 9H). ^13^C NMR: (75 MHz, methanol-*d*_4_) δ 156.5, 141.3, 133.8, 129.4, 127.2, 127.1, 125.5, 85.2, 78.6, 69.7, 56.7, 30.0, 29.9, 27.4, 21.1. HRMS: (NSI+) *m/z* calcd for C_17_H_24_ClNNaO_3_ [M + Na]^+^ 348.1338, found 348.1337.

#### (1*R*,2*R*)-2-((4-Isopropylbenzyl)oxy)cyclopentan-1-aminium Chloride (**3b**)

**3b** was synthesized according to general procedure C using **2b** (0.405 mmol) and HCl (2.03 mmol). The reaction was run for 3 h. **3b** was obtained as a solid (100 mg, 0.43 mmol, quant.). ^1^H NMR: (300 MHz, DMSO-*d*_6_) δ 8.05 (br s, 3H), 7.32–7.19 (m, 4H), 4.54–4.40 (m, 2H), 3.95–3.87 (m, 1H), 3.42 (br s, 1H), 2.88 (sept, *J* = 6.9, 1H), 2.09–1.90 (m, 2H), 1.76–1.45 (m, 4H), 1.20 (d, *J* = 6.9, 6H). HRMS: (NSI+) *m/z* calcd for C_15_H_24_NO [M]^+^ 234.1849, found 234.1852.

#### (1*R*,2*R*)-2-((4-(*tert*-Butyl)benzyl)oxy)cyclopentan-1-aminium Chloride (**3c**)

**3c** was synthesized according to general procedure C using **2c** (0.329 mmol) and HCl (1.649 mmol). The reaction was run for 5 h. **3c** was obtained as a colorless solid (93 mg, 0.328 mmol, 99%). ^1^H NMR: (300 MHz, DMSO-*d*_6_) δ 8.02 (br s, 3H), 7.38 (d, *J* = 8.4, 2H), 7.28 (d, *J* = 8.4, 2H), 4.54–4.39 (m, 2H), 3.93–3.86 (m, 1H), 3.47–3.36 (m, 1H), 2.11–1.92 (m, 2H), 1.75–1.48 (m, 4H), 1.28 (s, 9H). ^13^C NMR: (75 MHz, DMSO-*d*_6_) δ 150.4, 135.7, 128.0, 125.4, 82.8, 70.7, 56.3, 34.7, 31.6, 30.3, 28.9, 21.6. HRMS: (NSI+) *m/z* calcd for C_16_H_26_NO [M]^+^ 248.2005, found 248.2009.

#### (1*R*,2*R*)-2-((4-Cyanobenzyl)oxy)cyclopentan-1-aminium Chloride (**3d**)

**3d** was synthesized according to general procedure C using **2d** (0.825 mmol) and HCl (8 mmol). After stirring for 1 h, a colorless precipitate formed. Drops of water were added until all the solid dissolved. The reaction mixture was then stirred for an additional 3 h. **3d** was obtained as a colorless solid (209 mg, 0.825 mmol, quant.). ^1^H NMR: (300 MHz, DMSO-*d*_6_) δ 8.02 (s, 3H), 7.84 (d, *J* = 8.2, 2H), 7.58 (d, *J* = 8.3, 2H), 4.68–4.57 (m, 2H), 3.99–3.93 (m, 1H), 3.44 (s, 1H), 2.11–1.92 (m, 2H), 1.76–1.49 (m, 4H). HRMS: (NSI+) *m/z* calcd for C_13_H_17_N_2_O [M]^+^ 217.1335, found 217.1333.

#### (1*R*,2*R*)-2-((3-Methoxybenzyl)oxy)cyclopentan-1-aminium Chloride (**3e**)

**3e** was synthesized according to general procedure C using **2e** (0.69 mmol) and HCl (3.49 mmol). The reaction was run for 4 h. **3e** was obtained as a colorless solid (88 mg, 0.341 mmol, 50%). ^1^H NMR: (300 MHz, DMSO-*d*_6_) δ 8.20 (br s, 3H), 7.27 (t, *J* = 8.1, 1H), 6.98–6.82 (m, 3H), 4.57–4.42 (m, 2H), 3.98–3.88 (m, 1H), 3.76 (s, 3H), 3.47–3.37 (m, 1H), 1.76–1.49 (m, 2H), 1.76–1.49 (m, 4H). ^13^C NMR: (75 MHz, DMSO-*d_6_*) δ 159.7, 140.4, 129.7, 117.5, 113.5, 113.4, 82.9, 70.8, 56.3, 55.5, 30.2, 28.9, 21.6. HRMS: (NSI+) *m/z* calcd for C_13_H_20_NO_2_ [M]^+^ 222.1491, found 222.1489.

#### (1*R*,2*R*)-2-((3-Bromobenzyl)oxy)cyclopentan-1-aminium Chloride (**3f**)

**3f** was synthesized according to general procedure C using **2f** (0.716 mmol) and HCl (14.3 mmol). The reaction was run for 1 h, after which a few drops of MeOH were added to dissolve precipitate. The reaction mixture was then stirred for another 1.5 h. **3f** was obtained as a solid (224 mg, 0.732 mmol, quant.). ^1^H NMR: (300 MHz, methanol-*d*_4_) δ 7.48 (t, *J* = 1.9, 1H), 7.37–7.32 (m, 1H), 7.27–7.13 (m, 2H), 4.50 (d, *J* = 12.0, 1H), 4.42 (d, *J* = 12.0, 1H), 3.92–3.80 (m, 1H), 3.45–3.34 (m, 1H), 2.16–1.91 (m, 2H), 1.82–1.44 (m, 4H). ^13^C NMR: (75 MHz, methanol-*d*_4_) δ 140.7, 130.4, 130.3, 129.9, 126.1, 122.0, 82.8, 70.3, 56.6, 29.1, 27.6, 20.3. HRMS: (NSI+) *m/z* calcd for C_12_H_17_BrNO [M]^+^ 270.0489, found 270.0488.

#### (1*R*,2*R*)-2-((4-Bromobenzyl)oxy)cyclopentan-1-aminium Chloride (**3g**)

**3g** was synthesized according to general procedure C using **2g** (0.481 mmol) and HCl (9.61 mmol). The reaction was run for 30 min, after which a few drops of MeOH were added to dissolve precipitate. The reaction mixture was then stirred for another 30 min. **3g** was obtained as a solid (114 mg, 0.373 mmol, 77%). ^1^H NMR (300 MHz, methanol-*d*_4_) δ 7.40 (d, *J* = 8.4, 2H), 7.21 (d, *J* = 8.1, 2H), 4.51–4.37 (m, 2H), 3.89–3.80 (m, 1H), 3.44–3.34 (m, 1H), 2.17–1.90 (m, 2H), 1.79–1.41 (m, 4H). ^13^C NMR: (75 MHz, methanol-*d*_4_) δ 137.3, 131.1, 129.4, 121.1, 82.7, 70.4, 56.6, 29.1, 27.6, 20.3. HRMS: (NSI+) *m*/*z* calcd for C_12_H_17_BrNO [M]^+^ 270.0493, found 270.0488.

#### (1*R*,2*R*)-2-((2-Chlorobenzyl)oxy)cyclopentan-1-aminium Chloride (**3h**)

**3h** was synthesized according to general procedure C using **2h** (0.467 mmol) and HCl (9.33 mmol). The reaction was run for 45 min, after which a few drops of MeOH were added to dissolve precipitate. The reaction mixture was then stirred for another 1.5 h. **3h** was obtained as a solid (121 mg, 0.462 mmol, 99%). ^1^H NMR: (300 MHz, methanol-*d*_4_) δ 7.50–7.41 (m, 1H), 7.34–7.12 (m, 3H), 4.63–4.49 (m, 2H), 4.00–3.89 (m, 1H), 3.49–3.35 (m, 1H), 2.20–1.94 (m, 2H), 1.85–1.48 (m, 4H). ^13^C NMR: (75 MHz, methanol-*d*_4_) δ 132.8, 129.5, 128.93, 128.92, 126.7, 83.2, 68.5, 56.6, 29.2, 27.7, 20.4. HRMS: (NSI+) *m*/*z* calcd for C_12_H_17_ClNO [M]^+^ 226.0995, found 226.0993.

#### (1*R*,2*R*)-2-((3-Chlorobenzyl)oxy)cyclopentan-1-aminium Chloride (**3i**)

**3i** was synthesized according to general procedure C using **2i** (0.687 mmol) and HCl (13.75 mmol). The reaction was run for 45 min, after which a few drops of MeOH were added to dissolve the precipitate. The reaction mixture was then stirred for another 1.5 h. **3i** was obtained as a solid (176 mg, 0.671 mmol, 97%). ^1^H NMR: (300 MHz, DMSO-*d*_6_) δ 8.26 (br s, 3H), 7.48–7.29 (m, 4H), 4.60–4.46 (m, 2H), 4.00–3.92 (m, 1H), 3.49–3.37 (m, 1H), 2.09–1.93 (m, 2H), 1.80–1.50 (m, 4H). ^13^C NMR: (75 MHz, methanol-*d*_4_) δ 140.5, 133.9, 129.6, 127.4, 127.3, 125.7, 82.8, 66.8, 56.6, 29.1, 27.6, 20.3. HRMS: (NSI+) *m*/*z* calcd for C_12_H_17_ClNO [M]^+^ 226.1002, found 226.0993.

#### *tert*-Butyl ((1*R*,2*R*)-2-Phenoxycyclopentyl)carbamate (**5a**)

**5a** was synthesized according to general procedure B using **4** (0.994 mmol), phenol (1.242 mmol), PPh_3_ (1.242 mmol), and DIAD (1.242 mmol). The reaction was run for 21 h. After purification with flash column chromatography (EtOAc/cHex, 20%), **5a** was obtained (165 mg, 0.594 mmol, 60%). ^1^H NMR: (300 MHz, DMSO-*d*_6_) δ 7.33–7.23 (m, 2H), 7.04 (d, *J* = 7.3, 1H), 6.99–6.86 (m, 3H), 4.57–4.48 (m, 1H), 3.89–3.79 (m, 1H), 2.09–1.87 (m, 2H), 1.79–1.42 (m, 4H), 1.39 (s, 9H). ^13^C NMR: (101 MHz, DMSO-*d*_6_) δ 157.6, 155.2, 129.4, 120.4, 115.3, 82.2, 77.7, 56.54, 29.8, 29.6, 28.2, 21.2. HRMS: (NSI+) *m/z* calcd for C_16_H_24_NO_3_ [M + H]^+^ 278.1756, found 278.1747.

#### *tert*-Butyl ((1*R*,2*R*)-2-(4-(*tert*-Butyl)phenoxy)cyclopentyl)carbamate (**5c**)

**5c** was synthesized according to general procedure B using **4** (0.994 mmol), 4-*tert*-butylphenol (1.242 mmol), PPh_3_ (1.242 mmol), and DIAD (1.242 mmol). The reaction was run for 19 h. After purification with flash column chromatography (EtOAc/cHex, 20%), **5c** was obtained (189 mg, 0.567 mmol, 57%). ^1^H NMR: (300 MHz, DMSO-d_*6*_) δ 7.31–7.24 (m, 2H), 7.02 (d, *J* = 7.3, 1H), 6.86 (d, *J* = 8.5, 2H), 4.52–4.45 (m, 1H), 3.88–3.75 (m, 1H), 2.07–1.89 (m, 2H), 1.80–1.43 (m, 4H), 1.39 (s, 9H), 1.25 (s, 9H). ^13^C NMR: (75 MHz, methanol-d_*4*_) δ 156.5, 155.7, 143.0, 125.7, 114.8, 82.6, 78.7, 56.9, 33.5, 30.6, 29.8, 29.7, 27.4, 21.0. HRMS: (NSI+) *m/z* calcd for C_20_H_32_NO_3_ [M + H]^+^ 334.2364, found 334.2377.

#### *tert*-Butyl ((1*R*,2*R*)-2-(3-Methoxyphenoxy)cyclopentyl)carbamate (**5e**)

**5e** was synthesized according to general procedure B using **4** (1.987 mmol), 3-methoxyphenol (2.484 mmol), PPh_3_ (2.484 mmol), and DIAD (2.484 mmol). The reaction was run for 18 h. After purification with flash column chromatography (EtOAc/cHex, 20%), **5e** was obtained (381 mg, 1.236 mmol, 62%). ^1^H NMR: (300 MHz, DMSO-*d*_6_) δ 7.15 (t, *J* = 8.2, 1H), 7.05 (d, *J* = 7.3, 1H), 6.58–6.44 (m, 3H), 4.54–4.47 (m, 1H), 3.88–3.78 (m, 1H), 3.73 (s, 3H), 2.05–1.87 (m, 2H), 1.77–1.54 (m, 3H), 1.53–1.42 (m, 1H), 1.39 (s, 9H). ^13^C NMR: (101 MHz, methanol-*d*_4_) δ 161.0, 159.2, 156.5, 129.4, 107.6, 106.1, 101.4, 82.7, 78.7, 56.9, 54.3, 29.8, 27.4, 21.1. HRMS: (NSI+) *m/z* calcd for C_17_H_25_NNaO_4_ [M + Na]^+^ 330.1683, found 330.1687.

#### *tert*-Butyl ((1*R*,2*R*)-2-(3-Bromophenoxy)cyclopentyl)carbamate (**5f**)

**5f** was synthesized according to general procedure B using **4** (0.497 mmol), 3-bromophenol (0.621 mmol), PPh_3_ (0.621 mmol), and DIAD (0.621 mmol). The reaction was run for 18 h. After purification with flash column chromatography (EtOAc/cHex, 20%), **5f** was obtained (90 mg, 0.253 mmol, 51%). ^1^H NMR: (300 MHz, DMSO-*d*_6_) δ 7.26–6.91 (m, 4H), 4.58–4.51 (m, 1H), 3.88–3.76 (m, 1H), 2.09–1.86 (m, 2H), 1.80–1.42 (m, 4H), 1.39 (s, 9H). ^13^C NMR: (75 MHz, methanol-*d*_4_) δ 158.9, 156.4, 130.4, 123.33, 122.3, 118.6, 114.2, 83.2, 78.7, 56.9, 29.8, 29.7, 21.1, 20.4. HRMS: (NSI+) *m/z* calcd for C_16_H_22_BrNNaO_3_ [M + Na]^+^ 378.0686, found 378.0675.

#### *tert*-Butyl ((1*R*,2*R*)-2-(2-Chlorophenoxy)cyclopentyl)carbamate (**5h**)

**5h** was synthesized according to general procedure B using **4** (0.994 mmol), 2-chlorophenol (1.242 mmol), PPh_3_ (1.242 mmol), and DIAD (1.242 mmol). The reaction was run for 19 h. After purification with flash column chromatography (EtOAc/cHex, 10%), **5h** was obtained (187 mg, 0.597 mmol, 60%). ^1^H NMR: (300 MHz, DMSO-*d*_6_) δ 7.44–7.38 (m, 1H), 7.32–7.20 (m, 2H), 7.06 (d, *J* = 7.2, 1H), 6.98–6.90 (m, 1H), 4.64–4.57 (m, 1H), 3.92–3.81 (br s, 1H), 2.08–1.91 (m, 2H), 1.82–1.44 (m, 4H), 1.39 (s, 9H). ^13^C NMR: (75 MHz, methanol-*d*_4_) δ 156.4, 153.5, 129.8, 127.5, 123.2, 121.2, 115.3, 83.9, 78.7, 56.8, 29.9, 29.6, 27.4, 21.1. HRMS: (NSI+) *m/z* calcd for C_16_H_23_ClNO_3_ [M + H]^+^ 312.1359, found 312.1361.

#### *tert*-Butyl ((1*R*,2*R*)-2-(3-Chlorophenoxy)cyclopentyl)carbamate (**5i**)

**5i** was synthesized according to general procedure B using **4** (0.994 mmol), 3-chlorophenol (1.242 mmol), PPh_3_ (1.242 mmol), and DIAD (1.242 mmol). The reaction was run for 18 h. After purification with flash column chromatography (EtOAc/cHex, 10%), **5i** was obtained (170 mg, 0.544 mmol, 55%). ^1^H NMR: (300 MHz, DMSO-*d*_6_) δ 7.29 (t, *J* = 8.1, 1H), 7.11–6.90 (m, 4H), 4.59–4.52 (m, 1H), 3.89–3.77 (m, 1H), 2.12–1.86 (m, 2H), 1.81–1.42 (m, 4H), 1.39 (s, 9H). ^13^C NMR: (75 MHz, methanol-*d*_4_) δ 158.9, 156.5, 134.4, 130.1, 120.3, 115.59, 113.8, 83.2, 78.7, 56.9, 29.74, 29.65, 27.4, 21.1. HRMS: (NSI+) *m/z* calcd for C_16_H_23_ClNO_3_ [M + H]^+^ 312.1354, found 312.1361.

#### *tert*-Butyl ((1*R*,2*R*)-2-(4-Chlorophenoxy)cyclopentyl)carbamate (**5j**)

**5j** was synthesized according to general procedure B using **4** (0.994 mmol), 4-chlorophenol (1.242 mmol), PPh_3_ (1.242 mmol), and DIAD (1.242 mmol). The reaction was run for 20 h. After purification with flash column chromatography (EtOAc/cHex, 10%), **5j** was obtained (191 mg, 0.611 mmol, 62%). ^1^H NMR: (300 MHz, DMSO-*d*_6_) δ 7.30 (d, *J* = 9.0, 2H), 7.05 (d, *J* = 7.3, 1H), 6.98 (d, *J* = 8.9, 2H), 4.56–4.47 (m, 1H), 3.87–3.77 (m, 1H), 2.08–1.86 (m, 2H), 1.78–1.43 (m, 4H), 1.38 (s, 9H). ^13^C NMR: (75 MHz, DMSO-*d*_6_) δ 157.0, 155.6, 129.7, 124.6, 117.6, 83.2, 78.3, 57.0, 30.2, 30.0, 28.7, 21.6. HRMS: (NSI+) *m/z* calcd for C_16_H_22_ClNNaO_3_ [M + Na]^+^ 334.1182, found 334.1180.

#### (1*R*,2*R*)-2-Phenoxycyclopentan-1-aminium chloride (**6a**)

**6a** was synthesized according to general procedure C using **5a** (0.804 mmol) and HCl (16.09 mmol). The reaction was run for 18 h. **6a** was obtained as a solid (173 mg, 0.809 mmol, quant.). ^1^H NMR: (300 MHz, DMSO-*d_6_*) δ 8.20 (s, 3H), 7.39–7.25 (m, 2H), 7.04–6.91 (m, 3H), 4.77–4.68 (m, 1H), 3.64–3.55 (m, 1H), 2.25–2.02 (m, 2H), 1.83–1.56 (m, 4H). ^13^C NMR: (75 MHz, methanol-*d*_4_) δ 157.3, 129.3, 121.1, 115.3, 80.6, 57.0, 29.4, 28.0, 20.7. HRMS: (NSI+) *m/z* calcd for C_11_H_16_NO [M]^+^ 178.1228, found 178.1226.

#### (1*R*,2*R*)-2-(4-(*tert*-Butyl)phenoxy)cyclopentan-1-aminium Chloride (**6c**)

**6c** was synthesized according to general procedure C using **5c** (0.507 mmol) and HCl (10.14 mmol). The reaction was run for 20 h, after which a few drops of MeOH were added to dissolve the precipitate. Stirring was continued for another 21 h, and more 4 M HCl in dioxane (1 mL) was added to bring the reaction to completion. **6c** was obtained as a solid (117 mg, 0.432 mmol, 85%). ^1^H NMR: (300 MHz, DMSO-*d*_6_) δ 8.11 (br s, 3H), 7.37–7.28 (m, 2H), 6.92–6.85 (m, 2H), 4.69–4.63 (m, 1H), 3.65–3.53 (m, 1H), 2.23–2.02 (m, 2H), 1.84–1.54 (m, 4H), 1.26 (s, 9H). ^13^C NMR: (75 MHz, methanol-*d*_4_) δ 155.0, 144.0, 126.0, 114.9, 80.7, 57.0, 33.6, 30.6, 29.5, 28.0, 20.7. HRMS: (NSI+) *m/z* calcd for C_15_H_24_NO [M]^+^ 234.1850, found 234.1852.

#### (1*R*,2*R*)-2-(3-Methoxyphenoxy)cyclopentan-1-aminium chloride (**6e**)

**6e** was synthesized according to general procedure C using **5e** (1.236 mmol) and HCl (6.18 mmol). The reaction was run for 19 h, after which more 4 M HCl in dioxane (2 mL) was added to bring the reaction to completion. **6e** was obtained as a solid (322 mg, 1.23 mmol, quant.). ^1^H NMR: (300 MHz, DMSO-*d*_6_) δ 8.33 (br s, 3H), 7.21 (t, *J* = 8.2, 1H), 6.61–6.47 (m, 3H), 4.80–4.71 (m, 1H), 3.74 (s, 3H), 3.61–3.51 (m, 1H), 2.25–2.04 (m, 2H), 1.86–1.59 (m, 4H). ^13^C NMR: (101 MHz, methanol-*d*_4_) δ 161.1, 158.5, 129.8, 107.4, 106.6, 102.0, 80.8, 57.0, 54.4, 29.5, 27.9, 20.7. HRMS: (NSI+) *m/z* calcd for C_12_H_18_NO_2_ [M]^+^ 208.1329, found 208.1332.

#### (1*R*,2*R*)-2-(3-Bromophenoxy)cyclopentan-1-aminium chloride (**6f**)

**6f** was synthesized according to general procedure C using **5f** (0.253 mmol) and HCl (5.05 mmol). The reaction was run for 1 h, after which a few drops of MeOH were added to dissolve precipitate. Stirring was then continued for another 19 h. **6f** was obtained as a solid (74 mg, 0.252 mmol, quant.). ^1^H NMR: (300 MHz, DMSO-*d*_6_) δ 8.20 (br s, 3H), 7.29 (t, *J* = 8.3, 1H), 7.20–7.15 (m, 2H), 7.03–6.96 (m, 1H), 4.77–4.71 (m,1H), 3.63–3.53 (m, 1H), 2.24–2.03 (m, 2H), 1.84–1.56 (m, 4H). ^13^C NMR: (101 MHz, methanol-*d*_4_) δ 158.2, 130.7, 124.2, 122.4, 118.7, 114.2, 81.1, 56.9, 29.3, 27.9, 20.7. HRMS: (NSI+) *m/z* calcd for C_11_H_15_BrNO [M]^+^ 256.0330, found 256.0332.

#### (1*R*,2*R*)-2-(2-Chlorophenoxy)cyclopentan-1-aminium chloride (**6h**)

**6h** was synthesized according to general procedure C using **5 h** (0.6 mmol) and HCl (12 mmol). The reaction was run for 69 h. **6h** was obtained as a solid (149 mg, 0.6 mmol, quant.). ^1^H NMR: (300 MHz, DMSO-*d*_6_) δ 8.30 (br s, 3H), 7.46 (dd, *J* = 7.9, 1.6, 1H), 7.36–7.30 (m, 1H), 7.23 (dd, *J* = 8.4, 1.5, 1H), 7.05–6.97 (m, 1H), 4.90–4.83 (m, 1H), 3.68–3.59 (m, 1H), 2.24–2.09 (m, 2H), 1.86–1.62 (m, 4H). ^13^C NMR: (75 MHz, methanol-*d*_4_) δ 152.7, 130.1, 127.8, 123.4, 122.2, 115.6, 82.1, 57.0, 29.6, 28.4, 21.0. HRMS: (NSI+) *m/z* calcd for C_11_H_15_ClNO [M]^+^ 212.0832, found 212.0837.

#### (1*R*,2*R*)-2-(3-Chlorophenoxy)cyclopentan-1-aminium chloride (**6i**)

**6i** was synthesized according to general procedure C using **5i** (0.481 mmol) and HCl (4.81 mmol). The reaction was run for 48 h. **6i** was obtained as a solid (118 mg, 0.476 mmol, 99%). ^1^H NMR: (300 MHz, methanol-*d*_4_) δ 7.29 (t, *J* = 8.1, 1H), 7.06–6.91 (m, 3H), 4.90–4.80 (m, 1H), 3.78–3.69 (m, 1H), 2.38–2.23 (m, 2H), 1.99–1.67 (m, 4H). ^13^C NMR: (75 MHz, methanol-*d*_4_) δ 158.2, 134.6, 130.5, 121.2, 115.85, 113.9, 81.0, 57.0, 29.4, 28.1, 20.9. HRMS: (NSI+) *m/z* calcd for C_11_H_15_ClNO [M]^+^ 212.0830, found 212.0837.

#### (1*R*,2*R*)-2-(4-Chlorophenoxy)cyclopentan-1-aminium chloride (**6j**)

**6j** was synthesized according to general procedure C using **5j** (0.611 mmol) and HCl (12.22 mmol). The reaction was run for 1.5 h, after which a few drops of MeOH were added to dissolve the precipitate. Stirring was continued for another hour. **6j** was obtained as a solid (150 mg, 0.604 mmol, 99%). ^1^H NMR: (300 MHz, DMSO-*d*_6_) δ 8.59 (br s, 3H), 7.38–7.29 (m, 2H), 7.07–6.93 (m, 2H), 4.85–4.75 (m, 1H), 3.59–3.50 (m, 1H), 2.29–2.01 (m, 2H), 1.87–1.57 (m, 4H). ^13^C NMR: (75 MHz, DMSO-*d*_6_) δ 156.4, 129.8, 125.2, 117.8, 81.5, 56.4, 30.0, 28.7, 21.6. HRMS: (NSI+) *m/z* calcd for C_11_H_15_ClNO [M]^+^ 212.0840, found 212.0837.

#### (2*R*,3*R*,4*R*,5*R*)-2-(Acetoxymethyl)-5-(6-(((1*R*,2*R*)-2-((4-isopropylbenzyl)oxy)cyclopentyl)amino)-9*H*-purin-9-yl)tetrahydrofuran-3,4-diyl Diacetate (**8**)

**8** was synthesized according to general procedure D using **7** (0.286 mmol), **3b** (0.428 mmol), and NaHCO_3_ (0.857 mmol). The reaction was heated at reflux overnight. After purification with flash column chromatography (MeOH/EtOAc, 2%), **8** was obtained as a ∼1:1 mixture with starting material **7** (determined by ^1^H NMR). This mixture was used in the next step without further purification to obtain **15**.

#### (2*R*,3*R*,4*R*,5*R*)-2-(Acetoxymethyl)-5-(6-(((1*R*,2*R*)-2-((4-(*tert*-butyl)benzyl)oxy)cyclopentyl)amino)-9*H*-purin-9-yl)tetrahydrofuran-3,4-diyl Diacetate (**9**)

**9** was synthesized according to general procedure D using **7** (0.235 mmol), **3c** (0.352 mmol), and NaHCO_3_ (0.705 mmol). The reaction was heated at reflux for 15 h. After purification with flash column chromatography (EtOAc/cHex, 80%), **9** was obtained as a solid (53 mg, 0.0845 mmol, 36%). ^1^H NMR: (300 MHz, methanol-*d*_4_) δ 8.31 (s, 1H), 8.24 (s, 1H), 7.32 (d, *J* = 8.5, 2H), 7.23 (d, *J* = 8.4, 2H), 6.25 (d, *J* = 5.3, 1H), 6.03 (t, *J* = 5.5, 1H), 5.74–5.71 (m, 1H), 4.75–4.65 (m, 1H), 4.63 (s, 2H), 4.50–4.35 (m, 3H), 4.04–3.98 (m, 1H), 2.32–2.19 (m, 1H), 2.16 (s, 3H), 2.08 (s, 3H), 2.07 (s, 3H), 2.05–1.99 (m, 1H), 1.90–1.57 (m, 4H), 1.29 (s, 9H). ^13^C NMR: (101 MHz, methanol-*d*_4_) δ 170.8, 170.0, 169.7, 154.3, 152.9, 150.2, 148.5, 139.2, 135.5, 127.34, 124.7, 119.5, 86.4, 84.7, 80.2, 78.0, 73.0, 70.7, 70.6, 62.8, 33.9, 30.4, 30.3, 30.0, 21.1, 19.2, 19.0, 18.9. HRMS: (NSI+) *m/z* calcd for C_32_H_42_N_5_O_8_ [M + H]^+^ 624.3012, found 624.3028.

#### (2*R*,3*R*,4*R*,5*R*)-2-(Acetoxymethyl)-5-(6-(((1*R*,2*R*)-2-((4-cyanobenzyl)oxy)cyclopentyl)amino)-9*H*-purin-9-yl)tetrahydrofuran-3,4-diyl Diacetate (**10**)

**10** was synthesized according to general procedure D using **7** (0.83 mmol), **3d** (0.83 mmol), and NaHCO_3_ (2.5 mmol). The reaction was heated at reflux for 16 h. After purification with flash column chromatography (EtOAc/cHex, 80%), **10** was obtained as a solid (492 mg, 0.83 mmol, quant.). ^1^H NMR: (300 MHz, DMSO-*d*_6_) δ 8.37 (s, 1H), 8.27 (s, 1H), 7.99 (d, *J* = 6.0, 1H), 7.75 (d, *J* = 7.8, 2H), 7.47 (d, *J* = 7.8, 2H), 6.21 (d, *J* = 5.4, 1H), 6.04 (t, *J* = 5.6, 1H), 5.63 (t, *J* = 5.2, 1H), 4.70–4.52 (m, 1H), 4.66 (s, 2H), 4.45–4.32 (m, 2H), 4.28–4.19 (m, 1H), 4.02–3.87 (m, 1H), 2.15–1.88 (m, 2H), 2.12 (s, 3H), 2.04 (s, 3H), 2.01 (s, 3H), 1.81–1.54 (m, 4H). ^13^C NMR (101 MHz, CDCl_3_) δ 170.4, 169.7, 169.5, 153.8, 149.4, 144.5, 138.7, 132.2, 127.8, 120.3, 119.0, 111.2, 86.5, 85.69, 85.67, 80.5, 77.4, 73.4, 70.7, 70.5, 63.2, 31.02, 30.99, 30.5, 21.7, 20.9, 20.6, 20.5. HRMS: (NSI+) *m/z* calcd for C_29_H_33_N_6_O_8_ [M + H]^+^ 593.2360, found 593.2362.

#### (2*R*,3*R*,4*R*,5*R*)-2-(Acetoxymethyl)-5-(6-(((1*R*,2*R*)-2-((3-methoxybenzyl)oxy)cyclopentyl)amino)-9*H*-purin-9-yl)tetrahydrofuran-3,4-diyl Diacetate (**11**)

**11** was synthesized according to general procedure D using **7** (0.18 mmol), **3e** (0.271 mmol), and NaHCO_3_ (0.542 mmol). The reaction was heated at reflux for 23 h. After purification with flash column chromatography (EtOAc/cHex, 80%), **11** was obtained (68 mg, 0.114 mmol, 67%). ^1^H NMR: (300 MHz, methanol-*d*_4_) δ 8.37 (s, 1H), 8.26 (s, 1H), 7.98 (d, *J* = 7.9, 1H), 7.19 (t, *J* = 7.8, 1H), 6.89–6.75 (m, 3H), 6.22 (d, *J* = 5.4, 1H), 6.04 (t, *J* = 5.7, 1H), 5.64 (t, *J* = 5.3, 1H), 4.66–4.57 (m, 1H), 4.54 (s, 2H), 4.46–4.33 (m, 3H), 4.28–4.21 (m, 1H), 4.02–3.96 (m, 1H) 3.66 (s, 3H), 2.13 (s, 3H), 2.10–1.88 (m, 2H), 2.04 (s, 3H), 2.01 (s, 3H) 1.77–1.55 (m, 4H). ^13^C NMR: (101 MHz, methanol-*d*_4_) δ 170.8, 170.0, 169.8, 159.7, 154.3, 152.8, 148.5, 140.3, 139.2, 128.8, 119.7, 119.5, 112.62, 112.61, 86.4, 84.9, 80.2, 73.0, 70.9, 70.6, 62.8, 57.0, 54.1, 30.2, 30.0, 21.1, 19.2, 19.0, 18.9. HRMS: (NSI+) *m/z* calcd for C_29_H_36_N_5_O_9_ [M + H]^+^ 598.2506, found 598.2508.

#### (2*R*,3*R*,4*R*,5*R*)-2-(Acetoxymethyl)-5-(6-(((1*R*,2*R*)-2-phenoxycyclopentyl)amino)-9*H*-purin-9-yl)tetrahydrofuran-3,4-diyl Diacetate (**12**)

**12** was synthesized according to general procedure D using **7** (0.137 mmol), **6a** (0.206 mmol), and NaHCO_3_ (0.412 mmol). The reaction was heated at reflux for 18 h. After purification with flash column chromatography (EtOAc, 100%), **12** was obtained (50 mg, 0.09 mmol, 65%). ^1^H NMR: (300 MHz, methanol-*d*_4_) δ 8.30 (s, 1H), 8.22 (s, 1H), 7.22 (t, *J* = 7.7, 2H), 7.06–6.82 (m, 3H), 6.24 (d, *J* = 5.2, 1H), 6.02 (t, *J* = 5.6, 1H), 5.72 (t, *J* = 5.2, 1H), 4.85–4.73 (m, 2H), 4.50–4.33 (m, 3H), 2.38–2.18 (m, 2H), 2.15 (s, 3H), 2.07 (s, 6H), 1.98–1.69 (m, 4H). ^13^C NMR: (101 MHz, methanol-*d*_4_) δ 170.8, 170.0, 169.8, 158.0, 154.4, 152.8, 148.6, 139.4, 129.0, 120.3, 119.5, 115.4, 86.4, 82.5, 80.2, 73.0, 70.6, 62.8, 57.1, 30.0, 29.7, 21.1, 19.2, 19.0, 18.9. HRMS: (NSI+) *m/z* calcd for C_27_H_32_N_5_O_8_ [M + H]^+^ 554.2239, found 554.2245.

#### (2*R*,3*R*,4*R*,5*R*)-2-(Acetoxymethyl)-5-(6-(((1*R*,2*R*)-2-(3-methoxyphenoxy)cyclopentyl)amino)-9*H*-purin-9-yl)tetrahydrofuran-3,4-diyl Diacetate (**13**)

**13** was synthesized according to general procedure D using **7** (0.196 mmol), **6e** (0.197 mmol), and NaHCO_3_ (0.394 mmol). The reaction was heated at reflux for 23 h. After purification with flash column chromatography (EtOAc/cHex, 80%), **13** was obtained (63 mg, 0.108 mmol, 55%). ^1^H NMR: (300 MHz, DMSO-*d*_6_) δ 8.38 (s, 1H), 8.26 (s, 1H), 8.15 (d, *J* = 6.5, 1H), 7.14 (t, *J* = 8.1, 1H), 6.57–6.44 (m, 3H), 6.22 (d, *J* = 5.4, 1H), 6.07–6.01 (m, 1H), 5.66–5.61 (m, 1H), 4.89–4.83 (m, 1H), 4.70–4.60 (m, 1H), 4.46–4.34 (m, 2H), 4.28–4.20 (m, 1H), 3.67 (s, 3H), 2.23–2.13 (m, 2H), 2.12 (s, 3H), 2.04 (s, 3H), 2.02 (s, 3H), 1.89–1.62 (m, 4H). ^13^C NMR: (75 MHz, methanol-*d*_4_) δ 170.8, 170.0, 169.8, 160.9, 159.1, 154.3, 152.8, 139.4, 129.4, 119.5, 107.8, 106.0, 101.7, 86.4, 82.6, 80.2, 73.0, 70.6, 62.8, 57.0, 54.3, 30.0, 29.8, 21.1, 19.2, 19.1, 18.9. HRMS: (NSI+) *m/z* calcd for C_28_H_34_N_5_O_9_ [M + H]^+^ 584.2343, found 584.2351.

#### (2*R*,3*R*,4*R*,5*R*)-2-(Acetoxymethyl)-5-(6-(((1*R*,2*R*)-2-(4-chlorophenoxy)cyclopentyl)amino)-9*H*-purin-9-yl)tetrahydrofuran-3,4-diyl Diacetate (**14**)

**14** was synthesized according to general procedure D using **7** (0.347 mmol), **6j** (0.52 mmol), and NaHCO_3_ (1.04 mmol). The reaction was heated at reflux for 17 h. After purification with flash column chromatography (EtOAc/cHex, 80%), **14** was obtained (192 mg, 0.327 mmol, 82%). ^1^H NMR: (300 MHz, methanol-*d*_4_) δ 8.19 (s, 1H), 8.10 (s, 1H), 7.12–7.04 (m, 2H), 6.94–6.84 (m, 2H), 6.12 (d, *J* = 5.2, 1H), 5.91 (t, *J* = 5.5, 1H), 5.60 (t, *J* = 5.5, 1H), 4.68–4.63 (m, 1H), 4.63–4.55 (m, 1H), 4.35–4.22 (m, 3H), 2.23–2.05 (m, 2H), 2.03 (s, 3H), 1.95 (s, 6H), 1.87–1.55 (m, 4H). ^13^C NMR: (75 MHz, methanol-*d*_4_) δ 170.8, 170.0, 169.8, 156.7, 154.3, 152.8, 139.4, 128.8, 125.1, 119.5, 116.9, 86.4, 82.9, 80.2, 73.0, 70.6, 62.8, 56.9, 29.8, 29.6, 21.0, 19.3, 19.1, 18.9. HRMS: (NSI+) *m/z* calcd for C_27_H_31_ClN_5_O_8_ [M + H]^+^ 588.1854, found 588.1856.

#### (2*R*,3*S*,4*R*,5*R*)-2-(Hydroxymethyl)-5-(6-(((1*R*,2*R*)-2-((4-isopropylbenzyl)oxy)cyclopentyl)amino)-9*H*-purin-9-yl)tetrahydrofuran-3,4-diol (**15**)

**15** was synthesized according to general procedure E using the ∼1:1 mixture of **7** and **8** from above and K_2_CO_3_ (0.137 mmol). The reaction was run for 1.5 h. After purification with flash column chromatography (MeOH/EtOAc, 2%), **15** was obtained as a colorless solid (27 mg, 0.055 mmol, 19% over two steps). ^1^H NMR: (300 MHz, DMSO-*d*_6_) δ 8.37 (s, 1H), 8.23 (br s, 1H), 7.93 (d, *J* = 5.6, 1H), 7.22–7.12 (m, 4H), 5.89 (d, *J* = 6.1, 1H), 5.46–5.37 (m, 2H), 5.18 (d, *J* = 4.6, 1H), 4.66–4.44 (m, 3H), 4.62 (q, *J* = 6.0, 1H), 4.18–4.12 (m, 1H), 4.04–3.94 (m, 2H), 3.72–3.63 range (m, 1H), 3.61–3.50 (m, 1H), 2.84 (sept, *J* = 6.7, 1H), 2.13–1.88 (m, 2H), 1.78–1.56 (m, 4H), 1.17 (d, *J* = 6.9, 6H). HRMS: (NSI+) *m/z* calcd for C_25_H_34_N_5_O_5_ [M + H]^+^ 484.2539, found 484.2554. UPLC analysis: *^t^R* = 2.87 min; peak area > 95% (detection at 254 nm).

#### (2*R*,3*R*,4*S*,5*R*)-2-(6-(((1*R*,2*R*)-2-((4-(*tert*-Butyl)benzyl)oxy)cyclopentyl)amino)-9*H*-purin-9-yl)-5-(hydroxymethyl)tetrahydrofuran-3,4-diol (**16**)

**16** was synthesized according to general procedure E using **9** (0.083 mmol) and K_2_CO_3_ (0.05 mmol). The reaction was run for 30 min. After purification with flash column chromatography (MeOH/CH_2_Cl_2_, 5%), **16** was obtained as a colorless solid (30 mg, 0.06 mmol, 73%). ^1^H NMR: 300 MHz, methanol-*d*_4_) δ 8.16 (s, 2H), 7.21 (d, *J* = 8.4, 2H), 7.11 (d, *J* = 8.3, 2H), 5.85 (d, *J* = 6.5, 1H), 4.60–4.44 (m, 4H), 4.22 (dd, *J* = 5.1, 2.5, 1H), 4.06–4.09 (m, 1H), 3.87–3.91 (m, 1H), 3.79 (dd, *J* = 12.6, 2.5, 1H), 3.64 (dd, *J* = 12.5, 2.6, 1H), 2.20–2.09 (m, 1H), 1.99–1.85 (m, 1H), 1.80–1.45 (m, 3H), 1.58–1.18 (m, 1H) 1.18 (s, 9H). ^13^C NMR: (101 MHz, methanol-*d*_4_) δ 154.5, 152.2, 150.2, 147.8, 140.1, 135.5, 127.3, 124.7, 120.0, 90.0, 86.9, 84.6, 74.1, 71.3, 70.7, 62.1, 56.9, 33.9, 30.4, 30.2, 30.0, 21.1. HRMS: (NSI+) *m/z* calcd for C_26_H_36_N_5_O_5_ [M + H]^+^ 498.2690, found 498.2711. UPLC analysis: *^t^R* = 3.01 min; peak area > 95% (detection at 254 nm).

#### 4-((((1*R*,2*R*)-2-((9-((2*R*,3*R*,4*S*,5*R*)-3,4-Dihydroxy-5-(hydroxymethyl)tetrahydrofuran-2-yl)-9*H*-purin-6-yl)amino)cyclopentyl)oxy)methyl)benzonitrile (**17**)

**17** was synthesized according to general procedure E using **10** (0.552 mmol) and K_2_CO_3_ (0.552 mmol). The reaction was run for 3 h. After purification with flash column chromatography (EtOAc, 100%), **17** was obtained as a colorless solid (183 mg, 0.392 mmol, 71%). ^1^H NMR: (300 MHz, DMSO-d_6_) δ 8.37 (s, 1H), 8.23 (s, 1H), 7.93 (d, *J* = 6.0, 1H), 7.76 (d, *J* = 8.3, 2H), 7.48 (d, *J* = 8.0, 2H), 5.89 (d, *J* = 6.2, 1H), 5.42 (d, *J* = 6.3, 1H), 5.38 (dd, *J* = 7.1, 4.5, 1H), 5.18 (d, *J* = 4.7, 1H), 4.69–4.50 (m, 1H), 4.67 (s, 2H), 4.61 (q, *J* = 5.9, 1H), 4.28–4.11 (m, 1H), 4.08–3.88 (m, 2H), 3.68 (dt, *J* = 12.0, 4.1, 1H), 3.55 (ddd, *J* = 11.7, 7.2, 3.7, 1H), 2.10–2.01 (m, 1H), 2.00–1.89 (m, 1H), 1.89–1.55 (m, 4H). HRMS: (NSI+) *m/z* calcd for C_23_H_27_N_6_O_5_ [M + H]^+^ 467.2037, found 467.2031. UPLC analysis: *^t^R* = 2.28 min; peak area > 95% (detection at 254 nm).

#### 4-((((1*R*,2*R*)-2-((9-((2*R*,3*R*,4*S*,5*R*)-3,4-Dihydroxy-5-(hydroxymethyl)tetrahydrofuran-2-yl)-9*H*-purin-6-yl)amino)cyclopentyl)oxy)methyl)benzamide (**18**)

**10** (0.186 mmol) was dissolved in MeOH (15 mL) and K_2_CO_3_ (0.186 mmol) was added. The reaction mixture was stirred at room temperature for 4 h and concentrated under reduced pressure. The residue was dissolved in MeOH (1.5 mL), and aq. 30% H_2_O_2_ (0.15 mL) was added. The solution was heated to 40 °C and stirred for 3 h. The volatiles were removed under reduced pressure. After purification with flash column chromatography (MeOH/EtOAc, 20%), **18** was obtained as a colorless solid (75 mg, 0.155 mmol, 83%). ^1^H NMR: 400 MHz, methanol-*d*_4_) δ 8.24 (s, 2H), 7.76 (d, *J* = 8.3, 2H), 7.39 (d, *J* = 7.9, 2H), 5.95 (d, *J* = 6.5, 1H), 4.76 (dd, *J* = 6.5, 5.1, 1H), 4.75–4.56 (m, 1H), 4.71 (s, 2H), 4.33 (dd, *J* = 5.1, 2.5, 1H), 4.17 (q, *J* = 2.6, 1H), 4.00 (dt, *J* = 6.5, 4.1, 1H), 3.89 (dd, *J* = 12.6, 2.5, 1H), 3.75 (dd, *J* = 12.6, 2.7, 1H), 2.29–2.18 (m, 1H), 2.14–1.96 (m, 1H), 1.96–1.72 (m, 3H), 1.72–1.55 (m, 1H). ^13^C NMR: (101 MHz, methanol-*d*_4_) δ 172.1, 155.8, 153.5, 149.2, 144.5, 141.5, 133.8, 128.6, 128.4, 121.4, 91.4, 88.2, 86.6, 75.4, 72.7, 71.7, 63.5, 58.3, 31.5, 31.4, 22.5. HRMS: (NSI+) *m/z* calcd for C_23_H_28_N_6_NaO_6_ [M + Na]^+^ 485.2143, found 485.2156. UPLC analysis: *^t^R* = 1.79 min; peak area > 95% (detection at 254 nm).

#### (2*R*,3*S*,4*R*,5*R*)-2-(Hydroxymethyl)-5-(6-(((1*R*,2*R*)-2-((3-methoxybenzyl)oxy)cyclopentyl)amino)-9*H*-purin-9-yl)tetrahydrofuran-3,4-diol (**19**)

**19** was synthesized according to general procedure E using **11** (0.084 mmol) and K_2_CO_3_ (0.05 mmol). The reaction was run for 30 min. After purification with flash column chromatography (MeOH/EtOAc, 5–10%), **19** was obtained as a colorless solid (36 mg, 0.076 mmol, 93%). ^1^H NMR: (300 MHz, DMSO-*d*_6_) δ 8.36 (s, 1H), 8.23 (s, 1H), 7.93 (d, *J* = 4.9, 1H), 7.20 (t, *J* = 7.7, 1H), 6.89–6.73 (m, 3H), 5.89 (d, *J* = 6.2, 1H), 5.46–5.37 (m, 2H), 5.18 (d, *J* = 4.6, 1H), 4.71–4.56 (m, 2H), 4.54 (s, 2H), 4.18–4.11 (m, 1H), 4.06–3.92 (m, 2H), 3.72–3.67 (m, 1H), 3.67 (s, 3H), 3.61–3.52 (m, 1H), 2.12–1.89 (m, 2H), 1.76–1.57 (m, 4H). ^13^C NMR: (101 MHz, methanol-*d*_4_) δ 159.8, 154.4, 152.1, 147.8, 140.2, 140.0, 128.8, 120.0, 119.5, 112.7, 112.6, 90.0, 86.9, 84.8, 74.1, 71.3, 70.8, 62.1, 56.7, 54.1, 30.1, 30.0, 21.1*.* HRMS: (NSI+) *m/z* calcd for C_23_H_30_N_5_O_6_ [M + H]^+^ 472.2186, found 472.2191. UPLC analysis: *^t^R* = 2.33 min; peak area > 95% (detection at 254 nm).

#### (2*R*,3*R*,4*S*,5*R*)-2-(6-(((1*R*,2*R*)-2-((3-Bromobenzyl)oxy)cyclopentyl)amino)-9*H*-purin-9-yl)-5-(hydroxymethyl)tetrahydrofuran-3,4-diol (**20**)

**20** was synthesized according to general procedure G using **7** (0.296 mmol), **3f** (0.326 mmol), and NaHCO_3_ (0.652 mmol). The reaction was heated at reflux for 18 h. After purification with flash column chromatography (EtOAc, 100%) a mixture of the substitution product and fully and semi-deacetylated products was isolated (53 mg). This mixture was dissolved in MeOH (5 mL), and K_2_CO_3_ (0.05 mmol) was added. After stirring for 30 min at room temperature, the reaction mixture was filtered and concentrated under reduced pressure. After purification with flash column chromatography (MeOH/EtOAc, 5%), **20** was obtained as a colorless solid (33 mg, 0.063 mmol, 66% over two steps). ^1^H NMR: (300 MHz, methanol-*d*_4_) δ 8.15 (s, 1H), 8.14 (s, 1H), 7.37–7.32 (m, 1H), 7.25–6.99 (m, 3H), 5.85 (d, *J* = 6.5, 1H), 4.69–4.62 (m, 1H), 4.62–4.45 (m, 3H), 4.25–4.20 (m, 1H), 4.07 (q, *J* = 2.5, 1H), 3.89–3.82 (m, 1H), 3.79 (dd, *J* = 12.6, 2.4, 1H), 3.64 (dd, *J* = 12.6, 2.6, 1H), 2.19–2.05 (m, 1H), 1.97–1.85 (m, 1H), 1.81–1.45 (m, 4H). ^13^C NMR: (75 MHz, methanol-*d*_4_) δ 154.4, 152.2, 141.5, 140.1, 130.1, 130.0, 129.6, 125.8, 121.9, 120.0, 90.0, 86.9, 85.1, 74.1, 71.3, 70.0, 62.2, 56.7, 30.1, 29.9, 21. HRMS: (NSI+) *m/z* calcd for C_22_H_27_BrN_5_O_5_ [M + H]^+^ 520.1189, found 520.1189. UPLC analysis: *^t^R* = 2.58 min; peak area > 95% (detection at 254 nm).

#### (2*R*,3*R*,4*S*,5*R*)-2-(6-(((1*R*,2*R*)-2-((4-Bromobenzyl)oxy)cyclopentyl)amino)-9*H*-purin-9-yl)-5-(hydroxymethyl)tetrahydrofuran-3,4-diol (**21**)

**21** was synthesized according to general procedure G using **7** (0.209 mmol), **3g** (0.209 mmol), and NaHCO_3_ (0.417 mmol). The reaction was heated at reflux for 18 h. The crude material was deacetylated using K_2_CO_3_ (0.125 mmol) in MeOH for 30 min at room temperature. After purification with flash column chromatography (MeOH/EtOAc, 5%), **21** was obtained (25 mg, 0.048 mmol, 23% over two steps). ^1^H NMR (300 MHz, methanol-*d*_4_) δ 8.15 (s, 1H), 8.14 (s, 1H), 7.29 (d, *J* = 8.4, 2H), 7.11 (d, *J* = 8.3, 2H), 5.85 (d, *J* = 6.4, 1H), 4.65 (dd, *J* = 6.4, 5.1, 1H), 4.62–4.51 (m, 1H), 4.49 (s, 2H), 4.23 (dd, *J* = 5.1, 2.5, 1H), 4.07 (q, *J* = 2.5, 1H), 3.90–3.84 (m, 1H), 3.79 (dd, *J* = 12.6, 2.5, 1H), 3.64 (dd, *J* = 12.6, 2.6, 1H), 2.19–2.07 (m, 1H), 1.97–1.84 (m, 1H), 1.80–1.44 (m, 4H). ^13^C NMR: (75 MHz, methanol-*d*_4_) δ 154.4, 152.1, 140.1, 138.1, 130.9, 129.1, 120.7, 120.0, 90.0, 86.9, 85.0, 74.1, 71.3, 70.1, 62.1, 56.9, 30.1, 30.0, 21. HRMS: (NSI+) *m/z* calcd for C_22_H_27_BrN_5_O_5_ [M + H]^+^ 520.1187, found 520.1190. UPLC analysis: *^t^R* = 2.64 min; peak area > 99% (detection at 254 nm).

#### (2*R*,3*R*,4*S*,5*R*)-2-(6-(((1*R*,2*R*)-2-((2-Chlorobenzyl)oxy)cyclopentyl)amino)-9*H*-purin-9-yl)-5-(hydroxymethyl)tetrahydrofuran-3,4-diol (**22**)

**22** was synthesized according to general procedure G using **7** (0.229 mmol), **3h** (0.229 mmol), and NaHCO_3_ (0.458 mmol). The reaction was heated at reflux for 19 h. The crude material was deacetylated using K_2_CO_3_ (0.22 mmol) in MeOH for 30 min at room temperature. After purification with flash column chromatography (MeOH/EtOAc, 5%), **22** was obtained (51 mg, 0.107 mmol, 47% over two steps). ^1^H NMR: (300 MHz, methanol-*d*_4_) δ 8.13 (br s, 2H), 7.39–7.32 (m, 1H), 7.22–7.02 (m, 3H), 5.84 (d, *J* = 6.5, 1H), 4.71–4.47 (m, 4H), 4.25–4.20 (m, 1H), 4.07 (q, *J* = 2.5, 1H), 3.94–3.87 (m, 1H), 3.84–3.72 (m, 1H), 3.67–3.59 (m, 1H), 2.20–2.06 (m, 1H), 2.00–1.85 (m, 1H), 1.82–1.62 (m, 3H), 1.61–1.45 (m, 1H). ^13^C NMR: (75 MHz, methanol-*d*_4_) δ 154.4, 152.1, 147.8, 140.0, 136.3, 132.5, 128.9, 128.7, 128.4, 126.5, 120.0, 90.0, 86.9, 85.5, 74.1, 71.3, 68.1, 62.2, 56.7, 30.1, 30.0, 21.2. HRMS: (NSI+) *m/z* calcd for C_22_H_27_ClN_5_O_5_ [M + H]^+^ 476.1673, found 476.1695. UPLC analysis: *^t^R* = 2.51 min; peak area > 99% (detection at 254 nm).

#### (2*R*,3*R*,4*S*,5*R*)-2-(6-(((1*R*,2*R*)-2-((3-Chlorobenzyl)oxy)cyclopentyl)amino)-9*H*-purin-9-yl)-5-(hydroxymethyl)tetrahydrofuran-3,4-diol (**23**)

**23** was synthesized according to general procedure G using **7** (0.336 mmol), **3i** (0.336 mmol), and NaHCO_3_ (0.732 mmol). The reaction was heated at reflux for 19 h. The crude material was deacetylated using K_2_CO_3_ (0.22 mmol) in MeOH for 30 min at room temperature. After purification with flash column chromatography (MeOH/EtOAc, 4%), **23** was obtained (45 mg, 0.095 mmol, 26% over two steps). ^1^H NMR: (300 MHz, methanol-*d*_4_) δ 8.14 (s, 1H), 8.13 (s, 1H), 7.19 (s, 1H), 7.04–7.03 (m, 3H), 5.85 (d, *J* = 6.4, 1H), 4.69–4.61 (m, 1H), 4.61–4.44 (m, 3H), 4.26–4.20 (m, 1H), 4.07 (q, *J* = 2.5, 1H), 3.89–3.82 (m, 1H), 3.82–3.74 (m, 1H), 3.69–3.60 (m, 1H), 2.18–2.05 (m, 1H), 1.96–1.79 (m, 2H), 1.78–1.45 (m, 4H). ^13^C NMR: (75 MHz, methanol-*d*_4_) δ 154.4, 152.2, 148.0, 141.3, 140.1, 133.8, 129.3, 127.1, 127.0, 125.4, 120.0, 90.0, 86.9, 85.1, 74.1, 71.3, 70.0, 62.15, 56.8, 30.1, 29.9, 21.1. HRMS: (NSI+) *m/z* calcd for C_22_H_27_ClN_5_O_5_ [M + H]^+^ 476.1699, found 476.1695. UPLC analysis: *^t^R* = 2.56 min; peak area > 99% (detection at 254 nm).

#### (2*R*,3*S*,4*R*,5*R*)-2-(Hydroxymethyl)-5-(6-(((1*R*,2*R*)-2-phenoxycyclopentyl)amino)-9*H*-purin-9-yl)tetrahydrofuran-3,4-diol (**24**)

**24** was synthesized according to general procedure E using **12** (0.089 mmol) and K_2_CO_3_ (0.053 mmol). The reaction was run for 30 min. After purification with flash column chromatography (EtOAc, 100%), **24** was obtained as a colorless solid (16 mg, 0.038 mmol, 42%). ^1^H NMR (400 MHz, methanol-*d*_4_) δ 8.15 (s, 1H), 8.15 (s, 1H), 7.11 (t, *J* = 7.9, 2H), 6.94–6.87 (m, 2H), 6.77 (t, *J* = 7.3, 1H), 5.85 (d, *J* = 6.4, 1H), 4.72–4.67 (m, 1H), 4.66–4.54 (m, 2H), 4.22 (dd, *J* = 5.1, 2.5, 1H), 4.07 (q, *J* = 2.6, 1H), 3.79 (dd, *J* = 12.6, 2.5, 1H), 3.64 (dd, *J* = 12.5, 2.7, 1H), 2.26–2.05 (m, 2H), 1.60–1.56 (m, 4H). ^13^C NMR: (75 MHz, methanol-*d*_4_) δ 158.0, 154.5, 152.1, 148.0, 140.1, 129.0, 120.4, 120.0, 115.4, 89.9, 86.8, 82.5, 74.1, 71.3, 62.1, 57.2, 29.9, 29.7, 21.0. HRMS: (NSI+) *m/z* calcd for C_21_H_26_N_5_O_5_ [M + H]^+^ 428.1924, found 428.1928. HPLC analysis: *^t^R* = 7.22 min; peak area 95% (detection at 254 nm).

#### (2*R*,3*R*,4*S*,5*R*)-2-(6-(((1*R*,2*R*)-2-(4-(*tert*-Butyl)phenoxy)cyclopentyl)amino)-9*H*-purin-9-yl)-5-(hydroxymethyl)tetrahydrofuran-3,4-diol (**25**)

**25** was synthesized according to general procedure G using **7** (0.241 mmol), **6c** (0.241 mmol), and NaHCO_3_ (0.482 mmol). The reaction was heated at reflux for 19 h. The crude material was deacetylated using K_2_CO_3_ (0.144 mmol) in MeOH for 30 min at room temperature. After purification with flash column chromatography (MeOH/EtOAc, 3%), **25** was obtained (46 mg, 0.095 mmol, 40% over two steps). ^1^H NMR: (300 MHz, methanol-*d*_4_) δ 8.14 (s, 1H), 8.13 (s, 1H), 7.16–7.09 (m, 2H), 6.84–6.77 (m, 2H), 5.85 (d, *J* = 6.4, 1H), 4.68–4.51 (m, 3H), 4.25–4.20 (dd, *J* = 5.1, 2.5, 1H), 4.07 (q, *J* = 2.3, 1H), 3.78 (dd, *J* = 12.6, 2.5, 1H), 3.63 (dd, *J* = 12.6, 2.7, 1H), 2.24–1.98 (m, 2H), 1.85–1.54 (m, 4H), 1.14 (s, 9H). ^13^C NMR: (75 MHz, methanol-*d*_4_) δ 155.6, 154.4, 152.1, 147.9, 143.1, 140.1, 125.7, 120.0, 115.0, 90.0, 86.8, 82.5, 74.1, 71.3, 62.1, 57.0, 33.5, 30.6, 30.0, 29.7, 21.1. HRMS: (NSI+) *m/z* calcd for C_25_H_34_N_5_O_5_ [M + H]^+^ 484.2568, found 484.2554. UPLC analysis: *^t^R* = 3.21 min; peak area > 99% (detection at 254 nm).

#### (2*R*,3*S*,4*R*,5*R*)-2-(Hydroxymethyl)-5-(6-(((1*R*,2*R*)-2-(3-methoxyphenoxy)cyclopentyl)amino)-9*H*-purin-9-yl)tetrahydrofuran-3,4-Diol (**26**)

**26** was synthesized according to general procedure E using **13** (0.12 mmol) and K_2_CO_3_ (0.072 mmol). The reaction was run for 30 min. After purification with flash column chromatography (EtOAc, 100%), **26** was obtained as a solid (36 mg, 0.079 mmol, 66%). ^1^H NMR: (300 MHz, DMSO-*d*_6_) δ 8.38 (s, 1H), 8.23 (s, 1H), 8.10 (d, *J* = 7.6, 1H), 7.14 (t, *J* = 8.1, 1H), 6.57–6.42 (m, 3H), 5.89 (d, *J* = 6.1, 1H), 5.44 (d, *J* = 6.2, 1H), 5.41–5.35 (m, 1H), 5.19 (d, *J* = 4.7, 1H), 4.90–4.82 (m, 1H), 4.74–4.64 (m, 1H), 4.64–4.56 (m, 1H), 4.15 (q, *J* = 4.5, 1H), 3.99–3.94 (m, 1H), 3.73–3.64 (m, 1H) 3.68 (s, 3H), 3.60–3.51 (m, 1H), 2.23–2.09 (m, 2H), 1.87–1.62 (m, 4H). ^13^C NMR: (75 MHz, methanol-*d*_4_) δ 160.9, 159.1, 154.4, 152.1, 140.1, 129.4, 120.0, 107.8, 105.9, 101.7, 90.0, 86.8, 82.6, 74.1, 71.3, 62.1, 57.1, 54.3, 29.9, 29.8, 21.1. HRMS: (NSI+) *m/z* calcd for C_22_H_28_N_5_O_6_ [M + H]^+^ 458.2030, found 458.2034. UPLC analysis: *^t^R* = 2.50 min; peak area > 95% (detection at 254 nm).

#### (2*R*,3*R*,4*S*,5*R*)-2-(6-(((1*R*,2*R*)-2-(3-Bromophenoxy)cyclopentyl)amino)-9*H*-purin-9-yl)-5-(hydroxymethyl)tetrahydrofuran-3,4-diol (**27**)

**27** was synthesized according to general procedure G using **7** (0.12 mmol), **6f** (0.12 mmol), and NaHCO_3_ (0.239 mmol). The reaction was heated at reflux for 18 h. After purification with flash column chromatography (EtOAc, 100%), a mixture of the substitution product and fully and semi-deacetylated products was isolated (40 mg). This mixture was dissolved in MeOH (5 mL), and K_2_CO_3_ (0.033 mmol) was added. After stirring for 30 min at room temperature, the reaction mixture was filtered and concentrated under reduced pressure. After purification with flash column chromatography (EtOAc, 100%), **27** was obtained as a solid (21 mg, 0.042 mmol, 35% over two steps). ^1^H NMR (300 MHz, methanol-*d*_4_) δ 8.24 (s, 1H), 8.16 (s, 1H), 7.49 (br s, 1H), 7.02 (t, *J* = 8.0, 1H), 6.96–6.82 (m, 2H), 5.86 (d, *J* = 6.4, 1H), 4.70–4.62 (m, 2H), 4.61–4.51 (m, 1H), 4.23 (dd, *J* = 5.1, 2.5, 1H), 4.09–4.05 (m, 1H), 3.79 (dd, *J* = 12.6, 2.5, 1H), 3.65 (dd, *J* = 12.5, 2.6, 1H), 2.23–1.97 (m, 2H), 1.90–1.58 (m, 4H). ^13^C NMR: (75 MHz, methanol-*d*_4_) δ 158.9, 154.3, 152.2, 140.2, 130.3, 123.3, 122.3, 120.0, 118.4, 114.8, 90.0, 86.9, 82.8, 74.1, 71.3, 62.1, 56.6, 29.6, 29.4, 21.0. HRMS: (NSI+) *m/z* calcd for C_21_H_25_BrN_5_O_5_ [M + H]^+^ 506.1025, found 506.1034. UPLC analysis: *^t^R* = 2.83 min; peak area > 95% (detection at 254 nm).

#### (2*R*,3*R*,4*S*,5*R*)-2-(6-(((1*R*,2*R*)-2-(2-Chlorophenoxy)cyclopentyl)amino)-9*H*-purin-9-yl)-5-(hydroxymethyl)tetrahydrofuran-3,4-diol (**28**)

**28** was synthesized according to general procedure G using **7** (0.322 mmol), **6 h** (0.322 mmol), and NaHCO_3_ (0.644 mmol). The reaction was heated at reflux for 18 h. The crude material was deacetylated using K_2_CO_3_ (0.193 mmol) in MeOH for 45 min at room temperature. After purification with flash column chromatography (EtOAc, 100%), **28** was obtained (29 mg, 0.063 mmol, 20% over two steps). ^1^H NMR (300 MHz, methanol-*d*_4_) δ 8.15 (s, 1H), 8.14 (s, 1H), 7.30–7.01 (m, 3H), 6.80–6.71 (m, 1H), 5.85 (d, *J* = 6.4, 1H), 4.76–4.72 (m, 1H), 4.70–4.60 (m, *J* = 6.4, 5.1, 2H), 4.22 (dd, *J* = 5.1, 2.5, 1H), 4.07 (q, *J* = 2.5, 1H), 3.79 (dd, *J* = 12.6, 2.5, 1H), 3.64 (dd, *J* = 12.6, 2.6, 1H), 2.31–2.19 (m, 1H), 2.13–2.00 (m, 1H), 1.93–1.75 (m, 3H), 1.70–1.57 (m, 1H). ^13^C NMR: (75 MHz, methanol-*d*_4_) δ 154.4, 153.5, 152.1, 140.2, 129.8, 127.4, 123.2, 121.3, 120.0, 115.6, 89.9, 86.8, 83.8, 74.1, 71.3, 62.1, 56.9, 29.7, 29.6, 21.1. HRMS: (NSI+) *m/z* calcd for C_21_H_25_ClN_5_O_5_ [M + H]^+^ 462.1529, found 462.1539. UPLC analysis: *^t^R* = 2.72 min; peak area > 99% (detection at 254 nm).

#### (2*R*,3*R*,4*S*,5*R*)-2-(6-(((1*R*,2*R*)-2-(3-Chlorophenoxy)cyclopentyl)amino)-9*H*-purin-9-yl)-5-(hydroxymethyl)tetrahydrofuran-3,4-diol (**29**)

**29** was synthesized according to general procedure G using **7** (0.234 mmol), **6i** (0.234 mmol), and NaHCO_3_ (0.467 mmol). The reaction was heated at reflux for 18 h. The crude material was deacetylated using K_2_CO_3_ (0.138 mmol) in MeOH for 2 h at room temperature. After purification with flash column chromatography (MeOH/EtOAc, 6%), **29** was obtained (36 mg, 0.078 mmol, 33% over two steps). ^1^H NMR: (300 MHz, DMSO-d_6_) δ 8.39 (s, 1H), 8.27 (s, 1H), 8.09 (d, *J* = 7.1, 1H), 7.28 (t, *J* = 8.2, 2H), 7.00–6.91 (m, 2H), 5.90 (d, *J* = 6.1, 1H), 5.43 (d, *J* = 6.2, 1H), 5.41–5.34 (m, 1H), 5.19 (d, *J* = 4.7, 1H), 4.91–4.83 (m, 1H), 4.71–4.57 (m, 2H), 4.15 (q, *J* = 4.7, 2H), 4.00–3.95 (m, 1H), 3.73–3.64 (m, 1H), 3.61–3.51 (m, 1H), 2.22–2.07 (m, 2H), 1.88–1.64 (m, 4H). ^13^C NMR: (101 MHz, methanol-*d*_4_) δ 158.9, 154.4, 152.1, 152.1, 140.2, 134.4, 130.0, 120.3, 120.0, 115.6, 114.3, 90.0, 86.8, 82.9, 74.1, 71.3, 62.1, 56.8, 29.6, 29.5, 21.0. HRMS: (NSI+) *m/z* calcd for C_21_H_25_ClN_5_O_5_ [M + H]^+^ 462.1543, found 462.1539. HPLC analysis: *^t^R* = 7.16 min; peak area 94% (detection at 254 nm).

#### (2*R*,3*R*,4*S*,5*R*)-2-(6-(((1*R*,2*R*)-2-(4-Chlorophenoxy)cyclopentyl)amino)-9*H*-purin-9-yl)-5-(hydroxymethyl)tetrahydrofuran-3,4-diol (**30**)

**30** was synthesized according to general procedure E using **14** (0.325 mmol) and K_2_CO_3_ (0.195 mmol). The reaction was run for 45 min. After purification with flash column chromatography (MeOH/EtOAc, 10%), **30** was obtained as a solid (54 mg, 0.117 mmol, 36%). ^1^H NMR: (300 MHz, DMSO-*d*_6_) δ 8.38 (s, 1H), 8.25 (s, 1H), 8.08 (d, *J* = 7.5, 1H), 7.30 (d, *J* = 8.9, 2H), 7.03 (d, *J* = 8.3, 2H), 5.90 (d, *J* = 6.1, 1H), 5.43 (d, *J* = 6.0, 1H), 5.40–5.33 (m, 1H), 5.18 (d, *J* = 4.6, 1H), 4.87–4.80 (m, 1H), 4.74–4.56 (m, 2H), 4.15 (q, *J* = 4.2, 1H), 3.97 (dd, *J* = 9.9, 3.3, 2H), 3.72–3.63 (m, 1H), 3.59–3.50 (m, 1H), 2.24–2.06 (m, 2H), 1.88–1.60 (m, 4H). ^13^C NMR: (75 MHz, methanol-*d*_4_) δ 156.7, 154.4, 152.1, 148.0, 140.2, 128.8, 125.2, 120.0, 116.9, 89.9, 86.8, 82.9, 74.1, 71.3, 62.1, 57.0, 29.7, 29.6, 21.0. HRMS: (NSI+) *m/z* calcd for C_21_H_25_ClN_5_O_5_ [M + H]^+^ 462.1525, found 462.1539. UPLC analysis: *^t^R* = 2.81 min; peak area > 99% (detection at 254 nm).

#### (3a*S*,4*S*,6*R*,6a*R*)-*N*-Ethyl-6-(6-(((1*R*,2*R*)-2-((3-methoxybenzyl)oxy)cyclopentyl)amino)-9*H*-purin-9-yl)-2,2-dimethyltetrahydrofuro[3,4-*d*][1,3]dioxole-4-carboxamide (**32**)

**32** was synthesized according to general procedure D using **31** (0.256 mmol), **3e** (0.256 mmol), and NaHCO_3_ (0.698 mmol). The reaction was heated at reflux for 19 h. After purification with flash column chromatography (MeOH/EtOAc, 3%), **32** was obtained (92 mg, 0.166 mmol, 71%). ^1^H NMR: (300 MHz, methanol-*d*_4_) δ 8.25 (s, 1H), 8.17 (s, 1H), 7.16 (t, *J* = 8.1, 1H), 6.89–6.82 (m, 2H), 6.77–6.72 (m, 1H), 6.32 (d, *J* = 1.4, 1H), 5.60 (dd, *J* = 6.1, 1.9, 1H), 5.48 (dd, *J* = 6.1, 1.0, 1H), 4.72–4.57 (m, 1H), 4.63 (d, *J* = 1.9, 1H), 4.60 (s, 2H), 4.01–3.94 (m, 1H), 3.71 (s, 3H), 2.95–2.74 (m, 2H), 2.30–2.17 (m, 1H), 2.06–1.96 (m, 1H), 1.91–1.69 (m, 3H), 1.67–1.59 (m, 1H), 1.58 (s, 3H), 1.39 (s, 3H), 0.63 (t, *J* = 7.3, 3H). ^13^C NMR: (75 MHz, methanol-*d*_4_) δ 170.0, 159.7, 154.3, 152.7, 148.3, 140.4, 140.3, 128.9, 119.5, 119.3, 113.5, 112.7, 112.5, 91.0, 87.2, 84.8, 83.8, 83.7, 70.7, 56.8, 54.2, 33.4, 30.2, 30.0, 25.8, 24.0, 21.1, 12.8. HRMS: (NSI+) *m/z* calcd for C_28_H_37_N_6_O_6_ [M + H]^+^ 553.2774, found 553.2769.

#### (3a*S*,4*S*,6*R*,6a*R*)-6-(6-(((1*R*,2*R*)-2-((3-Bromobenzyl)oxy)cyclopentyl)amino)-9*H*-purin-9-yl)-*N*-ethyl-2,2-dimethyltetrahydrofuro[3,4-*d*][1,3]dioxole-4-carboxamide (**33**)

**33** was synthesized according to general procedure D using **31** (0.359 mmol), **3f** (0.326 mmol), and NaHCO_3_ (0.978 mmol). The reaction was heated at reflux for 18 h. After purification with flash column chromatography (EtOAc, 100%), **33** was obtained (119 mg, 0.198 mmol, 55%). ^1^H NMR: (300 MHz, methanol-*d*_4_) δ 8.14 (s, 1H), 8.07 (s, 1H), 7.32 (br s, 1H), 7.23–6.97 (m, 3H), 6.20 (d, *J* = 1.6, 1H), 5.47 (dd, *J* = 6.1, 1.9, 1H), 5.39–5.32 (m, 1H), 4.62–4.42 (m, 4H), 3.87–3.80 (m, 1H) 2.85–2.65 (m, 2H), 2.17–2.02 (m, 1H), 1.94–1.82 (m, 1H), 1.77–1.56 (m, 3H), 1.55–1.46 (m, 1H), 1.45 (s, 3H), 1.27 (s, 3H), 0.52 (t, *J* = 7.3, 3H). ^13^C NMR: (75 MHz, methanol-*d*_4_) δ 170.0, 154.2, 152.7, 148.2, 141.5, 140.5, 130.0, 130.0, 129.7, 125.8, 121.9, 119.3, 113.5, 91.0, 87.2, 85.0, 83.8, 83.7, 69.9, 56.7, 33.4, 30.2, 30.0, 25.8, 24.1, 21.1, 12.8. HRMS: (NSI+) *m/z* calcd for C_27_H_34_BrN_6_O_5_ [M + H]^+^ 601.1767, found 601.1796.

#### (3a*S*,4*S*,6*R*,6a*R*)-6-(6-(((1*R*,2*R*)-2-((4-Bromobenzyl)oxy)cyclopentyl)amino)-9*H*-purin-9-yl)-*N*-ethyl-2,2-dimethyltetrahydrofuro[3,4-*d*][1,3]dioxole-4-carboxamide (**34**)

**34** was synthesized according to general procedure D using **31** (0.163 mmol), **3g** (0.163 mmol), and NaHCO_3_ (0.489 mmol). The reaction was heated at reflux for 17 h. After purification with flash column chromatography (EtOAc, 100%), **34** was obtained (65 mg, 0.108 mmol, 66%). ^1^H NMR: (300 MHz, methanol-*d*_4_) δ 8.12 (s, 1H), 8.07 (s, 1H), 7.29–7.22 (m, 2H), 7.13–7.06 (m, 2H), 6.21 (d, *J* = 1.4, 1H), 5.49 (dd, *J* = 6.1, 1.9, 1H), 5.37 (dd, *J* = 6.1, 1.4, 1H), 4.60–4.42 (m, 4H), 3.90–3.82 (m, 1H), 2.84–2.63 (m, 2H), 2.18–2.04 (m, 1H), 1.98–1.83 (m, 1H), 1.76–1.56 (m, 3H), 1.54–1.42 (m, 1H), 1.46 (s, 3H), 1.28 (s, 3H), 0.51 (t, *J* = 7.3, 3H). ^13^C NMR: (75 MHz, methanol-*d*_4_) δ 170.1, 154.2, 152.7, 148.3, 140.5, 138.1, 130.9, 129.1, 120.7, 119.3, 113.5, 91.0, 87.3, 85.0, 83.9, 83.7, 70.1, 56.7, 33.4, 30.2, 30.0, 25.7, 24.0, 21.1, 12.8. HRMS: (NSI+) *m/z* calcd for C_27_H_34_BrN_6_O_5_ [M + H]^+^ 601.1789, found 601.1769.

#### (3a*S*,4*S*,6*R*,6a*R*)-6-(6-(((1*R*,2*R*)-2-((2-Chlorobenzyl)oxy)cyclopentyl)amino)-9*H*-purin-9-yl)-*N*-ethyl-2,2-dimethyltetrahydrofuro[3,4-*d*][1,3]dioxole-4-carboxamide (**35**)

**35** was synthesized according to general procedure D using **31** (0.221 mmol), **3h** (0.221 mmol), and NaHCO_3_ (0.663 mmol). The reaction was heated at reflux for 19 h. After purification with flash column chromatography (EtOAc/cHex, 90%), **35** was obtained (79 mg, 0.141 mmol, 64%). ^1^H NMR: (300 MHz, methanol-*d*_4_) δ 8.13 (s, 1H), 8.06 (s, 1H), 7.40–7.31 (m, 1H), 7.21–7.02 (m, 3H), 6.21 (d, *J* = 1.4, 1H), 5.48 (dd, *J* = 6.1, 1.9, 1H), 5.35 (dd, *J* = 6.1, 1.4, 1H), 4.69–4.52 (m, 3H), 4.51 (d, *J* = 1.9, 1H), 3.94–3.86 (m, 1H), 2.84–2.62 (m, 2H), 2.22–2.05 (m, 1H), 2.00–1.86 (m, 1H), 1.85–1.58 (m, 3H), 1.57–1.45 (m, 1H), 1.45 (s, 3H), 1.27 (s, 3H), 0.51 (t, *J* = 7.3, 3H). ^13^C NMR: (75 MHz, methanol-*d*_4_) δ 170.1, 154.2, 152.7, 148.3, 140.5, 136.3, 132.4, 128.9, 128.8, 128.4, 126.5, 119.3, 113.5, 91.0, 87.2, 85.4, 83.8, 83.7, 68.1, 56.9, 33.4, 30.2, 30.0, 25.7, 24.0, 21.2, 12.8. HRMS: (NSI+) *m/z* calcd for C_27_H_34_ClN_6_O_5_ [M + H]^+^ 557.2265, found 557.2274.

#### (3a*S*,4*S*,6*R*,6a*R*)-6-(6-(((1*R*,2*R*)-2-((3-Chlorobenzyl)oxy)cyclopentyl)amino)-9*H*-purin-9-yl)-*N*-ethyl-2,2-dimethyltetrahydrofuro[3,4-*d*][1,3]dioxole-4-carboxamide (**36**)

**36** was synthesized according to general procedure D using **31** (0.305 mmol), **3i** (0.305 mmol), and NaHCO_3_ (0.915 mmol). The reaction was heated at reflux for 18 h. After purification with flash column chromatography (EtOAc, 100%), **36** was obtained (115 mg, 0.206 mmol, 68%). ^1^H NMR: (300 MHz, methanol-*d*_4_) δ 8.26 (s, 1H), 8.19 (s, 1H), 7.30 (br s, 1H), 7.27–7.14 (m, 3H), 6.33 (d, *J* = 1.5, 1H), 5.60 (dd, *J* = 6.1, 1.9, 1H), 5.48 (dd, *J* = 6.1, 1.5, 1H), 4.72–4.57 (m, 4H), 4.00–3.93 (m, 1H), 2.98–2.75 (m, 2H), 2.30–2.17 (m, 1H), 2.08–1.96 (m, 1H), 1.92–1.59 (m, 4H), 1.57 (s, 3H), 1.39 (s, 3H), 0.64 (t, *J* = 7.3, 3H). ^13^C NMR: (75 MHz, methanol-*d*_4_) δ 170.0, 154.2, 152.7, 148.2, 141.3, 140.5, 133.7, 129.4, 127.1, 127.0, 125.4, 119.3, 113.5, 91.0, 87.2, 85.0, 83.8, 83.7, 70.0, 56.8, 33.4, 30.2, 29.9, 25.8, 24.0, 21.1, 12.8. HRMS: (NSI+) *m/z* calcd for C_27_H_34_ClN_6_O_5_ [M + H]^+^ 557.2277, found 557.2274.

#### (3a*S*,4*S*,6*R*,6a*R*)-*N*-Ethyl-2,2-dimethyl-6-(6-(((1*R*,2*R*)-2-phenoxycyclopentyl)amino)-9*H*-purin-9-yl)tetrahydrofuro[3,4-*d*][1,3]dioxole-4-arboxamide (**37**)

**37** was synthesized according to general procedure D using **31** (0.234 mmol), **6a** (0.234 mmol), and NaHCO_3_ (0.702 mmol). The reaction was heated at reflux for 18 h. After purification with flash column chromatography (EtOAc, 100%), **37** was obtained (90 mg, 0.176 mmol, 75%). ^1^H NMR: (300 MHz, methanol-*d*_4_) δ 8.25 (s, 1H), 8.18 (s, 1H), 7.26–7.17 (m, 2H), 7.05–6.94 (m, 2H), 6.86 (t, *J* = 7.3, 1H), 6.32 (d, *J* = 1.4, 1H), 5.60 (dd, *J* = 6.1, 2.0, 1H), 5.47 (dd, *J* = 6.0, 1.2, 1H), 4.82–4.68 (m, 2H), 4.63 (d, *J* = 1.9, 1H), 2.97–2.74 (m, 2H), 2.35–2.12 (m, 2H), 1.97–1.64 (m, 4H), 1.57 (s, 3H), 1.39 (s, 3H), 0.62 (t, *J* = 7.3, 3H). ^13^C NMR: (75 MHz, methanol-*d*_4_) δ 170.0, 157.9, 154.3, 152.6, 148.6, 140.6, 129.0, 120.3, 119.3, 115.4, 113.5, 91.0, 87.2, 83.8, 83.7, 82.4, 57.1, 33.4, 30.0, 29.7, 25.8, 24.1, 21.1, 12.8. HRMS: (NSI+) *m/z* calcd for C_26_H_33_N_6_O_5_ [M + H]^+^ 509.2505, found 509.2507.

#### (3a*S*,4*S*,6*R*,6a*R*)-6-(6-(((1*R*,2*R*)-2-(4-(*tert*-Butyl)phenoxy)cyclopentyl)amino)-9*H*-purin-9-yl)-*N*-ethyl-2,2-dimethyltetrahydrofuro[3,4-*d*][1,3]dioxole-4-carboxamide (**38**)

**38** was synthesized according to general procedure D using **31** (0.185 mmol), **6c** (0.185 mmol), and NaHCO_3_ (0.556 mmol). The reaction was heated at reflux for 18 h. After purification with flash column chromatography (EtOAc, 100%), **38** was obtained (58 mg, 0.103 mmol, 56%). ^1^H NMR: (300 MHz, methanol-*d*_4_) δ 8.12 (s, 1H), 8.07 (s, 1H), 7.12 (d, *J* = 8.8, 2H), 6.78 (d, *J* = 8.8, 2H), 6.21 (d, *J* = 1.4, 1H), 5.49 (dd, *J* = 6.1, 2.0, 1H), 5.36 (dd, *J* = 6.1, 1.4, 1H), 4.67–4.54 (m, 2H), 4.51 (d, *J* = 1.9, 1H), 2.82–2.62 (m, 2H), 2.22–2.02 (m, 2H), 1.87–1.51 (m, 4H), 1.46 (s, 3H), 1.28 (s, 3H), 1.14 (s, 9H), 0.50 (t, *J* = 7.3, 3H). ^13^C NMR: (75 MHz, methanol-*d*_4_) δ 170.1, 155.6, 154.3, 152.6, 148.4, 143.1, 140.6, 125.7, 119.3, 114.9, 113.5, 91.0, 87.3, 83.9, 83.71, 82.5, 57.2, 33.5, 33.4, 30.6, 30.1, 29.8, 25.7, 24.0, 21.1, 12.7.

#### (3a*S*,4*S*,6*R*,6a*R*)-*N*-Ethyl-6-(6-(((1*R*,2*R*)-2-(3-methoxyphenoxy)cyclopentyl)amino)-9*H*-purin-9-yl)-2,2-dimethyltetrahydrofuro[3,4-*d*][1,3]dioxole-4-carboxamide (**39**)

**39** was synthesized according to general procedure D using **31** (0.27 mmol), **6e** (0.27 mmol), and NaHCO_3_ (0.738 mmol). The reaction was heated at reflux for 18 h. After purification with flash column chromatography (EtOAc, 100%), **39** was obtained (92 mg, 0.166 mmol, 71%). ^1^H NMR: (300 MHz, methanol-*d*_4_) δ 8.25 (s, 1H), 8.18 (s, 1H), 7.09 (t, *J* = 8.2, 1H), 6.64–6.53 (m, 2H), 6.48–6.41 (m, 1H), 6.32 (d, *J* = 1.4, 1H), 5.60 (dd, *J* = 6.1, 1.9, 1H), 5.48 (dd, *J* = 6.1, 1.4, 1H), 4.80–4.66 (m, 2H), 4.63 (d, *J* = 1.9, 1H), 3.72 (s, 3H), 2.95–2.74 (m, 2H), 2.34–2.11 (m, 2H), 1.98–1.63 (m, 4H), 1.57 (s, 3H), 1.40 (s, 3H), 0.63 (t, *J* = 7.3, 3H). ^13^C NMR: (75 MHz, methanol-*d*_4_) δ 170.0, 160.9, 159.1, 154.3, 152.6, 148.4, 140.6, 129.4, 119.3, 113.5, 107.7, 105.9, 101.7, 91.0, 87.2, 83.8, 83.7, 82.5, 57.3, 54.3, 33.4, 30.0, 29.8, 25.7, 24.0, 21.1, 12.7. HRMS: (NSI+) *m/z* calcd for C_27_H_35_N_6_O_6_ [M + H]^+^ 539.2598, found 539.2613.

#### (3a*S*,4*S*,6*R*,6a*R*)-6-(6-(((1*R*,2*R*)-2-(3-Bromophenoxy)cyclopentyl)amino)-9*H*-purin-9-yl)-*N*-ethyl-2,2-dimethyltetrahydrofuro[3,4-*d*][1,3]dioxole-4-carboxamide (**40**)

**40** was synthesized according to general procedure D using **31** (0.109 mmol), **6f** (0.109 mmol), and NaHCO_3_ (0.218 mmol). The reaction was heated at reflux for 18 h. After purification with flash column chromatography (EtOAc, 100%), **40** was obtained (47 mg, 0.081 mmol, 73%). ^1^H NMR: (300 MHz, methanol-*d*_4_) δ 8.54 (s, 1H), 8.40 (s, 1H), 7.77 (br s, 1H), 7.32 (t, *J* = 8.0, 1H), 7.25–7.12 (m, 2H), 6.53 (d, *J* = 1.4, 1H), 5.81 (dd, *J* = 6.1, 2.0, 1H), 5.68 (dd, *J* = 6.1, 1.4, 1H), 4.99–4.93 (m, 1H), 4.92–4.80 (m, 1H), 4.83 (d, *J* = 1.9, 1H), 3.15–2.94 (m, 2H), 2.53–2.31 (m, 2H), 2.21–1.85 (m, 4H), 1.78 (s, 3H), 1.60 (s, 3H), 0.83 (t, *J* = 7.3, 3H). ^13^C NMR: (75 MHz, methanol-*d*_4_) δ 170.1, 158.9, 154.2, 152.7, 148.5, 140.7, 130.3, 123.3, 122.3, 119.3, 118.4, 114.76, 113.5, 91.1, 87.3, 83.9, 83.7, 82.8, 56.7, 33.4, 29.6, 29.5, 25.7, 24.0, 21.0, 12.7. HRMS: (NSI+) *m/z* calcd for C_26_H_32_BrN_6_O_5_ [M + H]^+^ 587.1595, found 587.1612.

#### (3a*S*,4*S*,6*R*,6a*R*)-6-(6-(((1*R*,2*R*)-2-(2-Chlorophenoxy)cyclopentyl)amino)-9*H*-purin-9-yl)-*N*-ethyl-2,2-dimethyltetrahydrofuro[3,4-*d*][1,3]dioxole-4-carboxamide (**41**)

**41** was synthesized according to general procedure D using **31** (0.282 mmol), **6h** (0.282 mmol), and NaHCO_3_ (0.846 mmol). The reaction was heated at reflux for 19 h. After purification with flash column chromatography (EtOAc, 100%), **41** was obtained (114 mg, 0.21 mmol, 74%). ^1^H NMR: (300 MHz, methanol-*d*_4_) δ 8.25 (s, 1H), 8.19 (s, 1H), 7.52 (t, *J* = 5.8, 1H), 7.35–7.26 (m, 2H), 7.23–7.16 (m, 1H), 6.91–6.81 (m, 1H), 6.33 (d, *J* = 1.4, 1H), 5.60 (dd, *J* = 6.1, 1.9, 1H), 5.48 (dd, *J* = 6.1, 1.4, 1H), 4.86–4.80 (m, 1H), 4.80–4.66 (m, 1H), 4.63 (d, *J* = 1.9, 1H), 2.96–2.75 (m, 2H), 2.40–2.27 (m, 1H), 2.23–2.11 (m, 1H), 2.00–1.83 (m, 3H), 1.80–1.65 (m, 1H), 1.57 (s, 3H), 1.39 (s, 3H), 0.63 (t, *J* = 7.3, 3H). ^13^C NMR: (75 MHz, methanol-*d*_4_) δ 170.0, 154.2, 153.5, 152.6, 148.4, 140.6, 129.8, 127.5, 123.2, 121.3, 119.3, 115.5, 113.5, 91.0, 87.2, 83.8, 83.8, 83.7, 57.0, 33.4, 29.8, 29.7, 25.8, 24.0, 21.1, 12.8. HRMS: (NSI+) *m/z* calcd for C_26_H_32_ClN_6_O_5_ [M + H]^+^ 543.2106, found 543.2117.

#### (3a*S*,4*S*,6*R*,6a*R*)-6-(6-(((1*R*,2*R*)-2-(3-Chlorophenoxy)cyclopentyl)amino)-9*H*-purin-9-yl)-*N*-ethyl-2,2-dimethyltetrahydrofuro[3,4-*d*][1,3]dioxole-4-carboxamide (**42**)

**42** was synthesized according to general procedure D using **31** (0.243 mmol), **6i** (0.243 mmol), and NaHCO_3_ (0.73 mmol). The reaction was heated at reflux for 19 h. After purification with flash column chromatography (EtOAc, 100%), **42** was obtained (101 mg, 0.186 mmol, 78%). ^1^H NMR: (300 MHz, methanol-*d*_4_) δ 8.31 (s, 1H), 8.20 (s, 1H), 7.38 (br s, 1H), 7.17 (t, *J* = 8.1, 1H), 6.99–6.84 (m, 2H), 6.33 (d, *J* = 1.5, 1H), 5.61 (dd, *J* = 6.1, 2.0, 1H), 5.48 (dd, *J* = 6.0, 1.5, 1H), 4.80–4.72 (m, 1H), 4.71–4.59 (m , 2H), 2.97–2.74 (m, 2H), 2.34–2.09 (m, 2H), 2.00–1.65 (m, 4H), 1.58 (s, 3H), 1.40 (s, 3H), 0.64 (t, *J* = 7.3, 3H). ^13^C NMR: (75 MHz, methanol-*d*_4_) δ 170.0, 158.9, 154.2, 152.6, 148.4, 140.6, 134.4, 130.0, 120.3, 119.3, 115.6, 114.2, 113.5, 91.1, 87.2, 83.8, 83.7, 82.8, 56.8, 33.4, 29.6, 29.5, 25.8, 24.0, 21.0, 12.8. HRMS: (NSI+) *m/z* calcd for C_26_H_32_ClN_6_O_5_ [M + H]^+^ 543.2123, found 543.2117.

#### (3a*S*,4*S*,6*R*,6a*R*)-6-(6-(((1*R*,2*R*)-2-(4-Chlorophenoxy)cyclopentyl)amino)-9*H*-purin-9-yl)-*N*-ethyl-2,2-dimethyltetrahydrofuro[3,4-*d*][1,3]dioxole-4-carboxamide (**43**)

**43** was synthesized according to general procedure D using **31** (0.35 mmol), **6j** (0.35 mmol), and NaHCO_3_ (1.052 mmol). The reaction was heated at reflux for 20 h. After purification with flash column chromatography (EtOAc, 100%), **43** was obtained (118 mg, 0.217 mmol, 65%). ^1^H NMR: (300 MHz, methanol-*d*_4_) δ 8.25 (s, 1H), 8.19 (s, 1H), 7.21–7.12 (m, 2H), 7.05–6.97 (m, 2H), 6.32 (d, *J* = 1.4, 1H), 5.60 (dd, *J* = 6.1, 2.0, 1H), 5.47 (dd, *J* = 6.1, 1.5, 1H), 4.77–4.65 (m, 2H), 4.63 (d, *J* = 1.9, 1H), 2.95–2.76 (m, 2H), 2.32–2.11 (m, 2H), 1.97–1.65 (m, 4H), 1.57 (s, 3H), 1.39 (s, 3H), 0.63 (t, *J* = 7.2, 3H). ^13^C NMR: (75 MHz, methanol-*d*_4_) δ 170.0, 156.7, 154.2, 152.6, 148.4, 140.6, 128.8, 125.1, 119.3, 116.9, 113.5, 91.0, 87.2, 83.8, 83.7, 82.8, 57.0, 33.39, 29.8, 29.6, 25.8, 24.1, 21.0, 12.8. HRMS: (NSI+) *m/z* calcd for C_26_H_32_ClN_6_O_5_ [M + H]^+^ 543.2109, found 543.2117.

#### (2*S*,3*S*,4*R*,5*R*)-*N*-Ethyl-3,4-dihydroxy-5-(6-(((1*R*,2*R*)-2-((3-methoxybenzyl)oxy)cyclopentyl)amino)-9*H*-purin-9-yl)tetrahydrofuran-2-carboxamide (**44**)

**44** was synthesized according to general procedure F using **32** (0.075 mmol) and acetic acid (4 mL). The reaction was run for 23 h. After purification with flash column chromatography (MeOH/CH_2_Cl_2_, 6%), **44** was obtained (23 mg, 0.046 mmol, 60%). ^1^H NMR: (300 MHz, methanol-*d*_4_) δ 8.20 (s, 1H), 8.13 (s, 1H), 7.04 (t, *J* = 8.0, 1H), 6.79–6.72 (m, 2H), 6.68–6.62 (m, 1H), 5.90 (d, *J* = 7.7, 1H), 4.65 (dd, *J* = 7.7, 4.8, 1H), 4.62–4.52 (m, 1H), 4.51 (s, 2H), 4.37 (d, *J* = 1.5, 1H), 4.21 (dd, *J* = 4.8, 1.6, 1H), 3.92–3.85 (m, 1H), 3.60 (s, 3H), 3.32–3.23 (m, 2H), 2.20–2.07 (m, 1H), 1.98–1.82 (m, 1H), 1.79–1.46 (m, 4H), 1.10 (t, *J* = 7.3, 3H. ^13^C NMR: (75 MHz, methanol-*d*_4_) δ 170.7, 159.8, 154.4, 152.4, 148.1, 140.6, 140.3, 128.9, 120.1, 119.5, 112.7, 112.6, 89.2, 85.1, 84.8, 73.6, 72.0, 70.8, 58.8, 54.2, 33.7, 30.1, 30.0, 21.1, 13.7. HRMS: (NSI+) *m/z* calcd for C_25_H_33_N_6_O_6_ [M + H]^+^ 513.2441, found 513.2456. UPLC analysis: *^t^R* = 2.59 min; peak area > 99% (detection at 254 nm).

#### (2*S*,3*S*,4*R*,5*R*)-5-(6-(((1*R*,2*R*)-2-((3-Bromobenzyl)oxy)cyclopentyl)amino)-9*H*-purin-9-yl)-*N*-ethyl-3,4-dihydroxytetrahydrofuran-2-carboxamide (**45**)

**45** was synthesized according to general procedure F using **33** (0.196 mmol) and acetic acid (16 mL). The reaction was run for 18 h. After purification with flash column chromatography (MeOH/CH_2_Cl_2_, 2%), **45** was obtained (69 mg, 0.123 mmol, 63%). ^1^H NMR: (300 MHz, methanol-*d*_4_) δ 8.33 (s, 1H), 8.25 (s, 1H), 7.46 (br s, 1H), 7.37–7.07 (m, 3H), 6.02 (d, *J* = 7.7, 1H), 4.77 (dd, *J* = 7.7, 4.8, 1H), 4.71–4.55 (m, 1H), 4.61 (s, 3H), 4.50 (d, *J* = 1.5, 1H), 4.38–4.28 (m, 1H), 3.42–3.28 (m, 2H), 2.29–2.10 (m, 1H), 1.87–1.59 (m, 4H), 1.21 (t, *J* = 7.3, 3H). ^13^C NMR: (75 MHz, methanol-*d*_4_) δ 174.0, 154.4, 152.4, 147.9, 141.5, 140.7, 130.1, 130.0, 129.7, 125.8, 121.9, 120.1, 113.6, 89.2, 85.1, 73.6, 72.1, 70.0, 56.8, 33.7, 30.1, 29.9, 19.5, 13.8. HRMS: (NSI+) *m/z* calcd for C_24_H_30_BrN_6_O_5_ [M + H]^+^ 561.1439, found 561.1456. UPLC analysis: *^t^R* = 2.97 min; peak area > 95% (detection at 254 nm).

#### (2*S*,3*S*,4*R*,5*R*)-5-(6-(((1*R*,2*R*)-2-((4-Bromobenzyl)oxy)cyclopentyl)amino)-9*H*-purin-9-yl)-*N*-ethyl-3,4-dihydroxytetrahydrofuran-2-carboxamide (**46**)

**46** was synthesized according to general procedure F using **34** (0.108 mmol) and acetic acid (8 mL). The reaction was run for 20 h. After purification with flash column chromatography (MeOH/CH_2_Cl_2_, 10%), **46** was obtained (36 mg, 0.064 mmol, 64%). ^1^H NMR: (300 MHz, methanol-*d*_4_) δ 8.19 (s, 1H), 8.14 (s, 1H), 7.28 (d, *J* = 8.4, 2H), 7.10 (d, *J* = 8.3, 2H), 5.90 (d, *J* = 7.7, 1H), 4.65 (dd, *J* = 7.7, 4.8, 1H), 4.62–4.46 (m, 1H), 4.49 (s, 2H), 4.37 (d, *J* = 1.5, 1H), 4.21 (dd, *J* = 4.8, 1.6, 1H), 3.92–3.82 (m, 1H), 3.32–3.21 (m, 2H), 2.20–2.06 (m, 1H), 1.97–1.84 (m, 1H), 1.78–1.58 (m, 3H), 1.58–1.45 (m, 1H), 1.10 (t, *J* = 7.3, 3H). ^13^C NMR: (75 MHz, methanol-*d*_4_) δ 170.7, 154.4, 152.4, 148.1, 140.7, 138.1, 130.9, 129.1, 120.7, 120.1, 89.2, 85.1, 85.0, 73.6, 72.0, 70.1, 56.9, 33.7, 30.1, 30.0, 21.1, 13.7. HRMS: (NSI+) *m/z* calcd for C_24_H_30_BrN_6_O_5_ [M + H]^+^ calculated 561.1458, found 561.1456. UPLC analysis: *^t^R* = 2.94 min; peak area > 99% (detection at 254 nm).

#### (2*S*,3*S*,4*R*,5*R*)-5-(6-(((1*R*,2*R*)-2-((2-Chlorobenzyl)oxy)cyclopentyl)amino)-9*H*-purin-9-yl)-*N*-ethyl-3,4-dihydroxytetrahydrofuran-2-carboxamide (**47**)

**47** was synthesized according to general procedure F using **35** (0.141 mmol) and acetic acid (15 mL). The reaction was run for 19 h. After purification with flash column chromatography (MeOH/CH_2_Cl_2_, 8%), **47** was obtained (33 mg, 0.069 mmol, 49%). ^1^H NMR: (300 MHz, DMSO-*d*_6_) δ 8.90 (t, *J* = 5.6, 1H), 8.42 (s, 1H), 8.29 (s, 1H), 8.07 (br s, 1H), 7.50–7.45 (m, 1H), 7.43–7.38 (m, 1H), 7.33–7.27 (m, 2H), 5.98 (d, *J* = 7.5, 1H), 5.75 (d, *J* = 4.3, 1H), 5.54 (d, *J* = 6.5, 1H), 4.75–4.58 (m, 4H), 4.32 (d, *J* = 1.5, 1H), 4.18–4.12 (m, 1H), 4.10–4.03 (m, 1H), 3.28–3.17 (m, 2H), 2.15–1.94 (m, 2H), 1.84–1.59 (m, 4H), 1.09 (t, *J* = 7.2, 3H). ^13^C NMR (101 MHz, DMSO-*d*_6_) δ 169.6, 154.7, 152.6, 148.8, 141.0, 136.8, 132.3, 129.6, 129.5, 129.4, 127.6, 120.5, 88.3, 85.3, 85.1, 73.6, 72.5, 68.0, 56.9, 33.8, 30.8, 30.7, 22.0, 15.2. HRMS: (NSI+) *m/z* calcd for C_24_H_30_ClN_6_O_5_ [M + H]^+^ calculated 517.1943, found 517.1961. UPLC analysis: *^t^R* = 2.82 min; peak area > 99% (detection at 254 nm).

#### (2*S*,3*S*,4*R*,5*R*)-5-(6-(((1*R*,2*R*)-2-((3-Chlorobenzyl)oxy)cyclopentyl)amino)-9*H*-purin-9-yl)-*N*-ethyl-3,4-dihydroxytetrahydrofuran-2-carboxamide (**48**)

**48** was synthesized according to general procedure F using **36** (0.206 mmol) and acetic acid (16 mL). The reaction was run for 20 h. After purification with flash column chromatography (MeOH/CH_2_Cl_2_, 8%), **48** was obtained (64 mg, 0.123 mmol, 60%). ^1^H NMR: (300 MHz, methanol-*d*_4_) δ 8.32 (s, 1H), 8.24 (s, 1H), 7.32 (br s, 1H), 7.27–7.14 (m, 3H), 6.02 (d, *J* = 7.7, 1H), 4.77 (dd, *J* = 7.7, 4.8, 1H), 4.70–4.60 (m, 1H) 4.62 (s, 2H), 4.50 (d, *J* = 1.5, 1H), 4.34 (dd, *J* = 4.8, 1.6, 1H), 4.01–3.94 (m, 1H) 3.43–3.29 (m, 2H), 2.31–2.15 (m, 1H), 2.07–1.94 (m, 1H), 1.90–1.58 (m, 4H), 1.21 (t, *J* = 7.3, 3H). ^13^C NMR (75 MHz, methanol-*d*_4_) δ 170.6, 154.4, 152.4, 148.0, 141.3, 140.7, 133.7, 129.3, 127.1, 127.0, 125.3, 120.1, 89.2, 85.1, 73.6, 72.0, 70.0, 56.8, 33.7, 30.1, 29.9, 21.1, 13.8. HRMS: (NSI+) *m/z* calcd for C_24_H_30_ClN_6_O_5_ [M + H]^+^ 517.1956, found 517.1961. UPLC analysis: *^t^R* = 2.87 min; peak area > 99% (detection at 254 nm).

#### (2*S*,3*S*,4*R*,5*R*)-*N*-Ethyl-3,4-dihydroxy-5-(6-(((1*R*,2*R*)-2-phenoxycyclopentyl)amino)-9*H*-purin-9-yl)tetrahydrofuran-2-carboxamide (**49**)

**49** was synthesized according to general procedure F using **37** (0.175 mmol) and acetic acid (16 mL). The reaction was run for 23 h. After purification with flash column chromatography (MeOH/CH_2_Cl_2_, 7%), **49** was obtained (56 mg, 0.12 mmol, 68%). ^1^H NMR: (300 MHz, methanol-*d*_4_) δ 8.31 (s, 1H), 8.25 (s, 1H), 7.26–7.16 (m, 2H), 7.05–6.96 (m, 2H), 6.87 (t, *J* = 7.3, 1H), 6.02 (d, *J* = 7.7, 1H), 4.83–4.66 (m, 3H), 4.49 (d, *J* = 1.6, 1H), 4.37–4.30 (m, 1H), 3.42–3.31 (m, 2H), 2.39–2.13 (m, 2H), 2.02–1.65 (m, 4H), 1.20 (t, *J* = 7.3, 3H). ^13^C NMR: (75 MHz, methanol-*d*_4_) δ 170.6, 157.9, 154.5, 152.4, 148.2, 140.8, 129.0, 120.3, 120.2, 115.4, 89.2, 85.1, 82.5, 73.6, 72.0, 57.1, 33.7, 29.9, 29.7, 21.1, 13.7. HRMS: (NSI+) *m/z* calcd for C_23_H_29_N_6_O_5_ [M + H]^+^ 469.2186, found 469.2194. UPLC analysis: *^t^R* = 2.77 min; peak area > 99% (detection at 254 nm).

#### (2*S*,3*S*,4*R*,5*R*)-5-(6-(((1*R*,2*R*)-2-(4-(*tert*-Butyl)phenoxy)cyclopentyl)amino)-9*H*-purin-9-yl)-*N*-ethyl-3,4-dihydroxytetrahydrofuran-2-carboxamide (**50**)

**50** was synthesized according to general procedure F using **38** (0.103 mmol) and acetic acid (8 mL). The reaction was run for 19 h. After purification with flash column chromatography (MeOH/CH_2_Cl_2_, 7%), **50** was obtained (31 mg, 0.059 mmol, 57%). ^1^H NMR (300 MHz, DMSO-*d*_6_) δ 8.88 (t, *J* = 5.6, 1H), 8.43 (s, 1H), 8.30 (s, 1H), 8.17 (d, *J* = 6.5, 1H), 7.26 (d, *J* = 8.8, 2H), 6.87 (d, *J* = 8.3, 2H), 5.98 (d, *J* = 7.5, 1H), 5.75 (d, *J* = 4.3, 1H), 5.54 (d, *J* = 6.5, 1H), 4.88–4.77 (m, 1H), 4.70–4.57 (m, 2H), 4.32 (d, *J* = 1.6, 1H), 4.17–4.12 (m, 1H), 3.27–3.18 (m, 2H), 2.22–1.97 (m, 2H), 1.87–1.63 (m, 4H), 1.23 (s, 9H), 1.09 (t, *J* = 7.2, 3H); (300 MHz, methanol-*d*_4_) δ 8.20 (s, 1H), 8.14 (s, 1H), 7.19–7.10 (m, 2H), 6.85–6.73 (m, 2H), 5.90 (d, *J* = 7.7, 1H), 4.70–4.56 (m, 3H), 4.36 (d, *J* = 1.6, 1H), 4.21 (dd, *J* = 4.9, 1.6, 1H), 3.31–3.21 (m, 2H), 2.24–2.02 (m, 2H), 1.90–1.54 (m, 4H), 1.15 (s, 9H), 1.09 (t, *J* = 7.3, 3H). ^13^C NMR: (75 MHz, methanol-*d*_4_) δ 170.6, 155.6, 154.5, 152.4, 148.1, 143.1, 140.7, 125.7, 120.2, 115.0, 89.2, 85.1, 82.6, 73.6, 72.0, 57.1, 33.7, 33.5, 30.6, 30.0, 29.8, 21.1, 13.7. HRMS: (NSI+) *m/z* calcd for C_27_H_37_N_6_O_5_ [M + H]^+^ 525.2816, found 525.2820. UPLC analysis: *^t^R* = 3.44 min; peak area > 99% (detection at 254 nm).

#### (2*S*,3*S*,4*R*,5*R*)-*N*-Ethyl-3,4-dihydroxy-5-(6-(((1*R*,2*R*)-2-(3-methoxyphenoxy)cyclopentyl)amino)-9*H*-purin-9-yl)tetrahydrofuran-2-carboxamide (**51**)

**51** was synthesized according to general procedure F using **39** (0.231 mmol) and acetic acid (16 mL). The reaction was run for 22 h. After purification with flash column chromatography (MeOH/CH_2_Cl_2_, 8%), **51** was obtained (49 mg, 0.099 mmol, 43%). ^1^H NMR: (300 MHz, DMSO-*d*_6_) δ 8.87 (t, *J* = 5.7, 1H), 8.43 (s, 1H), 8.29 (s, 1H), 8.20 (d, *J* = 7.6, 1H), 7.14 (t, *J* = 8.1, 1H), 6.59–6.43 (m, 3H), 5.98 (d, *J* = 7.5, 1H), 5.75 (d, *J* = 4.3, 1H), 5.54 (d, *J* = 6.4, 1H), 4.93–4.84 (m, 1H), 4.76–4.54 (m, 2H), 4.32 (d, *J* = 1.6, 1H), 4.18–4.13 (m, 1H), 3.28–3.20 (m, 2H), 2.25–2.06 (m, 2H), 1.88–1.60 (m, 4H), 1.09 (t, *J* = 7.2, 3H). ^13^C NMR: (75 MHz, methanol-*d*_4_) δ 170.6, 160.9, 159.1, 154.4, 152.4, 148.1, 140.7, 129.4, 120.2, 107.74, 105.9, 101.7, 89.2, 85.1, 82.6, 73.6, 72.0, 57.1, 54.3, 33.7, 29.9, 29.8, 21.1, 13.7. HRMS: (NSI+) *m/z* calcd for C_24_H_31_N_6_O_6_ [M + H]^+^ 499.2296, found 499.2300. UPLC analysis: *^t^R* = 2.78 min; peak area > 99% (detection at 254 nm).

#### (2*S*,3*S*,4*R*,5*R*)-5-(6-(((1*R*,2*R*)-2-(3-Bromophenoxy)cyclopentyl)amino)-9*H*-purin-9-yl)-*N*-ethyl-3,4-dihydroxytetrahydrofuran-2-carboxamide (**52**)

**52** was synthesized according to general procedure F using **40** (0.07 mmol) and acetic acid (4 mL). The reaction was run for 18 h. After purification with flash column chromatography (MeOH/CH_2_Cl_2_, 8%), **52** was obtained (28 mg, 0.052 mmol, 74%). ^1^H NMR: (300 MHz, methanol-*d*_4_) δ 8.30 (s, 1H), 8.15 (s, 1H), 7.50 (br s, 1H), 7.01 (t, *J* = 8.0, 1H), 6.94–6.82 (m, 2H), 5.90 (d, *J* = 7.7, 1H), 4.70–4.61 (m, 2H), 4.60–4.48 (m, 1H), 4.36 (d, *J* = 1.5, 1H), 4.25–4.18 (m, 1H), 3.30–3.21 (m, 2H), 2.22–1.98 (m, 2H), 1.93–1.80 (m, 2H), 1.80–1.59 (m, 2H), 1.10 (t, *J* = 7.3, 3H). ^13^C NMR: (75 MHz, methanol-*d*_4_) δ 170.6, 158.9, 154.4, 152.4, 148.2, 140.8, 130.3, 123.3, 122.3, 118.4, 114.8, 89.2, 85.1, 82.8, 73.6, 72.1, 56.7, 33.7, 29.6 29.4, 21.0, 13.7. HRMS: (NSI+) *m/z* calcd for C_23_H_28_BrN_6_O_5_ [M + H]^+^ 547.1295, found 547.1299. UPLC analysis: *^t^R* = 3.17 min; peak area > 99% (detection at 254 nm).

#### (2*S*,3*S*,4*R*,5*R*)-5-(6-(((1*R*,2*R*)-2-(2-Chlorophenoxy)cyclopentyl)amino)-9*H*-purin-9-yl)-*N*-ethyl-3,4-dihydroxytetrahydrofuran-2-carboxamide (**53**)

**53** was synthesized according to general procedure F using **41** (0.21 mmol) and acetic acid (16 mL). The reaction was run for 19 h. After purification with flash column chromatography (MeOH/CH_2_Cl_2_, 8%), **53** was obtained (55 mg, 0.109 mmol, 52%). ^1^H NMR: (300 MHz, DMSO-*d*_6_) δ 8.87 (t, *J* = 5.6, 1H), 8.43 (s, 1H), 8.30 (s, 1H), 8.20 (br s, 1H), 7.42–7.24 (m, 3H), 6.97–6.87 (m, 1H), 5.98 (d, *J* = 7.5, 1H), 5.75 (d, *J* = 4.3, 1H), 5.54 (d, *J* = 6.4, 1H), 4.97–4.90 (m, 1H), 4.77–4.66 (m, 1H), 4.66–4.57 (m, 1H), 4.32 (d, *J* = 1.6, 1H), 4.18–4.12 (m, 1H), 3.29–3.19 (m, 2H), 2.24–2.14 (m, 2H), 1.91–1.67 (m, 4H), 1.09 (t, *J* = 7.2, 3H). ^13^C NMR (75 MHz, methanol-*d*_4_) δ 170.6, 154.4, 153.4, 152.4, 148.2, 140.8, 129.8, 127.4, 123.2, 121.2, 120.2, 115.5, 89.2, 85.1, 83.8, 73.6, 72.0, 56.9, 33.71, 29.7, 29.6, 21.1, 13.7. HRMS: (NSI+) *m/z* calcd for C_23_H_28_ClN_6_O_5_ [M + H]^+^ 503.1810, found 503.1804. UPLC analysis: *^t^R* = 3.00 min; peak area > 95% (detection at 254 nm).

#### (2*S*,3*S*,4*R*,5*R*)-5-(6-(((1*R*,2*R*)-2-(3-Chlorophenoxy)cyclopentyl)amino)-9*H*-purin-9-yl)-*N*-ethyl-3,4-dihydroxytetrahydrofuran-2-carboxamide (**54**)

**54** was synthesized according to general procedure F using **42** (0.184 mmol) and acetic acid (16 mL). The reaction was run for 20 h. After purification with flash column chromatography (MeOH/CH_2_Cl_2_, 8%), **54** was obtained (68 mg, 0.135 mmol, 73%). ^1^H NMR: (300 MHz, DMSO-*d*_6_) δ 8.86 (t, *J* = 5.6, 1H), 8.44 (s, 1H), 8.33 (s, 1H), 8.19 (d, *J* = 6.4, 1H), 7.32–7.23 (m, 2H), 6.99–6.92 (m, 2H), 5.98 (d, *J* = 7.5, 1H), 5.75 (d, *J* = 4.3, 1H), 5.54 (d, *J* = 6.4, 1H), 4.94–4.87 (m, 1H), 4.70–4.57 (m, 2H), 4.32 (d, *J* = 1.6, 1H), 4.18–4.13 (m, 1H), 3.29–3.17 (m, 2H), 2.23–2.08 (m, 2H), 1.90–1.64 (m, 4H), 1.09 (t, *J* = 7.2, 3H). ^13^C NMR (75 MHz, methanol-*d*_4_) δ 170.6, 158.8, 154.3, 152.3, 148.2, 140.8, 134.4, 129.9, 120.3, 120.1, 115.6, 114.3, 89.2, 85.1, 82.8, 73.6, 72.1, 56.7, 33.7, 29.6, 29.4, 21.0, 13.7. HRMS: (NSI+) *m/z* calcd for C_23_H_28_ClN_6_O_5_ [M + H]^+^ 503.1780, found 503.1804. UPLC analysis: *^t^R* = 3.10 min; peak area > 95% (detection at 254 nm).

#### (2*S*,3*S*,4*R*,5*R*)-5-(6-(((1*R*,2*R*)-2-(4-Chlorophenoxy)cyclopentyl)amino)-9*H*-purin-9-yl)-*N*-ethyl-3,4-dihydroxytetrahydrofuran-2-carboxamide (**55**)

**55** was synthesized according to general procedure F using **43** (0.217 mmol) and acetic acid (8 mL). The reaction was run for 23 h. After purification with flash column chromatography (MeOH/CH_2_Cl_2_, 7%), **55** was obtained (74 mg, 0.146 mmol, 67%). ^1^H NMR: (300 MHz, DMSO-*d*_6_) δ 8.87 (t, *J* = 5.7, 1H), 8.43 (s, 1H), 8.32 (s, 1H), 8.19 (d, *J* = 6.5, 1H), 7.34–7.27 (m, 2H), 7.04 (d, *J* = 8.1, 2H), 5.98 (d, *J* = 7.5, 1H), 5.75 (d, *J* = 4.3, 1H), 5.54 (d, *J* = 6.4, 1H), 4.90–4.80 (m, 1H), 4.76–4.58 (m, 2H), 4.32 (d, *J* = 1.6, 1H), 4.18–4.12 (m, 1H), 3.28–3.17 (m, 2H), 2.26–2.10 (m, 2H), 1.91–1.57 (m, 4H), 1.09 (t, *J* = 7.2, 3H). ^13^C NMR: (75 MHz, methanol-*d*_4_) δ 170.6, 156.6, 154.4, 152.4, 148.2, 140.8, 128.8, 125.1, 120.1, 116.8, 89.2, 85.1, 82.8, 73.6, 72.1, 56.9, 33.7, 29.7, 29.6, 21.0, 13.7. HRMS: (NSI+) *m/z* calcd for C_23_H_28_ClN_6_O_5_ [M + H]^+^ 503.1789, found 503.1804. UPLC analysis: *^t^R* = 3.10 min; peak area > 99% (detection at 254 nm).

### Cell Culture

CHO-K1-hA_1_R, CHO-K1-hA_2A_R, CHO-K1-hA_2B_R, and CHO-K1-hA_3_R cells were routinely cultured in Ham’s F-12 supplemented with 10% fetal bovine serum (FBS). HEK293 human Nluc-A_1_R and HEK293 rat Nluc-A_1_R were cultured in Dulbecco’s modified Eagle’s medium (DMEM)/Nutrient Mixture F12 supplemented with 10% FBS. All cells were maintained at 37 °C with 5% CO_2_ in humidified air.

### cAMP Accumulation Assay

cAMP accumulation experiments were performed using a LANCE Ultra cAMP detection kit as described previously.^25,40^ Briefly, CHO-K1 cells stably expressing human WT A_1_R, A_2A_R, A_2B_R, and A_3_R were seeded at 2000 cells per well in a white 384-well OptiPlate. Cells were then incubated with adenosine receptor ligands (ranging between 100 μM and 1 pM) for 30 min at room temperature. For A_1_R and A_3_R expressing cells, 10 and 1 μM forskolin, respectively, was added at the same time as the addition of the adenosine receptor ligands, as we have described previously.^13,24,25^

### NanoBRET Assay for Binding

To determine the affinity (p*K*_i_) of adenosine receptor ligands, a NanoBRET competition binding assay was performed as described previously.^24,25^ Briefly, CA200645 was used at 20 nM. Kinetic data were fitted with the ″kinetic of competitive binding″ model^35^ (built into Prism v9.1 (GraphPad Software, San Diego, CA)) to determine affinity (p*K*_i_) values and the association rate constant (*k*_on_) and dissociation rates (*k*_off_) for AR ligands. In agreement with our previous studies, we determined the *K*_d_ of CA200645 to be 18.29 ± 2.4 nM at the human A_1_R and 32.96 ± 2.8 nM at the rat A_1_R.^25,40^ The ″one-site–*K*_i_ model″ derived from the Cheng and Prusoff correction and available in Prism was fitted with the BRET ratio at 10 min post simulation, and affinity (p*K*_i_) constant values at equilibrium for adenosine receptor ligands were determined.

### Data and Statistical Analysis

Data were analyzed using Prism v9.1 (GraphPad Software, San Diego, CA). Dose–response curves were fitted using a three-parameter logistic equation to calculate response range and pEC_50_, and normalized to forskolin stimulation (A_2A_R and A_2B_R) or forskolin inhibition (A_1_R and A_3_R), expressed as percentage of 100 μM forskolin. Adenosine and NECA stimulations were used as intrinsic controls across all experiments.

Receptor binding kinetics was determined as described previously^25^ using the Motulsky and Mahan method^35^ (built into Prism v9.1) to determine the test compound association rate constant and dissociation rate constant. The *k*_on_ and *k*_off_ values for binding of CA200645 were determined to be *k*_on_ = 3.67 ± 0.34 × 10^6^ M^–1^ min^–1^ and *k*_off_ = 0.064 ± 0.0023 min^–1^ at the human A_1_R and *k*_on_ = 2.93 ± 0.24 × 10^6^ M^–1^ min^–1^ and *k*_off_ = 0.066 ± 0.0022 min^–1^ at the rat A_1_R.

To calculate the relative activities (RA) of compounds (Figure 2), eq 1 was used:
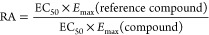
1where *E*_max_ is the maximal response and EC_50_ is the agonist concentration required to produce a half-maximal response, and web plot was plotted using Microsoft Excel. Since the receptors are expressed in the same cell background and adenosine and NECA are full potent agonists across all the adenosine subtypes, we reasoned that changes in log(RA) for a given ligand, relative to NECA or adenosine at A_1_R, would provide a quantitative means of comparing receptor selectivity of individual adenosine receptor ligands.

The statistical analysis was performed in Prism v9.1 using one-way ANOVA with a Dunnett’s post-test for multiple comparisons following the guidelines as described by Curtis *et al*.^31^ All experiments were performed in a minimum of three repeats conducted in duplicate, and data were reported as mean ± SEM.

### Adenosine Receptor Structures

The active state A_1_R coordinates were retrieved from Protein Data Bank (PDB) database^41^ entry 7LD4.^19^ PDB ID 5G53(42) was used for A_2A_R in active conformation. A_2B_R in active conformation was modeled using 5G53 as a template through Modeller 9.19.^43^ The A_2B_R ECL2 (L142^ECL2^-K170^ECL2^) was retrieved from the inactive state model by AlphaFold2^44^ (entry P29275) and inserted in the homology model by superposition. A_3_R in active conformation was modeled using 7LD4 as a template through Modeller 9.19.^43^ The A_3_R ECL2 (K152^ECL2^-S165^ECL2^) was retrieved from the inactive state model by AlphaFold2 (entry P0DMS8) and inserted in the homology model by superposition. All the ARs structures did not present either the ICL3 or the G protein bound to its intracellular binding site.

### Force Field and Ligand Parameters for MD Simulations

The CHARMM36^45,46^/CGenFF 3.0.1^47−49^ force field combination was employed in this work. The force field, topology, and parameter files for **20** and **27** were obtained from the ParamChem webserver.^47^

### System Preparations for MD Simulations

For all systems, hydrogen atoms were added by means of the pdb2pqr^50^ and propka^51^ software (considering a simulated pH of 7.0). The protonation of titratable side chains was checked by visual inspection. The resulting receptors were separately inserted in a square 90 × 90 Å 1-palmitoyl-2-oleyl-*sn*-glycerol-3-phosphocholine (POPC) bilayer (previously built by using the VMD Membrane Builder plugin 1.1, Membrane Plugin, Version 1.1. at http://www.ks.uiuc.edu/Research/vmd/plugins/membrane/) through an insertion method,^52^ along with their co-crystallized ligand (and the crystallographic water molecules within 5 Å of the ligand). The receptor orientation was obtained by superposing the coordinates on the corresponding structure retrieved from the OPM database.^53^ Lipids overlapping the receptor transmembrane helical bundle were removed, and TIP3P water molecules^54^ were added to the simulation box by means of the VMD Solvate plugin 1.5 (Solvate Plugin, Version 1.5. at http://www.ks.uiuc.edu/Research/vmd/plugins/solvate/). Finally, overall charge neutrality was reached by adding Na^+^/Cl^–^ counterions up to the final concentration of 0.150 M using the VMD Autoionize plugin 1.3 (Autoionize Plugin, Version 1.3. at http://www.ks.uiuc.edu/Research/vmd/plugins/autoionize/).

### System Equilibration and MD Settings

The MD engine ACEMD^55^ was employed for both the equilibration and productive simulations. The equilibration was achieved in isothermal–isobaric conditions (NPT) using a Berendsen barostat^56^ (target pressure 1 atm) and Langevin thermostat^57^ (target temperature 300 K) with low damping of 1 ps^–1^. A multistage procedure was performed (integration time step of 2 fs): first, clashes between protein and lipid atoms were reduced through 1500 conjugate-gradient minimization steps, and then 1 kcal mol^–1^ Å^–2^ positional restraints on lipid phosphorus atoms, protein atoms other than Cα, and protein Cα atoms were gradually removed over 2, 60, and 80 ns, respectively. The last 20 ns of equilibration was performed without any positional restraints. Productive trajectories were computed with an integration time step of 4 fs in the canonical ensemble (NVT). The target temperature was set at 300 K using a thermostat damping of 0.1 ps^–1^. The M-SHAKE algorithm^58,59^ was employed to constrain the bond lengths involving hydrogen atoms. The cutoff distance for electrostatic interactions was set at 9 Å, with a switching function applied beyond 7.5 Å. Long-range Coulomb interactions were handled using the particle mesh Ewald summation method (PME)^60^ by setting the mesh spacing to 1.0 Å.

### Molecular Docking

A first attempt to dock **20** and **27** into all the four ARs subtypes was performed on structures prepared as reported above using Vina^61^ in a 30 × 30 × 30 Å cube centered on the atom CZ of the ECL2 conserved phenylalanine residue (F171 in A_1_R, F168 in A_2A_R, F173 in A_2B_R, and F168 in A_3_R). Successive molecular docking simulations of **20** and **27**, with the same settings, were performed into the AR structure extracted from MD simulations (see below). A_1_R, A_2A_R, and A_2B_R were extracted from adiabatic MD simulations, while the A_3_R structure was the equilibrated apo structure.

### Adiabatic MD of the apo Adenosine Receptors

The apo AR structures were prepared and equilibrated as reported above. A_1_R, A_2A_R, and A_2B_R were subjected to 50 ns of adiabatic MD.^62^ A target distance of 13 Å and a force constant of 100 kJ mol^–1^ Å^–2^ were set to favor the opening of the salt bridge between ECL2 and ECL3: E172^ECL2^(Cδ)-K265^ECL3^(Nζ) on A_1_R, E169^ECL2^(Cδ)-H264(Hε2) on A_2A_R, and E174(Cδ)-K265(Nζ) and K267(Nζ) on A_2B_R.

### MD Analysis

Root mean square deviations (RMSD) and fluctuation (RMSF) were computed using VMD.^63^ Interatomic contacts and hydrogen bonds were detected using the GetContacts scripts tool (https://getcontacts.github.io). Contacts and hydrogen bond persistency were quantified as the percentage of frames (over all the frames obtained by merging the different replicas) in which protein residues formed contacts or hydrogen bonds with the ligand. Structural water molecules were detected in the apo ARs using AquaMMapS.^64^ Short 10 ns simulations were performed with a time step 2 fs, restraining the Cα atoms and saving a frame every 20 ps of simulation.

### Residue Numbering System

Throughout the manuscript, the Ballesteros–Weinstein residue numbering system for GPCRs is adopted.^65^

## Supporting Information Available

Summary of *O*-alkylation studies (Scheme S1); synthetic confirmation of 2-aminocyclopentanol stereochemistry (Figure S1); molecular docking of **20** and **27** (Figures S2–S3); molecular dynamics simulations (Figures S4 and S6); mutagenesis study (Figure S5); purity assessment for all tested compounds (Tables S1 and S2); molecular formula strings of all final compounds (Table S3); and reproduction of ^1^H and ^13^C NMR spectra for all tested compounds (PDF)
MD simulations of **27** (orange stick representation) in complex with the four AR subtypes (MP4)
MD simulations of **27** and **20** in complex with A_1_R (white transparent ribbon) during three MD simulations of 2 μs each (MP4)
Coordinates of **27** bound to A_1_R (PDB)
Molecular formula strings of all final compounds (CSV)

## Supplementary Material

jm2c01414_si_003.mp4

jm2c01414_si_004.pdb

## Abbreviations Used

AC: adenylyl cyclase
AR: adenosine receptor
A_1_R: adenosine A_1_ receptor
A_2A_R: adenosine A_2A_ receptor
A_2B_R: adenosine A_2B_ receptor
A_3_R: adenosine A_3_ receptor
BRET: bioluminescence resonance energy transfer
DAIB: (diacetoxyiodo)benzene
DIAD: diisopropyl azodicarboxylate
NECA: 5′-*N*-ethylcarboxamidoadenosine
RA: relative activity
RT: residence time
TEMPO: (2,2,6,6-tetramethylpiperidin-1-yl)oxyl
