# Supplementary material for: Discovery and Structure–Activity Relationship Studies of Novel Adenosine A1 Receptor-Selective Agonists
Source: J Med Chem. 2022 Oct 21;65(21):14864–90. doi: 10.1021/acs.jmedchem.2c01414 (PMC9661479; doi:10.1021/acs.jmedchem.2c01414)
Supplement: Supplementary file 1 — jm2c01414_si_001.pdf [file jm2c01414_si_001.pdf]

SUPPORTING INFORMATION

for

Discovery and Structure-Activity Relationship Studies of Novel Adenosine A_1 Receptor-Selective Agonists

Barbara Preti,[†,#] Anna Suchankova,[‡,#] Giuseppe Deganutti,[§,#] Michele Leuenberger,[†] Kerry Barkan,[‡] Iga Manulak,[‡] Xianglin Huang,[‡] Sabrina Carvalho,[‡] Graham Ladds,[‡,*] and Martin Lochner[†,*]

[†]*Institute of Biochemistry and Molecular Medicine, University of Bern, Bühlstrasse 28, 3012 Bern, Switzerland.*

[‡]*Department of Pharmacology, University of Cambridge, Tennis Court Road, Cambridge CB2 1PD, UK.*

[§]*Centre for Sport, Exercise and Life Sciences, Faculty of Health and Life Sciences, Coventry University, Coventry CV1 5FB, UK.*

*Corresponding authors: grl30@cam.ac.uk and martin.lochner@ibmm.unibe.ch

---

Table of Contents

1. *O*-Alkylation Studies (Scheme S1) S2
2. Synthetic Confirmation of 2-Aminocyclopentanol Stereochemistry (Figure S1) S3
3. Degree of Purity (Tables S1 and S2) and HPLC Traces for Tested Compounds S4-S8
4. Molecular Formula Strings (Table S3) S9
5. Molecular Dynamics Simulations (Movies S1 and S2, Figures S2-S4) S10-S13
6. Validation of Molecular Dynamics Simulations (Figures S5 and S6) S14-S15
7. ^{1}H and ^{13}C NMR Spectra for Final Compounds S16-S42

---

1. O-*Alkylation Studies*

Scheme S1. *O*-Alkylation Studies with Protected Adenosine and NECA Precursors

i.

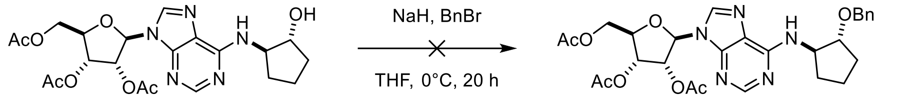

isolated side products apart from unreacted starting material:

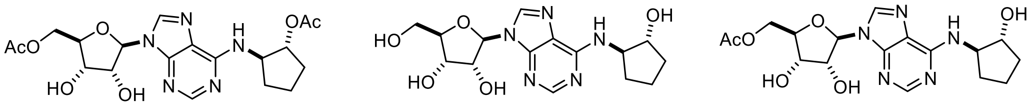

6% | 37% | amount not determined

ii.

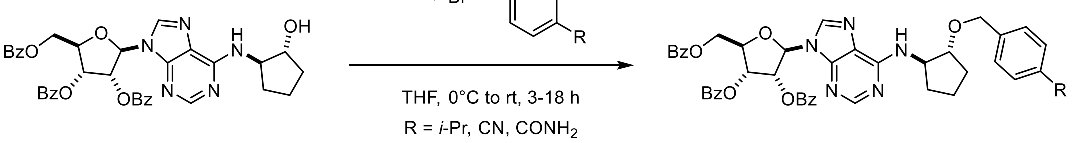

R = *i*-Pr: 8% (low purity)
R = CN: 0%
R = CONH$_2$: 0%

isolated side products:

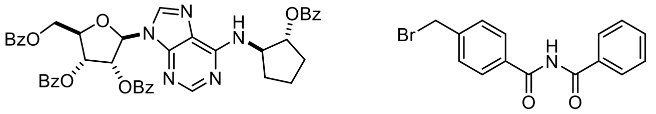

(if R = CONH$_2$)

iii.

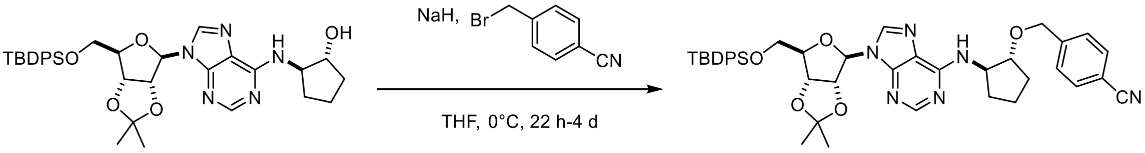

TBDPS = *t*-BuPh$_2$Si

6-34% (low purity)

iv.

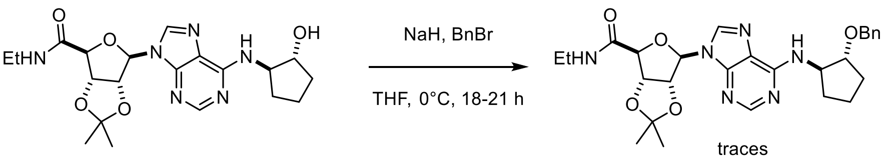

traces

isolated side products apart from unreacted starting material:

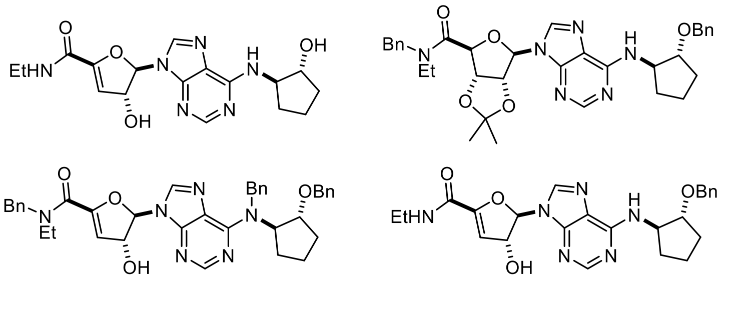

2. *Synthetic Confirmation of 2-Aminocyclopentanol Stereochemistry*

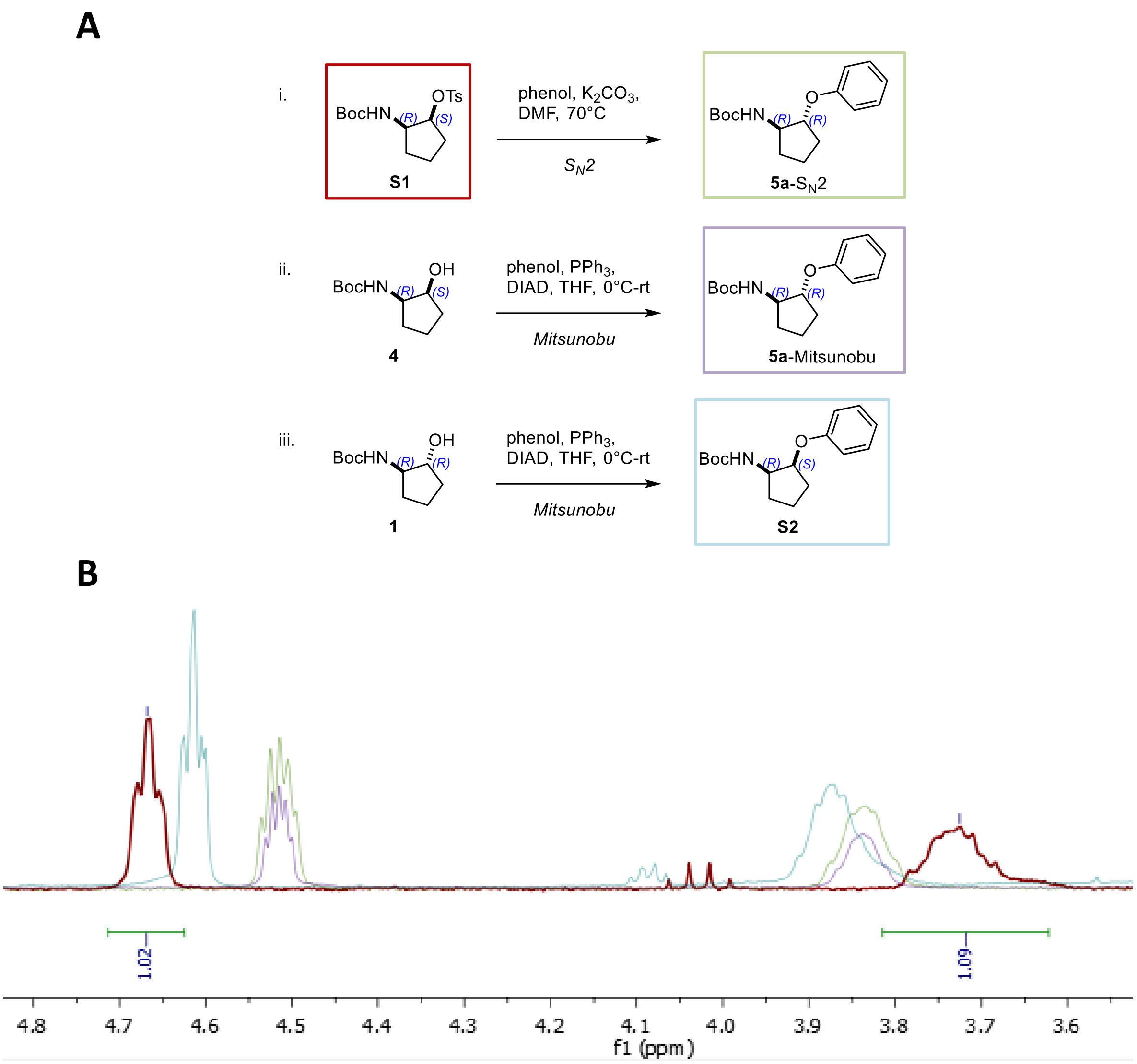

Figure S1. (**A**) Synthesis of *N*-protected phenoxycyclopentylamine **5a** via S_N2 (i.) or Mitsunobu (ii.) reaction and (**B**) comparison of the ^1H NMR spectra of the isolated products. The region of the ^1H NMR spectrum for the H-C(1) (4.5–4.7 ppm) and H-C(2) (3.7–3 .9 ppm) protons is shown. ^1H NMR Spectra of isolated **5a**-S_N2 (*green*) and **5a**-Mitsunobu (*purple*) overlap very well. Mitsunobu reaction starting from epimeric 2-aminocyclopentanol **1** (**A**.iii.) generated C-1 epimer **S2** that displayed a distinctively different ^1H NMR spectrum (*light blue*). ^1H NMR spectrum of tosylated starting material **S1** in *dark red*.

3. *Degree of Purity and HPLC Traces for Tested Compounds*

Table S1. Degree of HPLC Purity for Tested Adenosine Compounds

**Compound Number**	**Purity (%)** Total absorbance at 254 nm	**^{t}R (min)**
**15**	>95	2.87
**16**	>95	3.01
**17**	>95	2.28
**18**	>95	1.79
**19**	>95	2.33
**20**	>95	2.58
**21**	>99	2.64
**22**	>99	2.51
**23**	>99	2.56
**24**	95	7.22[a]
**25**	>99	3.21
**26**	>95	2.50
**27**	>95	2.83
**28**	>99	2.72
**29**	94	7.16[a]
**30**	>99	2.81

[a]Measured on a Thermo-Scientific UltiMate 3000 HPLC equipped with an Acclaim™ 120 C18 5 µm 120Å (4.6 x 150 mm) column. Compounds were eluted with H_2O + 0.1% TFA and MeCN + 0.1% TFA.

Representative HPLC traces:

Compound 18

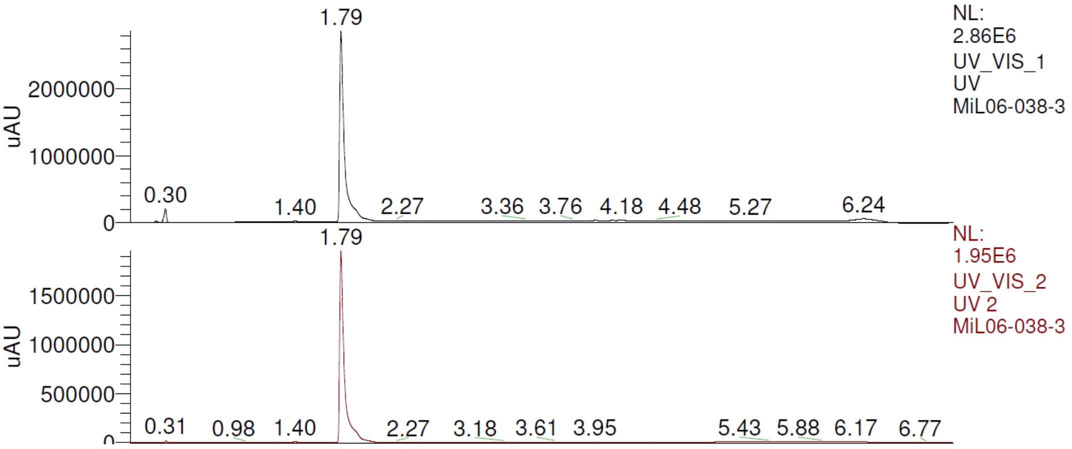

Compound 21

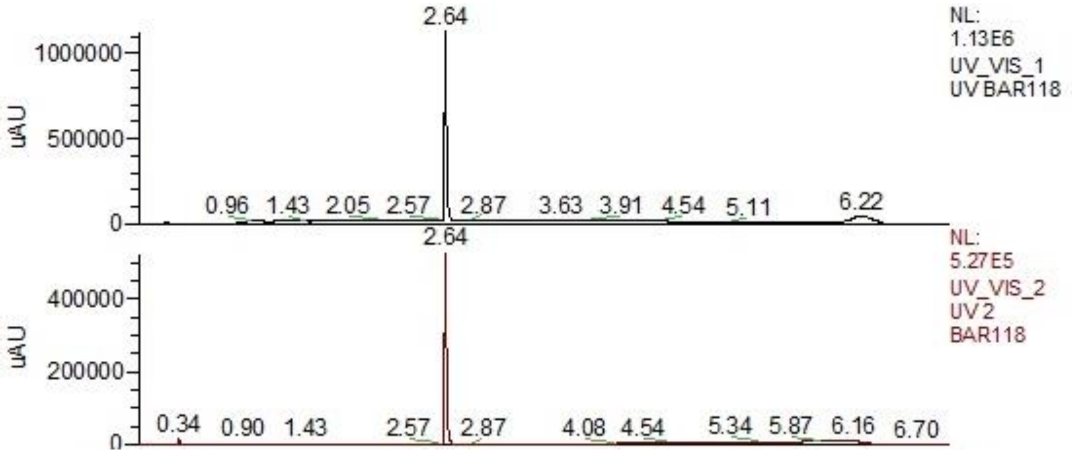

Compound 24 (at 214 nm)

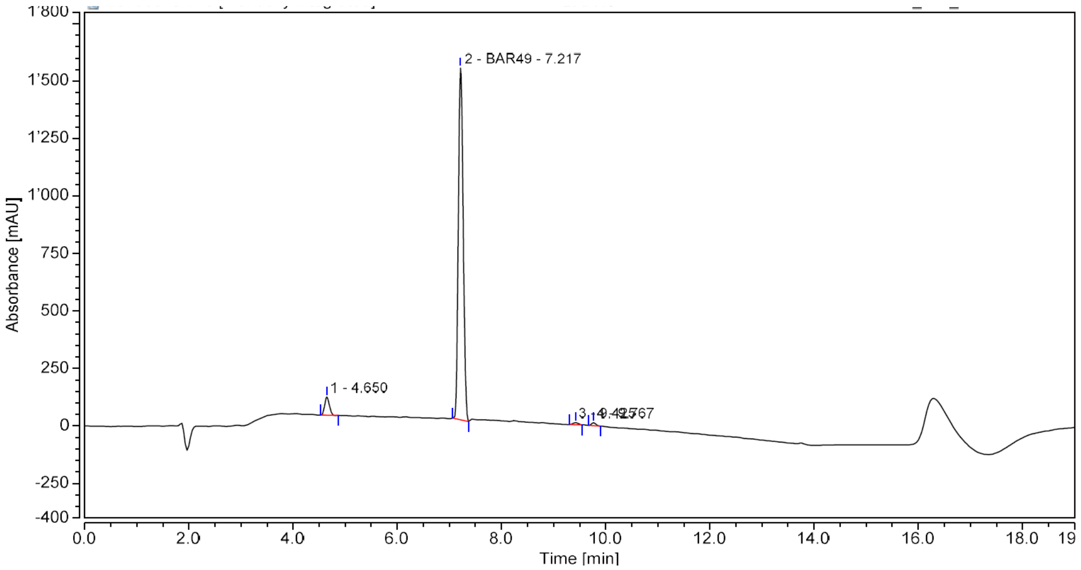

Compound 24 (at 254 nm)

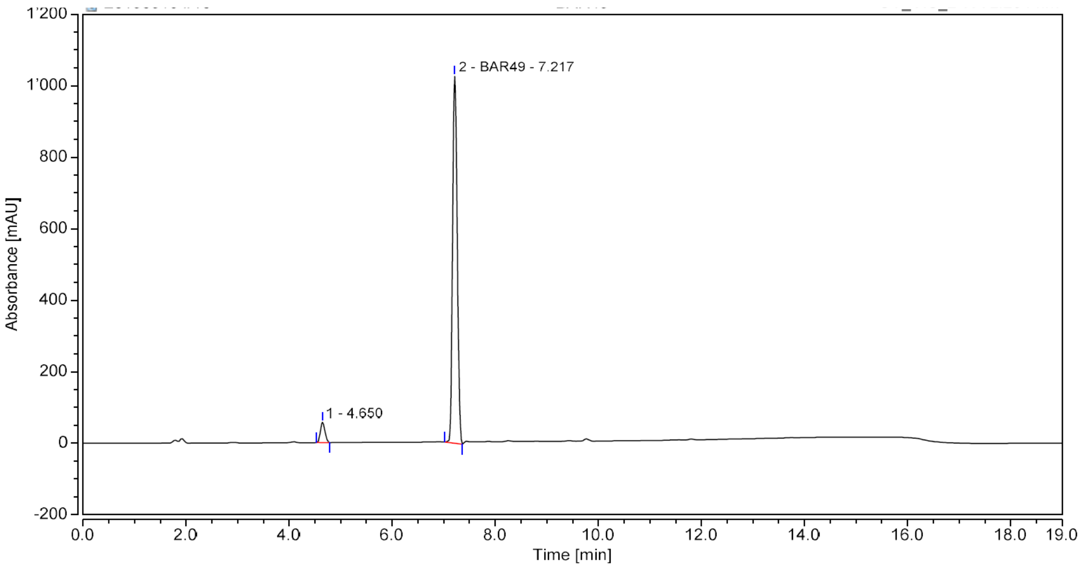

Compound 27

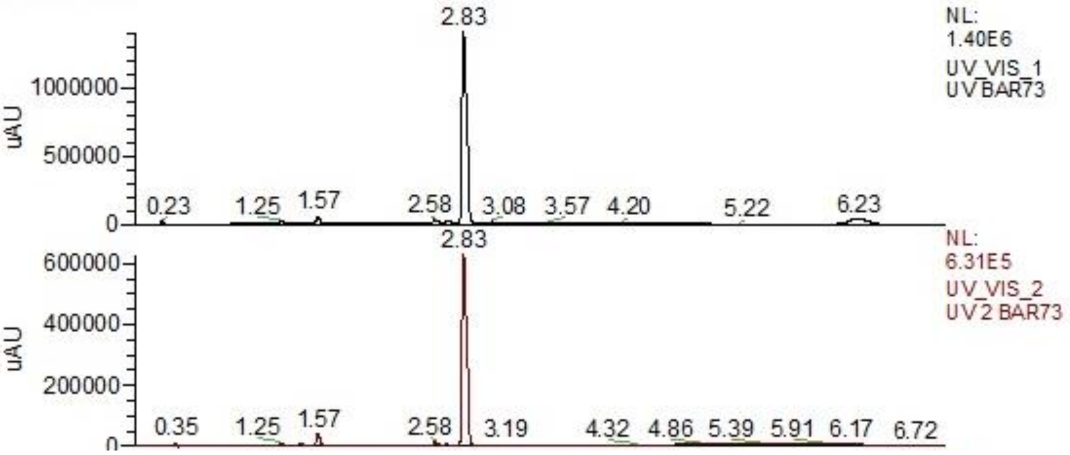

Compound 30

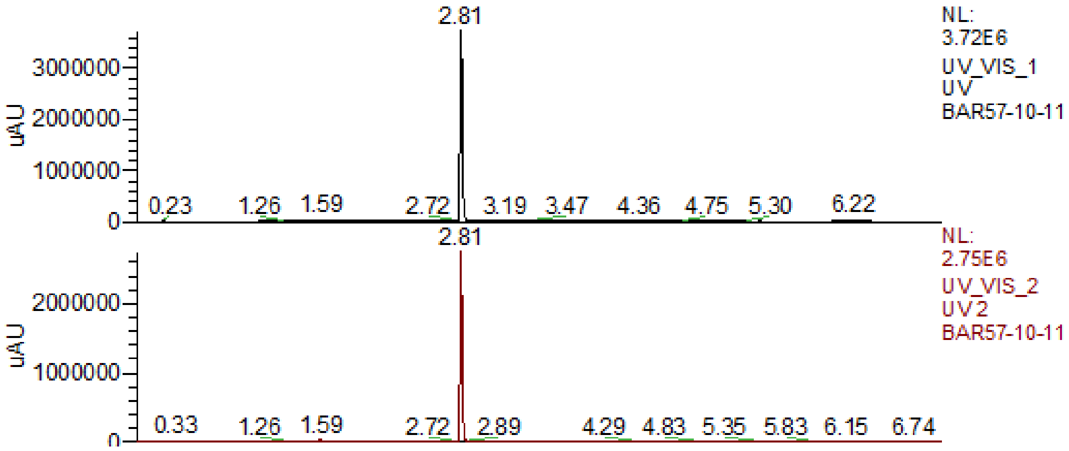

Table S2. Degree of HPLC Purity for Tested NECA Compounds

Compound Number	Purity (%) Total absorbance at 254 nm	^{t}R (min)
**44**	>99	2.59
**45**	>95	2.97
**46**	>99	2.94
**47**	>99	2.82
**48**	>99	2.87
**49**	>99	2.77
**50**	>99	3.44
**51**	>99	2.78
**52**	>99	3.17
**53**	>95	3.00
**54**	>95	3.10
**55**	>99	3.10

Representative HPLC traces:

Compound 46

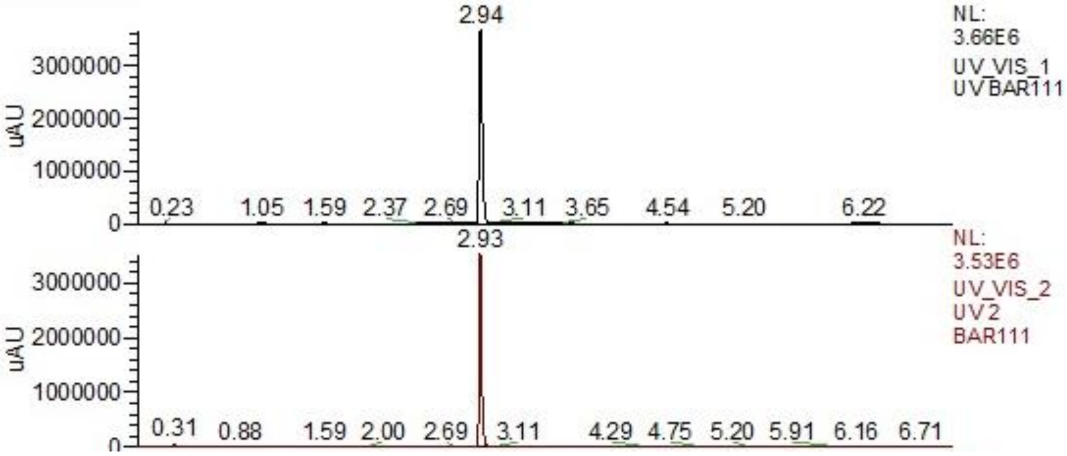

Compound 48

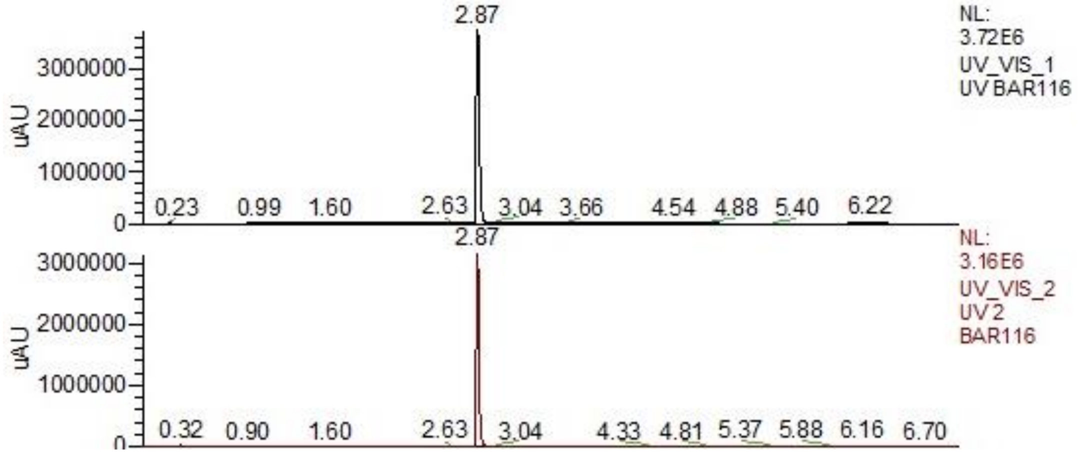

Compound 50

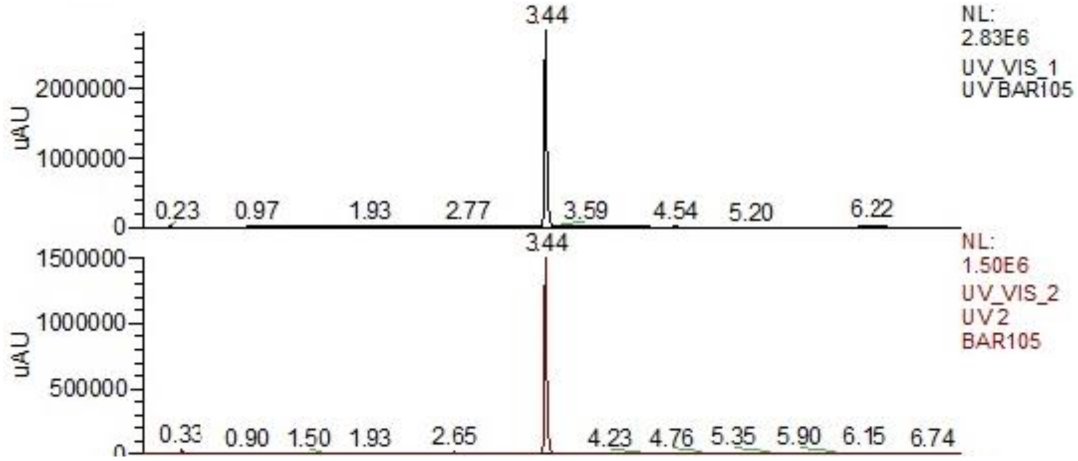

Compound 53

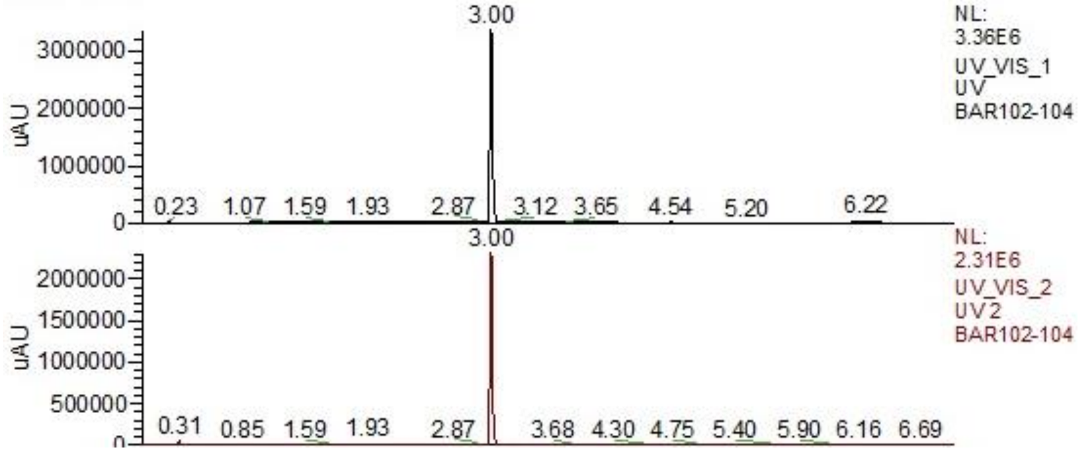

4. *Molecular Formula Strings*

Table S3. Compound SMILES and In Vitro Data

Compound	SMILE	$hA_1R\ pEC_{50}$	$hA_{2A}R\ pEC_{50}$	$hA_{2B}R\ pEC_{50}$	$hA_3R\ pEC_{50}$	$hA_1R\ pK_i$	$rA_1R\ pK_i$
adenosine	NC1=C(N=CN2[C@@H]3O[C@H](CO)[C@@H](O)[C@H]3O)C2=NC=N1	7.16 ± 0.23	7.60 ± 0.11	7.28 ± 0.12	7.87 ± 0.23	6.09 ± 0.06	6.06 ± 0.05
NECA	NC1=C(N=CN2[C@@H]3O[C@H](C(NCC)=O)[C@@H](O)[C@H]3O)C2=NC=N1	8.96 ± 0.11	7.95 ± 0.26	7.20 ± 0.07	7.83 ± 0.26	6.61 ± 0.06	6.38 ± 0.04
BnOCPA	OC[C@H]([C@@H](O)[C@H]1O)O[C@H]1N2C=NC3=C(N[C@@H]4CCC[C@H]4OCC5=CC=CC=C5)N=CN=C32	8.43 ± 0.09	4.95 ± 0.38	n.d.	n.r.	6.18 ± 0.09	6.41 ± 0.06
**15**	OC[C@H]([C@@H](O)[C@H]1O)O[C@H]1N2C=NC3=C(N[C@@H]4CCC[C@H]4OCC5=CC=C(C(C)C)C=C5)N=CN=C32	7.87 ± 0.16	n.a.	n.a.	n.a.	5.77 ± 0.08	n.a.
**16**	OC[C@H]([C@@H](O)[C@H]1O)O[C@H]1N2C=NC3=C(N[C@@H]4CCC[C@H]4OCC5=CC=C(C(C)(C)C)C=C5)N=CN=C32	8.20 ± 0.13	n.a.	n.a.	n.a.	5.58 ± 0.10	n.a.
**17**	OC[C@H]([C@@H](O)[C@H]1O)O[C@H]1N2C=NC3=C(N[C@@H]4CCC[C@H]4OCC5=CC=C(C#N)C=C5)N=CN=C32	7.40 ± 0.19	n.a.	n.a.	n.a.	5.85 ± 0.06	n.a.
**18**	OC[C@H]([C@@H](O)[C@H]1O)O[C@H]1N2C=NC3=C(N[C@@H]4CCC[C@H]4OCC5=CC=C(C(N)=O)C=C5)N=CN=C32	7.71 ± 0.13	n.a.	n.a.	n.a.	5.98 ± 0.05	n.a.
**19**	OC[C@H]([C@@H](O)[C@H]1O)O[C@H]1N2C=NC3=C(N[C@@H]4CCC[C@H]4OCC5=CC=CC(OC)=C5)N=CN=C32	8.74 ± 0.10	n.d.	n.d.	5.98 ± 0.47	6.67 ± 0.10	6.55 ± 0.06
**20**	OC[C@H]([C@@H](O)[C@H]1O)O[C@H]1N2C=NC3=C(N[C@@H]4CCC[C@H]4OCC5=CC=CC(Br)=C5)N=CN=C32	7.74 ± 0.13	n.r.	n.d.	5.08 ± 0.26	6.16 ± 0.10	6.13 ± 0.08
**21**	OC[C@H]([C@@H](O)[C@H]1O)O[C@H]1N2C=NC3=C(N[C@@H]4CCC[C@H]4OCC5=CC=C(Br)C=C5)N=CN=C32	7.39 ± 0.16	n.d.	n.d.	n.r.	5.94 ± 0.07	6.06 ± 0.07
**22**	OC[C@H]([C@@H](O)[C@H]1O)O[C@H]1N2C=NC3=C(N[C@@H]4CCC[C@H]4OCC5=CC=CC=C5Cl)N=CN=C32	8.47 ± 0.24	5.17 ± 0.33	4.57 ± 0.12	n.r.	6.56 ± 0.07	6.56 ± 0.04
**23**	OC[C@H]([C@@H](O)[C@H]1O)O[C@H]1N2C=NC3=C(N[C@@H]4CCC[C@H]4OCC5=CC=CC(Cl)=C5)N=CN=C32	7.88 ± 0.17	n.d.	n.d.	n.r.	6.15 ± 0.07	6.43 ± 0.03
**24**	OC[C@H]([C@@H](O)[C@H]1O)O[C@H]1N2C=NC3=C(N[C@@H]4CCC[C@H]4OC5=CC=CC=C5)N=CN=C32	8.98 ± 0.14	n.d.	4.90 ± 0.14	5.78 ± 0.42	6.84 ± 0.06	6.60 ± 0.02
**25**	OC[C@H]([C@@H](O)[C@H]1O)O[C@H]1N2C=NC3=C(N[C@@H]4CCC[C@H]4OC5=CC=C(C(C)(C)C)C=C5)N=CN=C32	7.74 ± 0.28	n.d.	n.d.	n.r.	6.35 ± 0.08	6.70 ± 0.05
**26**	OC[C@H]([C@@H](O)[C@H]1O)O[C@H]1N2C=NC3=C(N[C@@H]4CCC[C@H]4OC5=CC=CC(OC)=C5)N=CN=C32	9.28 ± 0.10	5.24±0.55	n.d.	n.r.	6.61 ± 0.07	6.56 ± 0.06
**27**	OC[C@H]([C@@H](O)[C@H]1O)O[C@H]1N2C=NC3=C(N[C@@H]4CCC[C@H]4OC5=CC=CC(Br)=C5)N=CN=C32	10.0 ± 0.24	n.d.	4.63 ± 0.12	n.r.	7.55 ± 0.11	6.94 ± 0.08
**28**	OC[C@H]([C@@H](O)[C@H]1O)O[C@H]1N2C=NC3=C(N[C@@H]4CCC[C@H]4OC5=CC=CC=C5Cl)N=CN=C32	9.03 ± 0.19	5.96 ± 0.28	5.31 ± 0.09	6.73 ± 0.42	7.17 ± 0.06	7.28 ± 0.04
**29**	OC[C@H]([C@@H](O)[C@H]1O)O[C@H]1N2C=NC3=C(N[C@@H]4CCC[C@H]4OC5=CC=CC(Cl)=C5)N=CN=C32	9.21 ± 0.19	6.26 ± 0.34	5.29 ± 0.07	6.81 ± 0.47	7.19 ± 0.07	7.36 ± 0.03
**30**	OC[C@H]([C@@H](O)[C@H]1O)O[C@H]1N2C=NC3=C(N[C@@H]4CCC[C@H]4OC5=CC=C(Cl)C=C5)N=CN=C32	8.19 ± 0.18	4.86 ± 0.58	n.d.	6.88 ± 0.60	6.23 ± 0.11	6.22 ± 0.06
**44**	O[C@@H]([C@H]1O)[C@@H](C(NCC)=O)O[C@H]1N2C=NC3=C(N[C@@H]4CCC[C@H]4OCC5=CC=CC(OC)=C5)N=CN=C32	8.67± 0.19	n.r.	4.42 ± 0.16	5.38 ± 0.09	6.39 ± 0.08	6.11 ± 0.07
**45**	O[C@@H]([C@H]1O)[C@@H](C(NCC)=O)O[C@H]1N2C=NC3=C(N[C@@H]4CCC[C@H]4OCC5=CC=CC(Br)=C5)N=CN=C32	9.85 ± 0.19	n.r.	n.d.	5.56 ± 0.10	6.54 ± 0.15	6.46 ± 0.07
**46**	O[C@@H]([C@H]1O)[C@@H](C(NCC)=O)O[C@H]1N2C=NC3=C(N[C@@H]4CCC[C@H]4OCC5=CC=C(Br)C=C5)N=CN=C32	7.97 ± 0.24	n.d.	n.d.	5.82 ± 0.20	6.15 ± 0.06	6.38 ± 0.03
**47**	O[C@@H]([C@H]1O)[C@@H](C(NCC)=O)O[C@H]1N2C=NC3=C(N[C@@H]4CCC[C@H]4OCC5=CC=CC=C5Cl)N=CN=C32	8.67 ± 0.15	5.68 ± 0.32	5.36 ± 0.08	5.85 ± 0.13	6.63 ± 0.07	6.85 ± 0.05
**48**	O[C@@H]([C@H]1O)[C@@H](C(NCC)=O)O[C@H]1N2C=NC3=C(N[C@@H]4CCC[C@H]4OCC5=CC=CC(Cl)=C5)N=CN=C32	8.29 ± 0.16	5.04 ± 0.29	4.72 ± 0.11	5.83 ± 0.39	6.49 ± 0.08	6.90 ± 0.05
**49**	O[C@@H]([C@H]1O)[C@@H](C(NCC)=O)O[C@H]1N2C=NC3=C(N[C@@H]4CCC[C@H]4OC5=CC=CC=C5)N=CN=C32	9.53 ± 0.20	5.48 ± 0.42	6.04 ± 0.09	7.17 ± 0.16	7.30 ± 0.05	7.41 ± 0.03
**50**	O[C@@H]([C@H]1O)[C@@H](C(NCC)=O)O[C@H]1N2C=NC3=C(N[C@@H]4CCC[C@H]4OC5=CC=C(C(C)(C)C)C=C5)N=CN=C32	7.81 ± 0.41	4.84 ± 0.30	4.77 ± 0.08	6.63 ± 0.19	6.35 ± 0.07	6.85 ± 0.05
**51**	O[C@@H]([C@H]1O)[C@@H](C(NCC)=O)O[C@H]1N2C=NC3=C(N[C@@H]4CCC[C@H]4OC5=CC=CC(OC)=C5)N=CN=C32	9.88 ± 0.29	5.20 ± 1.11	5.10 ± 0.07	5.56 ± 0.14	7.26 ± 0.14	6.85 ± 0.06
**52**	O[C@@H]([C@H]1O)[C@@H](C(NCC)=O)O[C@H]1N2C=NC3=C(N[C@@H]4CCC[C@H]4OC5=CC=CC(Br)=C5)N=CN=C32	9.62 ± 0.35	4.58 ± 0.87	5.37 ± 0.11	5.52 ± 0.12	7.05 ± 0.16	6.82 ± 0.10
**53**	O[C@@H]([C@H]1O)[C@@H](C(NCC)=O)O[C@H]1N2C=NC3=C(N[C@@H]4CCC[C@H]4OC5=CC=CC=C5Cl)N=CN=C32	9.91 ± 0.23	5.67 ± 0.46	6.22 ± 0.09	7.00 ± 0.19	7.39 ± 0.04	7.60 ± 0.04
**54**	O[C@@H]([C@H]1O)[C@@H](C(NCC)=O)O[C@H]1N2C=NC3=C(N[C@@H]4CCC[C@H]4OC5=CC=CC(Cl)=C5)N=CN=C32	9.28 ± 0.28	5.86 ± 0.41	6.01 ± 0.09	6.79 ± 0.14	7.43 ± 0.05	7.51 ± 0.06
**55**	O[C@@H]([C@H]1O)[C@@H](C(NCC)=O)O[C@H]1N2C=NC3=C(N[C@@H]4CCC[C@H]4OC5=CC=C(Cl)C=C5)N=CN=C32	7.99 ± 0.15	4.86 ± 0.32	5.10 ± 0.09	6.92 ± 0.17	6.86 ± 0.10	7.08 ± 0.04

n.d., not determined, full dose-response curve not feasible; n.r., no response detected in the assay; n.a., not attempted.

5. Molecular Dynamics Simulations

Movie S1 (see separate .mp4 file)**. MD Simulations of 27 (orange stick representation) in Complex with the Four AR Subtypes.** The initial binding mode in A_1R and $A_{2A}R$ was according to molecular docking, while binding mode in $A_{2B}R$ and A_3R was obtained by superimposing the pose from $A_{2A}R$. AR residues within 3.5 Å of **27** are shown throughout three MD simulations of 2 µs each.

Movie S2 (see separate .mp4 file)**. MD Simulations of 27 and 20 in Complex with A_1R (white transparent ribbon) During Three MD Simulations of 2 µs each.** Compound **27** (left side) is shown in dark green stick representation, while compound **20** (right side) is in light green stick. A_1R I69$^{2.64}$, F171^{ECL2}, N254$^{6.55}$, T257$^{6.58}$, T270$^{7.35}$ and Y271$^{7.36}$ are depicted as cyan stick, while N70$^{2.65}$ is highlighted in orange.

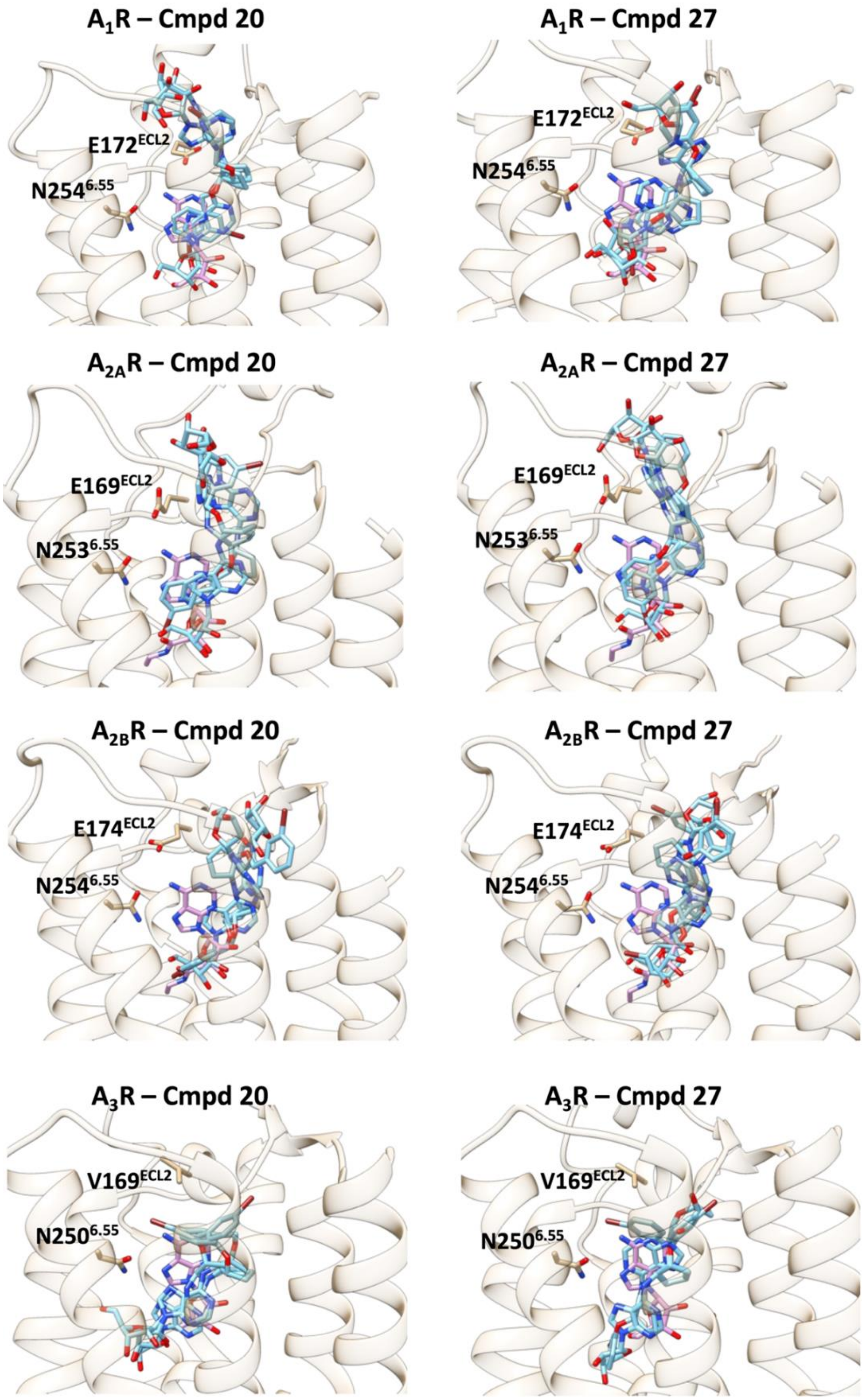

Figure S2. Initial Molecular Docking of 20 and 27 Did Not Produce Binding Modes Consistent with AR Full Agonists. The best five poses from molecular docking (cyan sticks) are reported alongside the binding mode of adenosine or NECA as a reference (purple). The conserved residue $N^{6.55}$ and the ECL2 residues above the agonist purine scaffold are shown. Computations were performed on experimental structures of A_1R (PDB ID 6D9H) and $A_{2A}R$ (PDB ID 5G53) and AlphaFold2 models of $A_{2B}R$ and A_3R. See *Experimental Section* for more details.

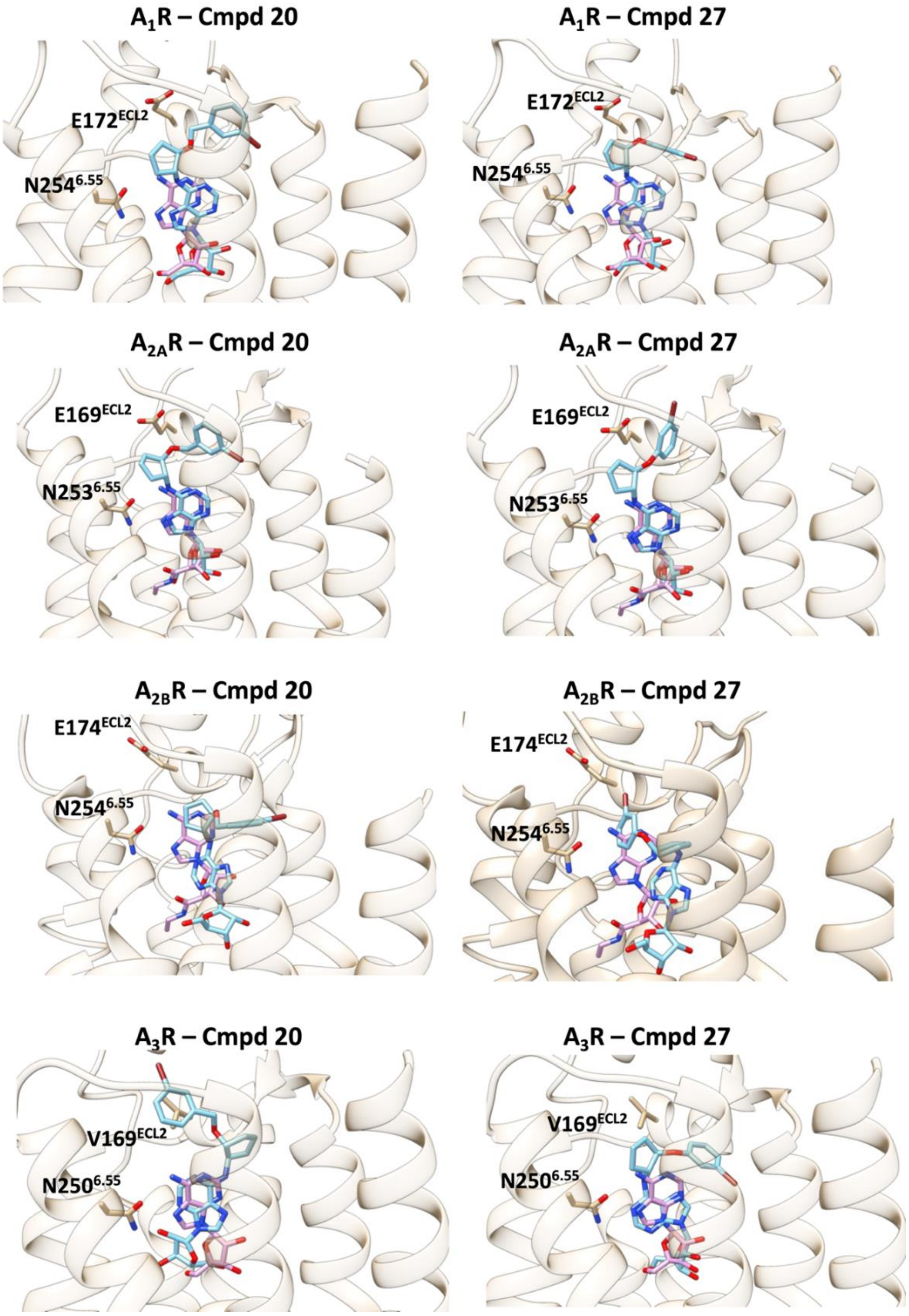

Figure S3. Molecular Docking of 20 and 27 Improved on MD-Derived Structures. The best pose from molecular docking (cyan sticks) is reported alongside the binding mode of adenosine or NECA as a reference (purple). The conserved residue $N^{6.55}$ and the ECL2 residues above the agonist purine scaffold are shown. Computations were performed on AR structures extracted from MD simulations of the apo receptors (experimental structures of A_1R (PDB ID 6DH9) and $A_{2A}R$ (PDB ID 5G53) and AlphaFold2 models of $A_{2B}R$ and A_3R). See *Experimental Section* for more details.

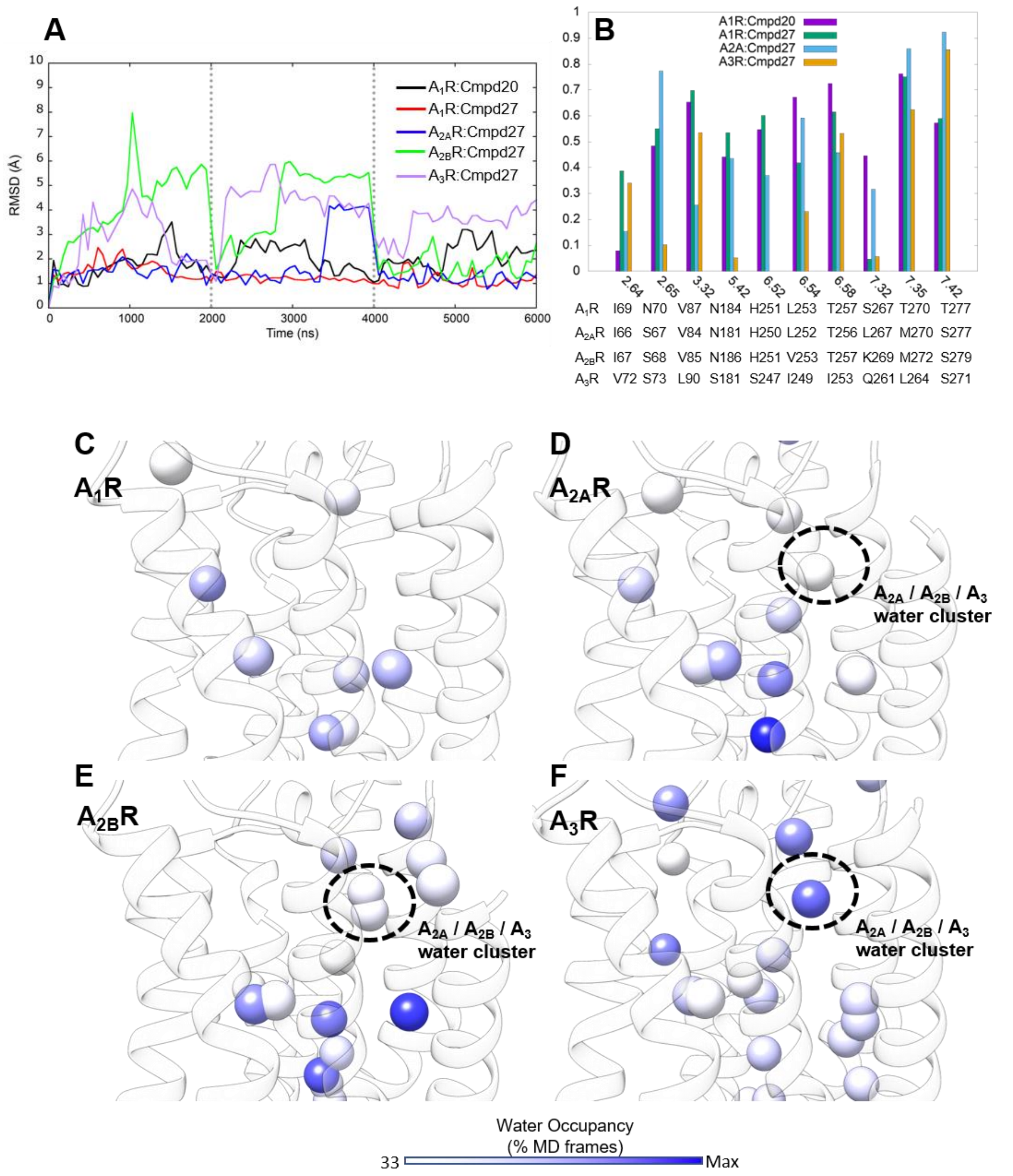

Figure S4. Molecular Dynamics Simulations of AR-Bound 20 and 27, and of Water Molecules in the Apo Receptors. (**A**) RMSD of **20** (bound to A_1R) and **27** (within all the four AR subtypes) during three MD replicas (2 µs each). (**B**) Contacts between **20** and A_1R or **27** and A_1R, $A_{2A}R$, A_3R. Orthosteric residues that differ in at least one receptor subtype are reported. (**C**-**F**) Positions occupied by structural water molecules within the four AR subtypes in the absence of any agonist (apo receptors). The intensity of the blue color correlates with the occupancy displayed during the AquaMMapS analysis. Presented data are based on experimental structures (PDB IDs 6D9H (A_1R) and 5G53 ($A_{2A}R$)), and AlphaFold2 models of $A_{2B}R$ and A_3R. See *Experimental Section* for more details.

6. *Validation of Molecular Dynamics Simulations*

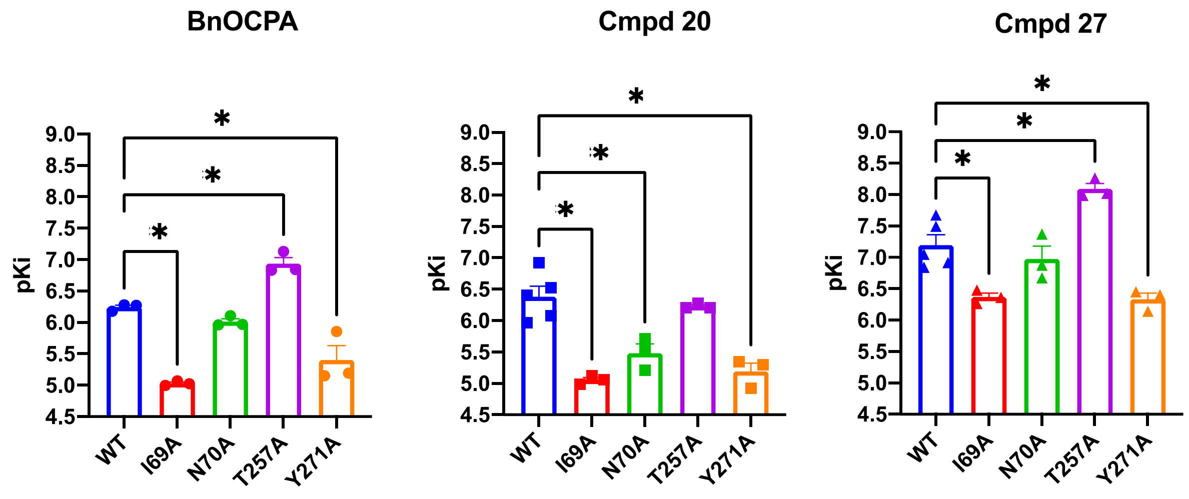

Figure S5. Binding Affinity of BnOCPA, 20 and 27 to Wild Type (WT) or Mutant Variants of the Human A_1R Measured by NanoBRET. pK_i values for the unlabeled AR ligands BnOCPA, **20** and **27** at WT and mutant versions of hNluc-A_1R expressed in HEK293 cells. Each version of the A_1R was treated with 20 nM CA200645 and increasing concentrations of unlabeled AR ligands enabling calculation of their pK_i values as shown. Data are the mean ± SEM of at least three independent repeats, conducted in duplicate. Statistical significance (* $p < 0.05$) determined using one-way ANOVA and Dunnett's post-test, presented as described in ref 31.

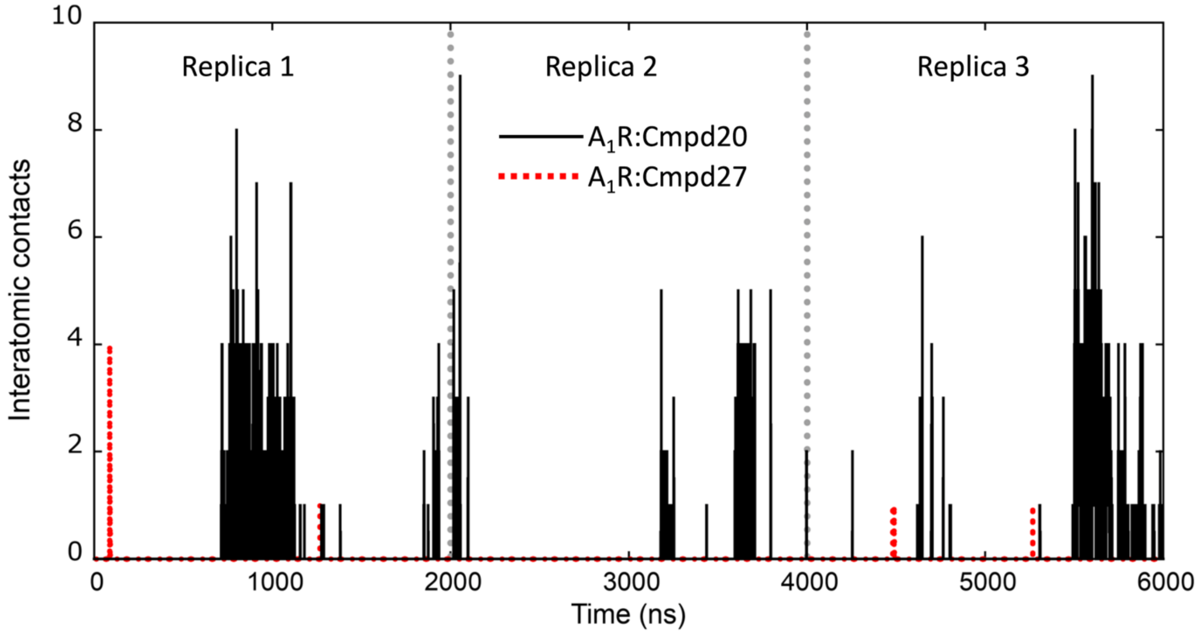

Figure S6. Interatomic contacts formed by the A_1R residue N70$^{2.65}$ and the purine ring of **20** or **27** during three MD simulation replicas (total of 6 μs). A contact was considered productive if the interatomic distance reached less than 4 Å.

7. *^{1}H and ^{13}C NMR Spectra for Final Compounds*

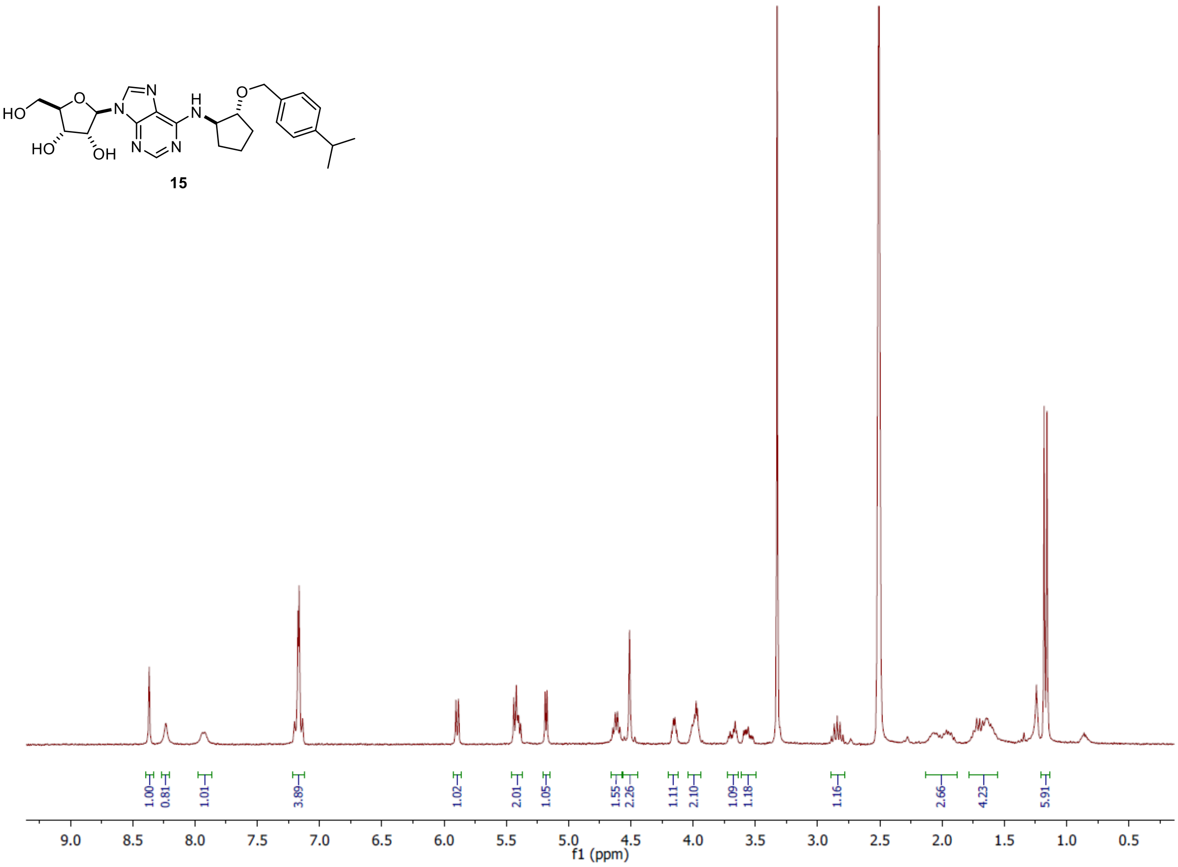

15

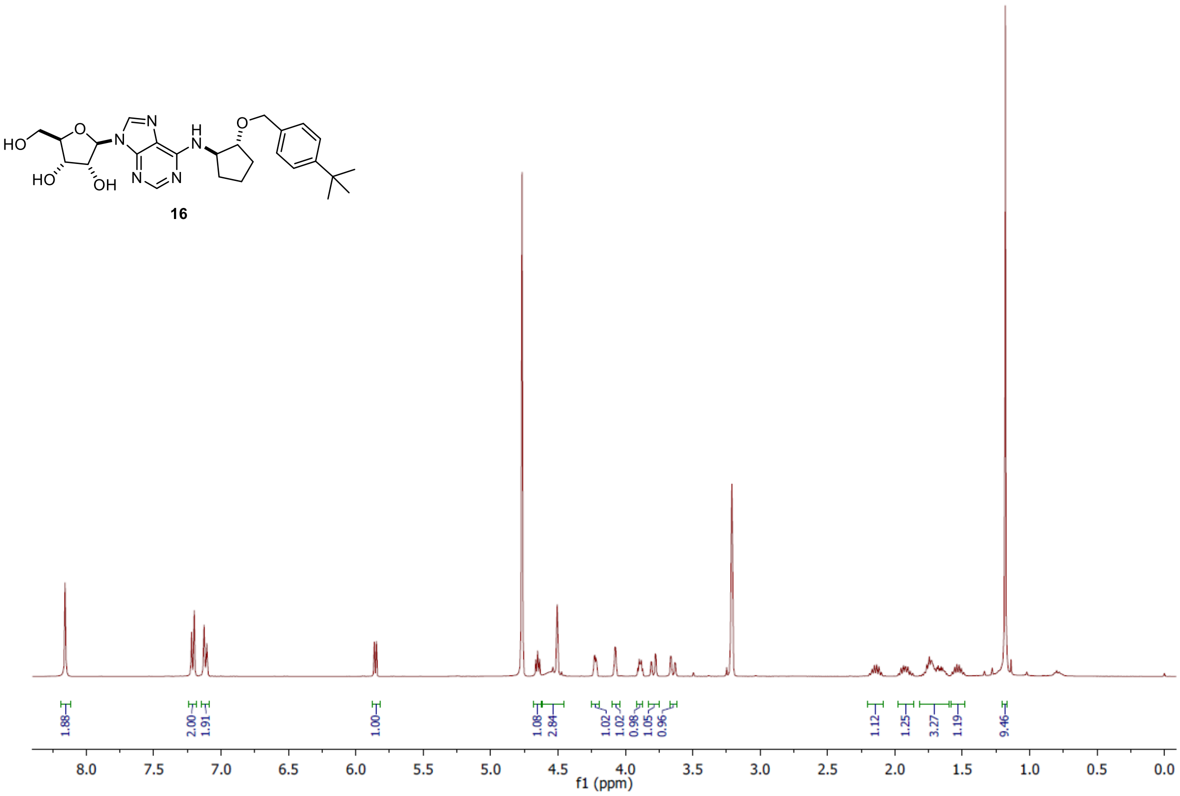

16

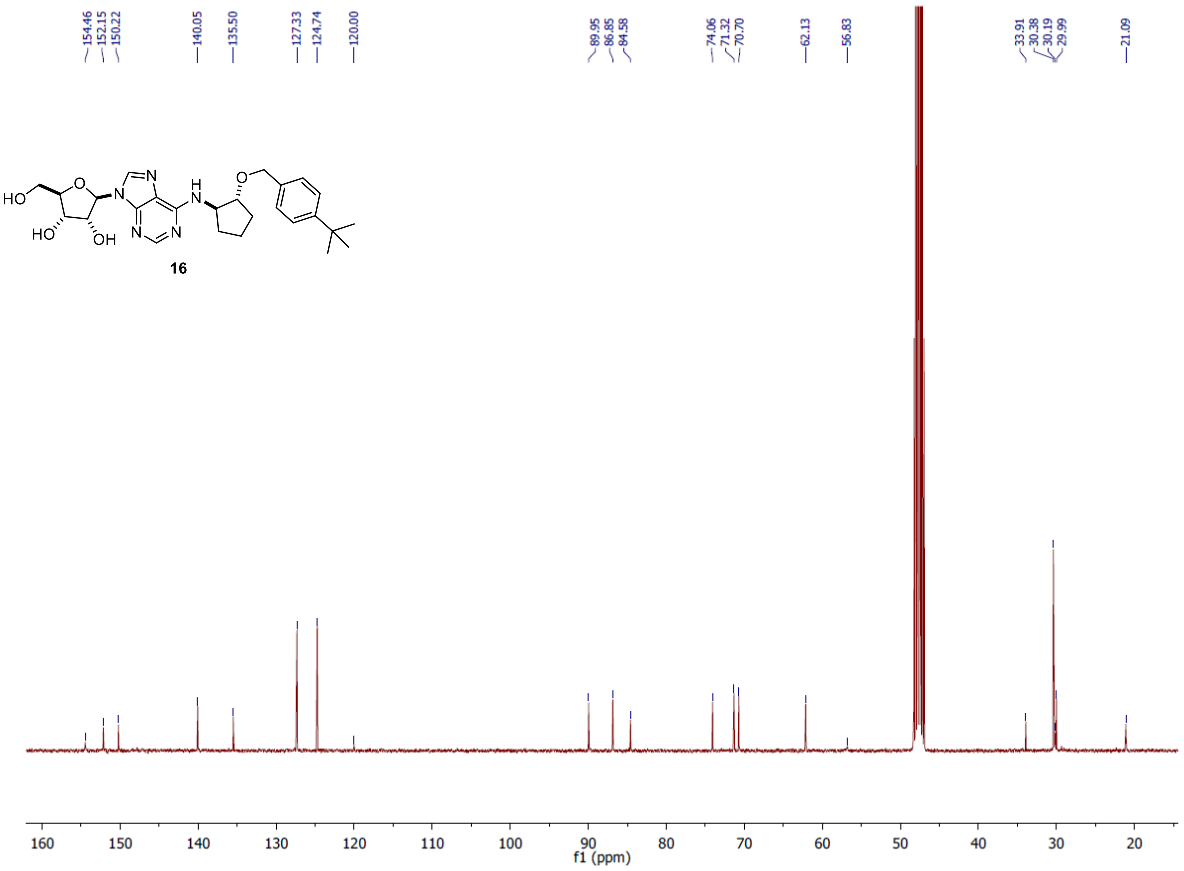

16
154.46
152.15
150.22
140.05
135.50
127.33
124.74
120.00
89.95
86.85
84.58
74.06
71.32
70.70
62.13
56.83
33.91
30.38
30.19
29.99
21.09
160 150 140 130 120 110 100 90 80 70 60 50 40 30 20
f1 (ppm)

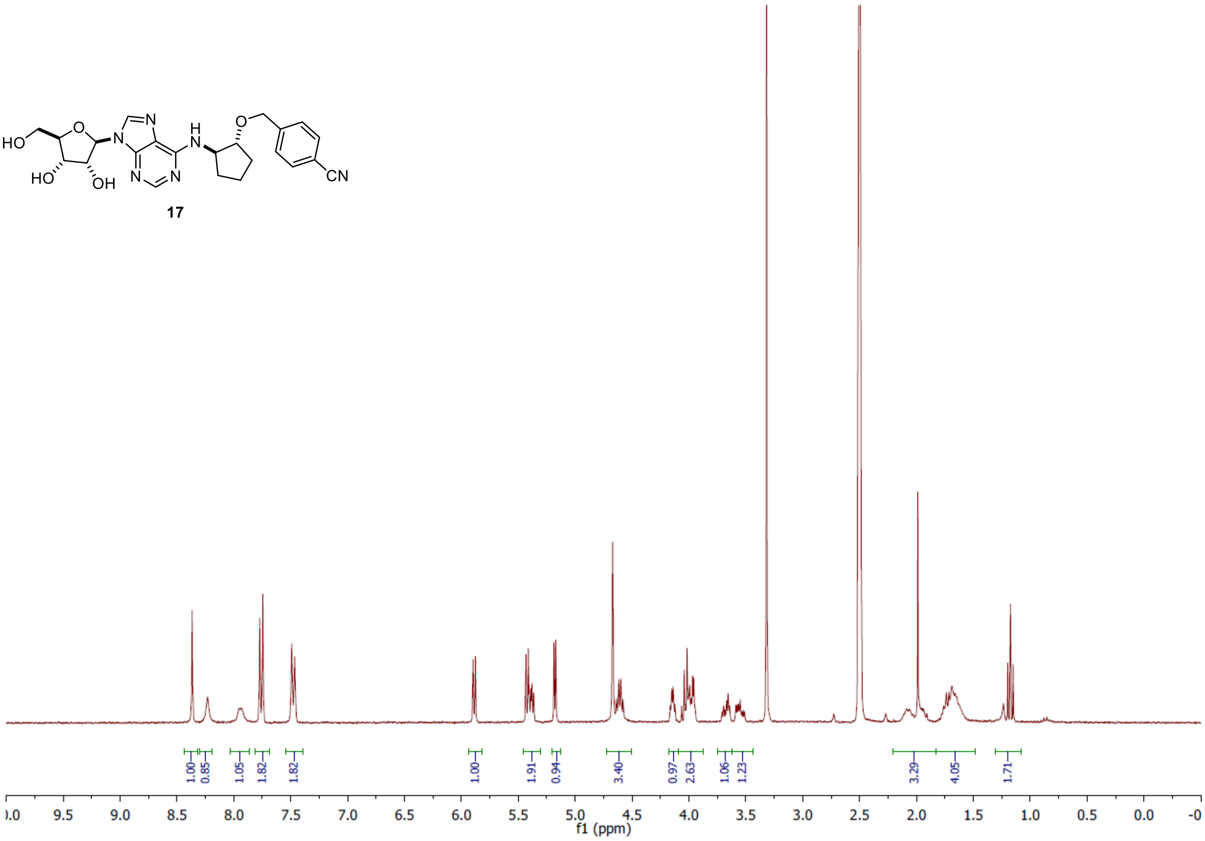

17
1.00
0.85
1.05
1.82
1.82
1.00
1.91
0.94
3.40
0.97
2.63
1.06
1.23
3.29
4.05
1.71
9.5 9.0 8.5 8.0 7.5 7.0 6.5 6.0 5.5 5.0 4.5 4.0 3.5 3.0 2.5 2.0 1.5 1.0 0.5 0.0
f1 (ppm)

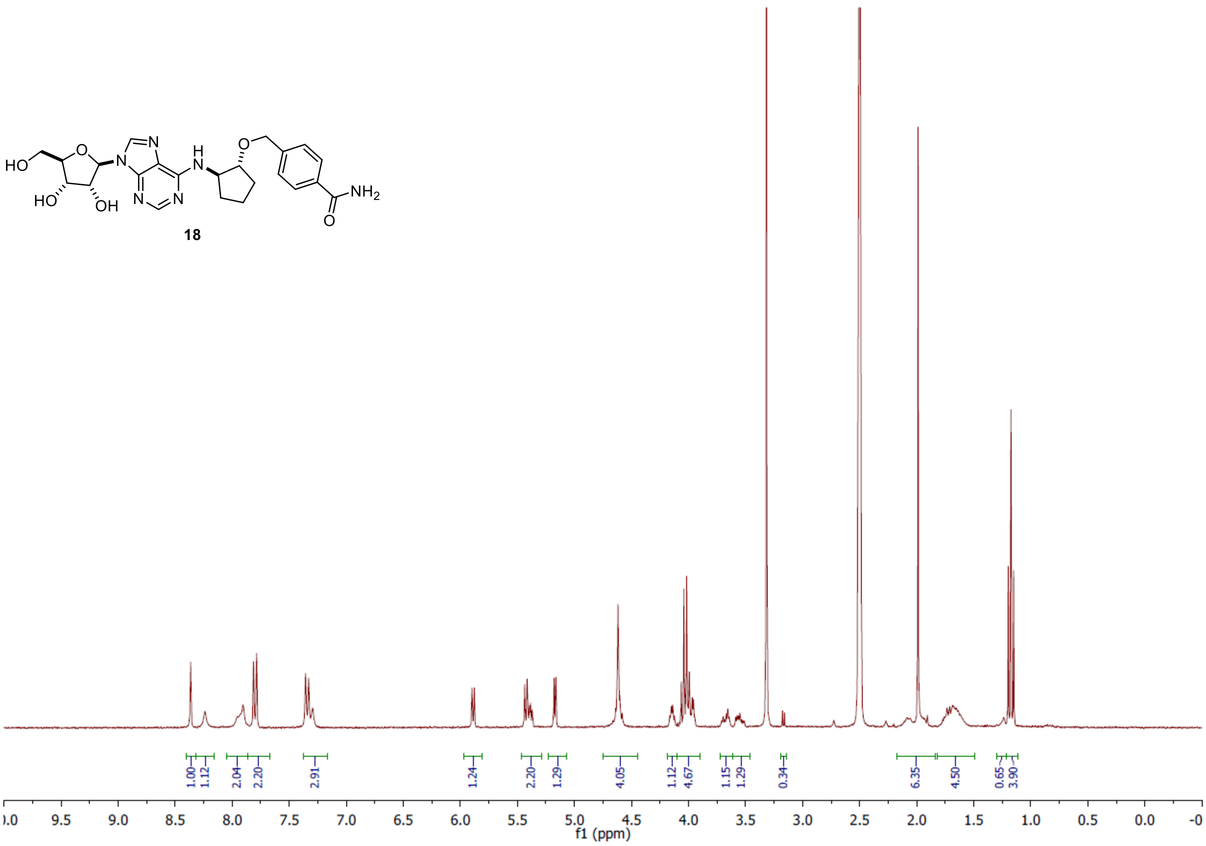
18
f1 (ppm)

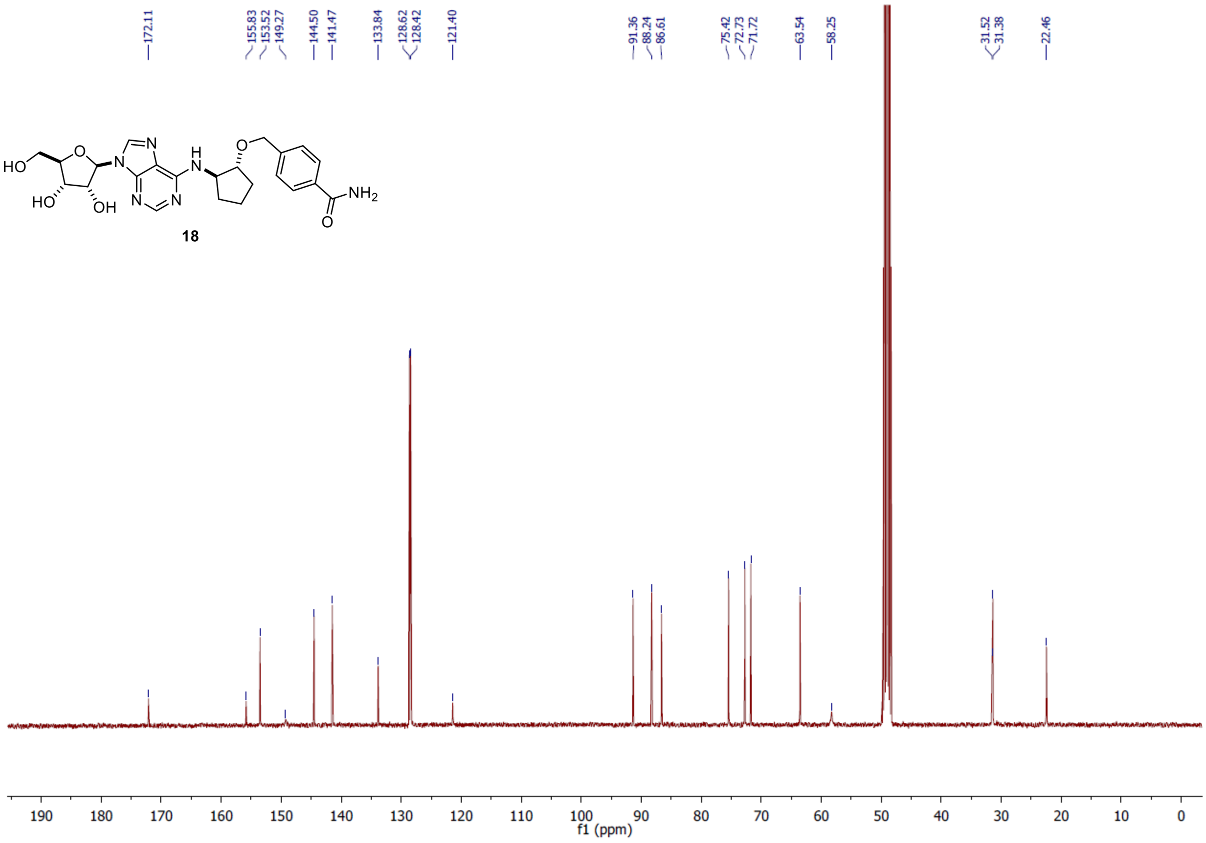
172.11
155.83
153.52
149.27
144.50
141.47
133.84
128.62
128.42
121.40
91.36
88.24
86.61
75.42
72.73
71.72
63.54
58.25
31.52
31.38
22.46
18
f1 (ppm)

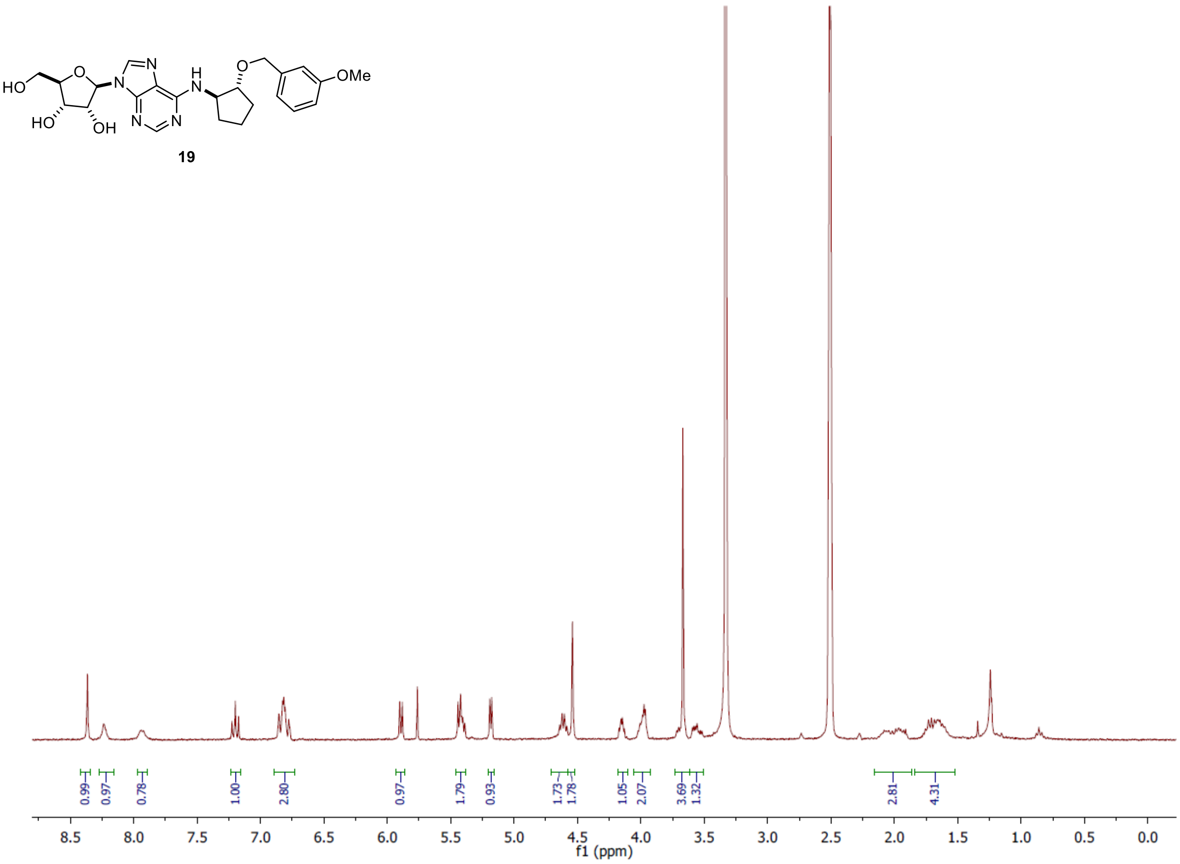
HO
O
N
N
H
N
O
OMe
HO
OH
N
N
19
0.99
0.97
0.78
1.00
2.80
0.97
1.79
0.93
1.73
1.78
1.05
2.07
3.69
1.32
2.81
4.31
8.5
8.0
7.5
7.0
6.5
6.0
5.5
5.0
4.5
4.0
3.5
3.0
2.5
2.0
1.5
1.0
0.5
0.0
f1 (ppm)

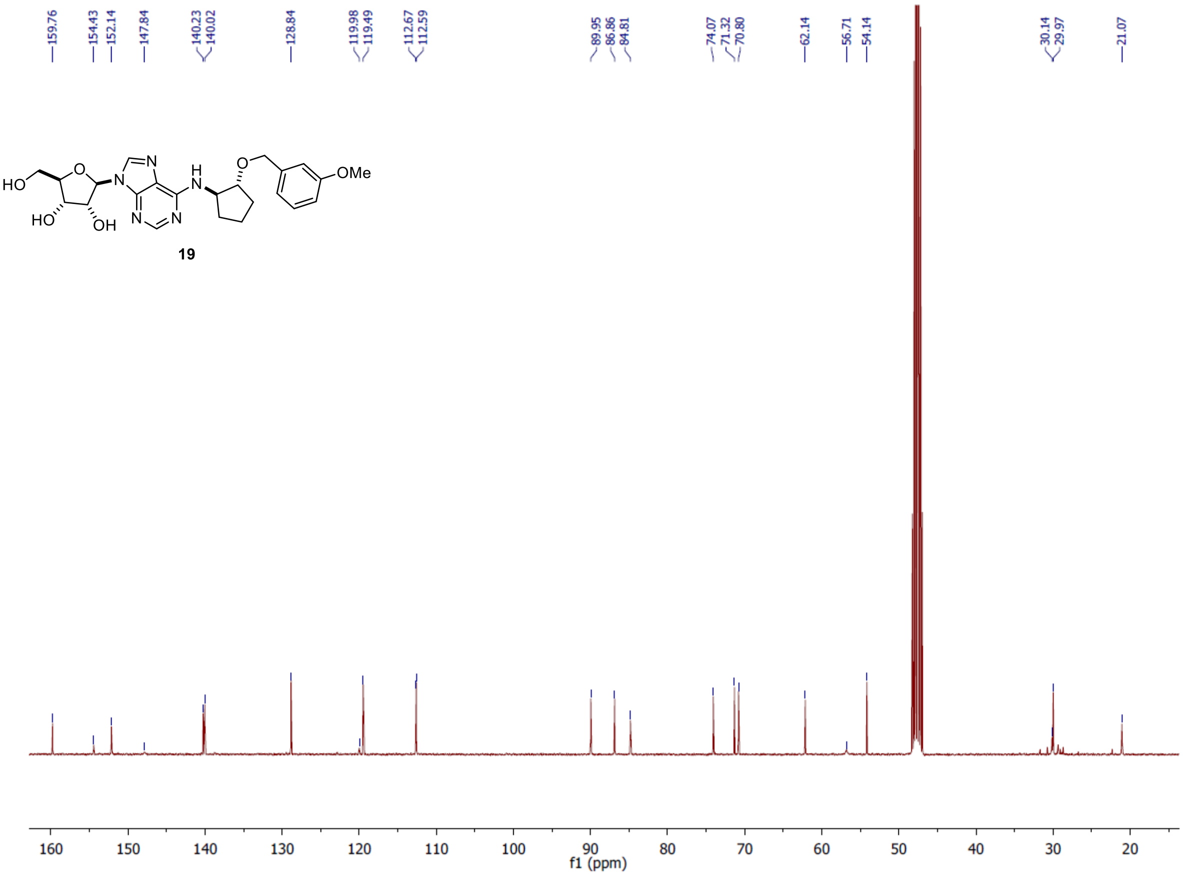
159.76
154.43
152.14
147.84
140.23
140.02
128.84
119.98
119.49
112.67
112.59
89.95
86.86
84.81
74.07
71.32
70.80
62.14
56.71
54.14
30.14
29.97
21.07
HO
O
N
N
H
N
O
OMe
HO
OH
N
N
19
160
150
140
130
120
110
100
90
80
70
60
50
40
30
20
f1 (ppm)

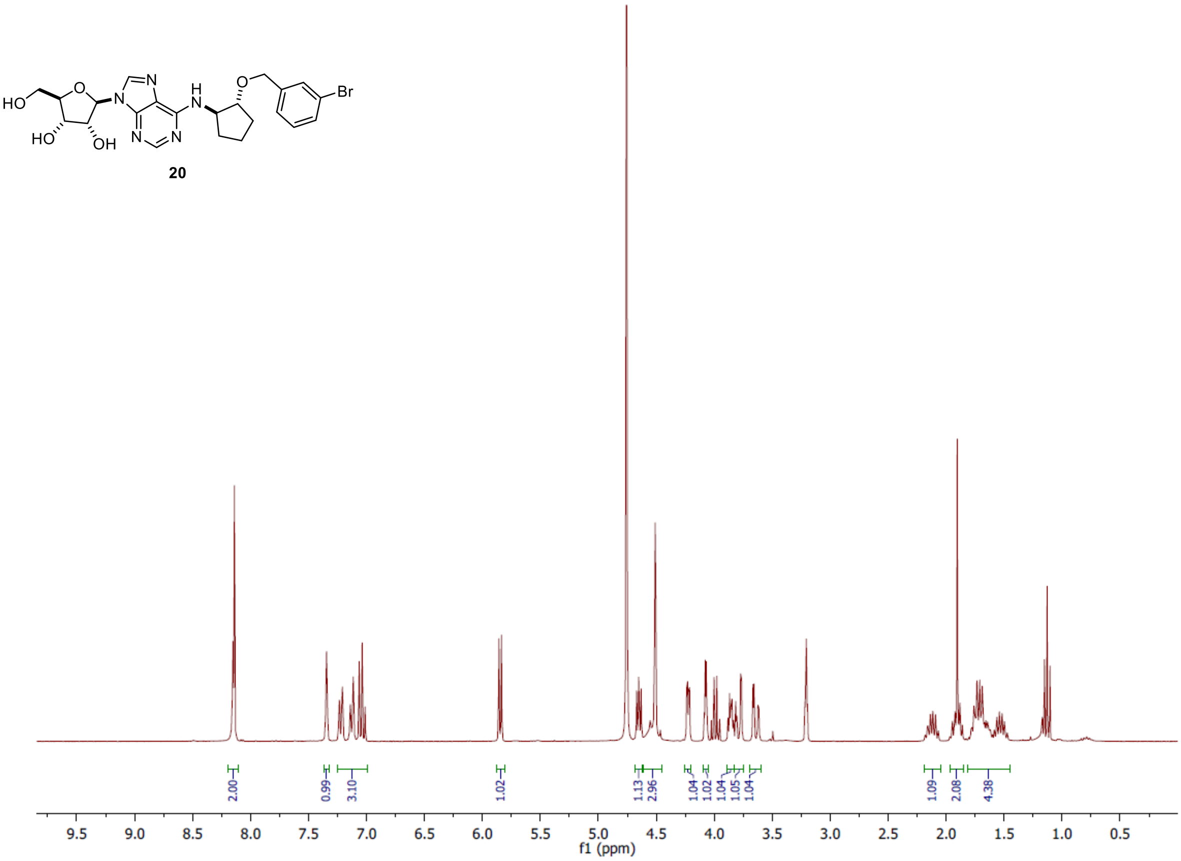

20
2.00
0.99
3.10
1.02
1.13
2.96
1.04
1.02
1.04
1.05
1.04
1.09
2.08
4.38
9.5 9.0 8.5 8.0 7.5 7.0 6.5 6.0 5.5 5.0 4.5 4.0 3.5 3.0 2.5 2.0 1.5 1.0 0.5
f1 (ppm)

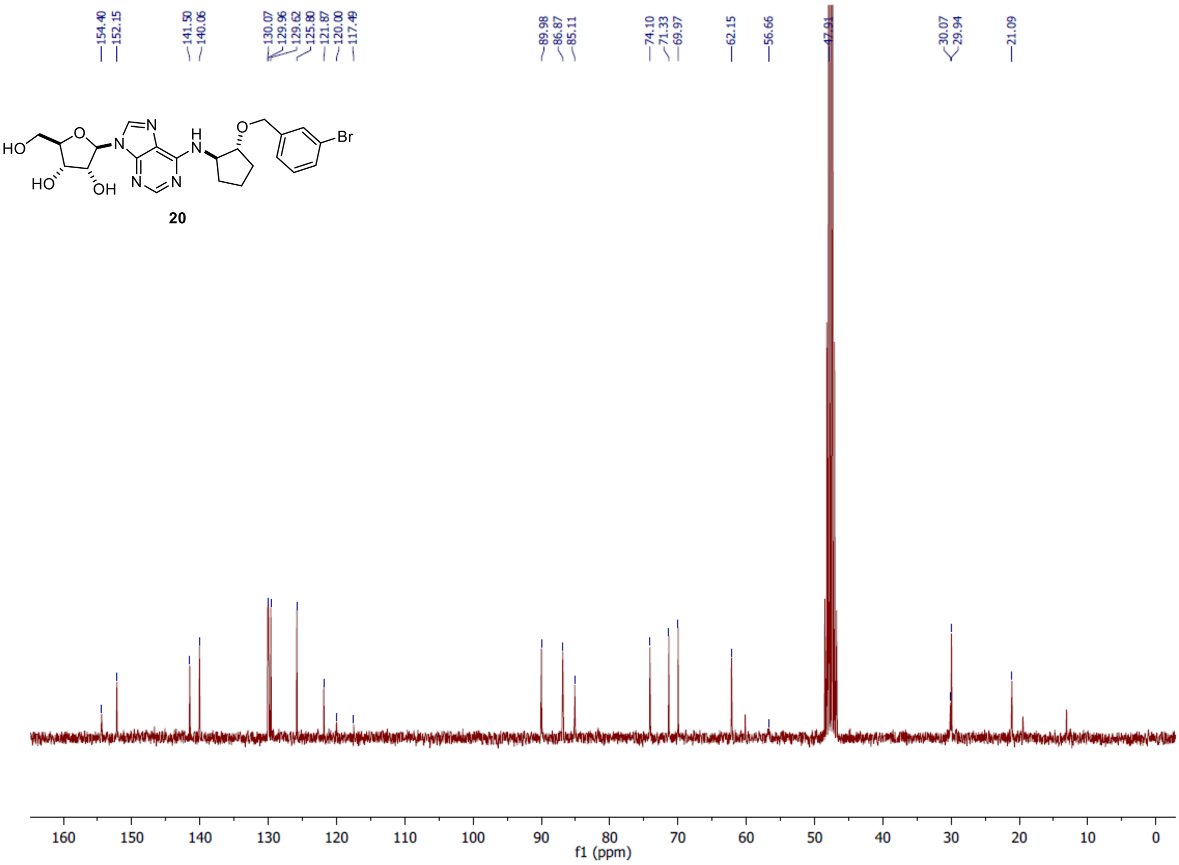

20
154.40
152.15
141.50
140.06
130.07
129.96
129.62
125.80
121.87
120.00
117.49
89.98
86.87
85.11
74.10
71.33
69.97
62.15
56.66
47.91
30.07
29.94
21.09
160 150 140 130 120 110 100 90 80 70 60 50 40 30 20 10 0
f1 (ppm)

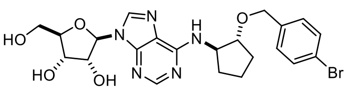
HO
O
N
N
N
N
H
N
O
Br
HO
OH

21

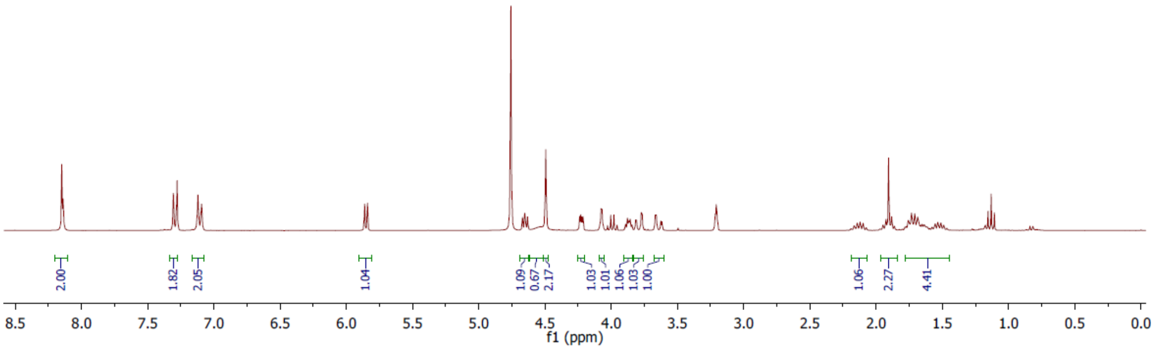
2.00
1.82
2.05
1.04
1.09
0.67
2.17
1.03
1.01
1.06
1.03
1.00
1.06
2.27
4.41
8.5
8.0
7.5
7.0
6.5
6.0
5.5
5.0
4.5
4.0
3.5
3.0
2.5
2.0
1.5
1.0
0.5
0.0
f1 (ppm)

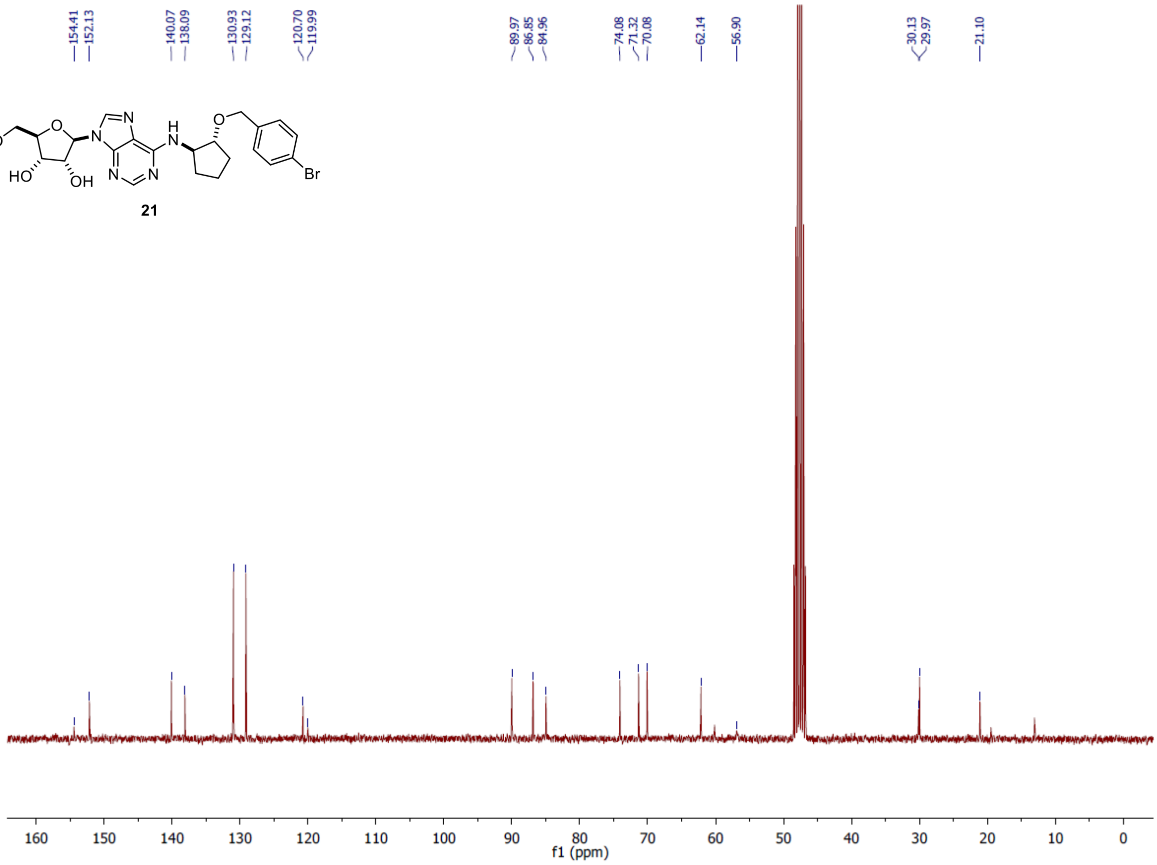
154.41
152.13
140.07
138.09
130.93
129.12
120.70
119.99
89.97
86.85
84.96
74.08
71.32
70.08
62.14
56.90
30.13
29.97
21.10
160
150
140
130
120
110
100
90
80
70
60
50
40
30
20
10
0
f1 (ppm)
HO
O
N
N
N
N
H
N
O
Br
HO
OH

21

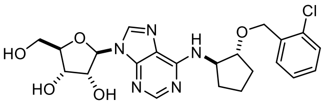
HO
O
N
N
N
N
H
N
O
Cl
HO
OH

22

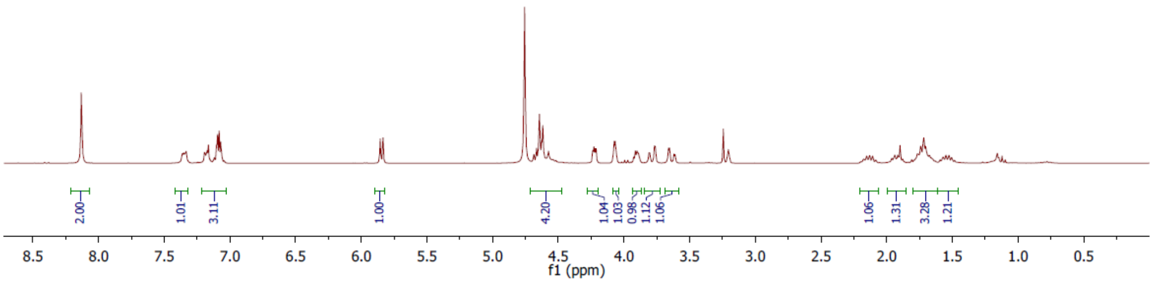
2.00
1.01
3.11
1.00
4.20
1.04
1.03
0.98
1.12
1.06
1.06
1.31
3.28
1.21
8.5 8.0 7.5 7.0 6.5 6.0 5.5 5.0 4.5 4.0 3.5 3.0 2.5 2.0 1.5 1.0 0.5
f1 (ppm)

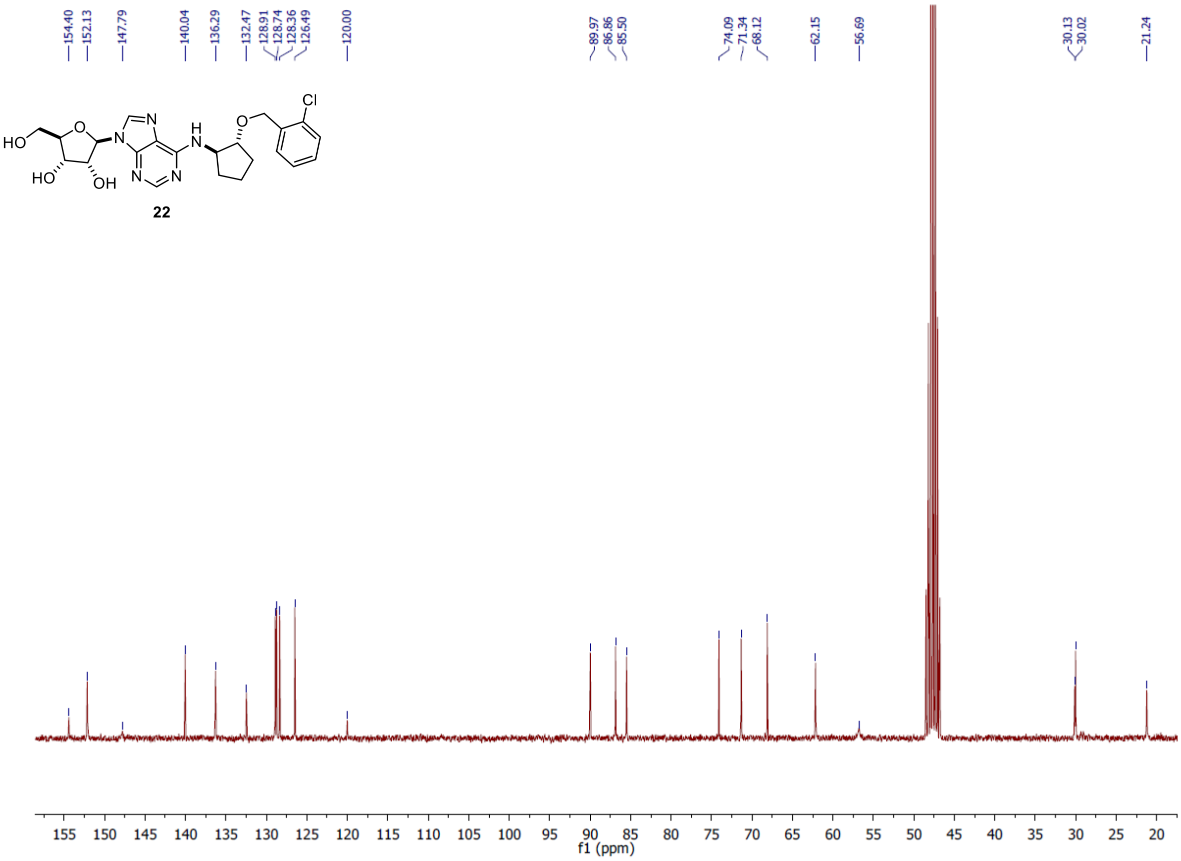
154.40
152.13
147.79
140.04
136.29
132.47
128.91
128.74
128.36
126.49
120.00
89.97
86.86
85.50
74.09
71.34
68.12
62.15
56.69
30.13
30.02
21.24
155 150 145 140 135 130 125 120 115 110 105 100 95 90 85 80 75 70 65 60 55 50 45 40 35 30 25 20
f1 (ppm)
HO
O
N
N
N
N
H
N
O
Cl
HO
OH
22

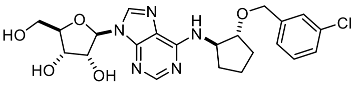
HO
O
N
N
H
N
O
Cl
HO
OH
N
N

23

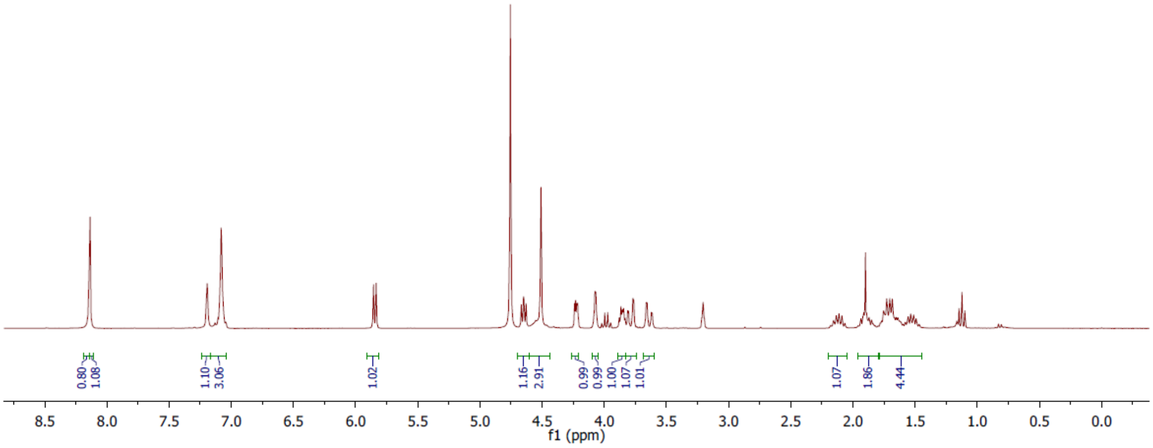
0.80
1.08
1.10
3.06
1.02
1.16
2.91
0.99
0.99
1.00
1.07
1.01
1.07
1.86
4.44
8.5 8.0 7.5 7.0 6.5 6.0 5.5 5.0 4.5 4.0 3.5 3.0 2.5 2.0 1.5 1.0 0.5 0.0
f1 (ppm)

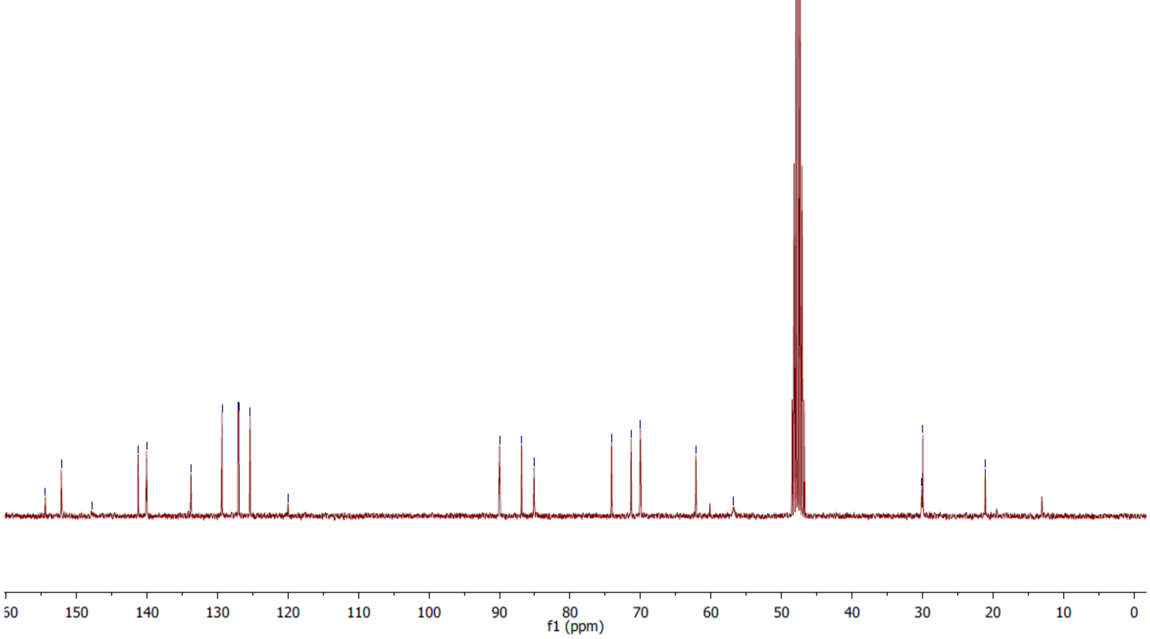
154.39
152.15
147.78
141.25
140.06
133.76
129.34
127.08
126.97
125.36
119.99
89.98
86.86
85.09
74.10
71.33
70.01
62.15
56.81
30.07
29.94
21.10
150 140 130 120 110 100 90 80 70 60 50 40 30 20 10 0
f1 (ppm)

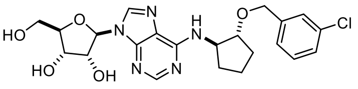
HO
O
N
N
H
N
O
Cl
HO
OH
N
N

23

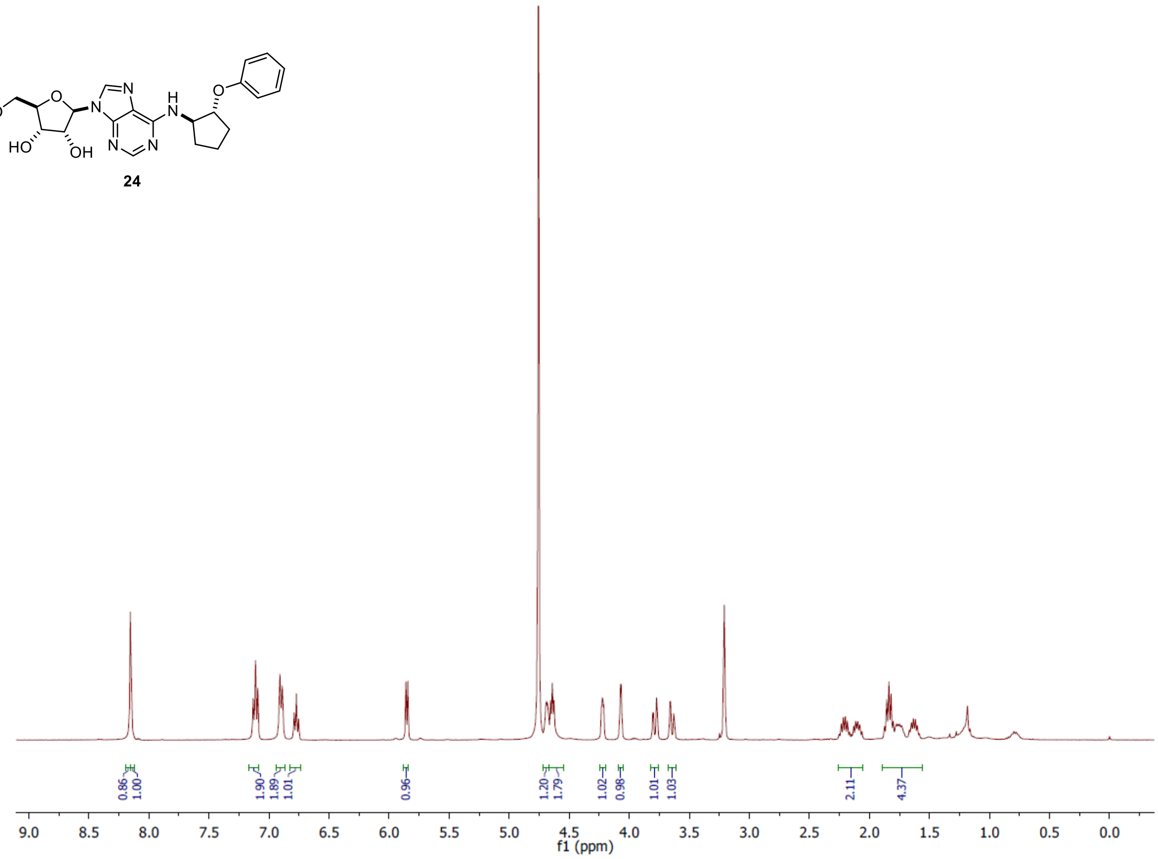

24
0.86 1.00 1.90 1.89 1.01 0.96 1.20 1.79 1.02 0.98 1.01 1.03 2.11 4.37
9.0 8.5 8.0 7.5 7.0 6.5 6.0 5.5 5.0 4.5 4.0 3.5 3.0 2.5 2.0 1.5 1.0 0.5 0.0
f1 (ppm)

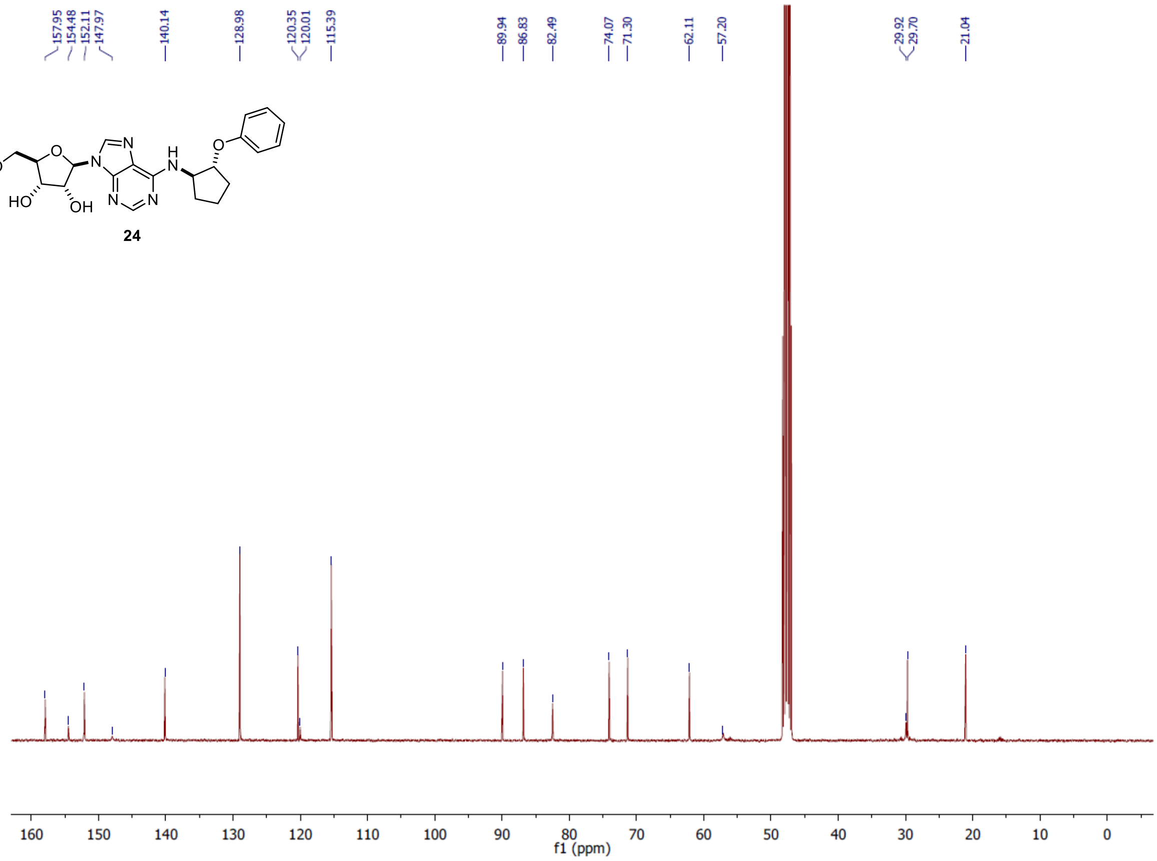

24
157.95 154.48 152.11 147.97 140.14 128.98 120.35 120.01 115.39 89.94 86.83 82.49 74.07 71.30 62.11 57.20 29.92 29.70 21.04
160 150 140 130 120 110 100 90 80 70 60 50 40 30 20 10 0
f1 (ppm)

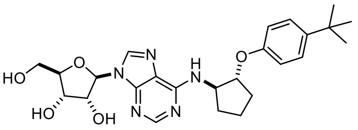

25

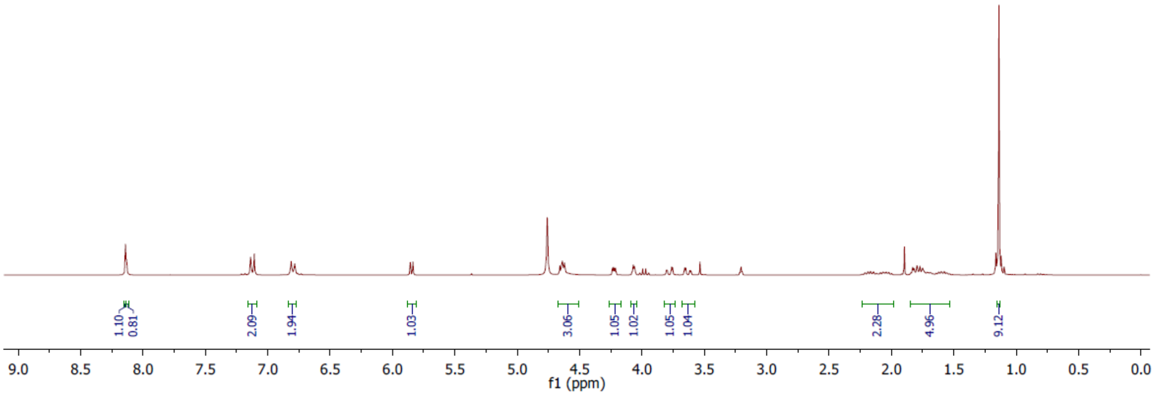
1.10
0.81
2.09
1.94
1.03
3.06
1.05
1.02
1.05
1.04
2.28
4.96
9.12
9.0 8.5 8.0 7.5 7.0 6.5 6.0 5.5 5.0 4.5 4.0 3.5 3.0 2.5 2.0 1.5 1.0 0.5 0.0
f1 (ppm)

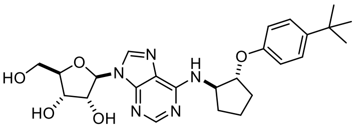

25

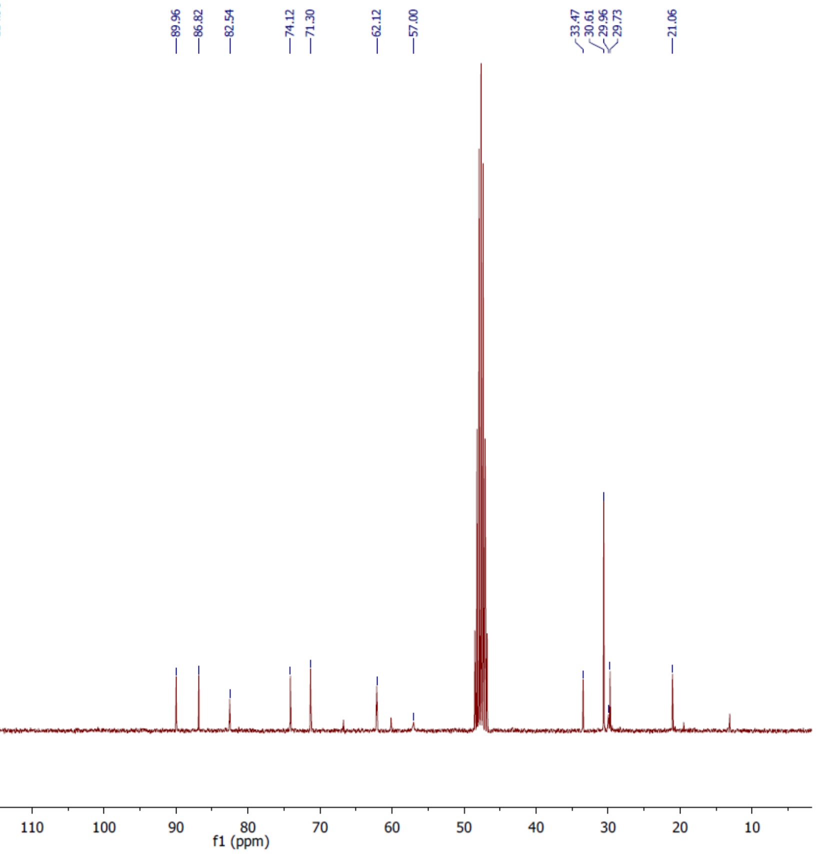
155.59
154.43
152.14
147.90
143.11
140.12
125.73
119.99
114.96
89.96
86.82
82.54
74.12
71.30
62.12
57.00
33.47
30.61
29.96
29.73
21.06
60 150 140 130 120 110 100 90 80 70 60 50 40 30 20 10
f1 (ppm)

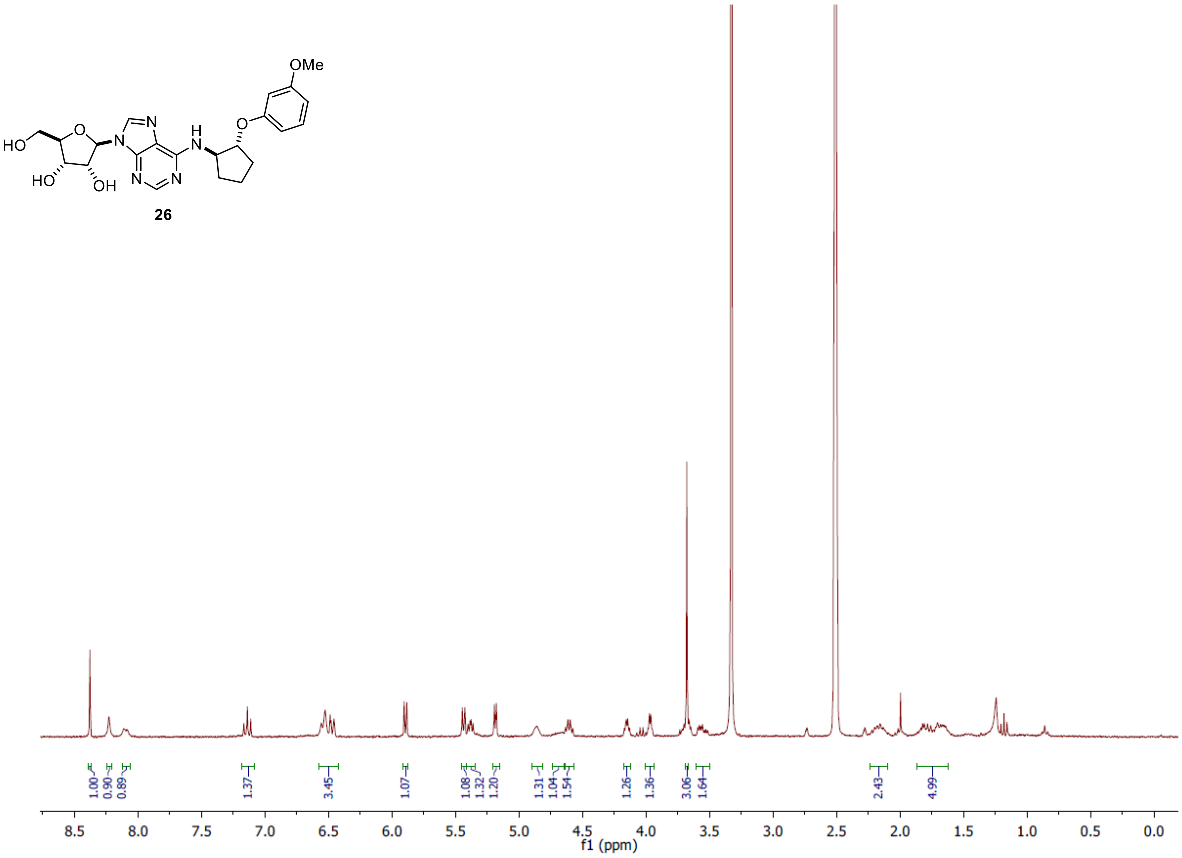
OMe
HO
O
N
N
H
N
O
HO
OH
N
N
26
1.00
0.90
0.89
1.37
3.45
1.07
1.08
1.32
1.20
1.31
1.04
1.54
1.26
1.36
3.06
1.64
2.43
4.99
8.5 8.0 7.5 7.0 6.5 6.0 5.5 5.0 4.5 4.0 3.5 3.0 2.5 2.0 1.5 1.0 0.5 0.0
f1 (ppm)

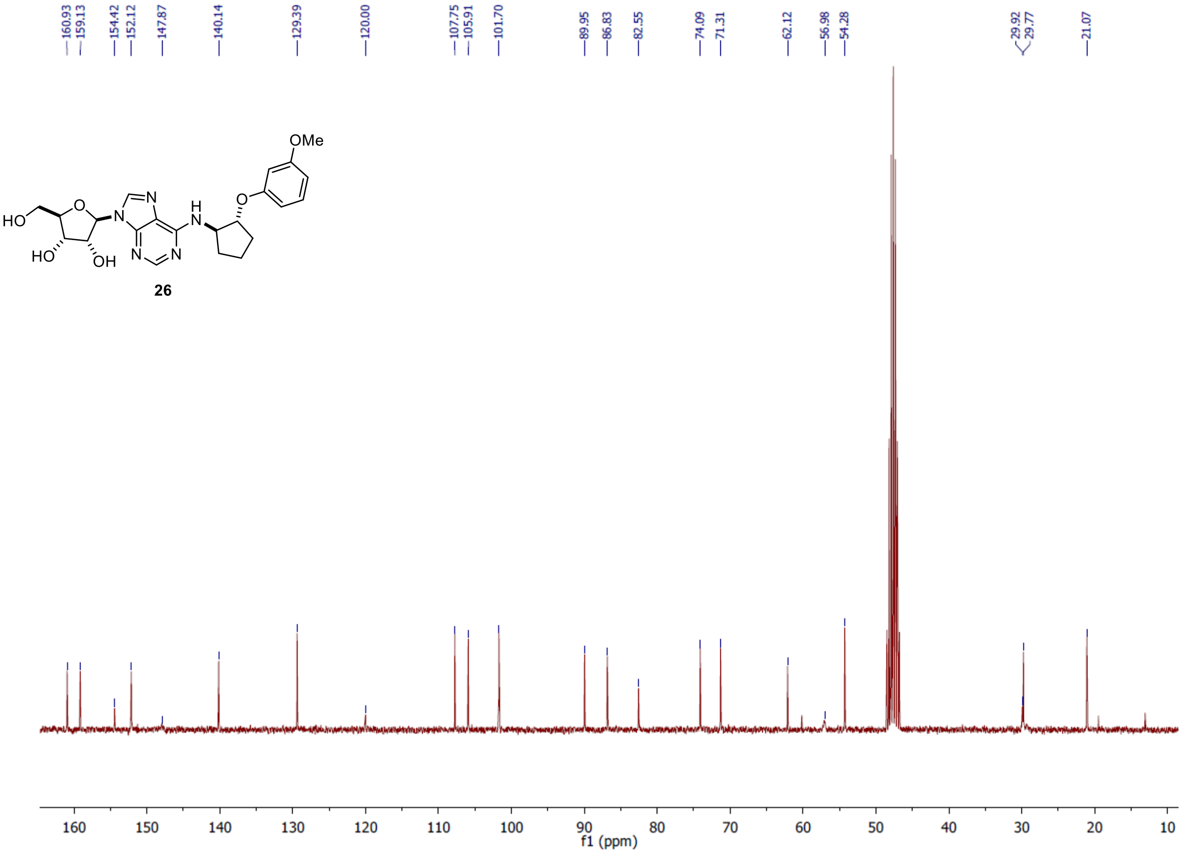
160.93
159.13
154.42
152.12
147.87
140.14
129.39
120.00
107.75
105.91
101.70
89.95
86.83
82.55
74.09
71.31
62.12
56.98
54.28
29.92
29.77
21.07
OMe
HO
O
N
N
H
N
O
HO
OH
N
N
26
160 150 140 130 120 110 100 90 80 70 60 50 40 30 20 10
f1 (ppm)

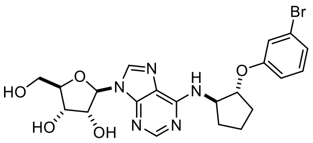
Br
O
HO
O
N
N
H
N
N
N
HO
OH

27

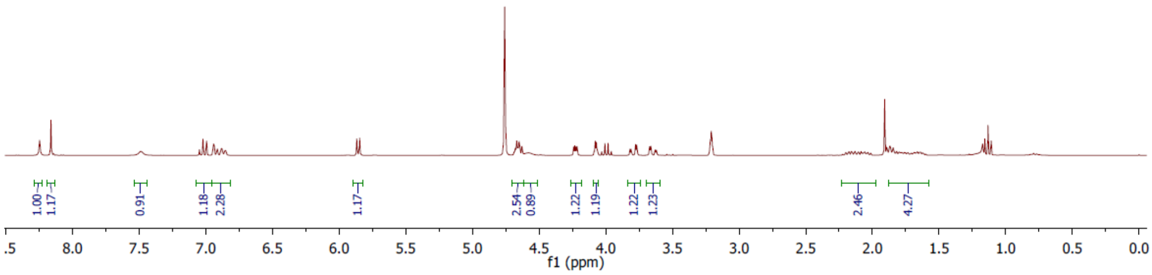
1.00
1.17
0.91
1.18
2.28
1.17
2.54
0.89
1.22
1.19
1.22
1.23
2.46
4.27
f1 (ppm)

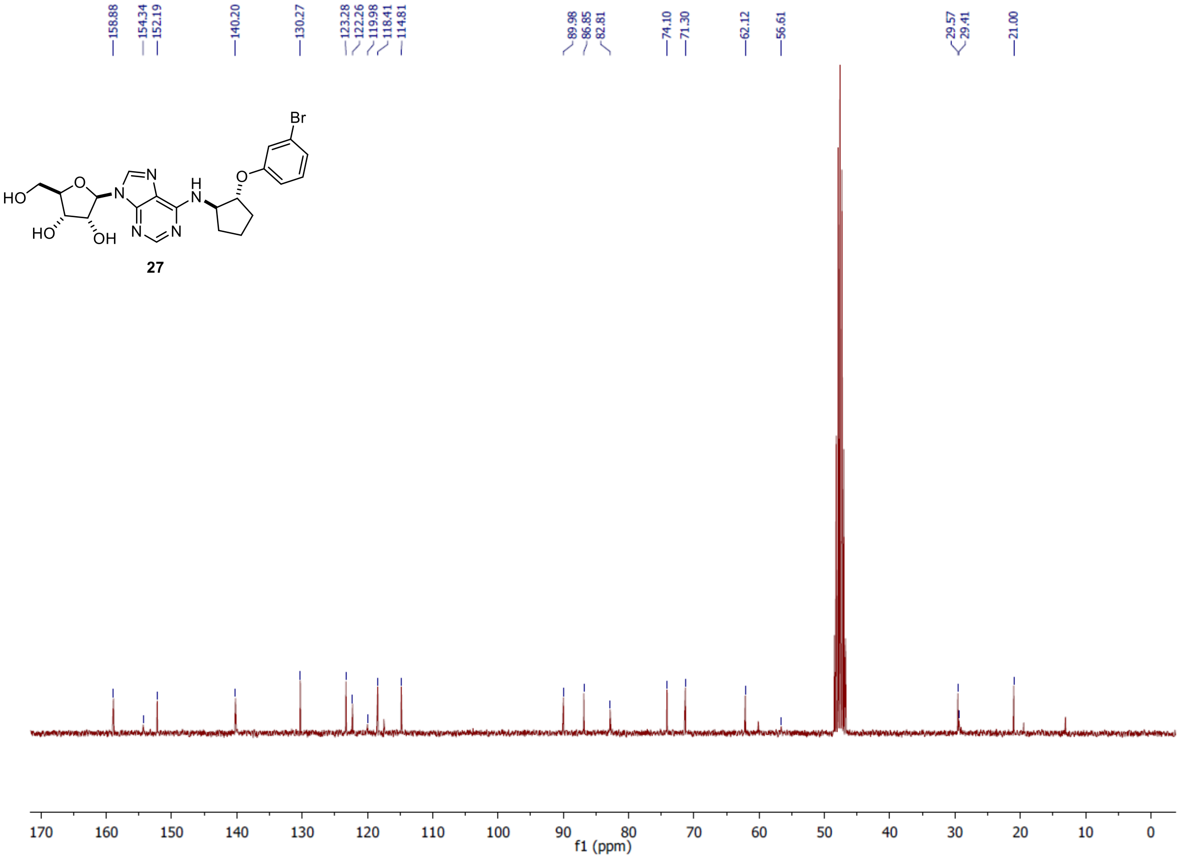
158.88
154.34
152.19
140.20
130.27
123.28
122.26
119.98
118.41
114.81
89.98
86.85
82.81
74.10
71.30
62.12
56.61
29.57
29.41
21.00
f1 (ppm)

27

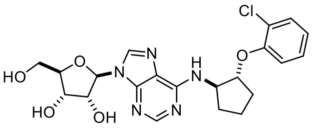
Cl
HO
O
N
N
H
N
O
HO
OH
N
N

28

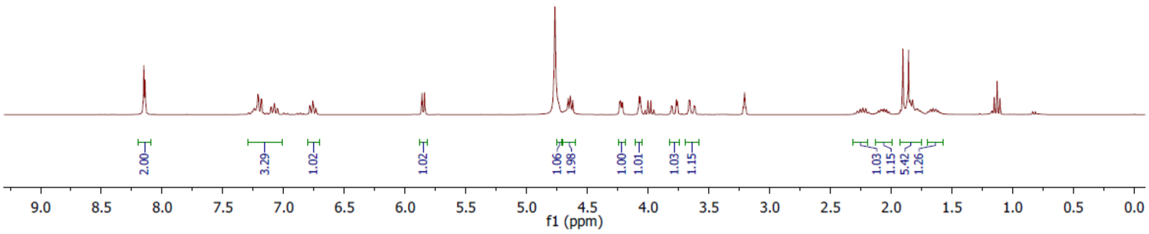
2.00
3.29
1.02
1.02
1.06
1.98
1.00
1.01
1.03
1.15
1.03
1.15
5.42
1.26
9.0 8.5 8.0 7.5 7.0 6.5 6.0 5.5 5.0 4.5 4.0 3.5 3.0 2.5 2.0 1.5 1.0 0.5 0.0
f1 (ppm)

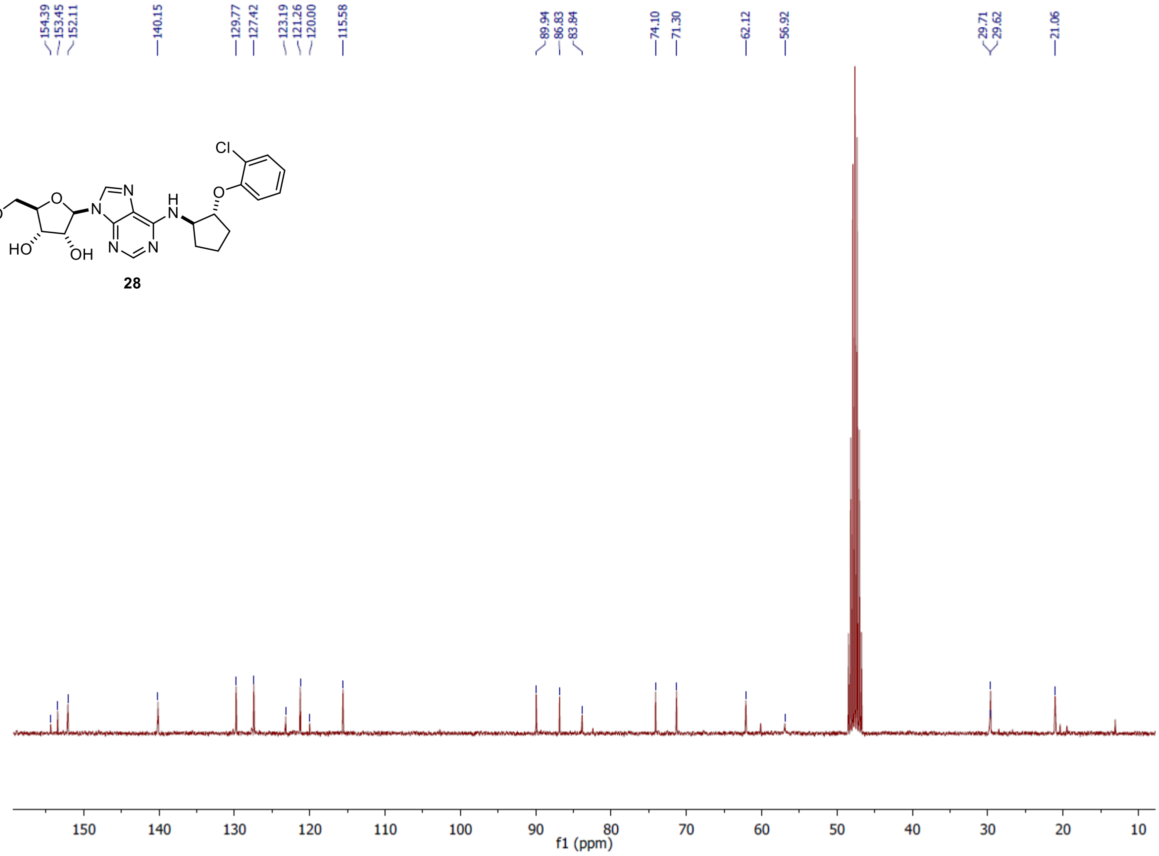
154.39
153.45
152.11
140.15
129.77
127.42
123.19
121.26
120.00
115.58
89.94
86.83
83.84
74.10
71.30
62.12
56.92
29.71
29.62
21.06
150 140 130 120 110 100 90 80 70 60 50 40 30 20 10
f1 (ppm)
Cl
HO
O
N
N
H
N
O
HO
OH
N
N

28

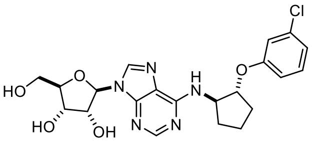
Cl
O
HO
O
N
N
H
N
HO
OH
N
N

29

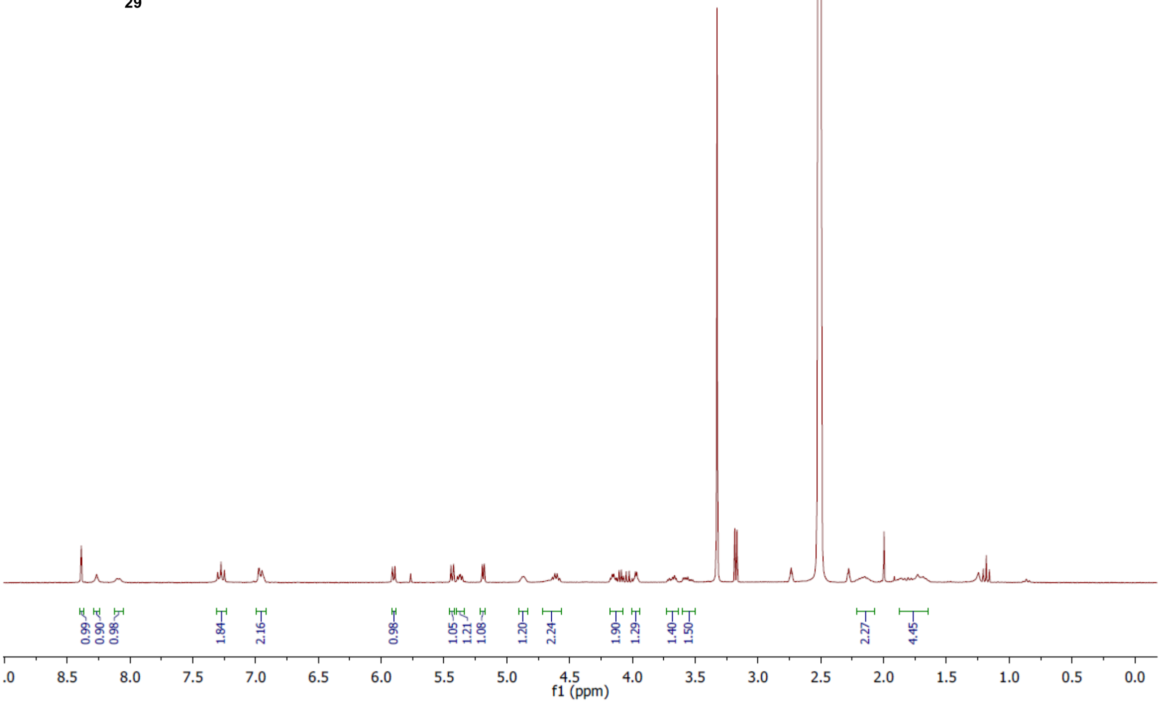
0.99
0.90
0.98
1.84
2.16
0.98
1.05
1.21
1.08
1.20
2.24
1.90
1.29
1.40
1.50
2.27
4.45
.0 8.5 8.0 7.5 7.0 6.5 6.0 5.5 5.0 4.5 4.0 3.5 3.0 2.5 2.0 1.5 1.0 0.5 0.0
f1 (ppm)

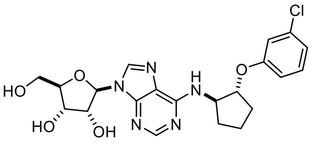
Cl
O
HO
O
N
N
H
N
HO
OH
N
N

29

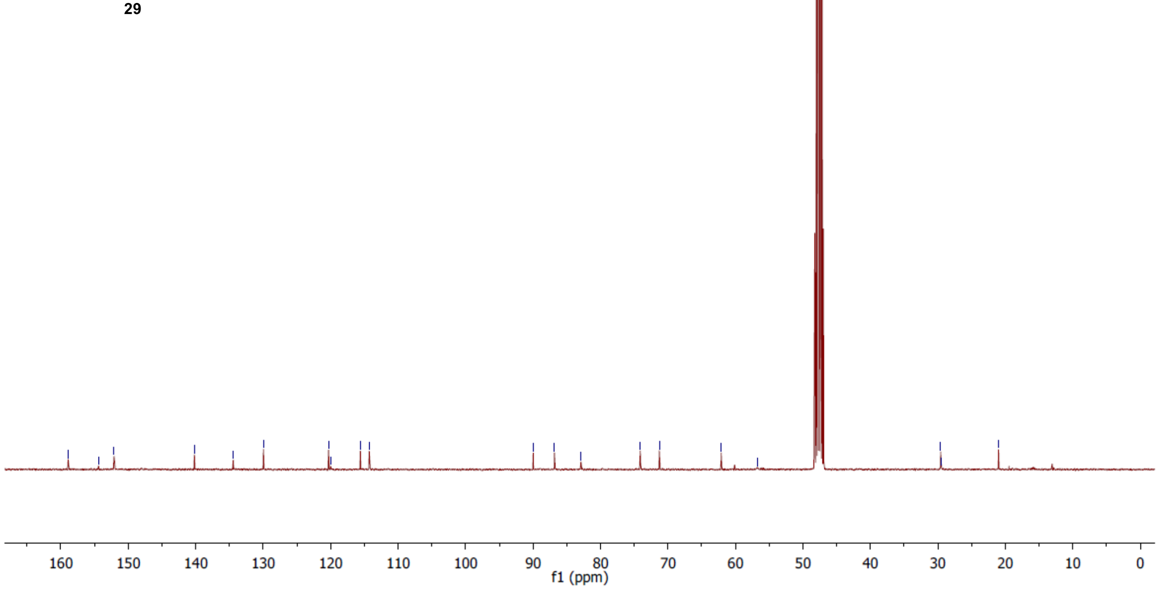
158.90
154.40
152.11
140.19
134.43
129.96
120.31
120.01
115.59
114.28
89.97
86.83
82.90
74.10
71.29
62.11
56.77
29.58
29.47
21.01
160 150 140 130 120 110 100 90 80 70 60 50 40 30 20 10 0
f1 (ppm)

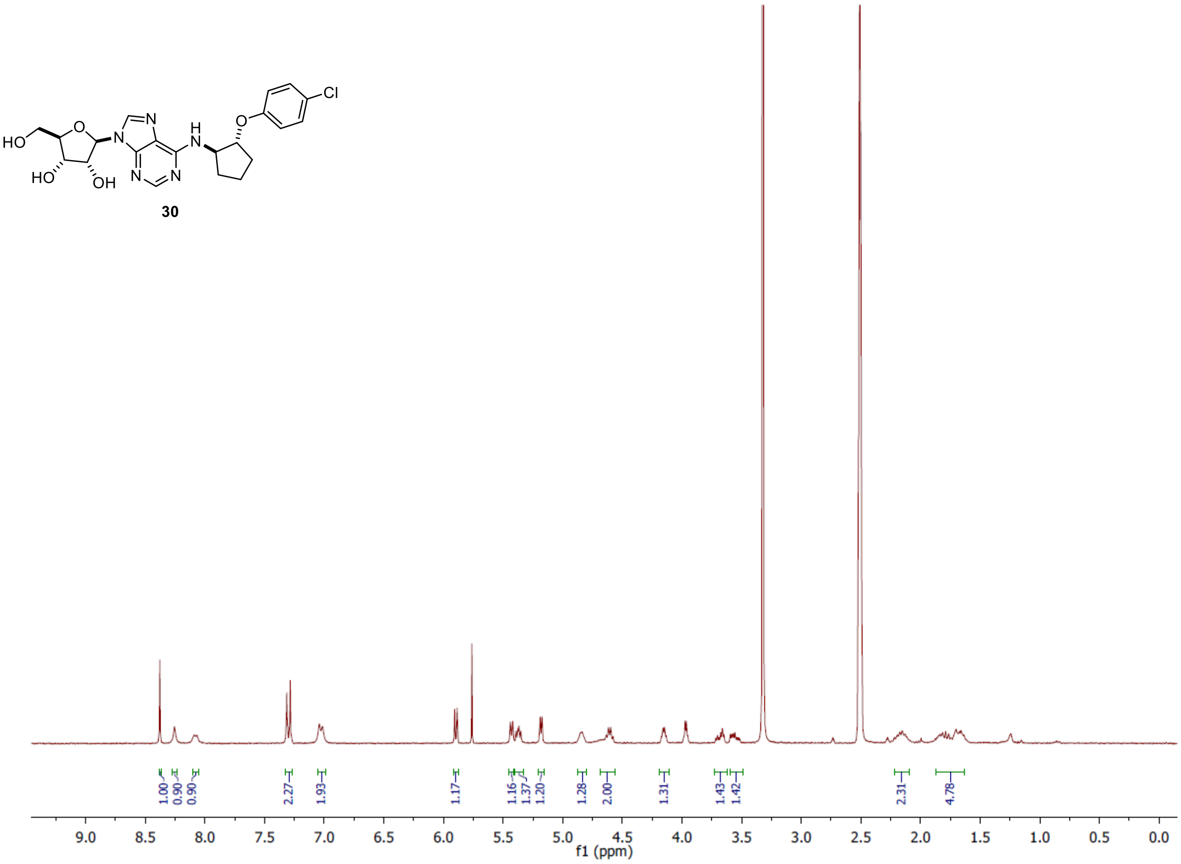
HO
O
N
N
N
N
H
N
O
Cl
HO
OH
30
1.00
0.90
0.90
2.27
1.93
1.17
1.16
1.37
1.20
1.28
2.00
1.31
1.43
1.42
2.31
4.78
9.0 8.5 8.0 7.5 7.0 6.5 6.0 5.5 5.0 4.5 4.0 3.5 3.0 2.5 2.0 1.5 1.0 0.5 0.0
f1 (ppm)

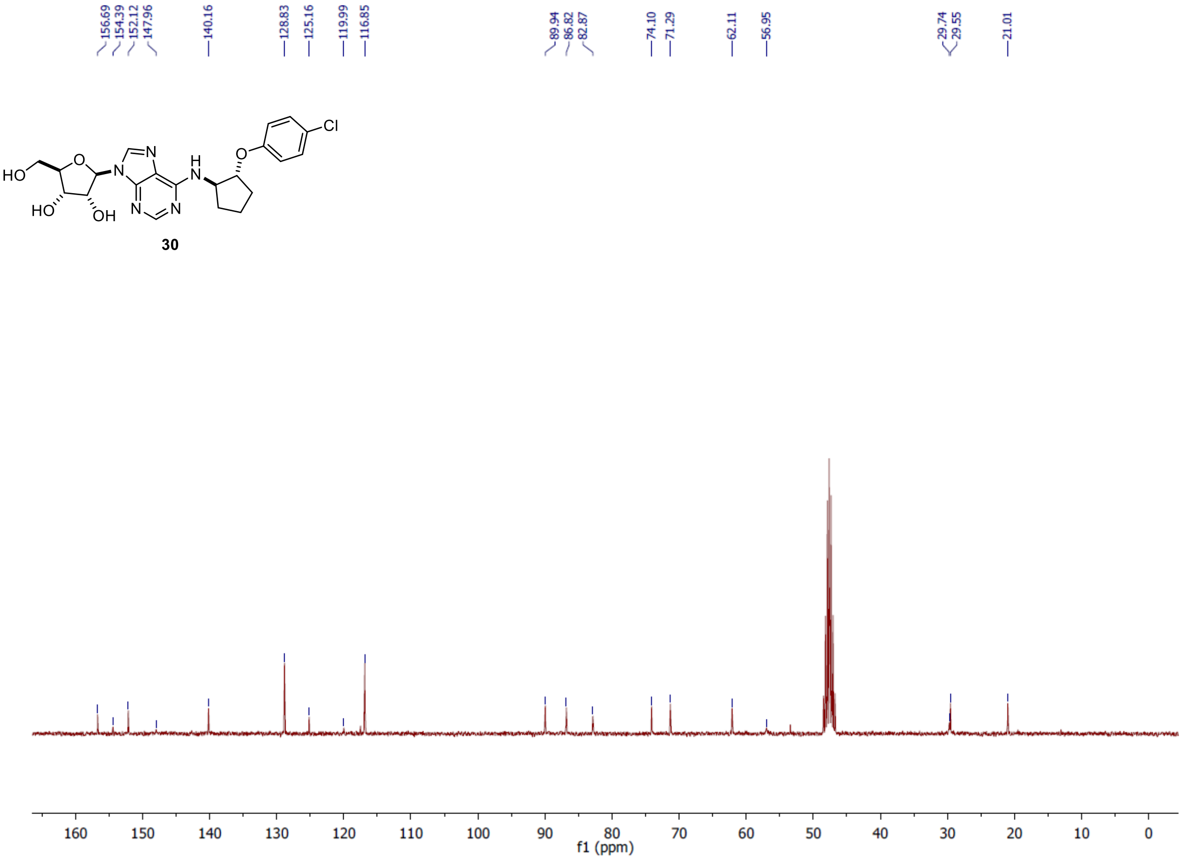
156.69
154.39
152.12
147.96
140.16
128.83
125.16
119.99
116.85
89.94
86.82
82.87
74.10
71.29
62.11
56.95
29.74
29.55
21.01
HO
O
N
N
N
N
H
N
O
Cl
HO
OH
30
160 150 140 130 120 110 100 90 80 70 60 50 40 30 20 10 0
f1 (ppm)

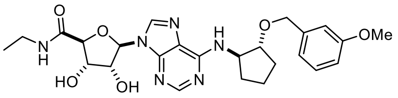
O
N
H
O
N
N
HO
OH
N
N
H
N
O
OMe

44

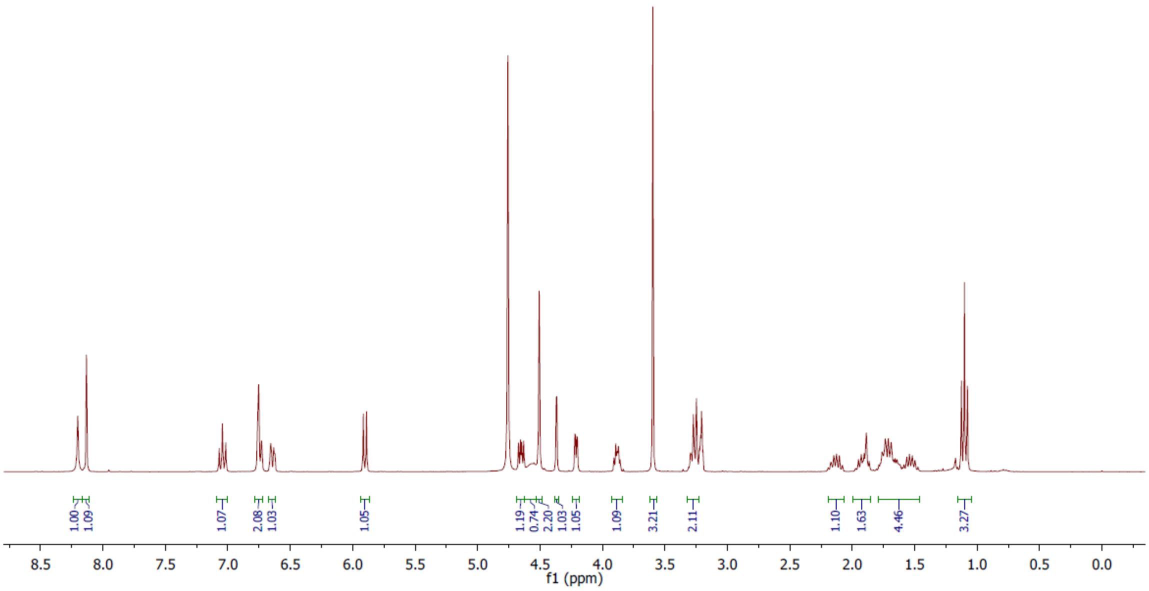
1.00
1.09
1.07
2.08
1.03
1.05
1.19
0.74
2.20
1.03
1.05
1.09
3.21
2.11
1.10
1.63
4.46
3.27
8.5 8.0 7.5 7.0 6.5 6.0 5.5 5.0 4.5 4.0 3.5 3.0 2.5 2.0 1.5 1.0 0.5 0.0
f1 (ppm)

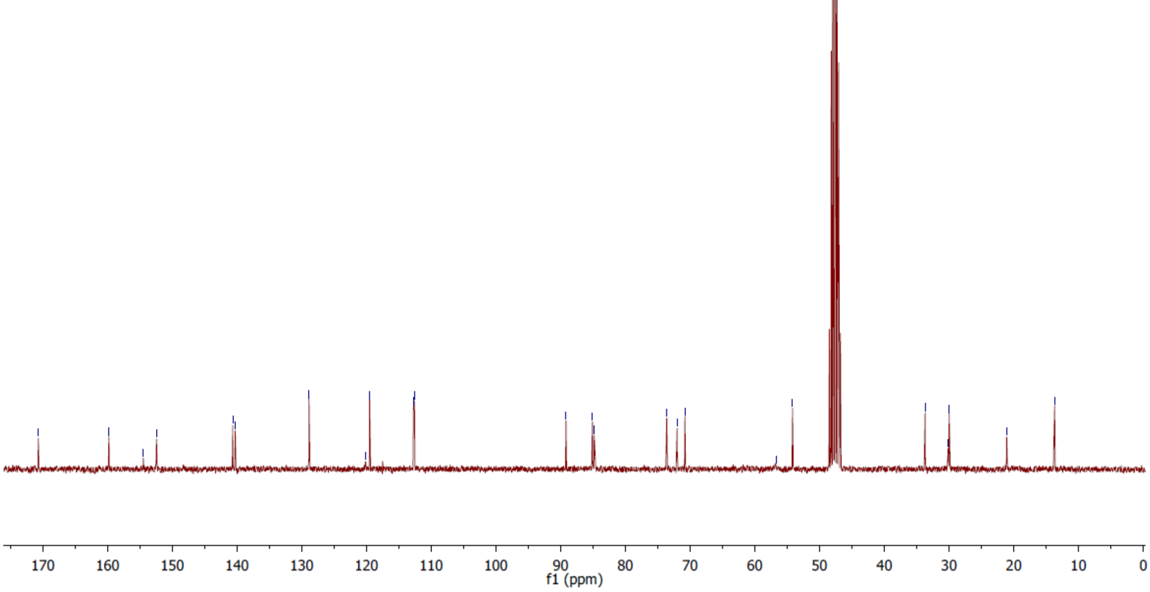
170.65
159.75
154.44
152.41
140.63
140.25
128.85
120.12
119.49
112.71
112.57
89.16
85.07
84.79
73.59
72.00
70.79
56.65
54.16
33.69
30.13
29.97
21.09
13.68
170 160 150 140 130 120 110 100 90 80 70 60 50 40 30 20 10 0
f1 (ppm)

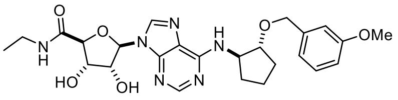
O
N
H
O
N
N
HO
OH
N
N
H
N
O
OMe

44

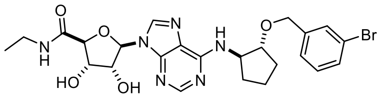
O
O
N
N
H
N
N
H
N
O
Br
HO
OH
N
N

45

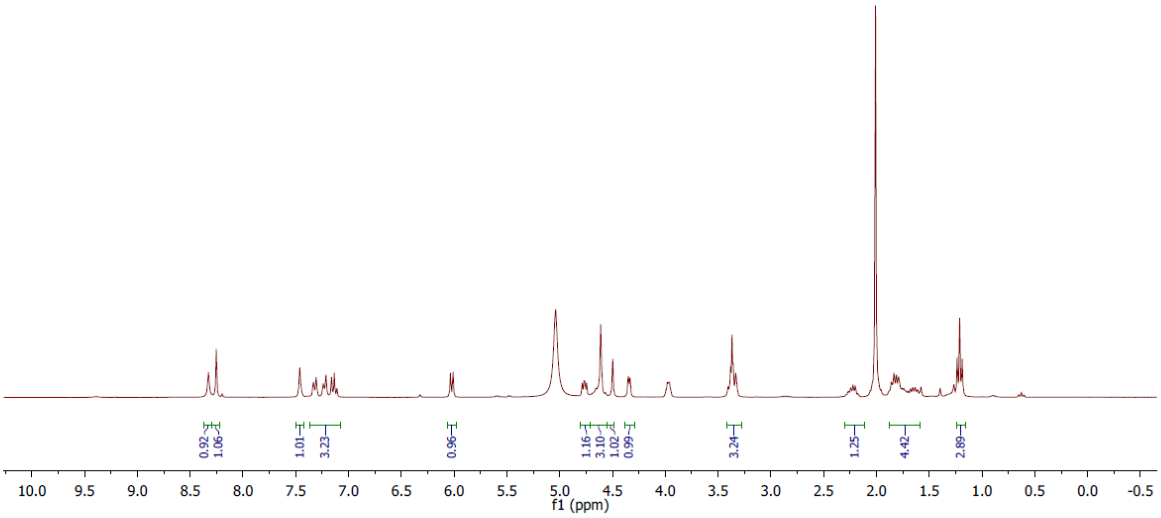
0.92
1.06
1.01
3.23
0.96
1.16
3.10
1.02
0.99
3.24
1.25
4.42
2.89
10.0 9.5 9.0 8.5 8.0 7.5 7.0 6.5 6.0 5.5 5.0 4.5 4.0 3.5 3.0 2.5 2.0 1.5 1.0 0.5 0.0 -0.5
f1 (ppm)

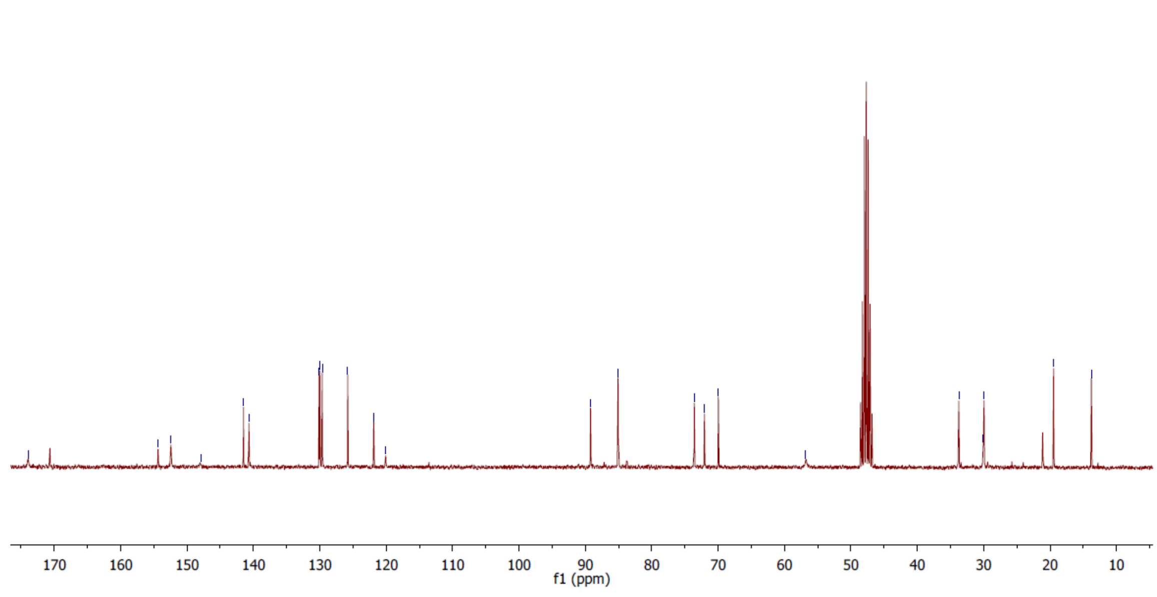
173.95
154.36
152.43
147.94
141.50
140.68
130.09
130.09
129.97
129.97
129.63
125.80
121.88
120.06
89.21
85.07
73.59
72.05
69.96
56.82
33.74
30.06
29.94
19.46
13.78
170 160 150 140 130 120 110 100 90 80 70 60 50 40 30 20 10
f1 (ppm)

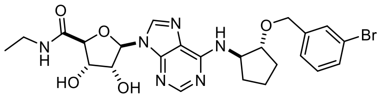
O
O
N
N
H
N
N
H
N
O
Br
HO
OH
N
N

45

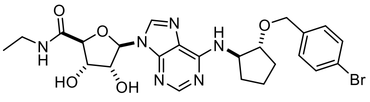
O
H
N
O
N
N
H
N
O
HO
OH
N
N
Br

46

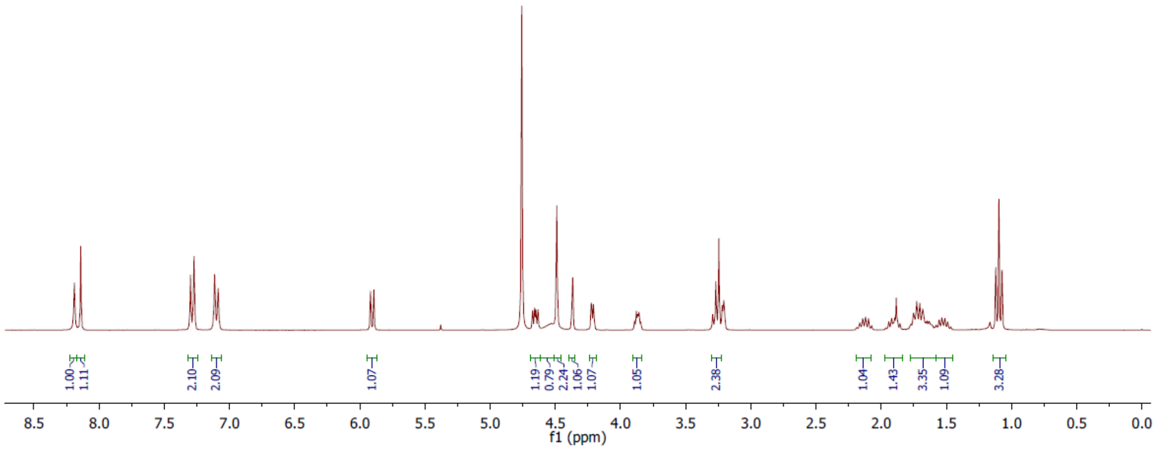
1.00
1.11
2.10
2.09
1.07
1.19
0.79
2.24
1.06
1.07
1.05
2.38
1.04
1.43
3.35
1.09
3.28
f1 (ppm)

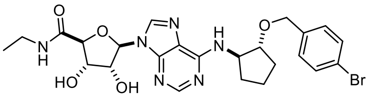
O
H
N
O
N
N
H
N
O
HO
OH
N
N
Br

46

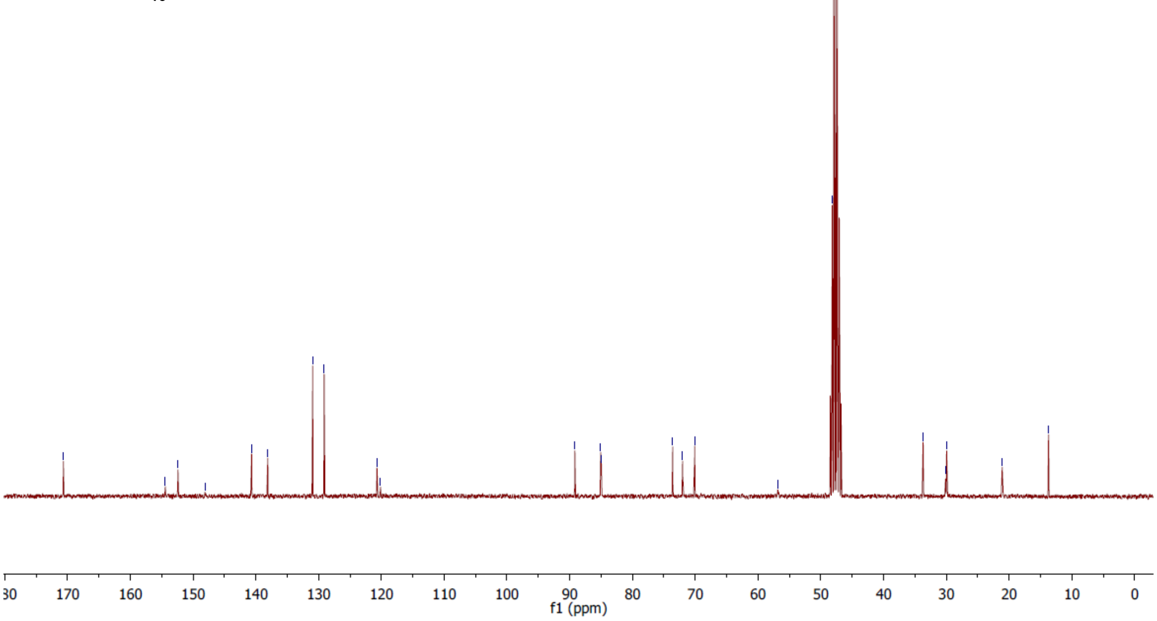
170.65
154.42
152.40
148.10
140.67
138.09
130.94
129.11
120.70
120.13
89.17
85.06
84.96
73.59
72.02
70.09
56.86
48.20
33.70
30.12
29.96
21.10
13.71
f1 (ppm)

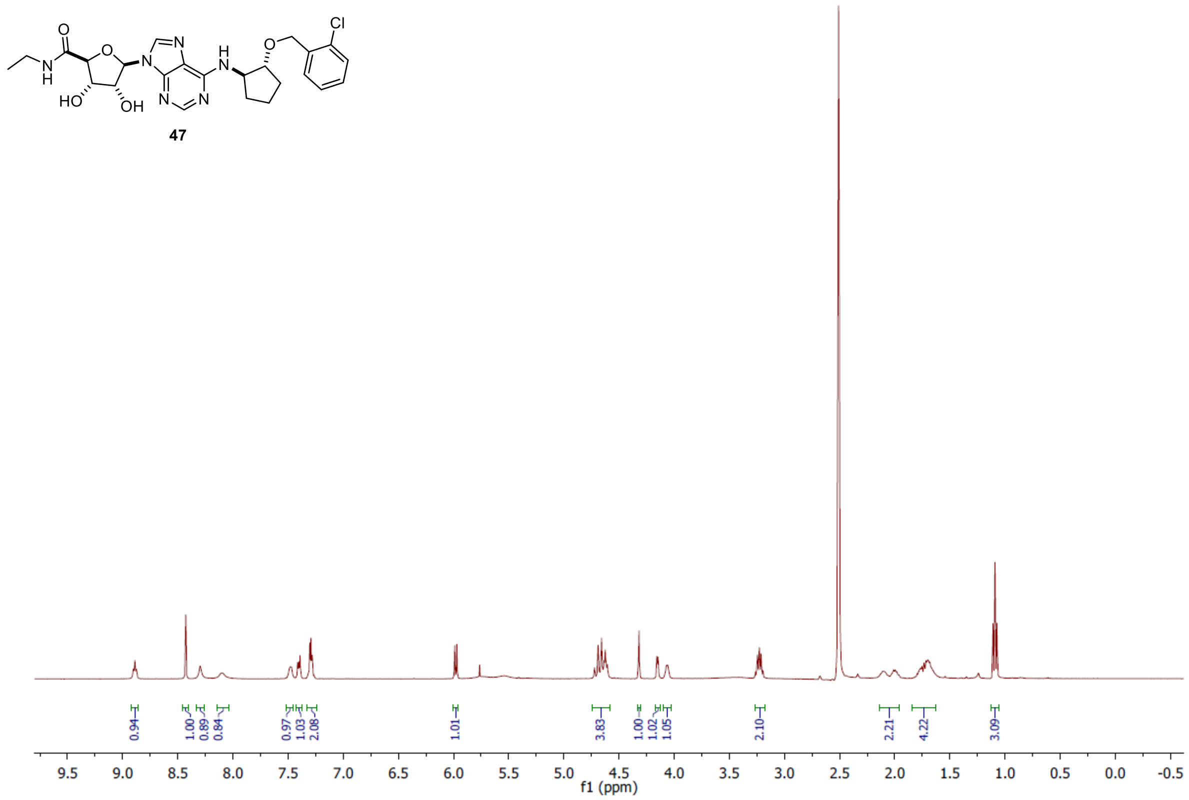
O
N
H
HO
OH
N
N
N
N
H
N
O
Cl
47
0.94
1.00
0.89
0.84
0.97
1.03
2.08
1.01
3.83
1.00
1.02
1.05
2.10
2.21
4.22
3.09
9.5 9.0 8.5 8.0 7.5 7.0 6.5 6.0 5.5 5.0 4.5 4.0 3.5 3.0 2.5 2.0 1.5 1.0 0.5 0.0 -0.5
f1 (ppm)

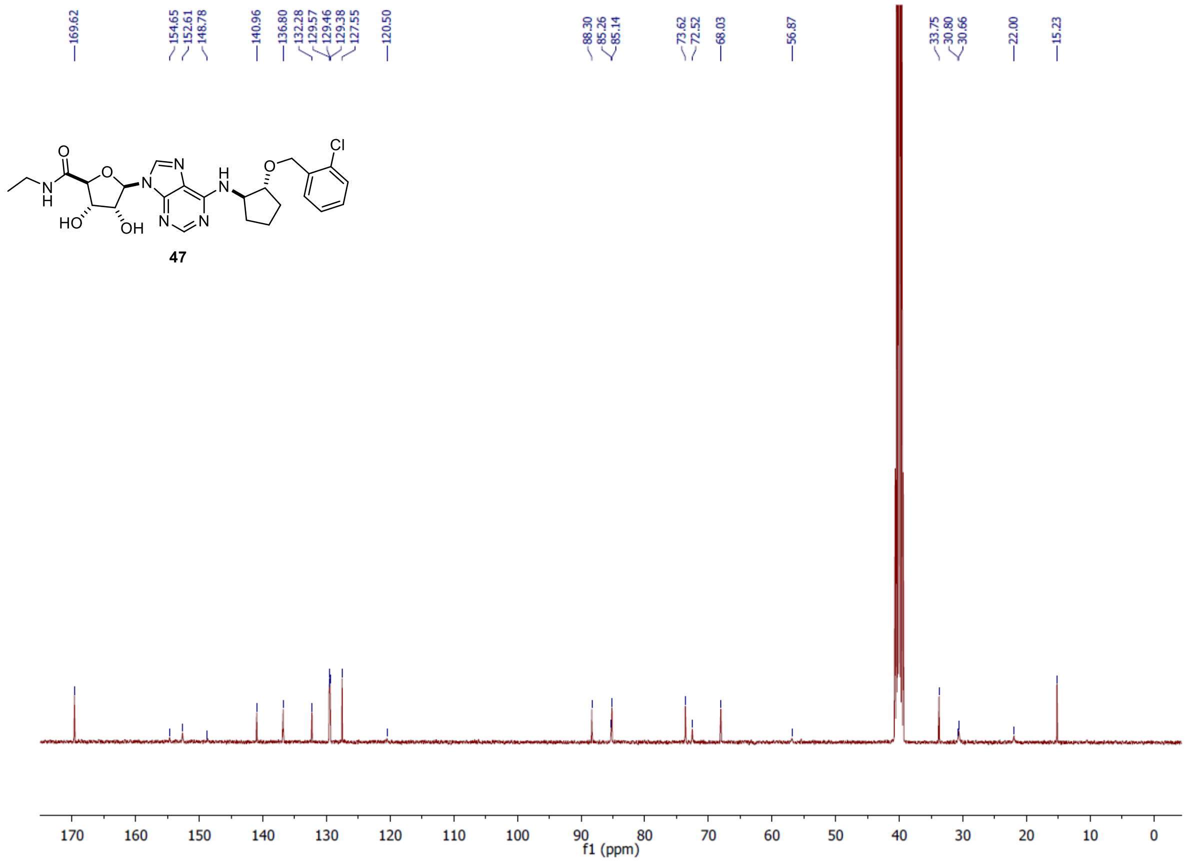
169.62
154.65
152.61
148.78
140.96
136.80
132.28
129.57
129.46
129.38
127.55
120.50
88.30
85.26
85.14
73.62
72.52
68.03
56.87
33.75
30.80
30.66
22.00
15.23
O
N
H
HO
OH
N
N
N
N
H
N
O
Cl
47
170 160 150 140 130 120 110 100 90 80 70 60 50 40 30 20 10 0
f1 (ppm)

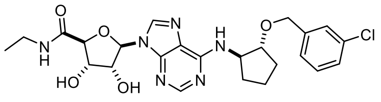

48

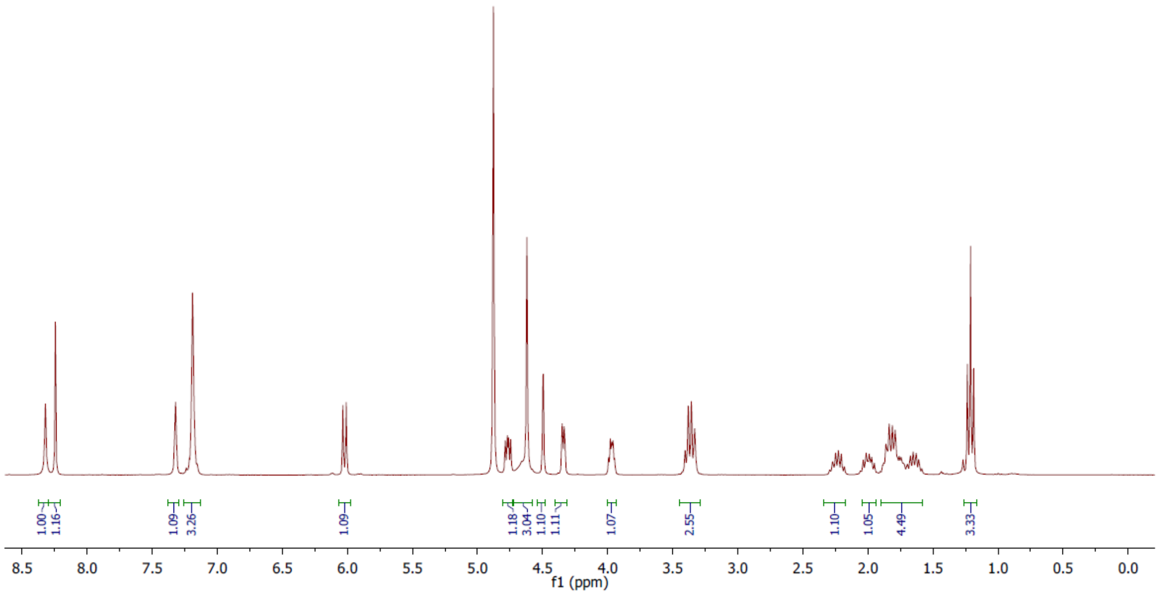
1.00
1.16
1.09
3.26
1.09
1.18
3.04
1.10
1.11
1.07
2.55
1.10
1.05
4.49
3.33
8.5 8.0 7.5 7.0 6.5 6.0 5.5 5.0 4.5 4.0 3.5 3.0 2.5 2.0 1.5 1.0 0.5 0.0
f1 (ppm)

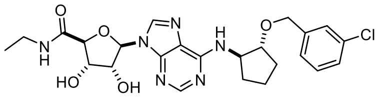

48

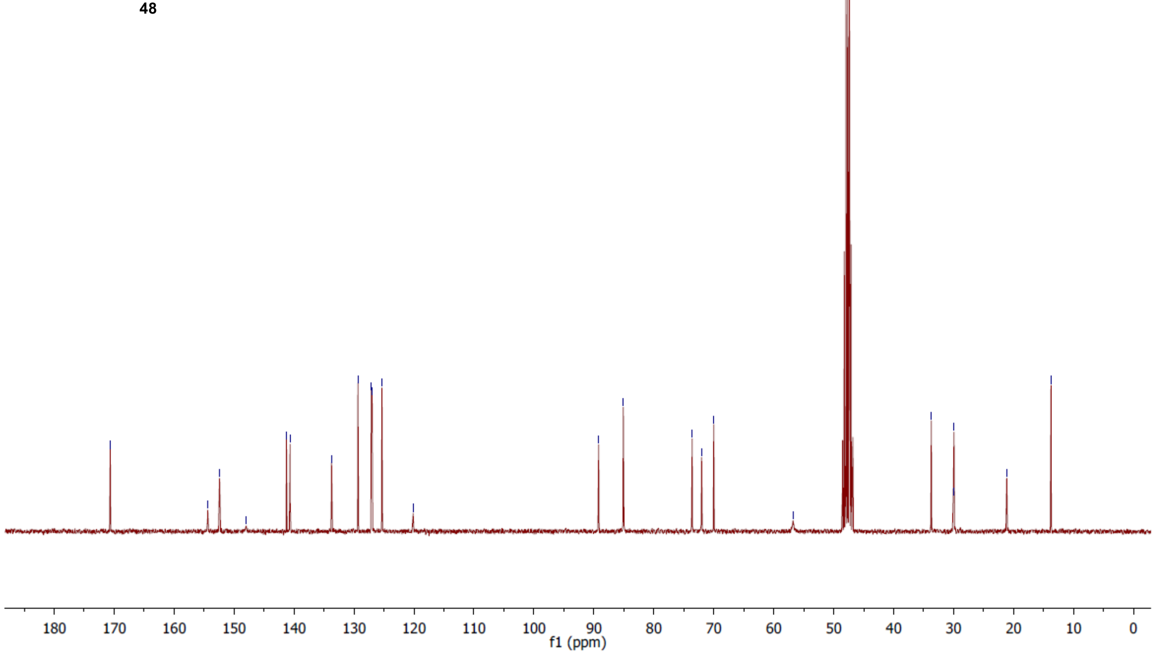
170.64
154.39
152.42
147.96
141.26
140.65
133.74
129.34
127.10
126.97
125.34
120.14
89.19
85.08
73.60
72.03
70.00
56.78
33.72
30.06
29.94
21.14
13.75
180 170 160 150 140 130 120 110 100 90 80 70 60 50 40 30 20 10 0
f1 (ppm)

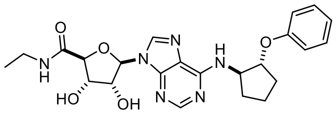

49

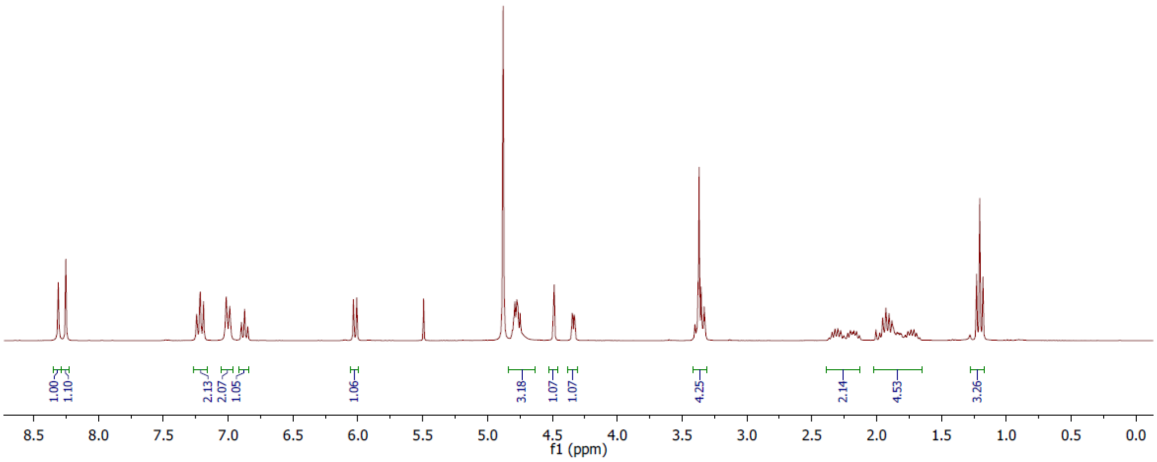
1.00
1.10
2.13
2.07
1.05
1.06
3.18
1.07
1.07
4.25
2.14
4.53
3.26
8.5 8.0 7.5 7.0 6.5 6.0 5.5 5.0 4.5 4.0 3.5 3.0 2.5 2.0 1.5 1.0 0.5 0.0
f1 (ppm)

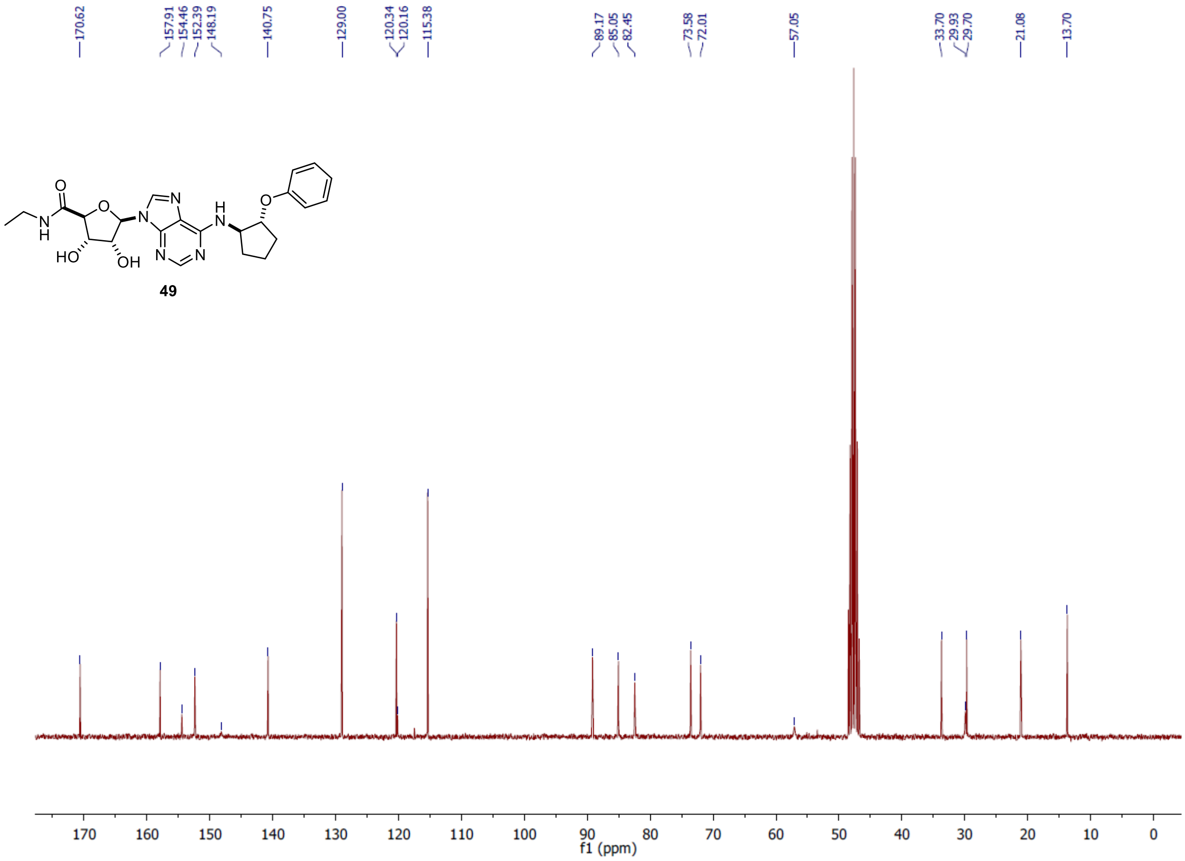
170.62
157.91
154.46
152.39
148.19
140.75
129.00
120.34
120.16
115.38
89.17
85.05
82.45
73.58
72.01
57.05
33.70
29.93
29.70
21.08
13.70
170 160 150 140 130 120 110 100 90 80 70 60 50 40 30 20 10 0
f1 (ppm)

49

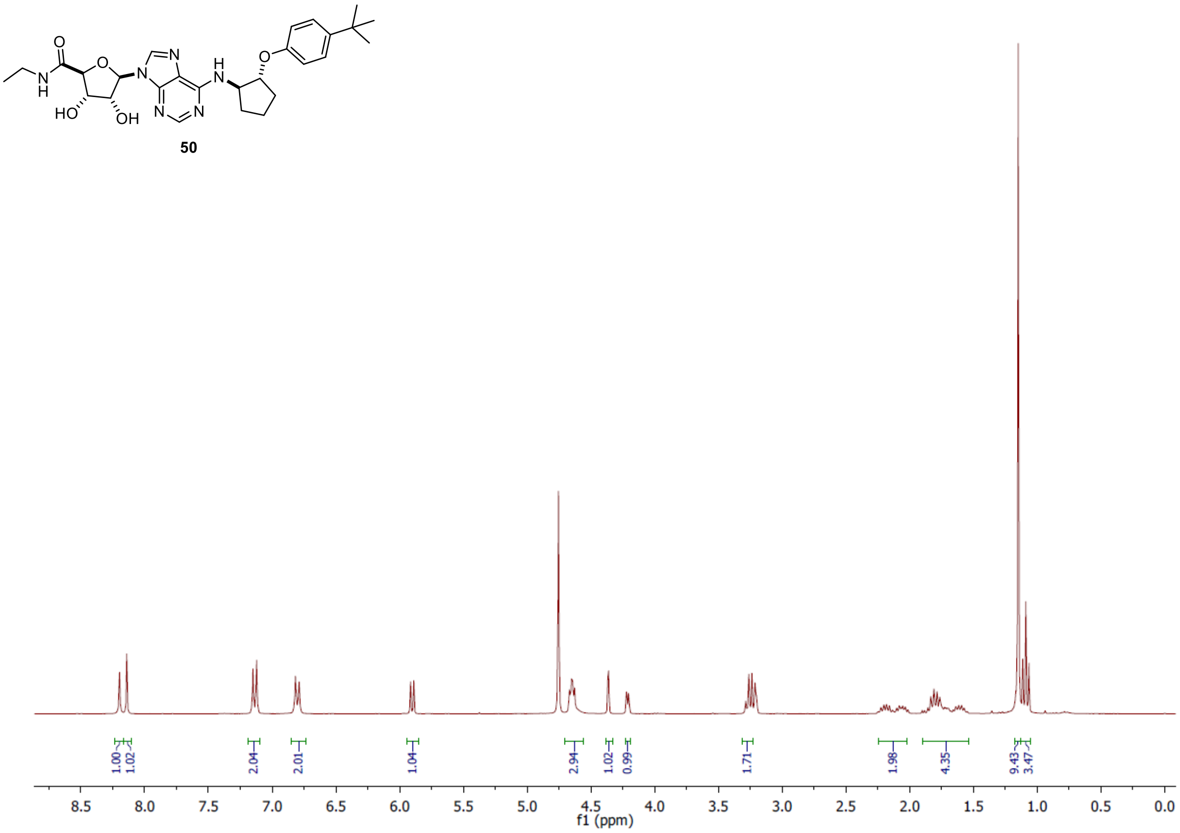

O
N
H
O
N
N
N
N
H
N
O
HO
OH
50
1.00
1.02
2.04
2.01
1.04
2.94
1.02
0.99
1.71
1.98
4.35
9.43
3.47
8.5 8.0 7.5 7.0 6.5 6.0 5.5 5.0 4.5 4.0 3.5 3.0 2.5 2.0 1.5 1.0 0.5 0.0
f1 (ppm)

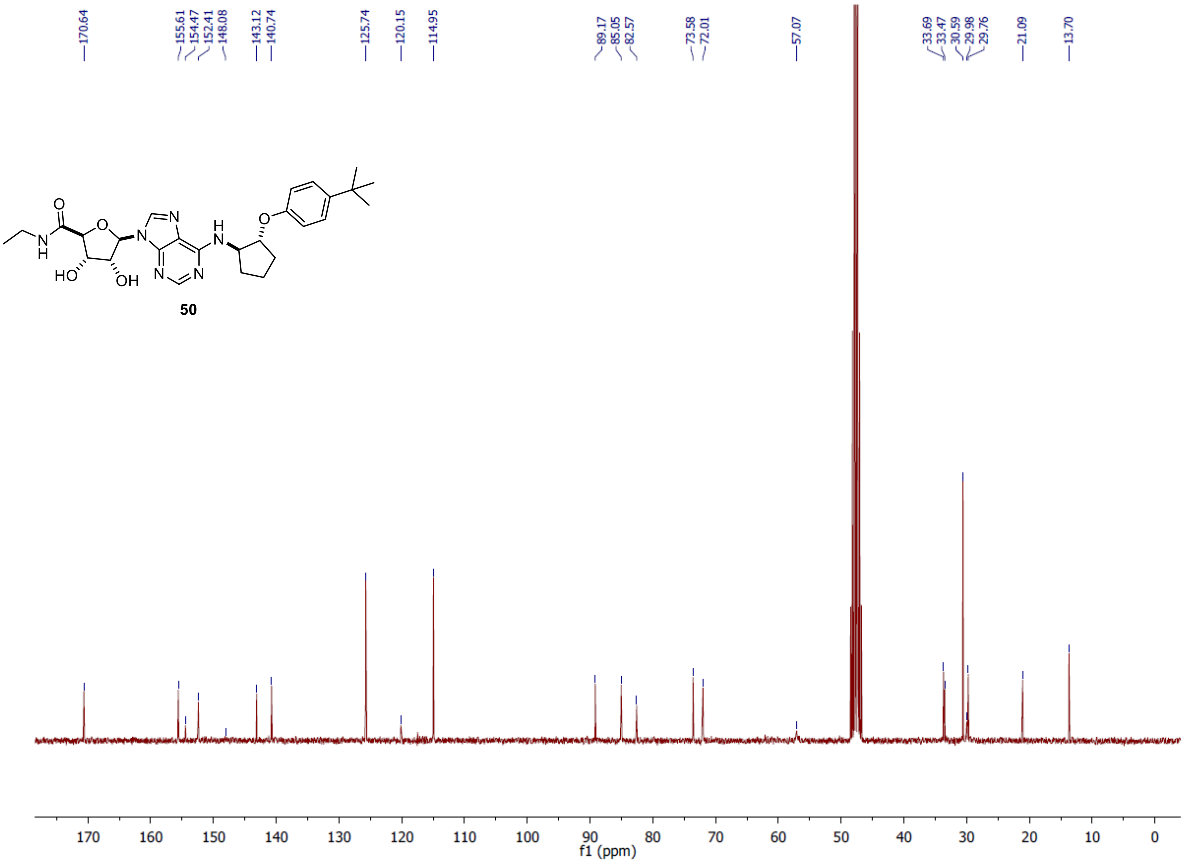

170.64
155.61
154.47
152.41
148.08
143.12
140.74
125.74
120.15
114.95
89.17
85.05
82.57
73.58
72.01
57.07
33.69
33.47
30.59
29.98
29.76
21.09
13.70
O
N
H
O
N
N
N
N
H
N
O
HO
OH
50
170 160 150 140 130 120 110 100 90 80 70 60 50 40 30 20 10 0
f1 (ppm)

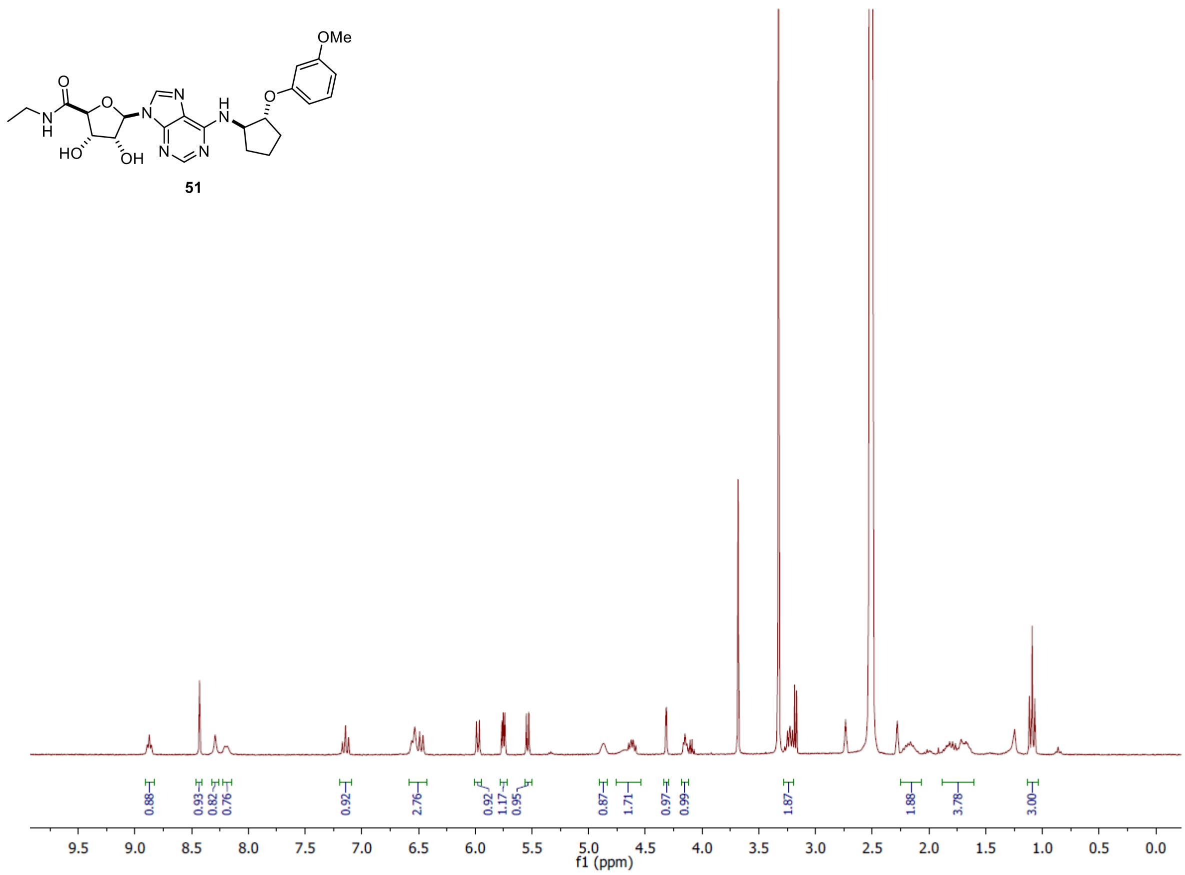
OMe
51
0.88
0.93
0.82
0.76
0.92
2.76
0.92
1.17
0.95
0.87
1.71
0.97
0.99
1.87
1.88
3.78
3.00
9.5 9.0 8.5 8.0 7.5 7.0 6.5 6.0 5.5 5.0 4.5 4.0 3.5 3.0 2.5 2.0 1.5 1.0 0.5 0.0
f1 (ppm)

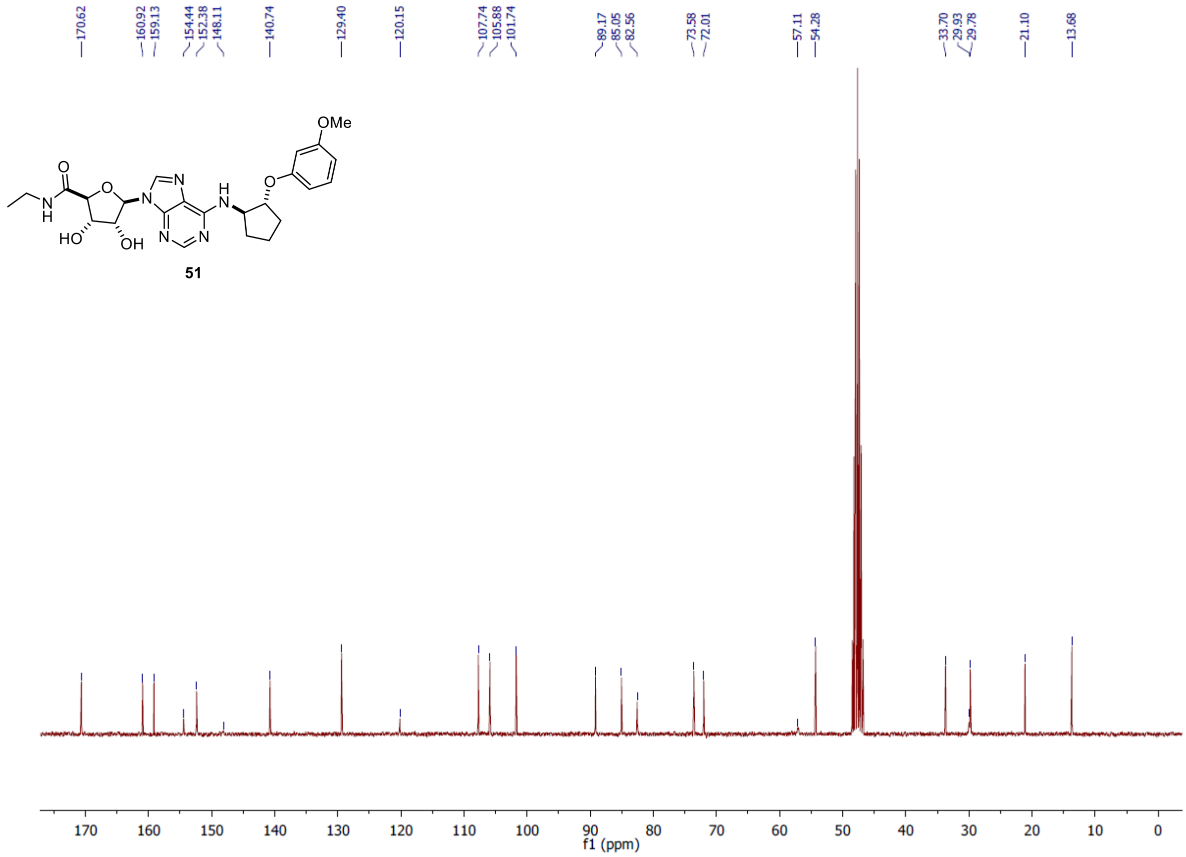
170.62
160.92
159.13
154.44
152.38
148.11
140.74
129.40
120.15
107.74
105.88
101.74
89.17
85.05
82.56
73.58
72.01
57.11
54.28
33.70
29.93
29.78
21.10
13.68
OMe
51
170 160 150 140 130 120 110 100 90 80 70 60 50 40 30 20 10 0
f1 (ppm)

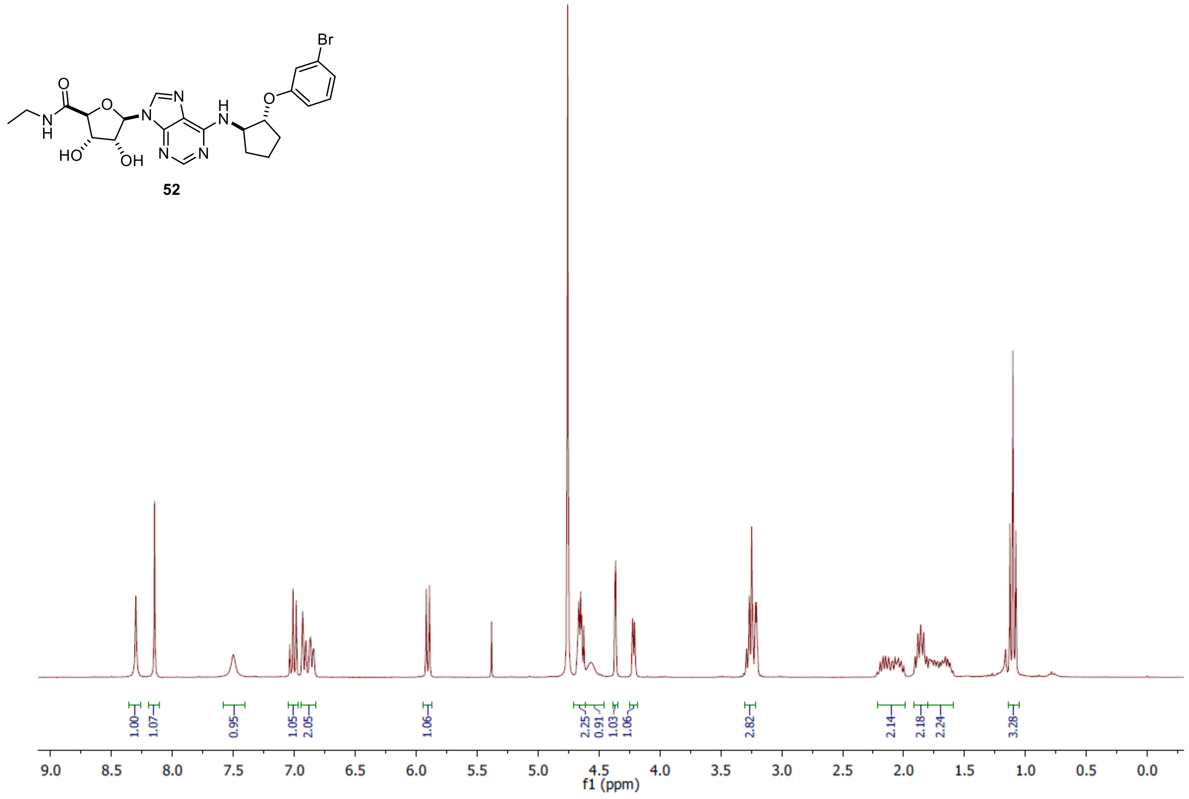
Br
O
N
N
H
O
HO
OH
52
1.00
1.07
0.95
1.05
2.05
1.06
2.25
0.91
1.03
1.06
2.82
2.14
2.18
2.24
3.28
f1 (ppm)

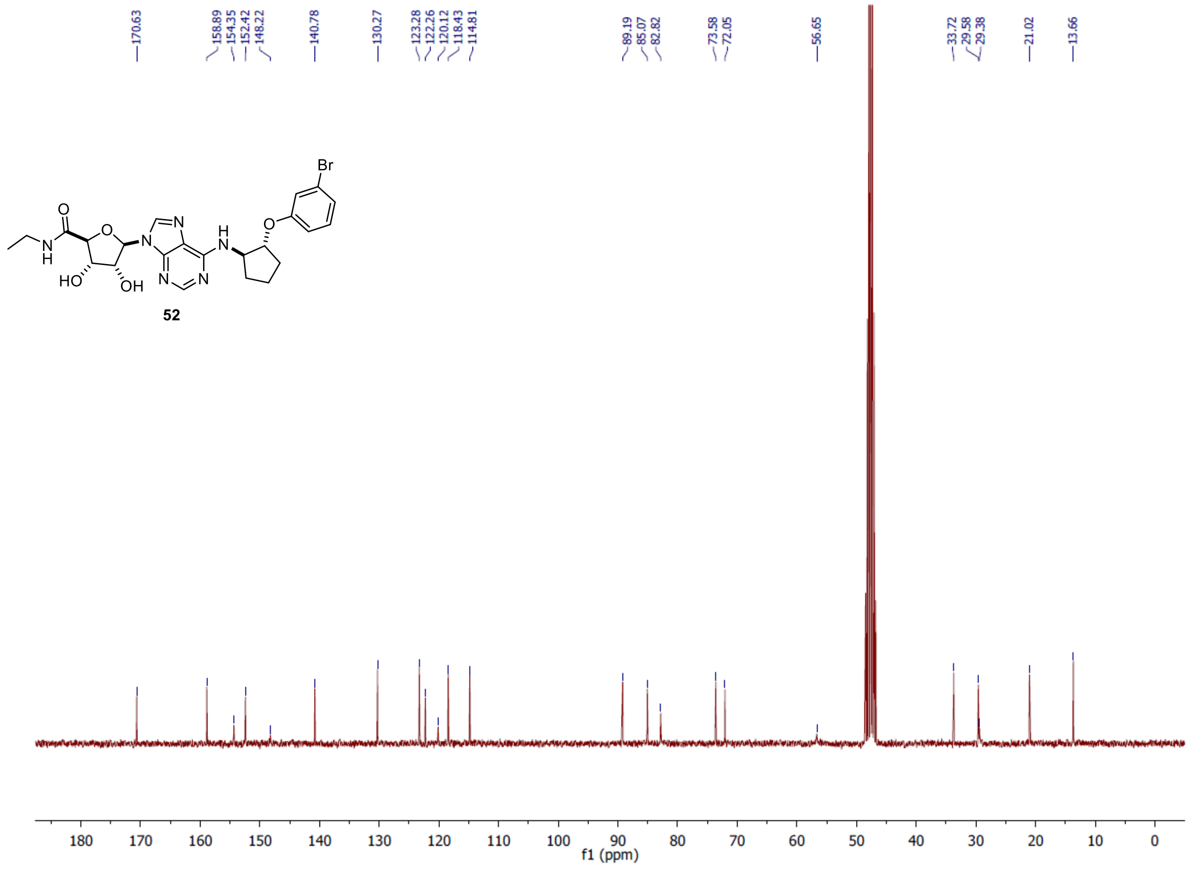
170.63
158.89
154.35
152.42
148.22
140.78
130.27
123.28
122.26
120.12
118.43
114.81
89.19
85.07
82.82
73.58
72.05
56.65
33.72
29.58
29.38
21.02
13.66
Br
O
N
N
H
O
HO
OH
52
f1 (ppm)

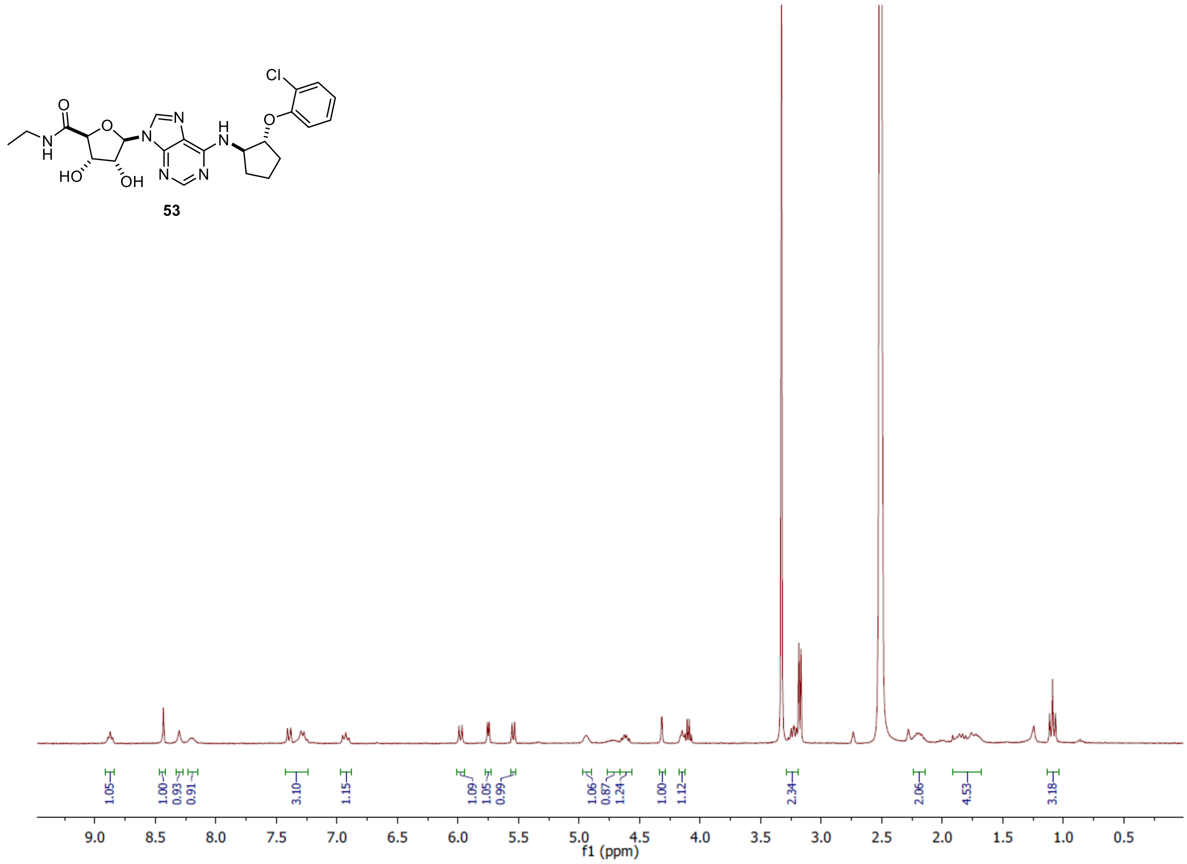
53
1.05
1.00
0.93
0.91
3.10
1.15
1.09
1.05
0.99
1.06
0.87
1.24
1.00
1.12
2.34
2.06
4.53
3.18
f1 (ppm)

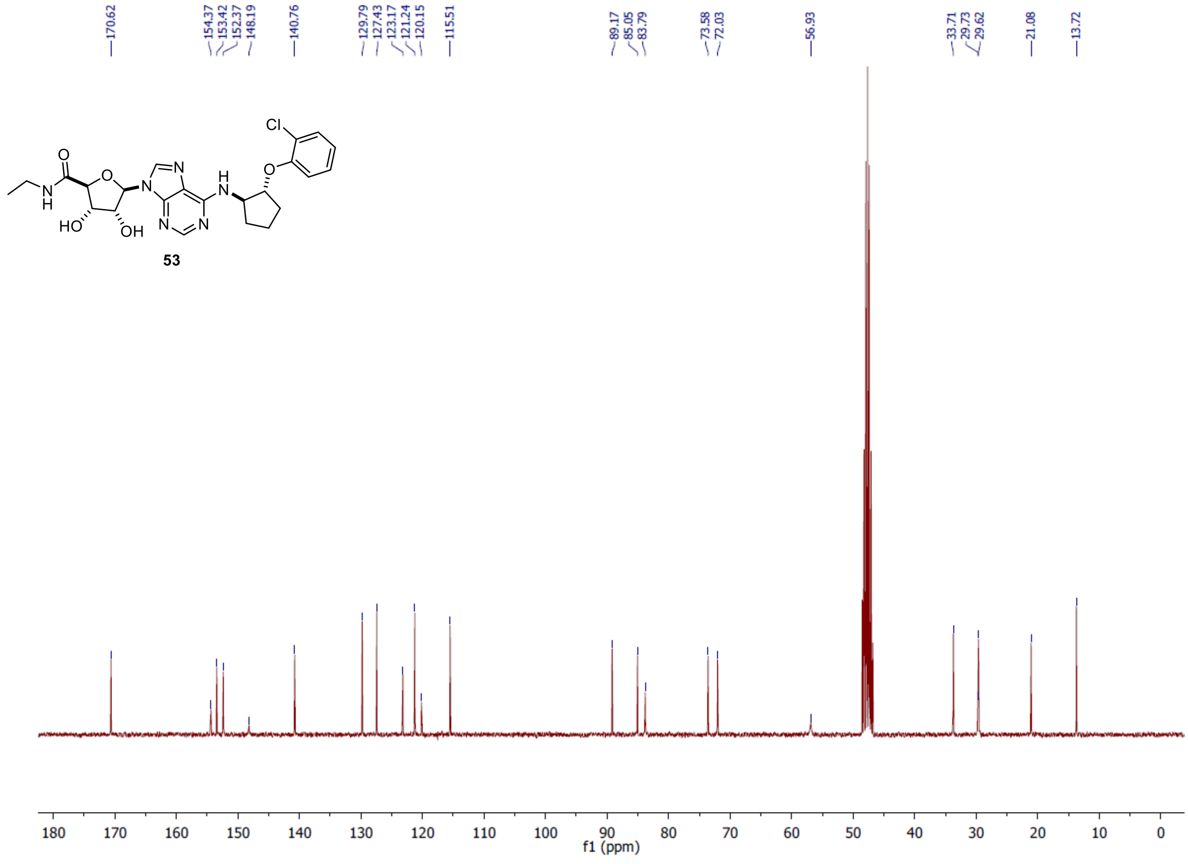
170.62
154.37
153.42
152.37
148.19
140.76
129.79
127.43
123.17
121.24
120.15
115.51
89.17
85.05
83.79
73.58
72.03
56.93
33.71
29.73
29.62
21.08
13.72
53
f1 (ppm)

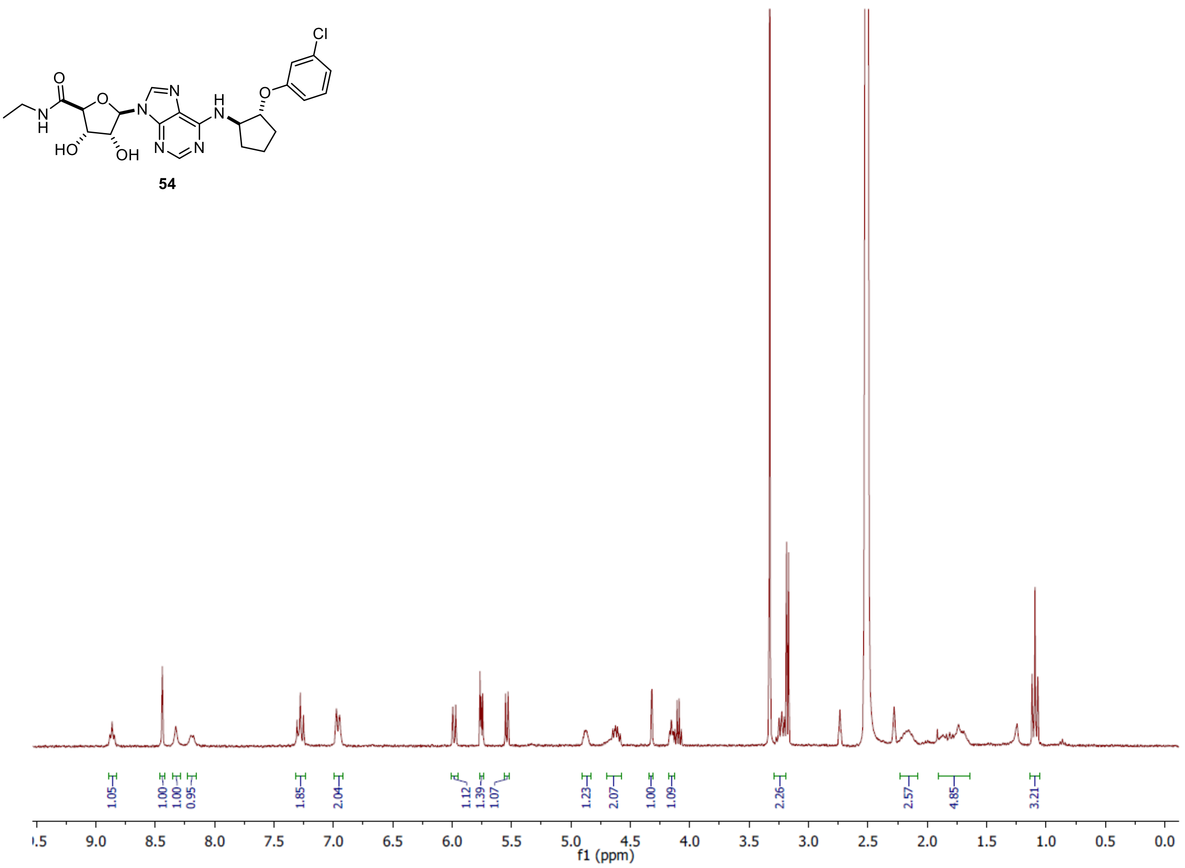
54
1.05
1.00
1.00
0.95
1.85
2.04
1.12
1.39
1.07
1.23
2.07
1.00
1.09
2.26
2.57
4.85
3.21
f1 (ppm)

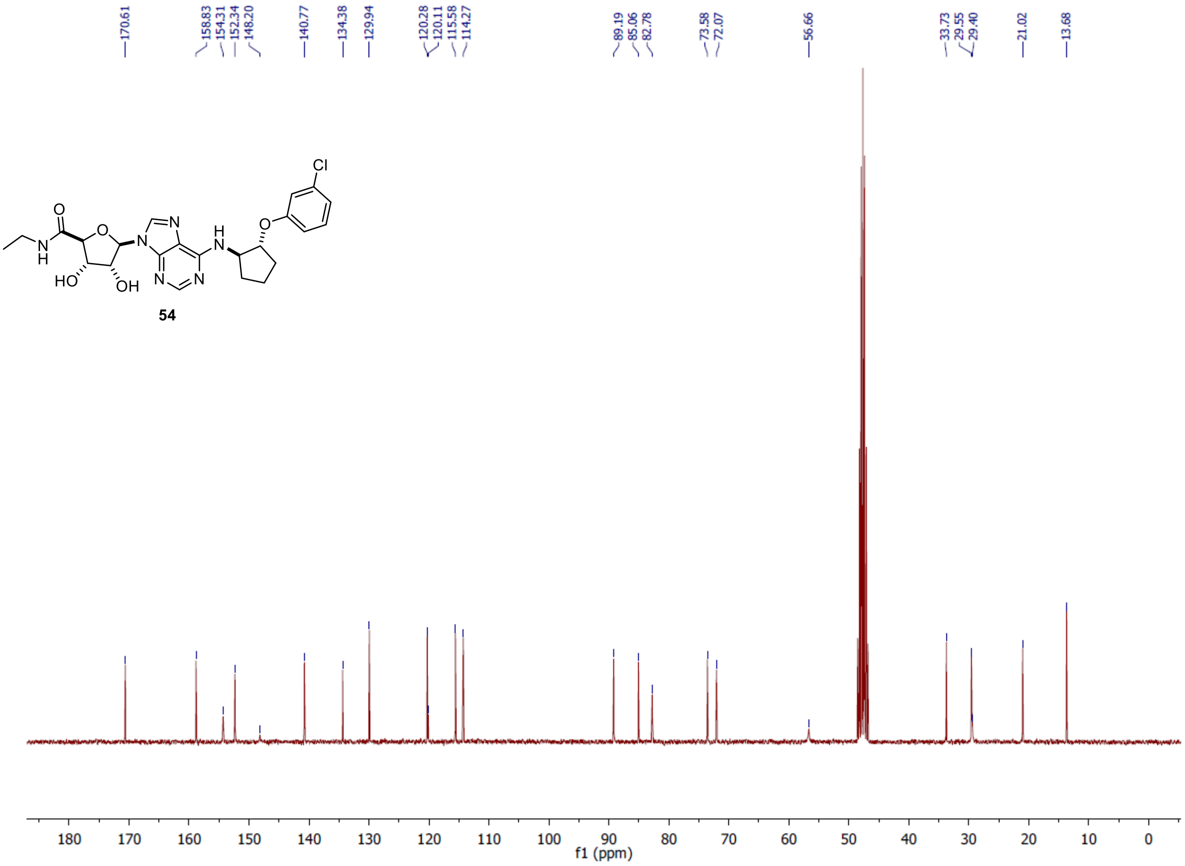
170.61
158.83
154.31
152.34
148.20
140.77
134.38
129.94
120.28
120.11
115.58
114.27
89.19
85.06
82.78
73.58
72.07
56.66
33.73
29.55
29.40
21.02
13.68
54
f1 (ppm)

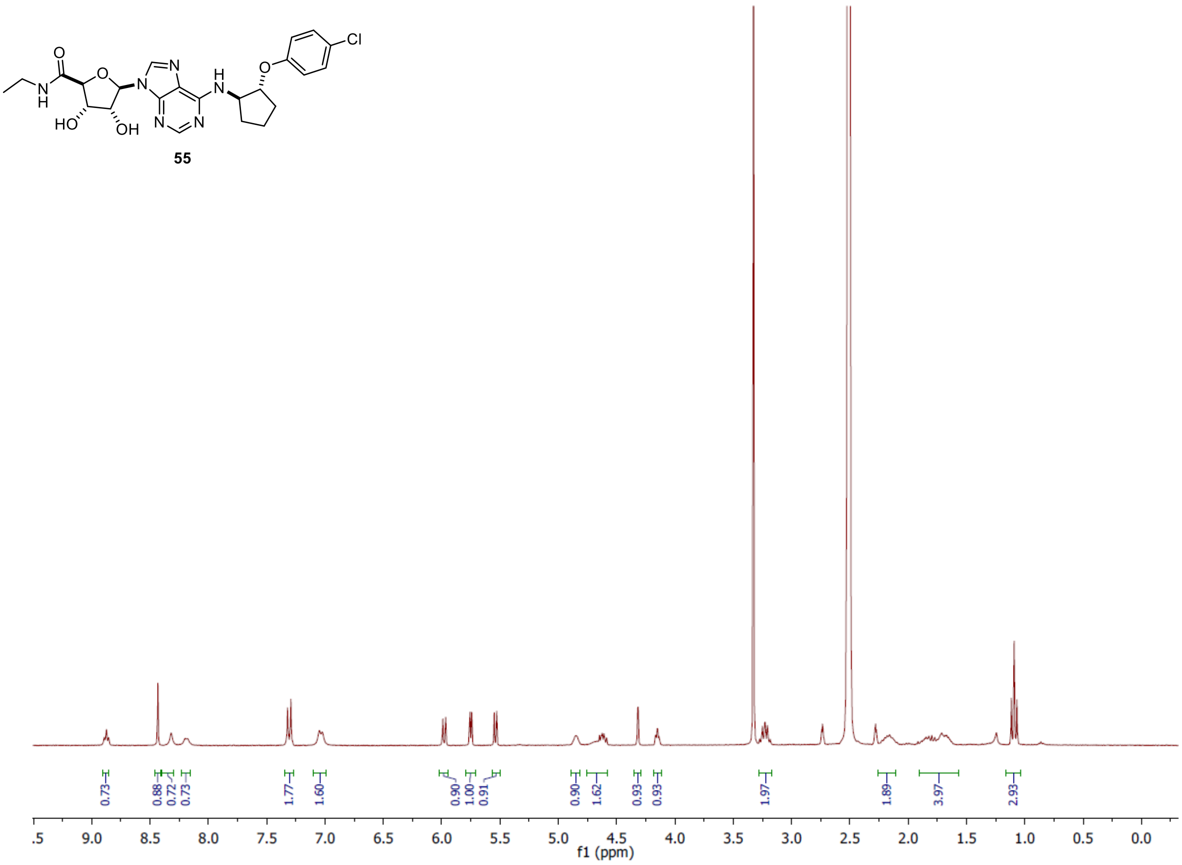

55
Cl
0.73
0.88
0.72
0.73
1.77
1.60
0.90
1.00
0.91
0.90
1.62
0.93
0.93
1.97
1.89
3.97
2.93
.5 9.0 8.5 8.0 7.5 7.0 6.5 6.0 5.5 5.0 4.5 4.0 3.5 3.0 2.5 2.0 1.5 1.0 0.5 0.0
f1 (ppm)

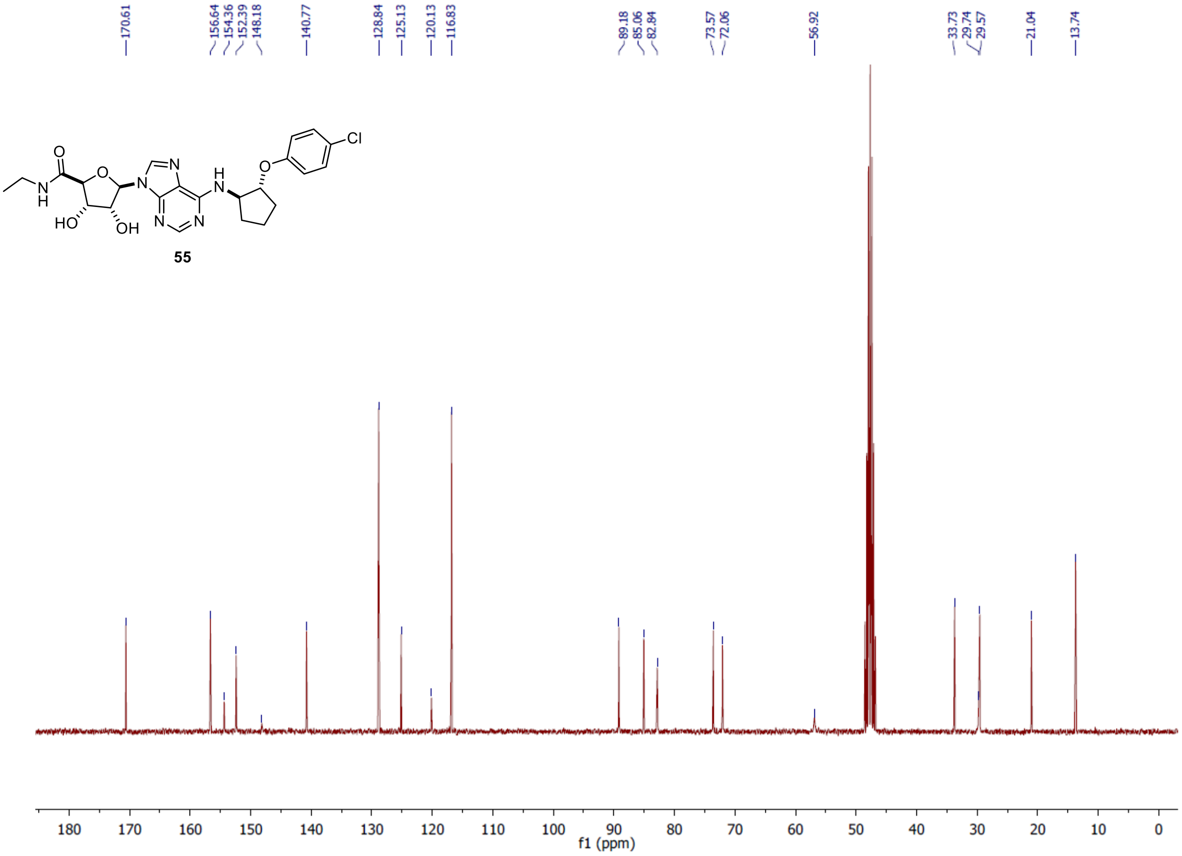

170.61
156.64
154.36
152.39
148.18
140.77
128.84
125.13
120.13
116.83
89.18
85.06
82.84
73.57
72.06
56.92
33.73
29.74
29.57
21.04
13.74
55
Cl
180 170 160 150 140 130 120 110 100 90 80 70 60 50 40 30 20 10 0
f1 (ppm)
